# Efficient Synthesis of Core-Fluorinated BODIPY-3,5-Diamides

**DOI:** 10.3390/ijms26104484

**Published:** 2025-05-08

**Authors:** Victoria E. Shambalova, Sofiya R. Mikheeva, Alexander S. Aldoshin, Anna A. Moiseeva, Evgeniya A. Safonova, Yulia G. Gorbunova, Valentine G. Nenajdenko

**Affiliations:** 1 Department of Chemistry, M. V. Lomonosov Moscow State University, Leninskie Gory 1, Building 3, Moscow 119991, Russia; 2 Frumkin Institute of Physical Chemistry and Electrochemistry, Russian Academy of Sciences, Leninsky pr., 31, Building 4, Moscow 119071, Russia; 3 Kurnakov Institute of General and Inorganic Chemistry, Russian Academy of Sciences, Leninsky pr., 31, Moscow 119991, Russia

**Keywords:** nitrostyrene, pyrrole, fluorine, BODIPY, fluorophore, photosensitizer

## Abstract

A modular synthesis of a new family of core-fluorinated BODIPYs was elaborated. *β*-Fluoro-*β*-nitrostyrenes were used as starting materials to prepare a set of mono-fluorinated pyrrole-2-amides using the Barton-Zard reaction with 2-isocyanoacetamides. The prepared monofluorinated building blocks were successively transformed into the corresponding dipyrromethanes and 1,7-difluoro-BODIPY-3,5-diamides. As a result, a new family of luminophores with a combination of fluorescence and photosensitizing properties was obtained. The photophysical properties of novel BODIPYs were studied by UV-vis and fluorescence spectroscopy, cyclic voltammetry and DFT calculations. In addition, their ability to generate singlet oxygen was assessed. The properties of 1,7-difluoro-BODIPY-3,5-diamides were compared with their analogues to reveal the substituent effect at the 3,5-positions. The fluorescent properties of the obtained dyes were significantly improved in comparison to their 3,5-diester analogous.

## 1. Introduction

Boron-dipyrromethenes (BODIPYs) are an important class of pyrrole-derived organic dyes [1,2,3,4]. The properties of BODIPYs are strictly related to their structure. Therefore, structural modification of BODIPY cores has been studied for tuning their photophysical and electrochemical properties for different applications [5,6,7,8,9,10]. As a result, this class of dyes has found a wide application as fluorescent markers for bioimaging [11,12], agents for photoacoustic imaging [13], fluorescent sensors [14,15], triplet photosensitizers for photodynamic therapy, photoredox catalysis [16,17,18,19,20] and solar energy conversion [21], in materials for optoelectronic devices [22], electro- and chemiluminescence [23], drug delivery [24], etc.

On the other hand, the introduction of fluorine into organic chromophores is a very powerful tool that can allow their significant improvement in different applications. Indeed, fluorination of dyes affects their electronic and structural properties and as a consequence their photophysical characteristics [25]. In addition, the replacement of potentially oxidizable C-H bonds with stronger C-F bonds can increase the chemical and photostability of fluorinated dyes. Indeed, fluorination of some classical dyes has improved their properties [26]. For example, enhanced photostability and lower pKa have been observed for fluorinated fluorescein, known as Oregon green, compared to the parent fluorescein [27,28]. Fluorinated derivatives of Rhodamine dyes have demonstrated enhanced resistance to photobleaching [29]. In turn, significant improvements in power output and photostability have been achieved for classical laser dyes even by non-selective electrophilic fluorination [30].

However, the preparation of core-fluorinated BODIPYs is not a trivial task. This is because late-stage fluorination of the BODIPY core is very challenging. Indeed, to our best knowledge, only three compounds of this type have been reported by electrophilic fluorination of the BODIPY core with Selectfluor [31]. The described approach was limited to introducing only one fluorine into the structure in a very low yield. Nevertheless, fluorination at the 2-position has considerably enhanced the photostability of BODIPY.

**Figure 1:**
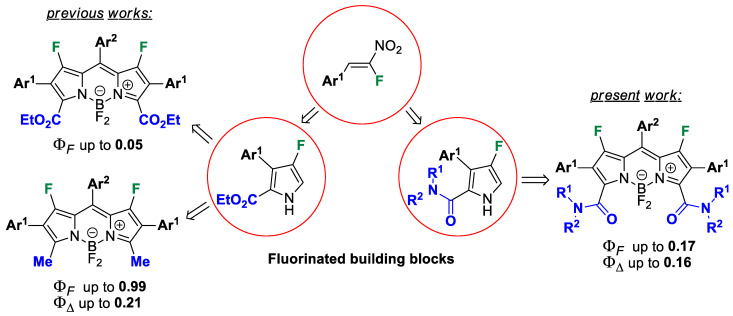
Families of core-fluorinated BODIPYs synthesized starting from *β*-fluoro-*β*-nitrostyrenes.

Recently we have initiated a new project aimed at the preparation of core-fluorinated BODIPYs via an alternative approach using fluorinated building blocks. Such a very convenient constructive approach would allow us to install fluorine atoms at the strictly specified position of the dyes. As a result, *β*-fluoro-*β*-nitrostyrenes [32,33] were found to be suitable building blocks for constructing 3-aryl-4-fluoro-1*H*-pyrrole-2-carboxylates [34], which were subsequently converted into two families of novel 1,7-difluorinated BODIPYs (Figure 1) [35,36,37]. However, diester-derived BODIPYs exhibited poor fluorescent properties.

We supposed that the replacement of ethoxycarbonyl groups with more rotationally restricted amide ones could improve the fluorescence properties of this type of dye (Figure 1). The corresponding rotational barrier for the ester group is considerably lower than the amide one [38,39]. Indeed, BODIPY-2,6- [40,41,42] and 3,5-diamides [43] have demonstrated much better fluorescence properties and have been studied as fluorescent probes for imaging living cells and detection of hypochlorous acid [44,45] and peroxynitrite anion [46].

This work is devoted to the modular synthesis of a new family of 1,7-difluoro-BODIPY-3,5-diamide from *β*-fluoro-*β*-nitrostyrene and the study of their photophysical properties.

## 2. Results and Discussion

### 2.1. Synthesis

**Scheme 1:**
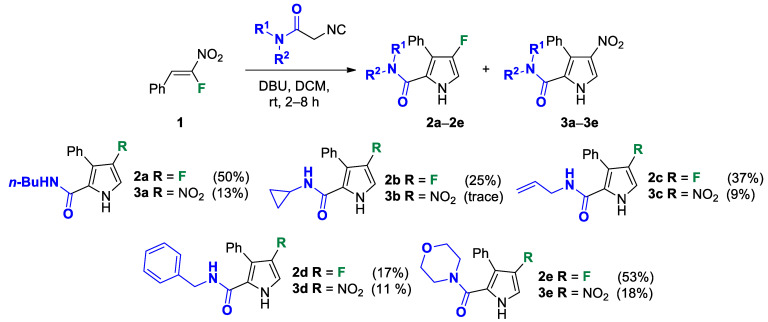
Barton-Zard reaction of *β*-fluoro-*β*-nitrostyrenes **1** with 2-isocyanoacetamides.

First, the Barton-Zard reaction of *β*-fluoro-*β*-nitrostyrene **1** with 2-isocyanoacetamides [47] was studied to prepare the corresponding monofluorinated pyrroles **2** (Scheme 1). We have found previously that the key step of this approach is aromatization to form the corresponding pyrrole via the elimination of either HF (pKa 3.17) or nitrous acid (pKa 3.4) [34]. As a result, we expected the formation of the target 4-fluoropyrroles **2** and the corresponding 4-nitropyrroles **3** as a side reaction product. It was found that the Barton-Zard reaction of *β*-fluoro-*β*-nitrostyrene **1** with 2-isocyanoacetamides afforded the desired pyrroles **2** in low to moderate yield (17–53%). As expected, less acidic 2-isocyanoacetamides proved to be less effective in terms of selectivity and product yield compared to the previously studied reaction of ethyl 2-isocyanoacetate [34]. Nevertheless, a set of pyrrole-amides **2** was prepared with up to 53% yield. In all cases, the corresponding nitro derivatives **3** were minor products easily separated from the target fluorinated pyrroles **2** by column chromatography.

**Scheme 2:**
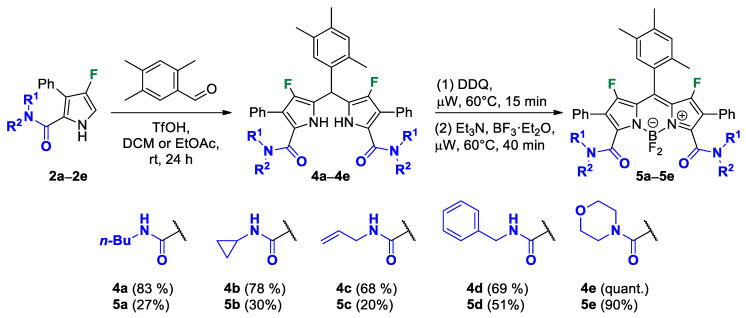
Preparation of BODIPYs **4** with different amido-groups.

Next, the synthesis of dipyrromethanes was studied. For this aim, these novel fluorinated blocks **2** were involved in the condensation with 2,4,5-trimethylbenzaldehyde in the presence of triflic acid (Scheme 2). As a result, a number of dipyrromethanes **4** were prepared in up to 100% yield. Dipyrromethanes **4** were subjected to the subsequent oxidation with DDQ followed by complexation with BF_3_∙Et_2_O. Both steps were activated by microwave irradiation at a constant temperature of 60 °C. Thus, a series of novel core-fluorinated BODIPY-3,5-diamides **5a**–**5e** with diverse amide moiety was obtained in up to 90% yield.

**Scheme 3:**
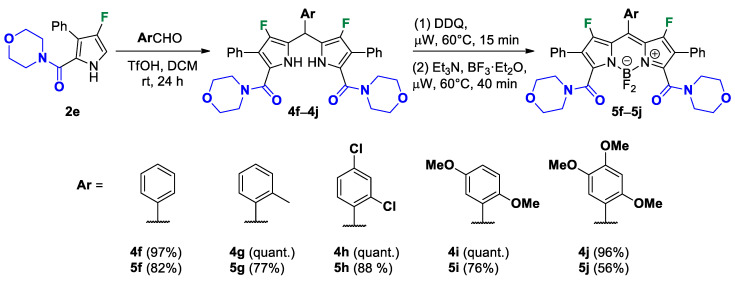
Preparation of BODIPYs **4** with different *meso*-aryl groups.

It is known that free rotation of a phenyl ring at the *meso*-position causes fluorescence quenching [48,49]. Rotationally restricted aryl substituents are favourable for fluorescence properties [50,51]. Therefore, modular synthesis of BODIPY **5g**–**5j** based on pyrrole **2e** involving other *ortho*-substituted aromatic aldehydes was carried out. BODIPY **5f** with phenyl ring at *meso*-position was also prepared to assess of *meso*-substituent effect for this structural type of BODIPY (Scheme 3).

We found that morpholine-derived pyrrole **2e** efficiently reacted with any aromatic aldehydes. Both sterically hindered and highly electron-rich aldehydes gave the corresponding dipyrromethanes **4** in quantitative yields. The subsequent oxidation and complexation afforded a series of novel core-fluorinated BODIPY-3,5-diamides **5f**–**5j** with diverse *meso*-aryl moiety in up to 88% yield. The structures of all novel intermediates and dyes were confirmed by a combination of ^1^H, ^13^C and ^19^F NMR spectroscopy and high-resolution mass-spectroscopy.

### 2.2. Photophysical Properties

Next, the photophysical properties of obtained luminophores **5** were studied. First, UV–vis absorption and fluorescence spectra were measured for BODIPY **5a** in different solvents to investigate solvatofluorochromism (Table 1, See Supplementary Materials, Figure S1).

It was found that the position of the absorption and emission maxima changes depending on the nature of the solvent. The largest bathochromic (18 nm) and bathofloral shifts (13 nm) were observed in nonpolar toluene compared to polar methanol. In addition, it was noted that fewer polar solvents are favourable for fluorescent properties (Table 1, Cf. entries 1–3 and 4–7). Thus, the best result, Φ*_F_* = 0.16, was achieved in THF (Table 1, entry 3).

**Figure 2:**
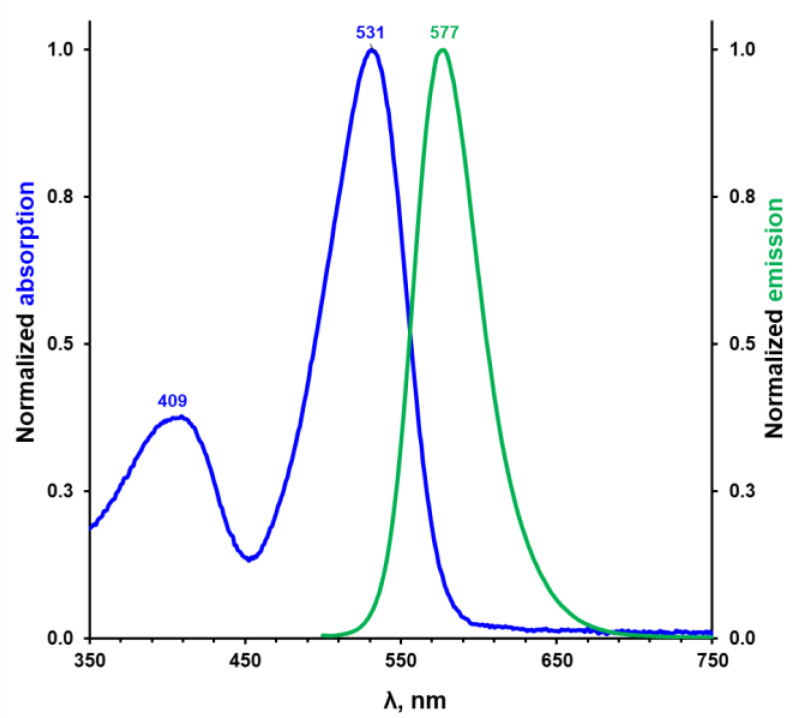
UV–vis absorption and emission spectra of solution of BODIPY **5e** in THF.

Next, UV–vis absorption and fluorescence spectra were measured for all BODIPY **5** in THF (Figure 2, Table 2). These spectra were found to be very similar for all BODIPYs **5** (See Supplementary Materials, Tables S1–S9, Figures S2–S10). For example, the absorption maximum for the main intense band varies in a narrow range of 531–545 nm. The emission maximum of BODIPYs **5a**–**h** varies in the range of 577–597 nm. Their Stokes shifts change from 42 nm to 52 nm. The variation of the structure of an amide group did not affect the absorption and emission maxima (Table 2, entries 1–5). In turn, the change of an aryl substituent at the *meso*-position has a noticeable effect only in the case of 2,4-dichlorophenyl group (**5h**). A red shift was observed for both absorption (12 nm) and emission maxima (13 nm) compared to *meso*-phenyl-substituted BODIPY **5f** (Table 2, Cf. entries 6 and 8).

The quantum yield of fluorescence for **5f** is expectedly low (Φ*_F_* = 0.01) due to the free rotation of the phenyl group (Table 2, entry 6). However, the replacement of phenyl with rotationally restricted 2-methylphenyl group led to a significant increase of Φ*_F_* from 0.01 to 0.16 (Table 2, Cf. entries 6 and 7). The change of the 2-methylphenyl group with 2,4,5-tri methylphenyl group gave somewhat lower Φ_Δ_ for BODIPY **5e** (Table 2, entry 5). BODIPYs **5a**–**5d** with primary amide groups demonstrated similar values of Φ*_F_* (0.09–0.17) compared to those of morpholine-substituted dyes **5e**, **5g** (0.12–0.16).

The usage of other rotationally restricted *meso*-aryls was found ineffective. For example, BODIPY **5h** having two chlorines at 2,4-positions of the phenyl ring showed Φ*_F_* of 0.03 (Table 2, entry 8). In turn, the replacement of methyl groups with strong electron-donating methoxy ones completely quenches fluorescents in dyes **5i**–**5j** (Table 2, Cf. entries 5, 7 with 9, 10).

It is also known that *meso*-aryl moiety can be used as a donor subunit, while a BODIPY core is an acceptor subunit, for the creation of donor-acceptor dyad. The dihedral angle between donor and acceptor subunits close to 90 °C is favourable for the realization of a spin-orbit charge transfer intersystem crossing (SOCT-ISC) [52]. Therefore, the usage of rotationally restricted *meso*-aryl is also favourable for singlet oxygen generation.

The ability of BODIPY **5** to generate singlet oxygen was measured in acetonitrile (Table 2). We found that photosensitizing and fluorescence properties for studied BODIPY demonstrated a very similar trend. Indeed, BODIPYs **5i**–**5j** with highly electron-rich methoxy-substituted phenyl rings did not show any singlet oxygen generation (Table 2, entries 9–10). In turn, *meso*-phenyl substituted BODIPY **5f** showed a very low singlet oxygen quantum yield (Φ_Δ_) of <0.05. For the other dyes, Φ_Δ_ was found in a range of 0.09–0.16.

### 2.3. Comparison of Structurally Related Core-Fluorinated BODIPYs

Next, the photophysical properties of obtained BODIPY-diamides were compared with structurally related core-fluorinated BODIPYs [35,36,37] to reveal the substituent effect at the 3,5-positions (Table 3). As one can see, the replacement of amide groups with methyl ones leads to a blue shift of both absorption and emission maxima (See Supplementary Materials, Table S11, Figure S12). In turn, the change of amide groups with ethoxycarbonyl ones results in a slight hypsochromic shift of λ_abs (max)_ and bathofloral shift of λ_em (max)_ (See Supplementary Materials, Table S10, Figure S11). Methyl groups provide a narrower Stokes shift (35 nm), whereas ethoxycarbonyl groups provide a broader shift (56 nm) than amide groups at the 3,5-positions (44–46 nm). However, the substituents at 3,5-position have the most significant effect on fluorescent properties. While the diester derivative **5k** exhibits virtually no fluorescent properties, the diamide derivatives **5a** and **5e** exhibit moderate fluorescent properties, and the dimethyl derivative **5l** exhibits excellent fluorescent properties. The electronic effect of 3,5-substituents on singlet oxygen generation is negligible. Indeed, BODIPY with both electron-donating (EDG) **5l** and electron-withdrawing groups (EWG) **5e** and **5k** demonstrated similar values of Φ_Δ_.

**Figure 3:**
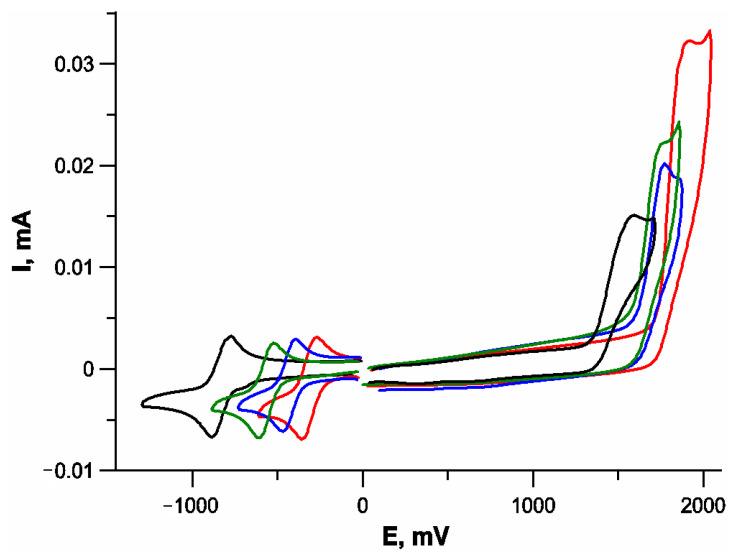
Cyclic voltammograms of BODIPYs **5a**–**5k** at 100 mVs^−1^ in 0.1 M of Bu_4_NClO_4_ in CH_3_CN using GC as a stationary electrode. Peaks of the first redox processes: **5a**—**blue**; **5e**—**green**; **5l**—**black**; **5k**—**red**.

Next, the cyclic voltammetry (CV) and rotating disk electrode (RDE) methods were performed to study the influence of 3,5-substituents of BODIPY **5a**, **5e**, **5k** and **5l** on their electrochemical properties. Measurements were performed on a cleaned surface of a glassy carbon (GC) electrode in acetonitrile solutions. Voltamperograms were recorded from 0 to the cathodic or anodic potential region (Figure 3). The values of half-peak potential are summarized in Table 4. The studied BODIPYs were reduced in two one-electron stages, the first of which was reversible. Oxidation is irreversible and more than one electron is accompanied by adsorption, as evidenced by the observed current drop in the RDE experiments (See Supplementary Materials, Figure S13).

The change in cathodic potentials turned out to be more significant (change range of 500 mV) compared to anodic potentials (change range of 340 mV) depending on the nature of 3,5-substituents (Figure 3, Table 4). It was found that the value of the potentials depends on the electronic effect of the substituents. As expected, both the cathodic and anodic potentials gradually decrease with the diminution of the electron-withdrawing capacity of 3,5-substituents in a row: **5k** > **5a** > **5e** > **5l**.

**Figure 4:**
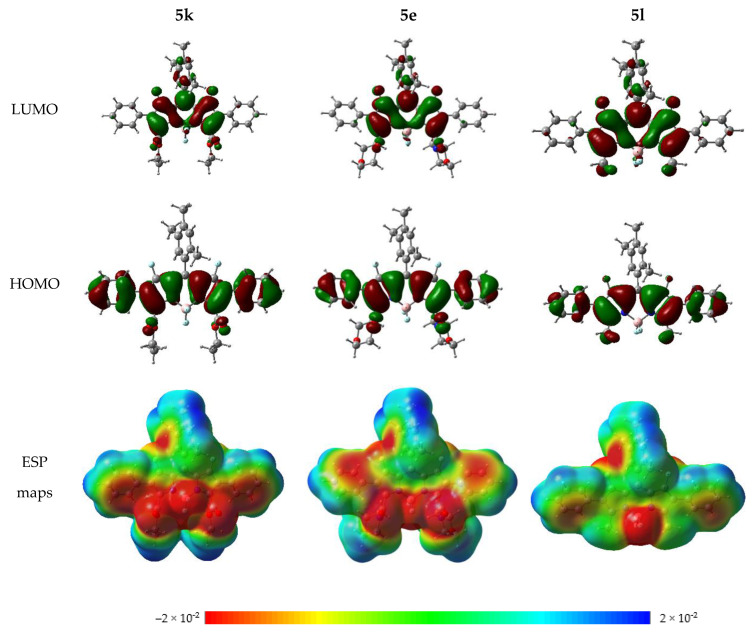
The optimized geometry of the ground electronic state of compounds, the frontier orbitals localization and EPMs of **5e**, **5k** and **5l**.

Finally, DFT calculations were performed to compare the electronic structure of BODIPYs **5a**, **5e**, **5k** and **5l** [53]. The optimized geometry of the ground electronic state of compounds **5e**, **5k** and **5l**, the frontier orbitals localization and electrostatic potential maps (EPM) are depicted in Figure 4.

Frontier orbital localization is very similar for all structures. Both the highest occupied molecular orbital (HOMO) and the lowest unoccupied molecular orbital (LUMO) are localized on the BODIPY core and on the 3,5-substituents. LUMO to some extent localized on a phenyl ring at the *meso*-position, whereas HOMO is not localized (Figure 4). LUMO is localized on the 1,7-fluorines, but HOMO is not localized, except for **5l** where slight localization of HOMO is observed.

EPMs of BODIPYs **5e**, **5k** and **5l** visualize charge distributions for these molecules (Figure 4). The nature of the substituents at the 3,5 positions makes a significant contribution to the charge distribution. For dyes **5k**, **5e** the most electronegative sites (coloured in red) are mainly located around the -BF_2_, pyrrole nitrogens, electron-withdrawing carbonyl groups and phenyls at the 2,6-positions. The replacement of EWG groups with electron-donating methyl ones (**5l**) significantly reduces electronegative charge around the 1,7-positions and on neighbouring phenyl rings. On the other hand, less negative sites (coloured in blue) are mainly located around the phenyl at the *meso*-position. This charge distribution is favourable for the implementation of SOCT-ISC, which is confirmed by experimental data on the generation of singlet oxygen.

**Figure 5:**
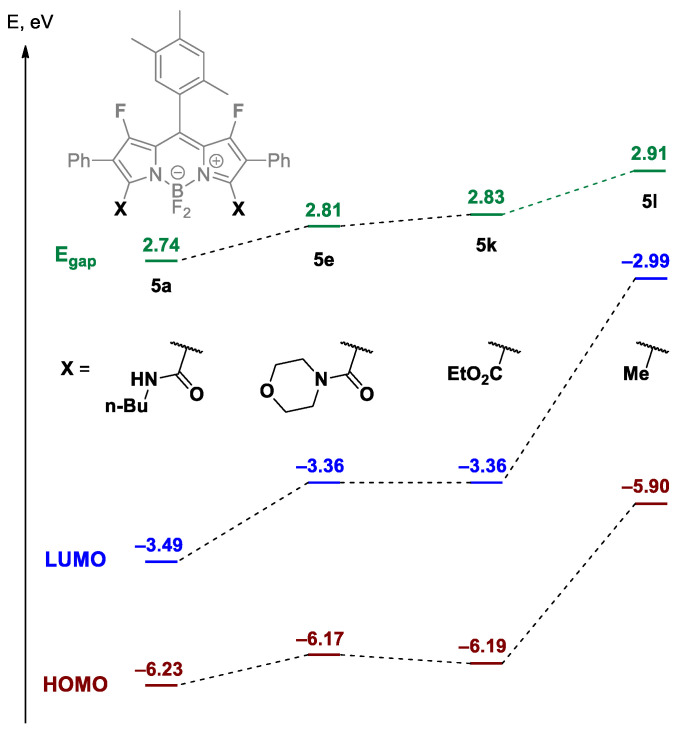
Frontier orbitals calculated for optimized geometry of selected BODIPYs.

In addition, the energies of HOMO, LUMO and the energy gap between them (E_gap_) were calculated (Figure 5).

Electron-withdrawing morpholine-amide and ethoxycarbonyl groups have a similar impact on the level of the frontier orbitals. In turn, n-butylamide groups lead to a decrease in both frontier orbitals. In contrast, electron-donating methyl groups increase the energy of both HOMO and LUMO compared to EWG-containing BODIPYs **5a**, **5e**, and **5k**. In both cases, the change of LUMO is more significant than HOMO, resulting in compression of E_gap_ for **5a** and extension of E_gap_ for **5l**.

## 3. Materials and Methods

### 3.1. Materials

Synthesis of the starting 2-isocyanoacetamides was carried out according to the described procedures and all of them are known compounds [54,55]. Diethyl 5,5′-((2,4,5-trimethylphenyl)methylene)bis(4-fluoro-3-phenyl-1*H*-pyrrole-2-carboxylate) and BODIPY **5l** were previously obtained and characterized [37]. All other reagents were purchased from commercial sources and used as received (Sigma-Aldrich, St. Louis, MO, USA; Macklin Inc., Shanghai, China). The solvents were dried before usage according to standard procedures [56]. Melting points (mp) were measured using a Büchi B-545 melting point apparatus (BÜCHI Labortechnik AG, Flawil, Switzerland). Microwave-assisted reactions were carried out in a Nova-2S microwave reactor (PreeKem Scientific Instruments Co.,Ltd., Shanghai, China) in sealed vessels at a constant temperature. Gradient column chromatography was performed using SepaBean™ machine T (Santai Science Inc., Montréal, QC, Canada).

^1^H, ^13^C{^1^H} and ^19^F NMR spectra were obtained with Bruker Avance-400 (Bruker Corporation, Karlsruhe, Germany). Chemical shifts for ^1^H NMR and ^13^C NMR spectroscopic data were referenced to the residual solvent resonance. Chemical shifts for ^19^F NMR spectroscopic data were referenced to PhCF_3_ (δ = − 63.72 ppm) added to analyzed samples as an internal standard. Spectroscopic data are presented in the following order: chemical shift, multiplicity, spin-spin coupling constant (Hz), and integrated intensity, where br = broad, s = singlet, d = doublet, t = triplet, q = quartet, m = multiplet, dd = doublet of doublets, ddd = doublet of doublet of doublets, dt = doublet of triplets, tt = triplet of triplets, ddt = doublet of doublet of triplets. The reactions were monitored by ^19^F NMR spectroscopy using a Magritek Spinsolve 60 MHz spectrometer (Magritek GmbH, Aachen, Germany).

Electronic absorption spectra were recorded on Agilent Cary 60 UV–vis (Agilent Technologies, Inc., Santa Clara, CA, USA) and JASCO V-770 UV-vis-NIR spectrophotometers (JASCO, Easton, MD, USA) in quartz cuvettes with an optical path length of 10 mm. Concentrations of the analyzed solutions were ~10^−4^ M. Emission spectra were recorded on a Hitachi F-2700 fluorescence spectrophotometer (Hitachi Ltd., Tokyo, Japan) with xenon lamp as excitation source in a 10 mm quartz cuvette. Concentrations of the analyzed solutions were ~10^−6^ M.

### 3.2. Fluorescence Quantum Yields

Fluorescence quantum yields (Φ*_F_*) were determined by the comparative method. The solution of Rhodamine 6G in EtOH (Φ*_F_* = 0.94) was used as a standard [57]. Both the samples and standard were excited at the same wavelength (490 nm). The absorbance of the solutions at the excitation wavelength is always below 0.1. Experiments were carried out at room temperature.

Equation (1) was employed for the calculations:

(1) $$\Phi_{F}=\Phi_{F\;(st)}\frac{S\cdot A_{st}\cdot n^{2}}{S_{st}\cdot A\cdot n_{st}^{2}}\;$$

where *S* and *S_st_* are the areas under the fluorescence emission curves of the samples and the standard, respectively; *A* and *A_st_* are the respective absorbances of the samples and standard at the excitation wavelengths, respectively; *n* and *n_st_* are refractive indexes of solvents in which the samples and standard are dissolved, respectively.

### 3.3. Singlet Oxygen Generation Measurements

Singlet oxygen generation measurements were performed using halogen-deuterium lamp (DH-2000, Ocean Optics, Inc., Orlando, FL, USA), monochromator MS 2004i (SOL Instruments, Minsk, Belarus), and fibre optics spectrophotometer AvaSpec-ULS2048CL-EVORS-UA (Avantes B.V., Apeldoorn, The Netherlands) and a thermostatically controlled cell holder CUV-UV/VIS-TCABS/FL (Ocean Optics, Inc., Orlando, FL, USA) with stirring. The light intensity was the same at all excitation wavelengths and was 2.43 × 10^16^ photon × s^−1^ × cm^−2^.

Singlet oxygen quantum yields (Φ_∆_) were determined in air in acetonitrile solutions using the relative method with Methylene Blue (in acetonitrile) as a reference. A singlet oxygen trap DPBF in acetonitrile was added to BODIPY solutions in acetonitrile (2.5 mL) in 10 mm quartz cuvettes equipped with caps, and its concentration did not exceed 2.5 × 10^−5^ M to prevent chain reactions [58]. The resulting solutions were irradiated at 555 nm and DPBF degradation at 410 nm was monitored. The constant temperature (25 °C) and continuous stirring (1200 rpm) were provided during the irradiation. Singlet oxygen quantum yields (Φ_Δ_) were calculated according to the following equation:

(2) $$\Phi_{\Delta }=\Phi_{\Delta (st)}\times \frac{R}{R_{st}}\times \frac{1-10^{-A_{st}}}{1-10^{-A}}\;$$

where Φ_Δ(st)_ is the quantum yields of singlet oxygen generation for the standard—Methylene blue (Φ_Δ(st)_ = 0.52) [59]; *R* is rate of photobleaching of DPBF under irradiation; *A* is optical density of BODIPY at the irradiation wavelength.

### 3.4. Electrochemical Measurements

Electrochemical measurements were performed using an IPC_Pro M potentiostat (Volta, Saint Petersburg, Russia) in a three-electrode system. A glassy carbon and gold disks (d = 2 mm) were used as the working electrode. Ag/AgCl/KCl (aq., sat.) was used as a reference electrode. In turn, a platinum plate was used as an auxiliary electrode. A solution of Bu_4_NClO_4_ (0.1 M) in MeCN was used as the supporting electrolyte. The working electrode surface was polished by alumina powder with a particle size of less than 0.5 µm (Sigma-Aldrich, St. Louis, MO, USA). In the CV measurements, the potential sweep rate was 100 mV·s^–1^. In studies with the rotating disk electrode (RDE) method, the potential sweep rate is 20 mV·s^–1^. The potentials are presented with *iR*-compensation. The number of transferred electrons in redox processes was determined by comparing the peak current in the substrate and the current of single-electron oxidation of ferrocene taken in the same concentration. All measurements were carried out in a dry argon atmosphere. Samples were dissolved in a dry, pre-deaerated solvent at 22 °C. The concentration of solutions of BODIPYs **5** was ~10^−3^ M.

### 3.5. Synthesis and Characterization

**General Procedure for the Barton−Zard Reaction of *β*-Fluoro-*β*-nitrostyrenes with 2-isocyanoacetamide**.

A solution of a selected 2-isocyanoacetamide (5.6 mmol, 2 mol equiv) and DBU (0.837 mL, 5.6 mmol, 2 mol equiv) in DCM (28 mL) was prepared and loaded into a round-bottom flask equipped with a dropping funnel. Then, a solution of (*Z*)-(2-fluoro-2-nitrovinyl)benzene (0.468 g, 2.8 mmol, 1 mol equiv) in DCM (57 mL) was added dropwise to the vigorously stirred reaction mixture at room temperature over 2 h. After completion of the reaction (TLC monitoring), a 5% aqueous solution of HCl (100 mL) was added to the reaction mixture to hydrolyze the residual 2-isocyanoacetamide. The resulting mixture was vigorously stirred for 2 h. Next, the organic layer was separated and the water layer was extracted with DCM (2 × 50 mL). The combined organic phase was dried over anhydrous Na_2_SO_4_, filtered and concentrated under a vacuum. The residue was separated by column chromatography on silica gel using gradient elution to obtain the desired pyrrole **2** and pyrrole **3** as a side-product.

*N-butyl-4-fluoro-3-phenyl-1H-pyrrole-2-carboxamide* (**2a**). Eluent: Hex/EtOAc (9:1), Hex/EtOAc (6:1), Hex/EtOAc (3:1). Yield: 0.357 g (50%). Orange solid; mp 110–112 °C. ^1^H NMR (400 MHz, CDCl_3_): δ 10.62 (br s, 1H), 7.50–7.38 (m, 5H), 6.75 (t, *J* = 3.5 Hz, 1H), 5.70 (t, *J* = 4.9 Hz, 1H), 3.25 (q, *J* = 6.8 Hz, 2H), 1.31 (dt, *J* = 14.8, 7.0 Hz, 2H), 1.21–1.08 (m, 2H), 0.83 (t, *J* = 7.3 Hz, 3H). ^13^C{^1^H} NMR (100 MHz, CDCl_3_): δ 161.3 (d, ^4^*J*_CF_ = 2.5 Hz), 149.6 (d, ^1^*J*_CF_ = 242.9 Hz), 131.2 (d, ^3^*J*_CF_ = 2.0 Hz), 130.5, 129.2, 128.3, 118.7 (d, ^3^*J*_CF_ = 2.3 Hz), 113.0 (d, ^2^*J*_CF_ = 12.9 Hz), 105.1 (d, ^2^*J*_CF_ = 27.0 Hz), 39.0, 31.2, 19.9, 13.7. ^19^F NMR (376 MHz, CDCl_3_): δ −169.02 (s, 1F). HRMS (ESI) *m*/*z*: [M + H]^+^ calcd. for C_15_H_18_FN_2_O^+^ 261.1398; found 261.1400.

*N-butyl-4-nitro-3-phenyl-1H-pyrrole-2-carboxamide* (**3a**). Eluent: Hex/EtOAc (9:1), Hex/EtOAc (6:1), Hex/EtOAc (3:1). Yellow solid; mp 113–115 °C. Yield: 0.101 g (13%). ^1^H NMR (400 MHz, CDCl_3_): δ 11.28 (br s, 1H), 7.81 (s, 1H), 7.60–7.46 (m, 3H), 7.45–7.34 (m, 2H), 5.43 (br s, 1H), 3.17 (q, *J* = 6.2 Hz, 2H), 1.26–1.13 (m, 2H), 1.09–0.96 (m, 2H), 0.79 (t, *J* = 7.2 Hz, 3H). ^13^C{^1^H} NMR (100 MHz, CDCl_3_): δ 160.3, 135.9, 131.4, 130.1, 129.4, 129.2, 123.7, 122.0, 120.9, 39.2, 30.9, 19.8, 13.6. HRMS (ESI) *m*/*z*: [M + H]^+^ calcd. for C_15_H_18_N_3_O_3_^+^ 288.1343; found 288.1343.

*N-cyclopropyl-4-fluoro-3-phenyl-1H-pyrrole-2-carboxamide* (**2b**). Eluent: Hex/EtOAc (4:1). Yield: 0.170 g (25%). Sandy solid; mp 182–184 °C. ^1^H NMR (400 MHz, DMSO-d6): δ 11.42 (s, 1H), 7.43–7.36 (m, 4H), 7.35–7.27 (m, 1H), 7.16–7.05 (m, 1H), 6.90 (t, *J* = 3.4 Hz, 1H), 2.67 (dt, *J* = 10.4, 3.5 Hz, 1H), 0.65–0.56 (m, 2H), 0.37–0.30 (m, 2H). ^13^C{^1^H} NMR (100 MHz, DMSO-d6): δ 161.9, 148.8 (d, ^1^*J*_CF_ = 239.7 Hz), 131.3 (d, ^3^*J*_CF_ = 2.5 Hz), 129.7, 128.2, 127.0, 119.7 (d, ^3^*J*_CF_ = 3.2 Hz), 112.5 (d, ^2^*J*_CF_ = 11.7 Hz), 104.3 (d, ^2^*J*_CF_ = 26.9 Hz), 22.4, 5.9. ^19^F NMR (376 MHz, DMSO-d6): δ −171.74 (s, 1F). HRMS (ESI) *m*/*z*: [M + H]^+^ calcd. for C_14_H_14_FN_2_O^+^ 245.1085; found 245.1086.

*N-allyl-4-fluoro-3-phenyl-1H-pyrrole-2-carboxamide* (**2c**). The pure product was isolated by column chromatography with gradient elution on silica gel using SepaBean™ machine T. Polarity of the elution system was gradually increased from Hex/EtOAc (9:1) to Hex/EtOAc (1:1). Yield: 0.260 g (37%). Dark yellow solid; mp 104–106 °C. ^1^H NMR (400 MHz, CDCl_3_): δ 10.96 (s, 1H), 7.54–7.36 (m, 5H), 6.79 (t, *J* = 3.6 Hz, 1H), 5.88 (t, *J* = 5.6 Hz, 1H), 5.73 (ddt, *J* = 22.4, 10.5, 5.3 Hz, 1H), 5.03 (dd, *J* = 10.4, 1.2 Hz, 1H), 4.96 (dd, *J* = 17.2, 1.3 Hz, 1H), 3.96–3.84 (m, 2H). ^13^C{^1^H} NMR (100 MHz, CDCl_3_): δ 161.3 (d, ^4^*J*_CF_ = 2.4 Hz), 149.4 (d, ^1^*J*_CF_ = 242.8 Hz), 133.5, 131.0 (d, ^3^*J*_CF_ = 1.8 Hz), 130.3, 129.1, 128.2, 118.2 (d, ^3^*J*_CF_ = 2.2 Hz), 115.8, 113.3 (d, ^2^*J*_CF_ = 12.8 Hz), 105.6 (d, ^2^*J*_CF_ = 26.9 Hz), 41.5. ^19^F NMR (376 MHz, CDCl_3_): δ −169.18 (s, 1F). HRMS (ESI) *m*/*z*: [M + H]^+^ calcd. for C_14_H_14_FN_2_O^+^ 245.1085; found 245.1086.

*N-allyl-4-nitro-3-phenyl-1H-pyrrole-2-carboxamide* (**3c**). The pure product was isolated by column chromatography with gradient elution on silica gel using SepaBean™ machine T. Polarity of the elution system was gradually increased from Hex/EtOAc (9:1) to Hex/EtOAc (1:1). Yield: 0.074 g (9%). Yellow solid; mp 158–160 °C. ^1^H NMR (400 MHz, CDCl_3_): δ 11.70 (br s, 1H), 7.83 (s, 1H), 7.58–7.46 (m, 3H), 7.45–7.36 (m, 2H) 5.71–5.49 (m, 2H), 5.00 (d, *J* = 10.2 Hz, 1H), 4.82 (d, *J* = 17.1 Hz, 1H), 3.81 (s, 2H). ^13^C{^1^H} NMR (100 MHz, CDCl_3_): δ 160.4, 135.8, 132.8, 131.2, 130.0, 129.4, 129.3, 123.4, 122.4, 121.3, 116.2, 41.7. HRMS (ESI) *m*/*z*: [M + H]^+^ calcd. for C_14_H_14_N_3_O_3_^+^ 272.1030; found 272.1030.

**Procedure for the Scaled-Up Preparation of 2d**.

A solution of *N*-benzyl-2-isocyanoacetamide (4.607 g, 26.4 mmol, 2 mol equiv) and DBU (3.955 mL, 26.4 mmol, 2 mol equiv) in DCM (134 mL) was prepared and loaded into a round-bottom flask equipped with a dropping funnel. Then, a solution of (*Z*)-(2-fluoro-2-nitrovinyl)benzene (2.210 g, 13.2 mmol, 1 mol equiv) in DCM (267 mL) was added dropwise to the vigorously stirred reaction mixture at room temperature over 6 h. After completion of the reaction (TLC monitoring), 5% aqueous solution of HCl (400 mL) was added to the reaction mixture to hydrolyze the residual *N*-benzyl-2-isocyanoacetamide. The resulting mixture was vigorously stirred for 2 h. Next, the organic layer was separated and the water layer was extracted with DCM (2 × 200 mL). The combined organic phase was dried over anhydrous Na_2_SO_4_, filtered and concentrated under a vacuum. The residue was separated by column chromatography on silica gel using SepaBean™ machine T to obtain the desired pyrrole **2d** and pyrrole **3d** as a side-product. The polarity of the elution system was gradually increased from Hex/EtOAc (9:1) to Hex/EtOAc (1:1), and then EtOAc was used.

*N-benzyl-4-fluoro-3-phenyl-1H-pyrrole-2-carboxamide* (**2d**). Yield: 0.658 g (17%). Viscous oil. ^1^H NMR (400 MHz, CDCl_3_): δ 10.10 (s, 1H), 7.46–7.22 (m, 8H), 7.10 (m, 2H), 6.71 (t, *J* = 3.4 Hz, 1H), 6.05 (t, *J* = 5.6 Hz, 1H), 4.45 (d, *J* = 5.8 Hz, 2H). ^13^C{^1^H} NMR (100 MHz, CDCl_3_): δ 161.1 (d, ^4^*J*_CF_ = 2.6 Hz), 149.7 (d, ^1^*J*_CF_ = 243.5 Hz), 137.8, 130.9 (d, ^3^*J*_CF_ = 1.7 Hz), 130.4, 129.2, 128.7, 128.4, 127.5, 127.5, 118.5 (d, ^3^*J*_CF_ = 2.1 Hz), 113.4 (d, ^2^*J*_CF_ = 12.9 Hz), 105.3 (d, ^2^*J*_CF_ = 27.1 Hz), 43.5. ^19^F NMR (376 MHz, CDCl_3_): δ −168.68 (*pseudo*-t, ^3^*J*_HF_ = ^4^*J*_HF_ = 3.0 Hz, 1F). HRMS (ESI) *m*/*z*: [M + H]^+^ calcd. for C_18_H_16_FN_2_O^+^ 295.1241; found 295.1243.

*N-benzyl-4-nitro-3-phenyl-1H-pyrrole-2-carboxamide* (**3d**). Yield: 0.448 g (11%). Orange viscous oil. ^1^H NMR (400 MHz, CDCl_3_) δ 11.72 (s, 1H), 7.69 (d, *J* = 3.8 Hz, 1H), 7.47–7.24 (m, 8H), 7.00–6.93 (m, 2H), 5.84 (t, *J* = 5.4 Hz, 1H), 4.34 (d, *J* = 5.6 Hz, 2H). ^13^C{^1^H} NMR (100 MHz, CDCl_3_) δ 160.0, 136.4, 135.3, 130.6, 129.5, 128.8, 128.7, 128.3, 127.3, 126.7, 122.8, 122.0, 120.9, 43.3. HRMS (ESI) *m*/*z*: [M + H]^+^ calcd. for C_18_H_16_N_3_O_3_^+^ 322.1186; found 322.1188.

**Procedure for the Scaled-Up Preparation of 2e**.

A solution of 2-isocyano-1-morpholinoethanone (5.486 g, 35.6 mmol, 2 mol equiv) and DBU (5.322 mL, 35.6 mmol, 2 mol equiv) in DCM (180 mL) was prepared and loaded into a round-bottom flask equipped with a dropping funnel. Then, a solution of (*Z*)-(2-fluoro-2-nitrovinyl)benzene (2.974 g, 17.8 mmol, 1 mol equiv) in DCM (359 mL) was added dropwise to the vigorously stirred reaction mixture at room temperature over 8 h. After completion of the reaction (TLC monitoring), 5% aqueous solution of HCl (500 mL) was added to the reaction mixture to hydrolyze the residual 2-isocyano-1-morpholinoethanone. The resulting mixture was vigorously stirred for 2 h. Next, the organic layer was separated and the water layer was extracted with DCM (2 × 250 mL). The combined organic phase was dried over anhydrous Na_2_SO_4_, filtered and concentrated under a vacuum. The residue was separated by column chromatography on silica gel using SepaBean™ machine T to obtain the desired pyrrole **2d** and pyrrole **3d** as a side-product. The polarity of the elution system was gradually increased from Hex/EtOAc (2:1) to Hex/EtOAc (1:4).

*(4-Fluoro-3-phenyl-1H-pyrrol-2-yl)(morpholino)methanone* (**2e**). Yield: 2.604 g (53%). Yellow solid; mp 209–211 °C. ^1^H NMR (400 MHz, CDCl_3_): δ 10.02 (s, 1H), 7.46–7.30 (m, 5H), 6.73 (t, *J* = 3.1 Hz, 1H), 3.33 (br s, 8H). ^13^C{^1^H} NMR (100 MHz, CDCl_3_): δ 164.0 (d, ^4^*J*_CF_ = 2.0 Hz), 149.0 (d, ^1^*J*_CF_ = 243.5 Hz), 131.7 (d, ^3^*J*_CF_ = 2.4 Hz), 129.3, 129.0, 127.6, 118.0 (d, ^3^*J*_CF_ = 2.9 Hz), 113.7 (d, ^2^*J*_CF_ = 12.1 Hz), 105.0 (d, ^2^*J*_CF_ = 27.5 Hz), 66.1, 66.1. ^19^F NMR (376 MHz, CDCl_3_): δ −170.39 (*pseudo*-t, ^3^*J*_HF_ = ^4^*J*_HF_ = 3.1 Hz, 1F). HRMS (ESI) *m*/*z*: [M + H]^+^ calcd. for C_15_H_16_FN_2_O_2_^+^ 275.1190; found 275.1190.

*Morpholino(4-nitro-3-phenyl-1H-pyrrol-2-yl)methanone* (**3e**). Yield: 0.980 g (18%). Dark yellow solid; mp 213–215 °C. ^1^H NMR (400 MHz, CDCl_3_): δ 11.61 (s, 1H), 7.84 (d, *J* = 3.6 Hz, 1H), 7.49–7.35 (m, 5H), 3.76–2.55 (m, 8H). ^13^C{^1^H} NMR (100 MHz, CDCl_3_): δ 162.9, 134.6, 131.1, 130.6, 128.8, 128.7, 123.2, 123.0, 121.3, 65.8, 65.7. HRMS (ESI) *m*/*z*: [M + H]^+^ calcd. for C_15_H_16_N_3_O_4_^+^ 302.1135; found 302.1136.

**General Procedure for the Preparation of Dipyrromethanes 4.**

In a typical experiment, a selected pyrrole **2** (0.4–1.4 mmol, 1 mol equiv) and a corresponding aldehyde (0.4–1.4 mmol, 1 mol equiv) were loaded into a suitable vial and dissolved in DCM (2–7 mL). In the case of obtaining dipyrromethane **4b**, EtOAc was used instead of DCM. Then triflic acid (0.4–1.4 mmol, 1 mol equiv) was added to the reaction mixture and the resulting mixture was stirred for 24 h. Progress of the reaction was monitored by TLC or ^19^F NMR spectroscopy. After completion of the reaction, the reaction mixture was diluted with EtOAc (50–100 mL) and extracted with water (3 × (50–100) mL). Then, the organic layer was separated, dried over Na_2_SO_4_, filtered and concentrated under a vacuum. The pure product was isolated by column chromatography on silica gel using appropriate elution mixtures.

*5,5′-((2,4,5-Trimethylphenyl)methylene)bis(N-butyl-4-fluoro-3-phenyl-1H-pyrrole-2-carboxamide)* (**4a**). The pure product was isolated by column chromatography with gradient elution on silica gel using SepaBean™ machine T. Polarity of the elution system was gradually increased from DCM/EtOAc (99:1) to DCM/EtOAc (4:1). Yield: 0.135 g (83%). Orange solid; mp 208–210 °C. ^1^H NMR (400 MHz, CDCl_3_): δ 10.14 (br s, 2H), 7.46–7.33 (m, 10H), 7.14 (s, 1H), 6.94 (s, 1H), 5.87 (s, 1H), 5.64 (t, *J* = 5.4 Hz, 2H), 3.22–3.08 (m, 4H), 2.30 (s, 3H), 2.19 (s, 3H), 2.17 (s, 3H), 1.28–1.15 (m, 4H), 1.14–1.01 (m, 4H), 0.77 (t, *J* = 7.3 Hz, 6H). ^13^C{^1^H} NMR (100 MHz, CDCl_3_): δ 160.8 (d, ^4^*J*_CF_ = 2.5 Hz), 146.3 (d, ^1^*J*_CF_ = 244.4 Hz), 135.8, 134.7, 134.2, 133.1, 132.3, 131.2 (d, ^3^*J*_CF_ = 1.9 Hz), 130.5, 129.5, 129.1, 128.2, 117.3 (d, ^2^*J*_CF_ = 21.4 Hz), 117.1 (d, ^3^*J*_CF_ = 2.2 Hz), 113.3 (d, ^2^*J*_CF_ = 12.4 Hz), 39.0, 34.3, 31.1, 20.0, 19.7, 19.4, 19.0, 13.7. ^19^F NMR (376 MHz, CDCl_3_): δ −168.15 (s, 2F). HRMS (ESI) *m*/*z*: [M + H]^+^ calcd. for C_40_H_45_F_2_N_4_O_2_^+^ 651.3505; found 651.3505.

*5,5′-((2,4,5-Trimethylphenyl)methylene)bis(N-cyclopropyl-4-fluoro-3-phenyl-1H-pyrrole-2-carboxamide)* (**4b**). The pure product was isolated by column chromatography with gradient elution on silica gel using SepaBean™ machine T. Polarity of the elution system was gradually increased from DCM/EtOAc (98:2) to DCM/EtOAc (2.3:1). Yield: 0.087 g (78%). Orange solid; mp 160–162 °C. ^1^H NMR (400 MHz, CDCl_3_): δ 10.74 (br s, 2H), 7.36 (s, 10H), 7.25 (s, 1H), 6.94 (s, 1H), 5.94 (s, 1H), 5.73 (d, *J* = 3.3 Hz, 2H), 2.70 (dt, *J* = 10.6, 3.5 Hz, 2H), 2.32 (s, 3H), 2.18 (s, 3H), 2.14 (s, 3H), 0.57 (q, *J* = 6.2 Hz, 4H), 0.16 (q, *J* = 6.6 Hz, 4H). ^13^C{^1^H} NMR (100 MHz, CDCl_3_): δ 162.1, 146.2 (d, ^1^*J*_CF_ = 244.2 Hz), 135.6, 134.5, 134.4, 133.2, 132.1, 131.1 (d, ^3^*J*_CF_ = 1.5 Hz), 130.4, 129.8, 128.9, 128.2, 118.0 (d, ^2^*J*_CF_ = 20.8 Hz), 116.9 (d, ^3^*J*_CF_ = 2.4 Hz), 113.5 (d, ^2^*J*_CF_ = 12.0 Hz), 34.3, 22.4, 19.6, 19.4, 19.1, 6.5, 6.5. ^19^F NMR (376 MHz, CDCl_3_): δ −167.94 (s, 2F). HRMS (ESI) *m*/*z*: [M + H]^+^ calcd. for C_38_H_37_F_2_N_4_O_2_^+^ 619.2879; found 619.2881.

*5,5′-((2,4,5-Trimethylphenyl)methylene)bis(N-allyl-4-fluoro-3-phenyl-1H-pyrrole-2-carboxamide)* (**4c**). The pure product was isolated by column chromatography with gradient elution on silica gel using SepaBean™ machine T. Polarity of the elution system was gradually increased from DCM/EtOAc (98:2) to DCM/EtOAc (3:1). Yield: 0.103 g (68%). Sandy solid; mp 237–239 °C. ^1^H NMR (400 MHz, CDCl_3_): δ 11.07 (br s, 2H), 7.43–7.30 (m, 11H), 6.95 (s, 1H), 5.94 (s, 1H), 5.78 (t, *J* = 5.3 Hz, 2H), 5.61 (ddt, *J* = 22.6, 10.7, 5.5 Hz, 2H), 4.96 (d, *J* = 10.3 Hz, 2H), 4.85 (d, *J* = 17.2 Hz, 2H), 3.86–3.69 (m, 4H), 2.37 (s, 3H), 2.20 (s, 3H), 2.18 (s, 3H). ^13^C{^1^H} NMR (100 MHz, CDCl_3_): δ 160.7 (d, ^4^*J*_CF_ = 2.6 Hz), 146.4 (d, ^1^*J*_CF_ = 244.0 Hz), 135.5, 134.6, 134.5, 133.5, 133.0, 132.0, 131.2 (d, ^3^*J*_CF_ = 1.8 Hz), 130.4, 129.9, 129.1, 128.1, 118.3 (d, ^2^*J*_CF_ = 20.2 Hz), 116.5 (d, ^3^*J*_CF_ = 2.5 Hz), 116.2, 113.5 (d, ^2^*J*_CF_ = 12.0 Hz), 41.8, 34.4, 19.6, 19.4, 19.1. ^19^F NMR (376 MHz, CDCl_3_): δ −168.08 (s, 2F). HRMS (ESI) *m*/*z*: [M + H]^+^ calcd. for C_38_H_37_F_2_N_4_O_2_^+^ 619.2879; found 619.2879.

*5,5′-((2,4,5-Trimethylphenyl)methylene)bis(N-benzyl-4-fluoro-3-phenyl-1H-pyrrole-2-carboxamide)* (**4d**). The pure product was isolated by column chromatography with gradient elution on silica gel using SepaBean™ machine T. Polarity of the elution system was gradually increased from DCM/EtOAc (99:1) to DCM/EtOAc (9:1). Yield: 0.179 g (56%). Sandy solid; mp 126–128 °C. ^1^H NMR (400 MHz, CDCl_3_): δ 11.46 (br s, 2H), 7.56 (s, 1H), 7.33–7.23 (m, 12H), 7.19 (t, *J* = 7.4 Hz, 4H), 7.02 (s, 1H), 7.02–6.96 (m, 4H), 6.10 (s, 1H), 6.06 (t, *J* = 5.2 Hz, 2H), 4.48–4.31 (m, 4H), 2.47 (s, 3H), 2.24 (s, 3H), 2.22 (s, 3H). ^13^C{^1^H} NMR (100 MHz, CDCl_3_): δ 160.8 (d, ^4^*J*_CF_ = 2.4 Hz), 146.4 (d, ^1^*J*_CF_ = 244.3 Hz), 137.2, 135.4, 134.8, 134.4, 133.1, 131.9, 130.9 (d, ^3^*J*_CF_ = 1.9 Hz), 130.3, 130.1, 128.9, 128.6, 127.9, 127.7, 127.4, 118.6 (d, ^2^*J*_CF_ = 20.2 Hz), 116.5 (d, ^3^*J*_CF_ = 2.6 Hz), 113.6 (d, ^2^*J*_CF_ = 12.0 Hz), 43.7, 34.4, 19.5, 19.4, 19.2. ^19^F NMR (376 MHz, CDCl_3_): δ −167.90 (s, 2F). HRMS (ESI) *m*/*z*: [M + H]^+^ calcd. for C_46_H_41_F_2_N_4_O_2_^+^ 719.3192; found 719.3192.

*(5,5′-((2,4,5-Trimethylphenyl)methylene)bis(4-fluoro-3-phenyl-1H-pyrrole-5,2-diyl))bis(morpholinomethanone)* (**4e**). Eluent: Hex/EtOAc (1:1), EtOAc. Yield: 0.261 g (84%). Orange solid; mp 180–182 °C. ^1^H NMR (400 MHz, CDCl_3_): δ 9.40 (br s, 2H), 7.42–7.27 (m, 10H), 7.05 (s, 1H), 6.95 (s, 1H), 5.89 (s, 1H), 3.21 (br s, 16H), 2.29 (s, 3H), 2.18 (s, 3H), 2.14 (s, 3H). ^13^C{^1^H} NMR (100 MHz, CDCl_3_): δ 163.6, 145.5 (d, ^1^*J*_CF_ = 244.0 Hz), 136.0, 134.8, 134.0, 133.2, 132.5, 131.5, 129.3, 129.1, 128.9, 127.7, 117.0 (d, ^2^*J*_CF_ = 21.6 Hz), 116.5 (d, ^3^*J*_CF_ = 3.6 Hz), 114.3 (d, ^2^*J*_CF_ = 10.3 Hz), 65.9, 65.9, 34.1, 19.6, 19.4, 19.0. ^19^F NMR (376 MHz, CDCl_3_): δ −169.47 (s, 2F). HRMS (ESI) *m*/*z*: [M + H]^+^ calcd. for C_40_H_41_F_2_N_4_O_4_^+^ 679.3090; found 679.3090.

*(5,5′-(Phenylmethylene)bis(4-fluoro-3-phenyl-1H-pyrrole-5,2-diyl))bis(morpholinomethanone)* (**4f**). Eluent: Hex/EtOAc (1:1), EtOAc. Yield: 0.125 g (97%). Colourless solid; mp 194–196 °C. ^1^H NMR (400 MHz, CDCl_3_): δ 10.77 (s, 2H), 7.41–7.28 (m, 10H), 7.13 (d, *J* = 7.2 Hz, 2H), 7.10–7.00 (m, 3H), 5.90 (s, 1H), 3.22 (br s, 16H). ^13^C{^1^H} NMR (100 MHz, CDCl_3_): δ 163.9 (d, ^4^*J*_CF_ = 2.1 Hz), 145.9 (d, ^1^*J*_CF_ = 243.9 Hz), 139.5, 131.6 (d, ^3^*J*_CF_ = 2.5 Hz), 129.4, 128.9, 128.6, 127.7, 127.6, 127.2, 117.4 (d, ^2^*J*_CF_ = 22.4 Hz), 117.0 (d, ^3^*J*_CF_ = 3.6 Hz), 113.7 (d, ^2^*J*_CF_ = 11.7 Hz), 65.9, 65.9, 36.1. ^19^F NMR (376 MHz, CDCl_3_): δ −171.02 (s, 2F). HRMS (ESI) *m*/*z*: [M + H]^+^ calcd. for C_37_H_35_F_2_N_4_O_4_^+^ 637.2621; found 637.2621.

*(5,5′-(O-tolylmethylene)bis(4-fluoro-3-phenyl-1H-pyrrole-5,2-diyl))bis(morpholinomethanone)* (**4g**). Eluent: Hex/EtOAc (1:1), EtOAc. Yield: 0.130 g (quant.). Pale pink solid; mp 184–186 °C. ^1^H NMR (400 MHz, CDCl_3_): δ 10.47 (s, 2H), 7.42–7.25 (m, 11H), 7.10 (d, *J* = 7.1 Hz, 1H), 7.04 (t, *J* = 7.2 Hz, 1H), 6.91 (t, *J* = 7.3 Hz, 1H), 5.97 (s, 1H), 3.22 (br s, 16H), 2.33 (s, 3H). ^13^C{^1^H} NMR (100 MHz, CDCl_3_): δ 163.8 (d, ^4^*J*_CF_ = 2.1 Hz), 145.4 (d, ^1^*J*_CF_ = 244.0 Hz), 137.4, 136.1, 131.6 (d, ^3^*J*_CF_ = 2.2 Hz), 130.9, 129.3, 128.8, 127.9, 127.5, 127.4, 126.4, 117.0 (d, ^2^*J*_CF_ = 21.3 Hz), 116.8 (d, ^3^*J*_CF_ = 3.1 Hz), 113.7 (d, ^2^*J*_CF_ = 11.7 Hz), 65.9, 65.9, 34.0, 19.6. ^19^F NMR (376 MHz, CDCl_3_): δ −170.16 (s, 2F). HRMS (ESI) *m*/*z*: [M + H]^+^ calcd. for C_38_H_37_F_2_N_4_O_4_^+^ 651.2777; found 651.2777.

*(5,5′-((2,4-Dichlorophenyl)methylene)bis(4-fluoro-3-phenyl-1H-pyrrole-5,2-diyl))bis(morpholinomethanone)* (**4h**). Eluent: Hex/EtOAc (1:1), EtOAc. Yield: 0.141 g (quant.). Powdery solid; mp 195–197 °C. ^1^H NMR (400 MHz, CDCl_3_): δ 10.87 (s, 2H), 7.42–7.25 (m, 12H), 6.93 (dd, *J* = 8.4, 2.0 Hz, 1H), 6.19 (s, 1H), 3.22 (br s, 16H). ^13^C{^1^H} NMR (100 MHz, CDCl_3_): δ 163.7 (d, ^4^*J*_CF_ = 2.0 Hz), 145.7 (d, ^1^*J*_CF_ = 245.0 Hz), 135.7, 134.3, 133.6, 131.4 (d, ^3^*J*_CF_ = 2.3 Hz), 130.7, 129.6, 129.0, 128.9, 127.6, 127.3, 117.1 (d, ^3^*J*_CF_ = 3.7 Hz), 115.6 (d, ^2^*J*_CF_ = 21.4 Hz), 113.5 (d, ^2^*J*_CF_ = 11.6 Hz), 65.8, 65.8, 34.4. ^19^F NMR (376 MHz, CDCl_3_): δ −169.19 (s, 2F). HRMS (ESI) *m*/*z*: [M + H]^+^ calcd. for C_37_H_33_Cl_2_F_2_N_4_O_4_^+^ 705.1841; found 705.1841.

*(5,5′-((2,5-Dimethoxyphenyl)methylene)bis(4-fluoro-3-phenyl-1H-pyrrole-5,2-diyl))bis(morpholinomethanone)* (**4i**). Eluent: Hex/EtOAc (1:1), EtOAc. Yield: 0.138 g (quant.). Sandy solid; mp 212–214 °C. ^1^H NMR (400 MHz, CDCl_3_): δ 10.38 (s, 2H), 7.39–7.23 (m, 10H), 6.87 (d, *J* = 2.9 Hz, 1H), 6.83 (d, *J* = 8.9 Hz, 1H), 6.71 (dd, *J* = 8.9, 3.0 Hz, 1H), 6.04 (s, 1H), 3.77 (s, 3H), 3.61 (s, 3H), 3.19 (br s, 16H). ^13^C{^1^H} NMR (100 MHz, CDCl_3_): δ 163.7 (d, ^4^*J*_CF_ = 2.0 Hz), 153.6, 151.1, 145.2 (d, ^1^*J*_CF_ = 244.0 Hz), 131.7 (d, ^3^*J*_CF_ = 2.2 Hz), 129.3, 129.2, 128.7, 127.3, 116.7 (d, ^2^*J*_CF_ = 22.1 Hz), 116.5, 116.5 (d, ^3^*J*_CF_ = 3.9 Hz), 113.6 (d, ^2^*J*_CF_ = 11.7 Hz), 112.4, 111.7, 65.9, 65.9, 56.6, 55.5, 32.1. ^19^F NMR (376 MHz, CDCl_3_): δ −170.19 (s, 2F). HRMS (ESI) *m*/*z*: [M + H]^+^ calcd. for C_39_H_39_F_2_N_4_O_6_^+^ 697.2832; found 697.2832.

*(5,5′-((2,4,5-Trimethoxyphenyl)methylene)bis(4-fluoro-3-phenyl-1H-pyrrole-5,2-diyl))bis(morpholinomethanone)* (**4j**). Eluent: Hex/EtOAc (1:1), EtOAc, EtOAc/EtOH (98:2). Yield: 0.140 g (96%). Pale orange solid; mp 192–194 °C. ^1^H and ^19^F NMR spectra of the sample are complex due to the presence of two sets of signals corresponding to two stable rotamers in a ratio of 75:25. In ^1^H spectra some similar signals overlap. ^1^H NMR (400 MHz, CDCl_3_): δ 11.27 (br s, 1H), 10.04 (br s, 1H), 7.43–7.20 (m, 10H), 7.05 (s, 1H), 6.60 (s, 1H), 5.95 (s, 1H), 3.88 (s, 3H), 3.86 (s, 3H), 3.78 (s, 3H), 3.20 (br s, 16H). ^13^C{^1^H} NMR (100 MHz, CDCl_3_): δ 163.8 (d, ^4^*J*_CF_ = 1.0 Hz), 151.1, 149.3, 145.3 (d, ^1^*J*_CF_ = 243.8 Hz), 143.5, 131.7 (d, ^3^*J*_CF_ = 2.5 Hz), 128.9, 128.8, 128.6, 127.4, 127.3, 119.4, 117.1 (d, ^2^*J*_CF_ = 21.4 Hz), 116.3 (d, ^3^*J*_CF_ = 3.6 Hz), 114.1, 113.2 (d, ^2^*J*_CF_ = 11.5 Hz), 66.0, 65.8, 57.3, 56.6, 56.2. ^19^F NMR (376 MHz, CDCl_3_): major rotamer δ −170.4 (s, 1F); minor rotamer δ -171.9 (s, 1F). HRMS (ESI) *m*/*z*: [M + H]^+^ calcd. for C_40_H_41_F_2_N_4_O_7_^+^ 727.2938; found 727.2938.

**General Procedure for the Preparation of BODIPYs 5.**

In a typical experiment, a selected dipyrromethane **4** (0.15 mmol, 1 mol equiv) and DDQ (0.102 g, 0.45 mmol, 3 mol equiv) were loaded into a screw cap vial (30 mL) for the microwave reactor Nova-2S (PreeKem) and dissolved in MeCN (7.5 mL). The reaction mixture was heated under microwave irradiation at 60 °C for 15 min with stirring. After completion of the oxidation step (monitoring by TLC or ^19^F NMR spectroscopy), the reaction mixture was cooled to room temperature. Then, triethylamine (0.209 mL, 1.5 mmol, 10 mol equiv) was added and the resulting mixture was stirred for 3 min. Next, BF_3_⋅Et_2_O (0.278 mL, 2.25 mmol, 15 mol equiv) was added to the reaction mixture. The vial with the resulting mixture was purged with argon and then heated under microwave irradiation at 60 °C for 40 min with stirring. After completion of the reaction (monitoring by TLC or ^19^F NMR spectroscopy), the reaction mixture was diluted with EtOAc (100 mL) and extracted with water (5 × 100 mL). Then, the organic layer was separated, dried over Na_2_SO_4_, filtered and concentrated under a vacuum. The pure product was isolated by column chromatography on silica gel using appropriate eluents.

*3,7-Bis(butylcarbamoyl)-1,5,5,9-tetrafluoro-2,8-diphenyl-10-(2,4,5-trimethylphenyl)-5H-dipyrrolo[1,2-c:2′,1′-f][1,3,2]diazaborinin-4-ium-5-uide* (**5a**). The pure product was isolated by column chromatography with gradient elution on silica gel using SepaBean™ machine T (Santai Science Inc., Montréal, QC, Canada). Polarity of the elution system was gradually increased from DCM/EtOAc (98:2) to DCM/EtOAc (96:4). Yield: 0.031 g (27%). Red solid; mp 219–221 °C. ^1^H NMR (400 MHz, CDCl_3_): δ 7.46–7.40 (m, 4H), 7.38–7.27 (m, 6H), 7.09 (s, 1H), 7.02 (s, 1H), 6.64 (br s, 2H), 3.45 (q, J = 6.8 Hz, 4H), 2.29 (s, 3H), 2.25 (s, 6H), 1.60 (dt, J = 15.0, 7.5 Hz, 4H), 1.44–1.33 (m, 4H), 0.94 (t, J = 7.4 Hz, 6H). ^13^C NMR (100MHz, CDCl_3_): δ 160.6, 159.4 (d, ^1^J_CF_ = 287.5 Hz), 147.5, 146.7, 139.0, 134.4, 132.6, 131.8, 128.8, 128.6, 128.6, 128.4, 128.0 (d, ^3^J_CF_ = 1.8 Hz), 127.3, 121.0 (d, ^2^J_CF_ = 11.2 Hz), 118.3 (d, ^2^J_CF_ = 7.4 Hz), 40.1, 31.2, 20.2, 19.8, 19.4, 19.2, 13.8. ^19^F NMR (376 MHz, CDCl_3_): δ −132.19 (s, 2F), −136.36–−136.81 (m, 2F). HRMS (ESI) *m*/*z*: [M + H]^+^ calcd. for C_40_H_42_BF_4_N_4_O_2_^+^ 697.3331; found 697.3331.

*3,7-Bis(cyclopropylcarbamoyl)-1,5,5,9-tetrafluoro-2,8-diphenyl-10-(2,4,5-trimethylphenyl)-5H-dipyrrolo[1,2-c:2′,1′-f][1,3,2]diazaborinin-4-ium-5-uide* (**5b**). The pure product was isolated by column chromatography with gradient elution on silica gel using SepaBean™ machine T (Santai Science Inc., Montréal, QC, Canada). Polarity of the elution system was gradually increased from DCM/EtOAc (98:2) to DCM/EtOAc (96:4). Yield: 0.028 g (30%). Red solid; mp 195–197 °C. ^1^H NMR (400 MHz, CDCl_3_): δ 7.46–7.40 (m, 4H), 7.40–7.28 (m, 6H), 7.09 (s, 1H), 6.99 (s, 1H), 6.70 (s, 1H), 6.69 (s, 1H), 2.89 (dt, *J* = 10.5, 3.5 Hz, 2H), 2.29 (s, 3H), 2.24 (s, 3H), 2.23 (s, 3H), 0.91–0.85 (m, 4H), 0.75–0.68 (m, 4H). ^13^C{^1^H} NMR (100 MHz, CDCl_3_): δ 162.0, 159.4 (d, ^1^*J*_CF_ = 282.3 Hz), 147.2, 146.9, 139.1, 134.4, 132.5, 131.9, 128.8, 128.7, 128.6, 128.5, 127.9 (d, ^3^*J*_CF_ = 2.0 Hz), 127.2, 121.0 (d, ^2^*J*_CF_ = 12.7 Hz), 118.4 (d, ^2^*J*_CF_ = 8.8 Hz), 23.1, 19.8, 19.3, 19.1, 6.8. ^19^F NMR (376 MHz, CDCl_3_): δ −131.80 (s, 2F), −135.74–−136.17 (m, 2F). HRMS (ESI) *m*/*z*: [M + Na]^+^ calcd. for C_38_H_33_BF_4_N_4_NaO_2_^+^ 687.2525; found 687.2525.

*3,7-Bis(allylcarbamoyl)-1,5,5,9-tetrafluoro-2,8-diphenyl-10-(2,4,5-trimethylphenyl)-5H-dipyrrolo[1,2-c:2′,1′-f][1,3,2]diazaborinin-4-ium-5-uide* (**5c**). The pure product was isolated by column chromatography with gradient elution on silica gel using SepaBean™ machine T (Santai Science Inc., Montréal, QC, Canada). Polarity of the elution system was gradually increased from DCM/EtOAc (98:2) to DCM/EtOAc (96:4). Yield: 0.018 g (20%). Red solid; mp 253–255 °C (with decomp.). ^1^H NMR (400 MHz, CDCl_3_): δ 7.43 (d, *J* = 6.9 Hz, 4H), 7.39–7.28 (m, 6H), 7.10 (s, 1H), 7.03 (s, 1H), 6.73 (br s, 2H), 5.90 (ddt, *J* = 22.6, 10.8, 5.7 Hz, 2H), 5.28 (dd, *J* = 17.2, 1.0 Hz, 2H), 5.18 (dd, *J* = 10.3, 0.9 Hz, 2H), 4.09 (t, *J* = 5.7 Hz, 4H), 2.30 (s, 3H), 2.26 (s, 6H). ^13^C{^1^H} NMR (100 MHz, CDCl_3_): δ 160.5, 159.5 (d, ^1^*J*_CF_ = 285.0 Hz), 147.1, 147.0, 139.1, 134.4, 133.1, 132.6, 131.9, 128.8, 128.7, 128.6, 128.5, 127.9 (d, ^3^*J*_CF_ = 1.5 Hz), 127.3, 121.1 (d, ^2^*J*_CF_ = 13.5 Hz), 118.55 (d, ^2^*J*_CF_ = 9.7 Hz), 117.4, 42.7, 19.9, 19.4, 19.2. ^19^F NMR (376 MHz, CDCl_3_): δ −131.91 (s, 2F), −135.64–−136.76 (m, 2F). HRMS (ESI) *m*/*z*: [M + H]^+^ calcd. for C_38_H_34_BF_4_N_4_O_2_^+^ 665.2705; found 665.2706.

*3,7-Bis(benzylcarbamoyl)-1,5,5,9-tetrafluoro-2,8-diphenyl-10-(2,4,5-trimethylphenyl)-5H-dipyrrolo[1,2-c:2′,1′-f][1,3,2]diazaborinin-4-ium-5-uide* (**5d**). The pure product was isolated by column chromatography with gradient elution on silica gel using SepaBean™ machine T (Santai Science Inc., Montréal, QC, Canada). Polarity of the elution system was gradually increased from DCM/EtOAc (99:1) to DCM/EtOAc (17:1). Yield: 0.016 g (51%), 0.045 g (46%). Red solid; mp 223–225 °C. ^1^H NMR (400 MHz, CDCl_3_): δ 7.41–7.27 (m, 20H), 7.09 (s, 1H), 7.01 (s, 1H), 6.88 (br s, 2H), 4.64 (d, *J* = 5.7 Hz, 4H), 2.29 (s, 3H), 2.25 (s, 3H), 2.24 (s, 3H). ^13^C{^1^H} NMR (100 MHz, CDCl3): δ 160.7, 159.3 (d, ^1^*J*_CF_ = 286.7 Hz), 147.1 (d, ^3^*J*_CF_ = 1.9 Hz), 147.0, 139.1, 137.1, 134.4, 132.6, 131.8, 128.8, 128.7, 128.6, 128.4, 128.2, 128.0 (d, ^3^*J*_CF_ = 3.8 Hz), 127.8, 127.8, 127.2, 121.1 (d, ^2^*J*_CF_ = 11.8 Hz), 118.4 (d, ^2^*J*_CF_ = 9.4 Hz), 44.3, 19.8, 19.3, 19.1.^19^F NMR (376 MHz, CDCl_3_): δ −131.99 (s, 2F), −136.55–−137.14 (m, 2F). HRMS (ESI) *m*/*z*: [M + H]^+^ calcd. for C_46_H_38_BF_4_N_4_O_2_^+^ 765.3018; found 765.3020.

*1,5,5,9-Tetrafluoro-3,7-di(morpholine-4-carbonyl)-2,8-diphenyl-10-(2,4,5-trimethylphenyl)-5H-dipyrrolo[1,2-c:2′,1’-f][1,3,2]diazaborinin-4-ium-5-uide* (**5e**). The pure product was isolated by column chromatography with gradient elution on silica gel using SepaBean™ machine T (Santai Science Inc., Montréal, QC, Canada). Polarity of the elution system was gradually increased from DCM/EtOAc (98:2) to DCM/EtOAc (3:1). Yield: 0.098 g (90%). Red solid; mp 298–300 °C. The NMR spectra of the sample are complex due to the presence of two sets of signals corresponding to two stable rotamers in ratio 1:1, where many similar signals overlap. ^1^H NMR (400 MHz, CDCl_3_): δ 7.43 (d, *J* = 7.4 Hz, 4H), 7.41–7.29 (m, 6H), 7.10 (s, 1H), 7.02 (s, 1H), 3.92–3.26 (m, 12H), 3.04–2.91 (m, 2H), 2.85–2.67 (m, 2H), 2.29 (s, 3H), 2.27 (s, 3H), 2.24 (s, 3H). ^13^C{^1^H} NMR (100 MHz, CDCl_3_): δ 160.8, 160.7, 158.8 (d, ^1^*J*_CF_ = 285.7 Hz), 158.4 (d, ^1^*J*_CF_ = 284.8 Hz), 147.1, 146.7, 146.1, 139.2, 134.3, 132.9, 131.9, 129.2, 129.0, 128.7, 127.9, 127.7 (d, ^3^*J*_CF_ = 2.5 Hz), 127.1, 121.4 (d, ^2^*J*_CF_ = 12.3 Hz), 116.2 (d, ^2^*J*_CF_ = 9.2 Hz), 66.1, 66.0, 47.0, 42.2, 42.2, 19.8, 19.3, 19.2. ^19^F NMR (376 MHz, CDCl_3_): δ −131.57 (s, 1F), −133.29 (s, 1F), −142.27–−143.52 (m, 2F). HRMS (ESI) *m*/*z*: [M + H]^+^ calcd. for C_40_H_38_BF_4_N_4_O_4_^+^ 725.2917; found 725.2917.

*1,5,5,9-Tetrafluoro-3,7-di(morpholine-4-carbonyl)-2,8,10-triphenyl-5H-dipyrrolo[1,2-c:2′,1′-f][1,3,2]diazaborinin-4-ium-5-uide* (**5f**). The pure product was isolated by column chromatography with gradient elution on silica gel using SepaBean™ machine T (Santai Science Inc., Montréal, QC, Canada). Polarity of the elution system was gradually increased from DCM/EtOAc (98:2) to DCM/EtOAc (2:1). Yield: 0.083 g (82%). Red solid; mp 316–318 °C. ^1^H NMR (400 MHz, CDCl_3_): δ 7.63–7.47 (m, 5H), 7.46–7.29 (m, 10H), 3.89–3.79 (m, 2H), 3.78–3.47 (m, 6H), 3.43–3.26 (m, 4H), 3.03–2.91 (m, 2H), 2.73 (t, *J* = 8.4 Hz, 2H). ^13^C NMR (100 MHz, CDCl_3_): δ 160.6, 158.6 (d, ^1^*J*_CF_ = 286.6 Hz), 147.3, 145.9, 131.1, 129.8, 129.0, 128.8, 128.6, 128.5, 127.9, 127.6, 121.0 (d, ^2^*J*_CF_ = 11.9 Hz), 116.4 (d, ^2^*J*_CF_ = 9.5 Hz), 66.1, 66.0, 47.0, 42.2. ^19^F NMR (376 MHz, CDCl_3_): δ −130.54 (s, 2F), −142.77 (dd, *J* = 58.8, 28.7 Hz, 2F). HRMS (ESI) *m*/*z*: [M + H]^+^ calcd. for C_37_H_32_BF_4_N_4_O_4_^+^ 683.2447; found 683.2448.

*1,5,5,9-Tetrafluoro-3,7-di(morpholine-4-carbonyl)-2,8-diphenyl-10-(o-tolyl)-5H-dipyrrolo[1,2-c:2′,1′-f][1,3,2]diazaborinin-4-ium-5-uide* (**5g**). The pure product was isolated by column chromatography with gradient elution on silica gel using SepaBean™ machine T (Santai Science Inc., Montréal, QC, Canada). Polarity of the elution system was gradually increased from DCM/EtOAc (98:2) to DCM/EtOAc (2:1). Yield: 0.081g (77%). Red solid; mp 324–326 °C (with decomp.). The NMR spectra of the sample are complex due to the presence of two sets of signals corresponding to two stable rotamers in ratio 1:1, where many similar signals overlap. ^1^H NMR (400 MHz, CDCl_3_): δ 7.46–7.26 (m, 14H), 3.88–3.78 (m, 2H), 3.78–3.60 (m, 4H), 3.59–3.48 (m, 2H), 3.46–3.26 (m, 4H), 3.04–2.91 (m, 2H), 2.85–2.70 (m, 2H), 2.35 (s, 3H). ^13^C{^1^H} NMR (100 MHz, CDCl_3_): δ 160.6, 160.6, 158.9 (d, ^1^*J*_CF_ = 280.5 Hz), 158.8 (d, ^1^*J*_CF_ = 289.3 Hz), 147.5, 147.2, 145.3, 135.6, 130.5, 130.4, 129.7, 129.0, 128.8, 128.1, 127.8, 127.6, 126.0, 121.3 (d, ^2^*J*_CF_ = 12.3 Hz), 121.2 (d, ^2^*J*_CF_ = 13.5 Hz), 116.3 (d, *J* = 8.6 Hz), 66.1, 66.0, 47.0, 42.2, 42.2, 19.8. ^19^F NMR (376 MHz, CDCl_3_): δ −131.79 (s, 1F), −133.28 (s, 1F), −142.92 (ddd, *J* = 39.5, 28.5, 10.0 Hz, 2F). HRMS (ESI) *m*/*z*: [M + H]^+^ calcd. for C_38_H_34_BF_4_N_4_O_4_^+^ 697.2604; found 697.2604.

*10-(2,4-Dichlorophenyl)-1,5,5,9-tetrafluoro-3,7-di(morpholine-4-carbonyl)-2,8-diphenyl-5H-dipyrrolo[1,2-c:2′,1′-f][1,3,2]diazaborinin-4-ium-5-uide* (**5h**). The pure product was isolated by column chromatography with gradient elution on silica gel using SepaBean™ machine T (Santai Science Inc., Montréal, QC, Canada). Polarity of the elution system was gradually increased from DCM/EtOAc (98:2) to DCM/EtOAc (3:1). The NMR spectra of the sample are complex due to the presence of two sets of signals corresponding to two stable rotamers in ratio 53:47, where many similar signals overlap. Yield: 0.098 g (88%). Violet solid; mp 186–188 °C. ^1^H NMR (400 MHz, CDCl_3_): δ 7.57 (d, *J* = 1.9 Hz, 1H), 7.46–7.31 (m, 12H), 3.88–3.60 (m, 6H), 3.60–3.48 (m, 2H), 3.45–3.26 (m, 4H), 3.06–2.91 (m, 2H), 2.86–2.71 (m, 2H). ^13^C{^1^H} NMR (100 MHz, CDCl_3_): δ 160.4, 158.6 (d, ^1^*J*_CF_ = 283.8 Hz), 158.5 (d, ^1^*J*_CF_ = 285.6 Hz), 148.3, 139.7, 137.3, 133.6, 130.7, 130.1, 129.1, 128.9, 127.9, 127.7, 127.6, 127.4 (d, ^3^*J*_CF_ = 3.4 Hz), 127.3 (d, ^3^*J*_CF_ = 3.9 Hz), 121.2 (d, ^2^*J*_CF_ = 9.4 Hz), 121.1 (d, ^2^*J*_CF_ = 13.1 Hz), 116.6 (d, ^2^*J*_CF_ = 10.2 Hz), 116.5 (d, ^2^*J*_CF_ = 9.4 Hz), 66.1, 66.0, 47.0, 42.0, 42.2. ^19^F NMR (376 MHz, CDCl_3_): −132.40 (s, 1F), −132.84 (s, 1F), −141.90–−142.60 (m, 1F), −143.21–−143.90 (m, 1F). HRMS (ESI) *m*/*z*: [M + H]^+^ calcd. for C_37_H_30_BCl_2_F_4_N_4_O_4_^+^ 751.1668; found 751.1669.

*10-(2,4-Dimethoxyphenyl)-1,5,5,9-tetrafluoro-3,7-di(morpholine-4-carbonyl)-2,8-diphenyl-5H-dipyrrolo[1,2-c:2′,1′-f][1,3,2]diazaborinin-4-ium-5-uide* (**5i**). The pure product was isolated by column chromatography with gradient elution on silica gel using SepaBean™ machine T (Santai Science Inc., Montréal, QC, Canada). Polarity of the elution system was gradually increased from DCM/EtOAc (98:2) to DCM/EtOAc (3:1). Yield: 0.086 g (76%). Violet solid; mp 325–327 °C (with decomp.). The NMR spectra of the sample are complex due to the presence of two sets of signals corresponding to two stable rotamers in ratio 1:1, where many similar signals overlap. ^1^H NMR (400 MHz, CDCl_3_): δ 7.47–7.29 (m, 10H), 7.04 (dd, *J* = 9.0, 3.0 Hz, 1H), 6.94 (d, *J* = 9.1 Hz, 1H), 6.86 (d, *J* = 2.1 Hz, 1H), 3.87–3.79 (m, 2H), 3.76 (s, 3H), 3.74 (s, 3H), 3.77–3.58 (m, 4H), 3.57–3.46 (m, 2H), 3.43–3.25 (m, 4H), 3.03–2.89 (m, 2H), 2.80–2.67 (m, 2H). ^13^C{^1^H} NMR (100 MHz, CDCl_3_): δ 160.8, 160.7, 158.6 (d, ^1^*J*_CF_ = 285.8 Hz), 153.4, 151.1, 146.8, 146.6, 142.4, 129.0, 128.7, 127.9, 127.8 (d, ^3^*J*_CF_ = 3.2 Hz), 121.8 (d, ^2^*J*_CF_ = 13.3 Hz), 121.3 (d, ^2^*J*_CF_ = 13.3 Hz), 119.4, 117.4, 116.1 (d, ^2^*J*_CF_ = 7.2 Hz), 116.0 (d, ^2^*J*_CF_ = 8.7 Hz), 115.6, 112.0 (s), 66.1, 66.0, 56.1, 55.9, 47.0, 42.2, 42.1. ^19^F NMR (376 MHz, CDCl_3_): δ −131.24 (s, 1F), −133.17 (s, 1F), −141.53–−142.36 (m, 1F), −143.23–−143.96 (m, 1F). HRMS (ESI) *m*/*z*: [M + Na]^+^ calcd. for C_39_H_35_BF_4_N_4_NaO_6_^+^ 765.2478; found 765.2479.

*1,5,5,9-Tetrafluoro-3,7-di(morpholine-4-carbonyl)-2,8-diphenyl-10-(2,4,5-trimethoxyphenyl)-5H-dipyrrolo[1,2-c:2′,1′-f][1,3,2]diazaborinin-4-ium-5-uide* (**5j**). The pure product was isolated by column chromatography with gradient elution on silica gel using SepaBean™ machine T (Santai Science Inc., Montréal, QC, Canada). Polarity of the elution system was gradually increased from DCM/EtOAc (6:1) to DCM/EtOAc (3:1). Yield: 0.064 g (56%). Violet solid; mp 195–197 °C. The NMR spectra of the sample are complex due to the presence of two sets of signals corresponding to two stable rotamers in ratio 1:1, where many similar signals overlap. ^1^H NMR (400 MHz, CDCl_3_): δ 7.47–7.30 (m, 10H), 6.82 (d, *J* = 2.3 Hz, 1H), 6.59 (s, 1H), 3.97 (s, 3H), 3.79 (s, 3H), 3.88–3.68 (m, 4H), 3.77 (s, 3H), 3.67–3.56 (m, 2H), 3.55–3.44 (m, 2H), 3.41–3.23 (m, 4H), 3.00–2.87 (m, 2H), 2.74–2.62 (m, 2H). ^13^C{^1^H} (100 MHz, CDCl_3_): δ 160.9, 160.8, 158.3 (d, ^1^*J*_CF_ = 283.2 Hz), 158.1 (d, ^1^*J*_CF_ = 285.5 Hz), 152.8, 152.6, 146.1, 145.8, 143.1, 142.8, 129.0, 128.6 (d, ^3^*J*_CF_ = 3.5 Hz), 128.0 (d, ^3^*J*_CF_ = 2.9 Hz), 128.0, 127.9, 127.8, 122.0 (d, ^2^*J*_CF_ = 12.4 Hz), 121.1 (d, ^2^*J*_CF_ = 11.8 Hz), 116.0 (d, ^2^*J*_CF_ = 9.6 Hz), 115.9 (d, ^2^*J*_CF_ = 7.9 Hz), 113.9 (d, *J* = 3.6 Hz), 109.7, 96.4, 66.1, 66.0, 66.0, 56.6, 56.2, 56.2, 47.0, 42.2, 42.1. ^19^F NMR (376 MHz, CDCl_3_): δ −131.45 (s, 1F), −133.70 (s, 1F), −141.55–−142.33 (m, 1F), −143.09–−143.77 (m, 1F). HRMS (ESI) *m*/*z*: [M + Na]^+^ calcd. for C_40_H_37_BF_4_N_4_NaO_7_^+^ 795.2584; found 795.2585.

**Procedure for the Preparation of BODIPYs 5k.**

Diethyl 5,5′-((2,4,5-trimethylphenyl)methylene)bis(4-fluoro-3-phenyl-1*H*-pyrrole-2-carboxylate) (0.090 g, 0.15 mmol, 1 mol equiv) and DDQ (0.102 g, 0.45 mmol, 3 mol equiv) were loaded into a screw cap vial (30 mL) for the microwave reactor Nova-2S (PreeKem) and dissolved in MeCN (7.5 mL). The reaction mixture was heated under microwave irradiation at 80 °C for 2 h with stirring. After completion of the oxidation step (TLC monitoring), the reaction mixture was cooled to room temperature. Then, triethylamine (0.209 mL, 1.5 mmol, 10 mol equiv) was added and the resulting mixture was stirred for 3 min. Next, BF_3_⋅Et_2_O (0.278 mL, 2.25 mmol, 15 mol equiv) was added to the reaction mixture. The vial with the resulting mixture was purged with argon and then heated under microwave irradiation at 80 °C for 2 h with stirring.

After completion of the reaction (TLC monitoring), the reaction mixture was diluted with EtOAc (100 mL) and extracted with water (5 × 100 mL). Then, the organic layer was separated, dried over Na_2_SO_4_, filtered and concentrated under a vacuum. The pure product was isolated by column chromatography with gradient elution on silica gel using SepaBean™ machine T (Santai Science Inc., Montréal, QC, Canada). Polarity of the elution system was gradually increased from Hex to Hex/EtOAc (1:1).

*3,7-Bis(ethoxycarbonyl)-1,5,5,9-tetrafluoro-2,8-diphenyl-10-(2,4,5-trimethylphenyl)-5H-dipyrrolo[1,2-c:2′,1′-f][1,3,2]diazaborinin-4-ium-5-uide* (**5k**). Yield: 0.055 g (57%). Red solid; mp 210–212 °C. ^1^H NMR (400 MHz, CDCl_3_): δ 7.42–7.27 (m, 10H), 7.10 (s, 1H), 7.04 (s, 1H), 4.41 (q, *J* = 7.1 Hz, 4H), 2.29 (s, 3H), 2.27 (s, 3H), 2.26 (s, 3H), 1.30 (t, *J* = 7.1 Hz, 6H). ^13^C{^1^H} NMR (100 MHz, CDCl_3_): δ 161.2, 159.3 (d, ^1^*J*_CF_ = 285.5 Hz), 148.1, 144.2, 139.2, 134.3, 132.7, 131.9, 128.8, 128.6, 128.6, 128.5, 128.0 (d, ^3^*J*_CF_ = 1.4 Hz), 127.3, 121.90 (d, ^2^*J*_CF_ = 10.5 Hz), 118.3 (d, ^2^*J*_CF_ = 10.2 Hz), 62.9, 19.8, 19.3, 19.1, 13.8. ^19^F NMR (376 MHz, CDCl_3_): δ −131.29 (s, 2F), −141.20–−141.92 (m, 1F), −142.12–−142.82 (m, 1F). HRMS (ESI) *m*/*z*: [M + Na]^+^ calcd. for C_36_H_31_BF_4_N_2_NaO_4_^+^ 665.2205; found 665.2207.

## 4. Conclusions

In summary, the efficient synthetic route to novel 1,7-difluoro-BODIPY-3,5-diamides **5** based on sequential transformation on *β*-fluoro-*β*-nitrostyrene **1** was developed. This modular approach allowed the introduction of fluorine atoms strictly into the 1,7-positions of the BODIPY core. A new family of BODIPYs **5** with various amide groups at the 3,5-positions and aryl substituents at the 8-position was prepared in up to 90% yield.

The photophysical properties of the obtained BODIPYs **5** were investigated. The solvatofluorochromic effect was observed upon transition from polar solvents to nonpolar ones. The largest bathochromic (18 nm) and bathofloral shifts (13 nm) for BODIPY **5a** were observed in toluene compared to methanol. In addition, it was found that less polar solvents are more favourable for fluorescent properties. The largest value of Φ*_F_* for **5a** was achieved in THF (0.16), while the lowest value was observed in methanol (0.04). The obtained dyes showed the absorption maxima in the range of 531–545 nm, the emission maxima in the range of 577–597 nm, Φ*_F_* in up to 0.17 and Φ_Δ_ in up to 0.16 in THF solutions.

The photophysical and electrochemical properties of 1,7-difluoro-BODIPY-3,5-diamides were compared with their analogues to study the substituent effect at the 3,5-positions. It was found that the amide groups provide much higher Φ*_F_* (0.16) compared to ethoxycarbonyl ones (0.03), but significantly lower Φ*_F_* compared to methyl ones (0.60). On the other hand, both amide and ester and methyl derivatives showed moderate photosensitizing properties in MeCN (Φ_Δ_ = 0.15–0.22). All compared dyes demonstrated similar electrochemical properties: reduction occurs in two one-electron stages, the first of which is reversible, oxidation is more than one-electron and irreversible. Both the reduction and oxidation potentials increase with a rise of the electron-withdrawing ability of 3,5-substituents from −1.32 to −0.82 V and from 0.95 to 1.29 V, respectively. In turn, DFT studies revealed the influence of 3,5-substituents on the electronic structures of these molecules.

## Figures and Tables

**Table 1:** Photophysical properties of BODIPY **5a** in different solvents.

Entry	Solvent	Dielectric Constant	λ_abs_ (max), nm	λ_em_ (max), nm	Stokes Shift, nm	Φ*_F_* ^1^
1	Toluene	2.4	538	584	46	0.14
2	Chloroform	4.8	534	583	49	0.12
3	THF	7.5	533	577	44	0.16
4	Acetone	21.0	527	575	48	0.09
5	Methanol	32.6	520	571	51	0.04
6	Acetonitrile	36.6	525 ^2^	575	50	0.05
7	DMSO	47.0	531	581	50	0.07

^1^ related to Rhodamin 6G in EtOH; ^2^ ε = 41,706 M^−1^·cm^−1^.

**Table 2:** Photophysical properties of BODIPYs **5a**–**5j.**

Entry	Code	*Meso*-Aryl	Amido Group	λ_abs_ (max), nm ^1^	λ_em_ (max), nm ^1^	Stokes Shift, nm	Φ*_F_* ^1,2^	Φ_Δ_ ^3^
1	**5a**	2,4,5-(Me)_3_C_6_H_2_	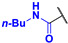	533	577	44	0.16	0.10
2	**5b**		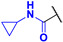	535	577	42	0.09	0.12
3	**5c**		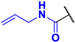	531	577	46	0.14	0.16
4	**5d**		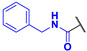	533	577	44	0.17	0.12
5	**5e**		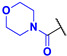	531	577	46	0.12	0.15
6	**5f**	C_6_H_5_	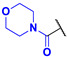	533	584	51	0.01	<0.05
7	**5g**	2-MeC_6_H_4_		533	582	49	0.16	0.10
8	**5h**	2,4-(Cl)_2_C_6_H_3_		545	597	52	0.03	0.09
9	**5i**	2,5-(MeO)_2_C_6_H_3_		535	- ^4^	-	-	-
10	**5j**	2,4,5-(MeO)_3_C_6_H_2_		533	-	-	-	-

^1^ measured in THF; ^2^ related to Rhodamin 6G in EtOH; ^3^ measured in MeCN related to methylene blue; ^4^ not detected.

**Table 3:** Photophysical properties of 3,5-disubstituted core-fluorinated BODIPYs.

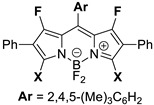	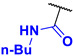	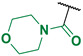	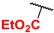	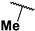
BODIPY	5a	5e	5k	5l
λ_abs (max)_, nm ^1^	533	531	525	517
λ_em (max)_, nm ^1^	577	577	581	552
Stokes shift, nm	44	46	56	35
Φ*_F_* ^1,2^	0.16	0.12	0.03	0.60
Φ_Δ_^3^	0.10	0.15	0.18	0.22

^1^ measured in THF; ^2^ related to Rhodamin 6G in EtOH; ^3^ measured in MeCN related to methylene blue.

**Table 4:** Electrochemical potentials of 3,5-disubstituted core-fluorinated BODIPYs *.

BODIPY	X	E^Red^, V		E^Ox^, V
		E_1/2_, V I	E_pc/2,_ V II	E_pa/2_, V I
**5k**	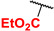	–0.82	–1.75	1.29
**5a**	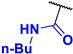	–0.93	–1.84	1.18
**5e**	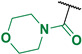	–1.07	–2.13	1.16
**5l**	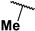	–1.32	–2.30	0.95

* Measured in MeCN with 0.1 M Bu_4_NClO_4_ as supporting electrolyte; potentials were measured relative to Ag|AgCl|KCl aq. sat. reference electrode; the potentials of half-waves are shown for reversible redox processes; the potentials of half-peaks are shown for irreversible redox processes. Potential values are given regarding the potential of the redox process Fc/Fc^+^.

## Data Availability

Data are contained within the article and Supplementary Materials.

## Supplementary Materials

The following supporting information can be downloaded at: https://www.mdpi.com/article/10.3390/ijms26104484/s1.
