# Supplementary material for: Efficient Synthesis of Core-Fluorinated BODIPY-3,5-Diamides
Source: Int J Mol Sci. 2025 May 8;26(10):4484. doi: 10.3390/ijms26104484 (PMC12111513; doi:10.3390/ijms26104484)
Supplement: Supplementary file 1 [file ijms-26-04484-s001.zip › ijms-3610439-supplementary.pdf]

Supplementary Materials

Efficient Synthesis of Core-Fluorinated BODIPY-3,5-Diamides

Victoria E. Shambalova[1], Sofiya R. Mikheeva,[1] Alexander S. Aldoshin[1], Anna A. Moiseeva[1], Evgenia A. Safonova[2], Yulia G. Gorbunova[2,3], Valentine G. Nenajdenko[1,*]

[1] *Department of Chemistry, M. V. Lomonosov Moscow State University, Leninskie Gory 1, Building 3, Moscow 119991, Russia; vshambalova00@gmail.com (V.E.S.); sofiya.mikheeva.02@mail.ru (S.R.M.); aldon2258@mail.ru (A.S.A.); moiseeva.1955@mail.ru (A.A.M.)*

[2] *Frumkin Institute of Physical Chemistry and Electrochemistry, Russian Academy of Sciences, Leninsky pr., 31, building 4, Moscow 119071, Russia; safevgal@mail.ru (E.A.S.); yulia.gorbunova@gmail.com (Y.G.G.)*

[3] *Kurnakov Institute of General and Inorganic Chemistry, Russian Academy of Sciences, Leninsky pr., 31, Moscow 119991, Russia*

* Corresponding Author E-mail: nenajdenko@org.chem.msu.ru

Table of content

Additional experimental data **S2**

Computational details **S14**

Electrochemical measurements **S18**

NMR spectra of obtained compounds **S19**

Additional experimental data

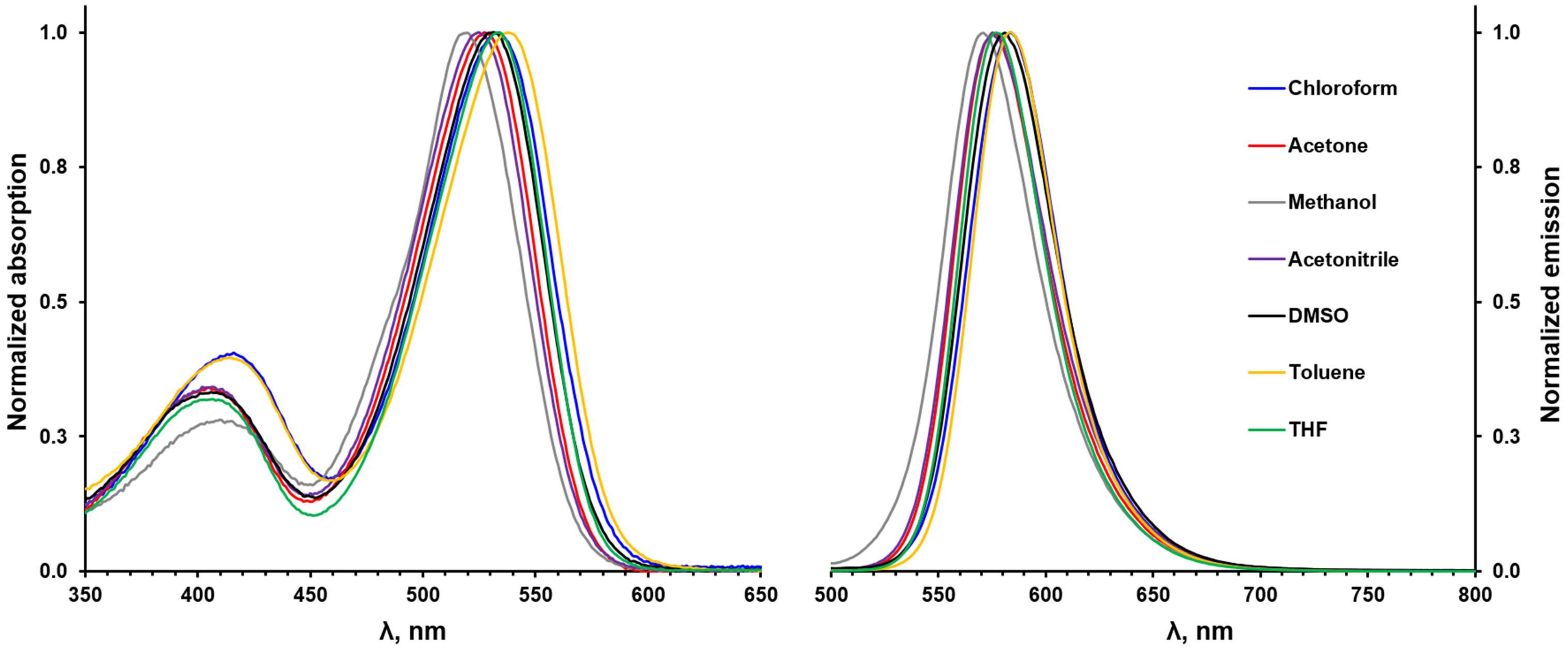

Fig.S1. UV–vis absorption and emission spectra of solutions of BODIPY **5a** in different solvents

Table S1. Photophysical properties of BODIPY **5b** in different solvents

Solvent	λ_{abs} (max), nm	λ_{em} (max), nm	Φ_F
THF	535	577	0.09
Acetonitrile	525 (ε = 34015 $M^{-1} \cdot cm^{-1}$)	577	0.04

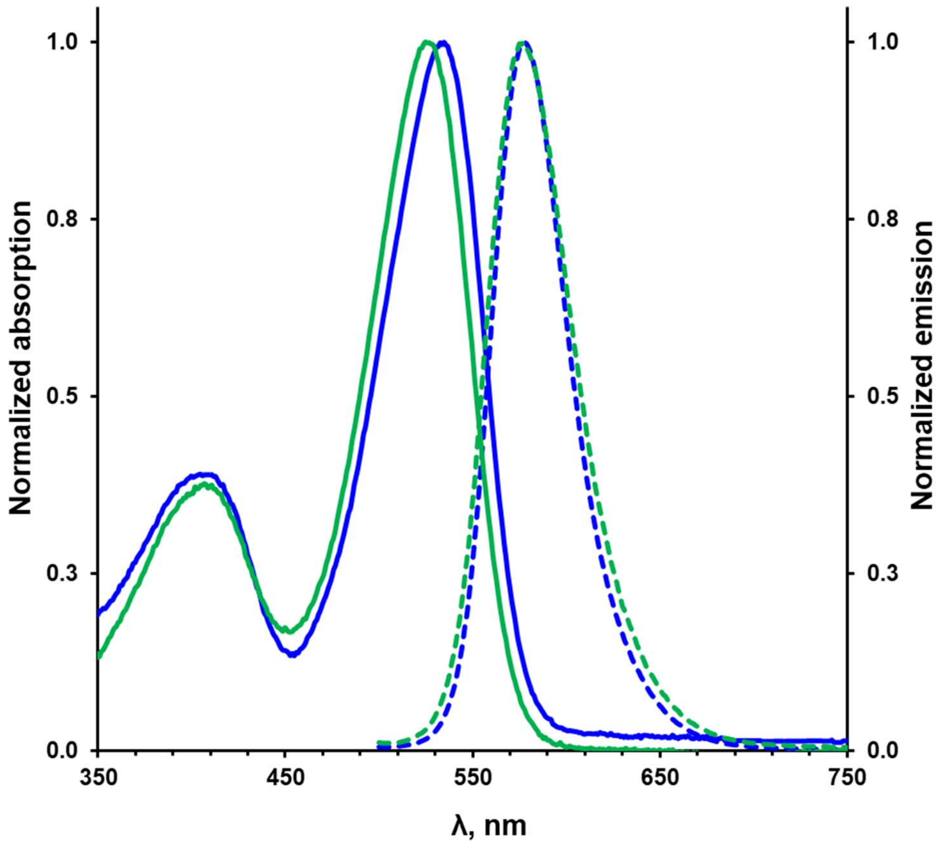

Fig.S2. UV–vis absorption (solid line) and emission (dash line) spectra of solutions of BODIPY **5b** in **THF** (blue) and **Acetonitrile** (green)

Table S2. Photophysical properties of BODIPY **5c** in different solvents

Solvent	λ_{abs} (max), nm	λ_{em} (max), nm	Φ_F
THF	531	577	0.14
Acetonitrile	525 (ε = 37714 $M^{-1}\cdot cm^{-1}$)	577	0.04

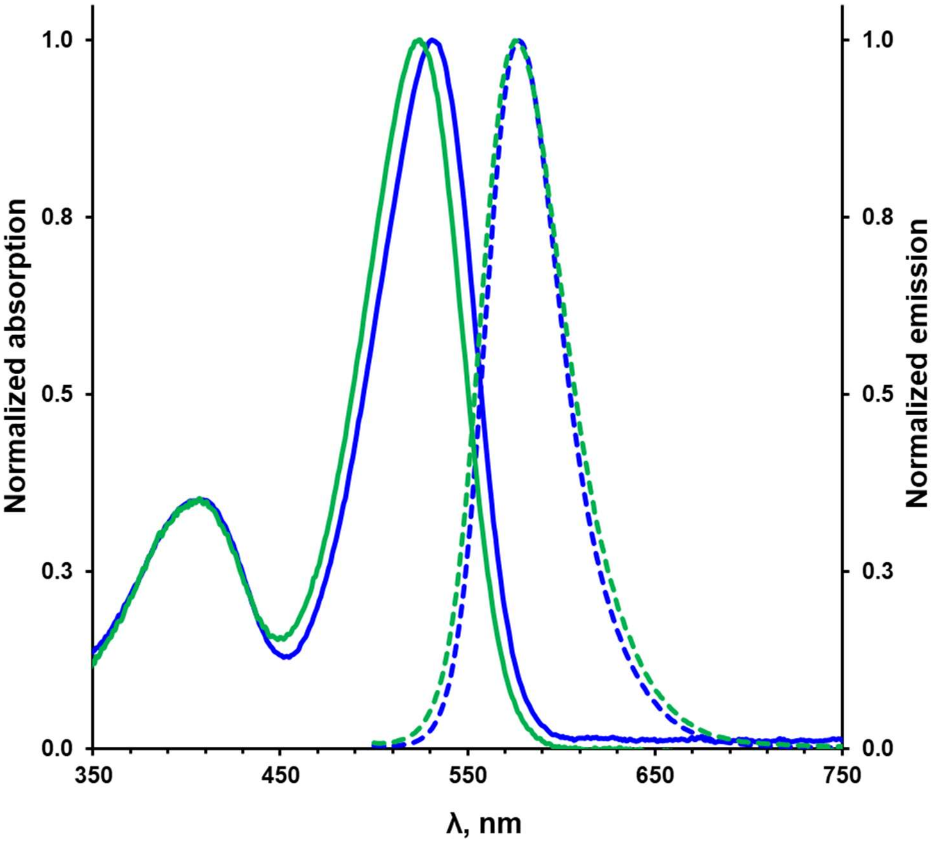

Fig.S3. UV–vis absorption (solid line) and emission (dash line) spectra of solutions of BODIPY **5c** in **THF** (blue) and **Acetonitrile** (green)

Table S3. Photophysical properties of BODIPY **5d** in different solvents

Solvent	λ_{abs} (max), nm	λ_{em} (max), nm	Φ_F
THF	533	577	0.17
Acetonitrile	524 (ε = 38572 $M^{-1}\cdot cm^{-1}$)	577	0.05

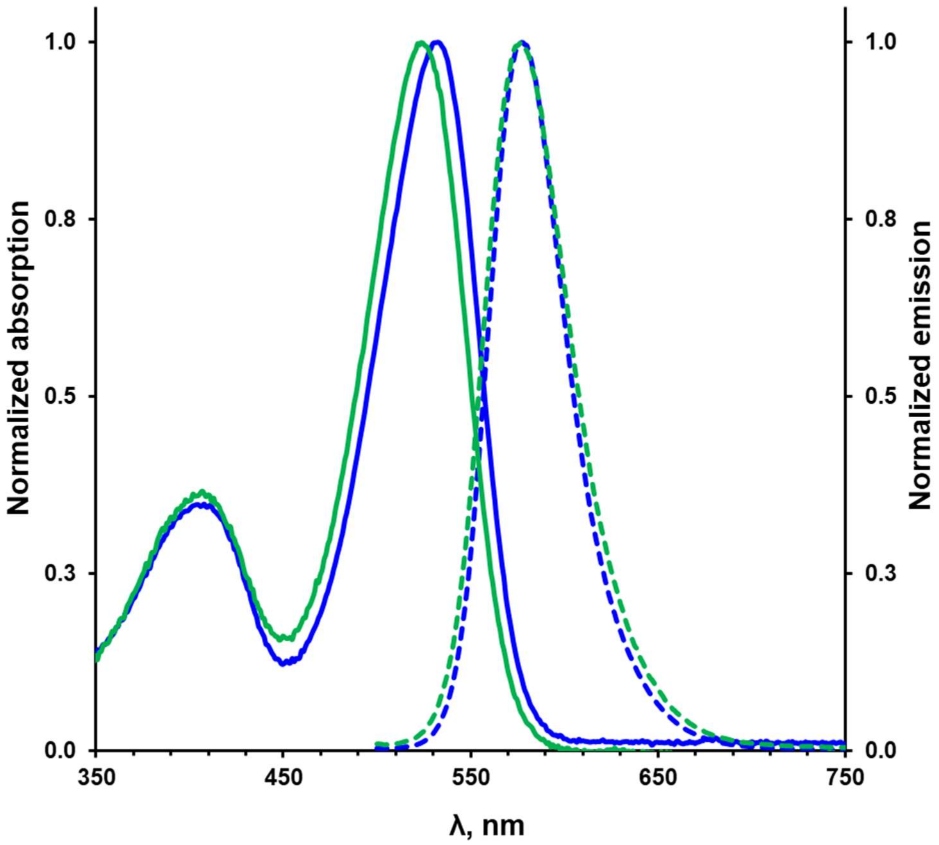

Fig.S4. UV–vis absorption (solid line) and emission (dash line) spectra of solutions of BODIPY **5d** in **THF** (blue) and **Acetonitrile** (green)

Table S4. Photophysical properties of BODIPY **5e** in different solvents

Solvent	λ_{abs} (max), nm	λ_{em} (max), nm	Φ_F
THF	531	577	0.12
Acetonitrile	525 (ε = 44347 $M^{-1} \cdot cm^{-1}$)	575	0.06

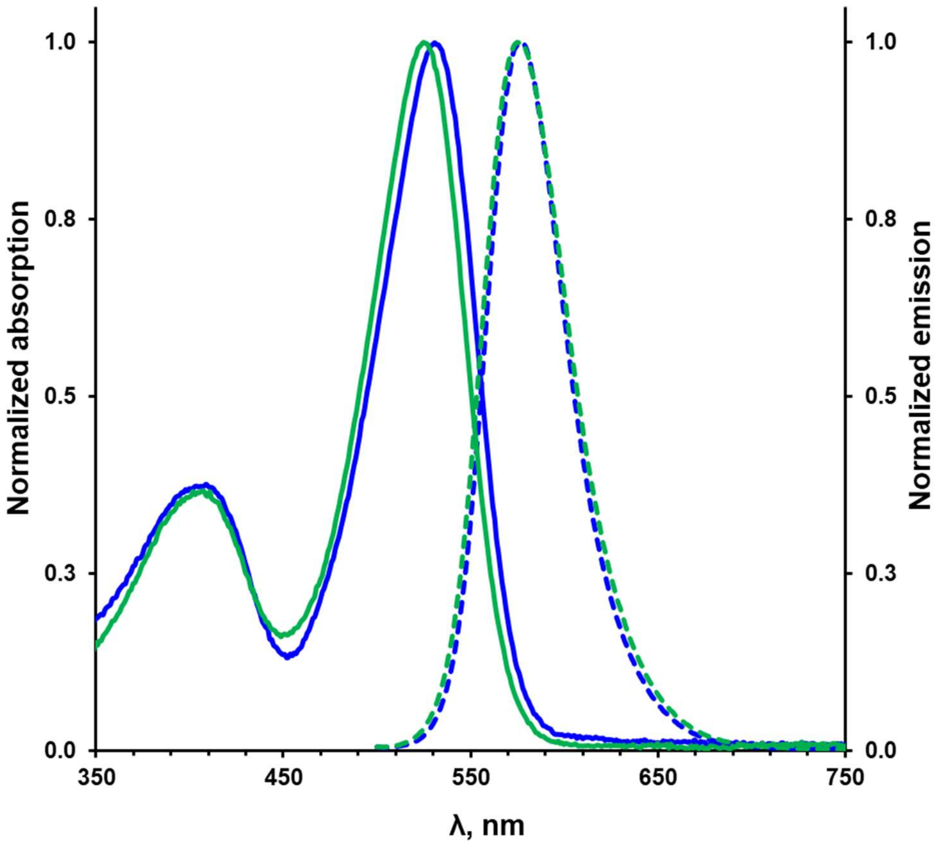

Fig.S5. UV–vis absorption (solid line) and emission (dash line) spectra of solutions of BODIPY **5e** in **THF** (blue) and **Acetonitrile** (green)

Table S5. Photophysical properties of BODIPY **5f** in different solvents

Solvent	λ_{abs} (max), nm	λ_{em} (max), nm	Φ_F
THF	533	584	0.01
Acetonitrile	527 (ε = 46222 $M^{-1}\cdot cm^{-1}$)	579	0.01

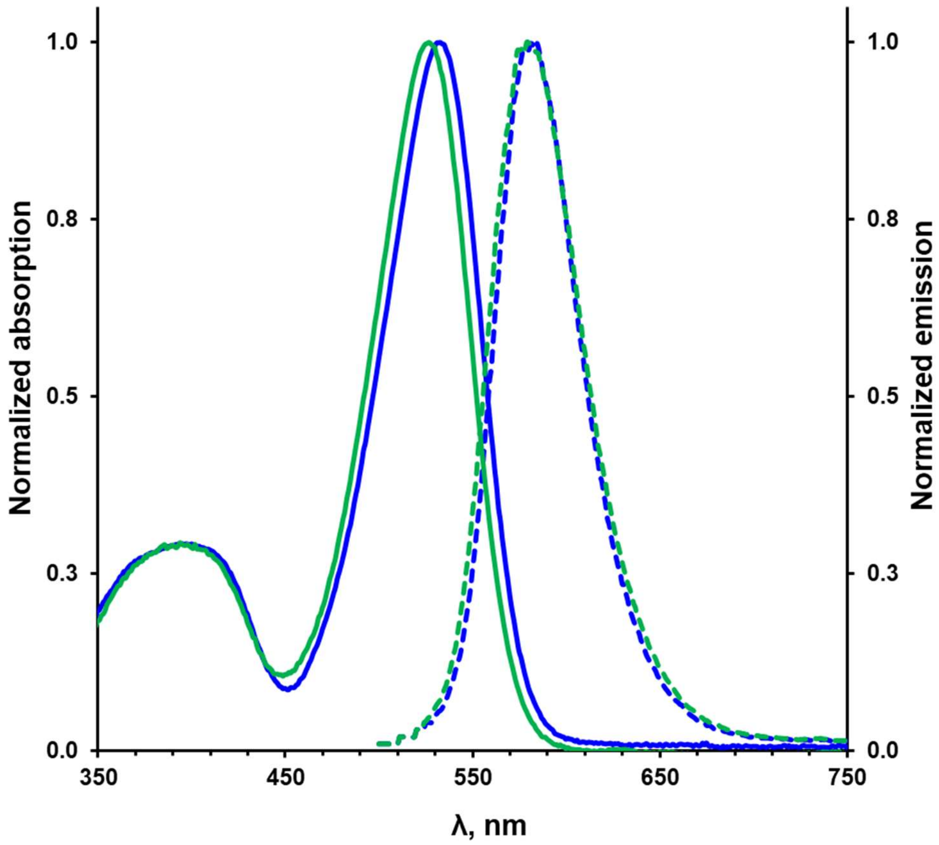

Fig.S6. UV–vis absorption (solid line) and emission (dash line) spectra of solutions of BODIPY **5f** in **THF** (blue) and **Acetonitrile** (green)

Table S6. Photophysical properties of BODIPY **5g** in different solvents

Solvent	λ_{abs} (max), nm	λ_{em} (max), nm	Φ_F
THF	533	582	0.16
Acetonitrile	527 (ε = 47806 $M^{-1}\cdot cm^{-1}$)	578	0.04

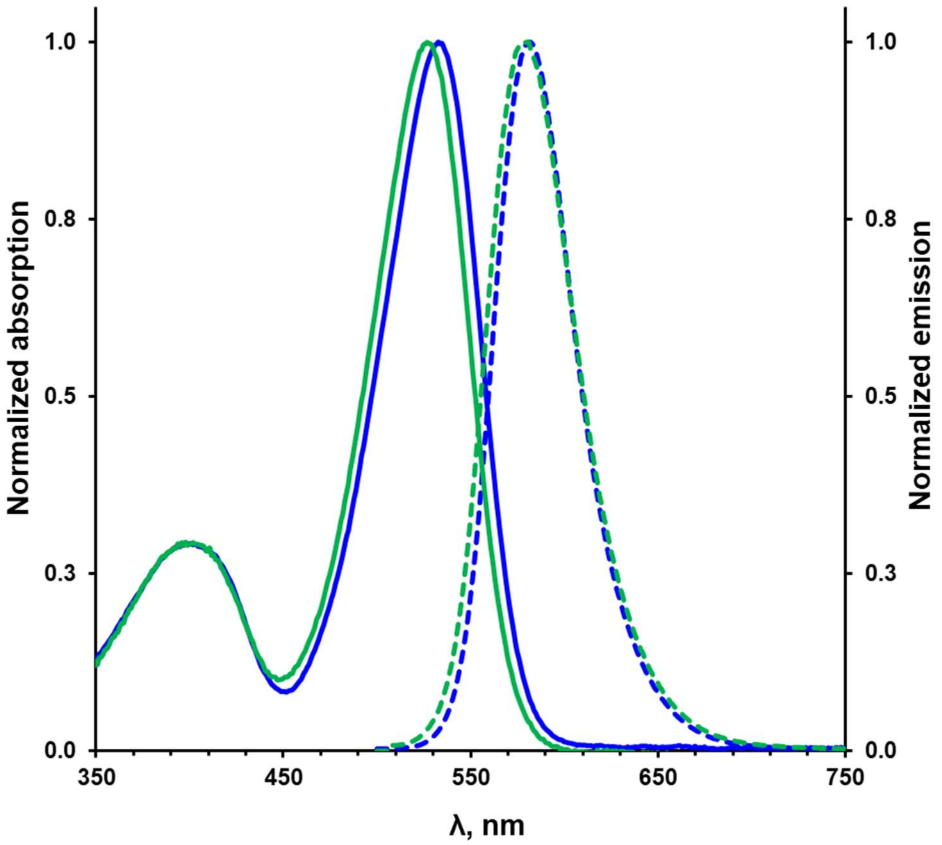

Fig.S7. UV–vis absorption (solid line) and emission (dash line) spectra of solutions of BODIPY **5g** in **THF** (blue) and **Acetonitrile** (green)

Table S7. Photophysical properties of BODIPY **5h** in different solvents

Solvent	λ_{abs} (max), nm	λ_{em} (max), nm	Φ_F
THF	545	597	0.03
Acetonitrile	537 (ε = 46319 $M^{-1}\cdot cm^{-1}$)	595	0.01

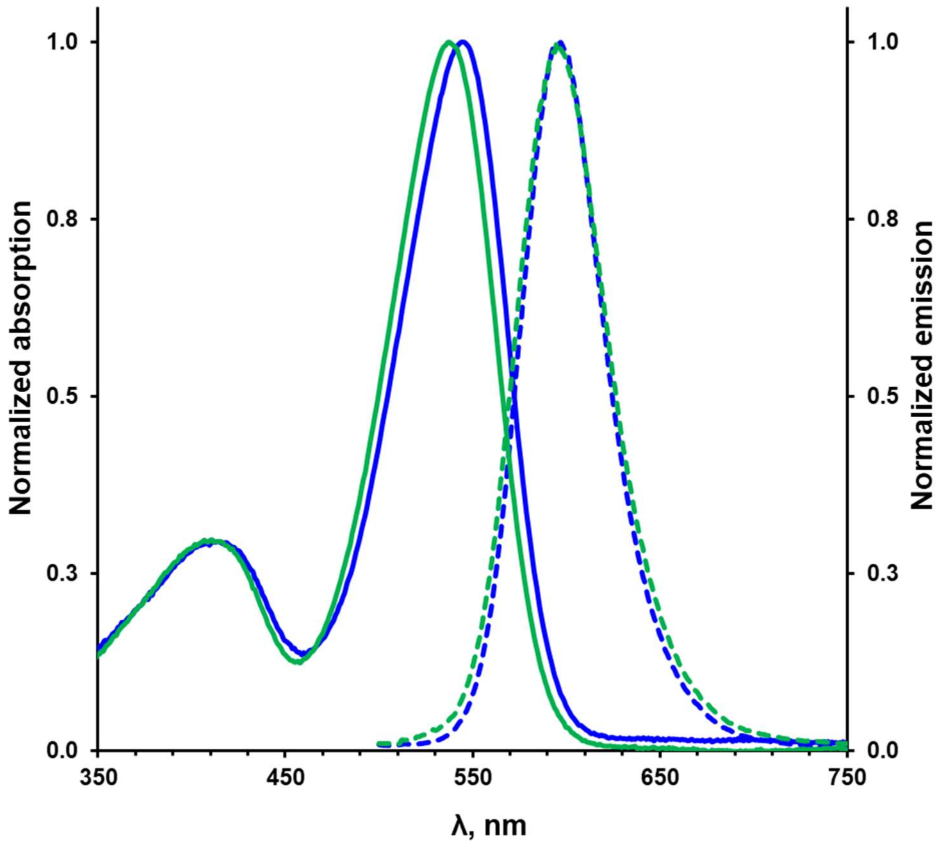

Fig.S8. UV–vis absorption (solid line) and emission (dash line) spectra of solutions of BODIPY **5h** in **THF** (blue) and **Acetonitrile** (green)

Table S8. Photophysical properties of BODIPY **5i** in different solvents

Solvent	λ_{abs} (max), nm
THF	535
Acetonitrile	530 (ε = 48970 $M^{-1}\cdot cm^{-1}$)

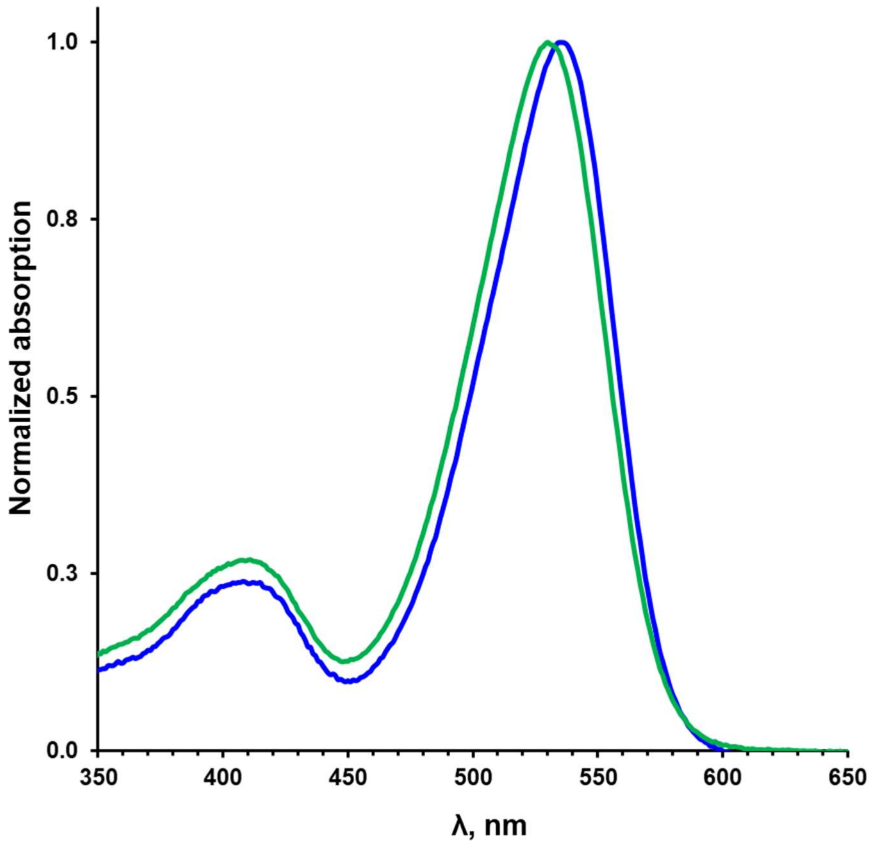

Fig.S9. UV–vis absorption spectra of solutions of BODIPY **5i** in **THF** (blue) and **Acetonitrile** (green)

Table S9. Photophysical properties of BODIPY **5j** in different solvents

Solvent	λ_{abs} (max), nm
THF	533
Acetonitrile	527 (ε = 50353 $M^{-1}\cdot cm^{-1}$)

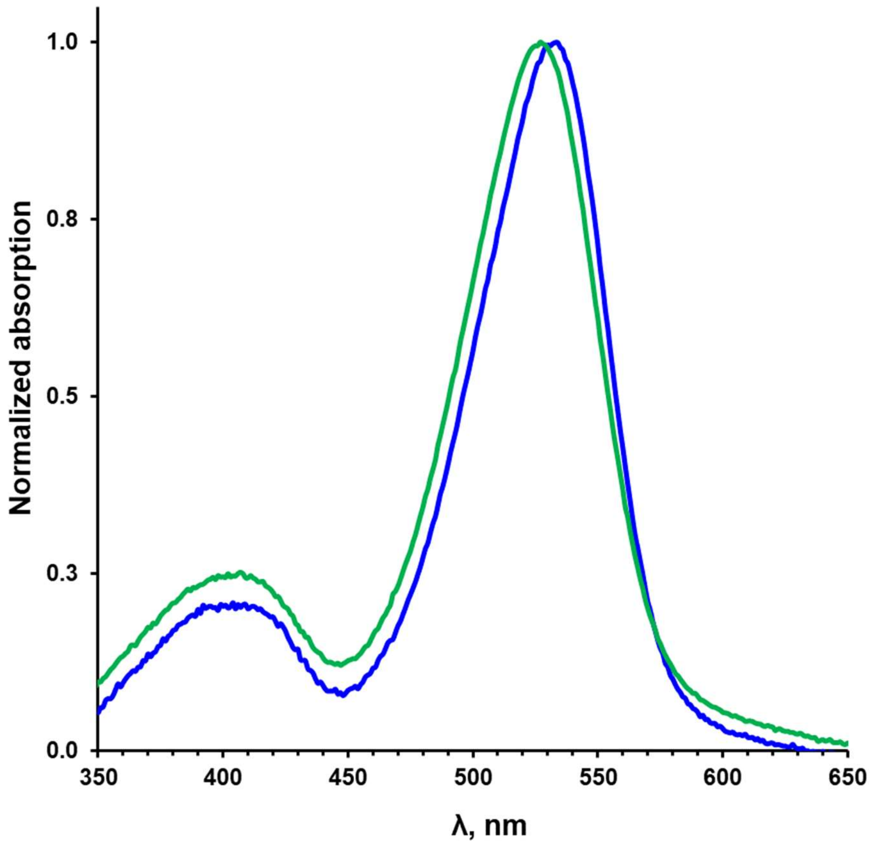

Fig.S10. UV–vis absorption spectra of solutions of BODIPY **5j** in **THF** (blue) and **Acetonitrile** (green)

Table S10. Photophysical properties of BODIPY **5k** in different solvents

Solvent	λ_{abs} (max), nm	λ_{em} (max), nm	Φ_F
THF	525	581	0.03
Acetonitrile	520 (ε = 39419 $M^{-1}\cdot cm^{-1}$)	578	0.01

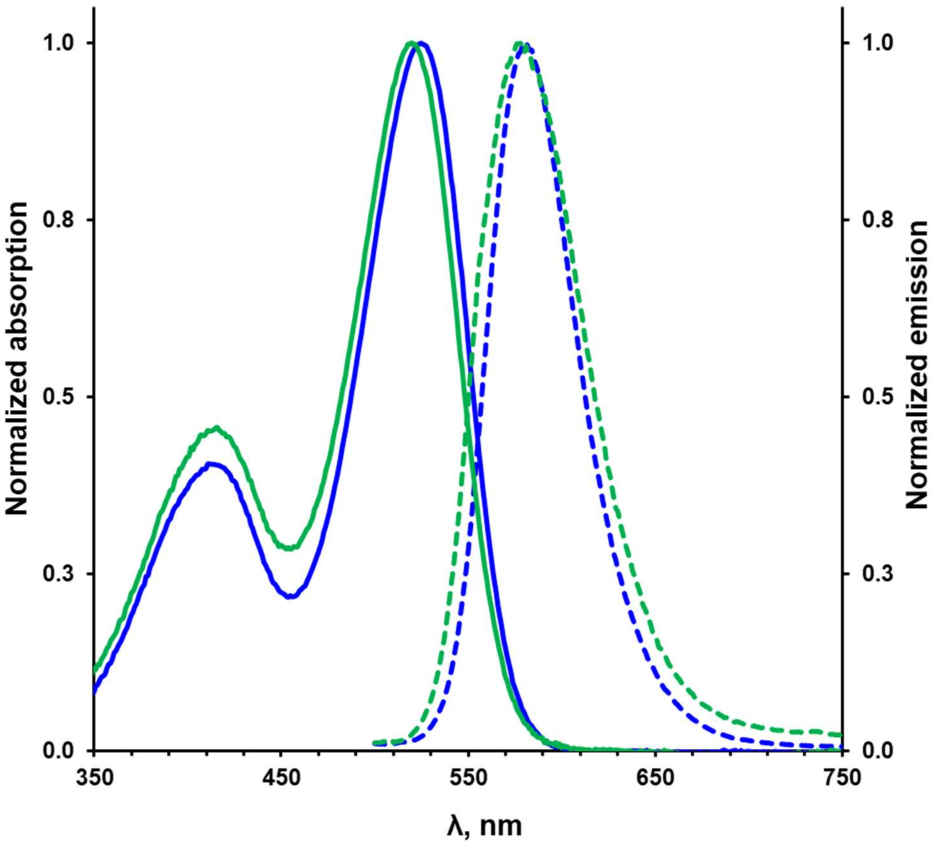

Fig.S11. UV–vis absorption (solid line) and emission (dash line) spectra of solutions of BODIPY **5k** in **THF** (blue) and **Acetonitrile** (green)

Table S11. Photophysical properties of BODIPY **5l** in different solvents

Solvent	λ_{abs} (max), nm	λ_{em} (max), nm	Φ_F
THF	517	552	0.60
Acetonitrile	513 (ε = 60896 $M^{-1}\cdot cm^{-1}$)	552	0.50

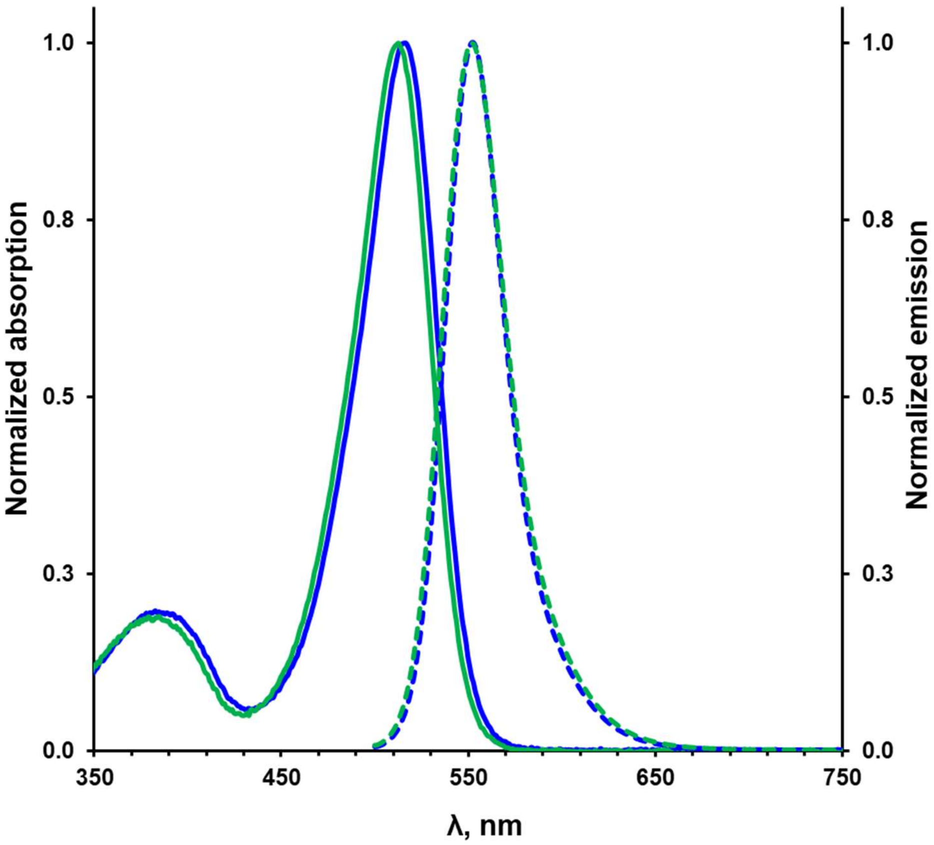

Fig.S12. UV–vis absorption (solid line) and emission (dash line) spectra of solutions of BODIPY **5l** in **THF** (blue) and **Acetonitrile** (green)

Computational details

All structures were optimized at the DFT level in the gas phase using the Gaussian 16 program. Hybrid functional B3LYP and 6-31G(d,p) basis set were used for the geometry optimization and frequency calculations of all structures. All stationary points on the potential energy surface were checked by vibrational analysis and none of them had imaginary frequencies. Single point energy calculations were done using the same functional and 6-311++G(d,p) basis set.

XYZ coordinates

5a

Symbol	X	Y	Z
C	1.2652070	-1.2326750	0.0270850
C	-1.1446710	-1.3125760	-0.1211340
C	0.0844260	-1.9815440	-0.0730630
C	2.6105130	-1.6337680	0.1930570
C	2.5380410	0.5793830	0.3326500
C	-2.4662350	-1.7976350	-0.2893220
C	-3.3634530	-0.7407350	-0.3676670
C	3.4322050	-0.5291600	0.3945460
C	0.1219630	-3.4737420	-0.0883830
C	-0.3278500	-4.1569860	1.0492130
C	0.5823510	-4.1979900	-1.2025510
C	-0.3206530	-5.5492360	1.1315220
H	-0.6861900	-3.5805360	1.8978160
C	0.5678890	-5.5937050	-1.1158120
C	0.1371170	-6.2851080	0.0194790
H	0.9084190	-6.1667800	-1.9752360
C	-4.8294280	-0.8406490	-0.4845850
C	-5.6753350	0.0367490	0.2126000
C	-5.4086080	-1.8523610	-1.2677920
C	-7.0589250	-0.0865560	0.1184280
C	-6.7938170	-1.9748890	-1.3582680
H	-4.7703400	-2.5392230	-1.8127220
C	-7.6245930	-1.0921470	-0.6676760
H	-7.6969810	0.6023510	0.6640890
H	-7.2234400	-2.7598740	-1.9737600
H	-8.7040090	-1.1871900	-0.7397590
C	4.9030920	-0.5873840	0.5473040
C	5.6357570	-1.4550570	-0.2833590
C	5.5937620	0.1568120	1.5179490
C	7.0178030	-1.5691070	-0.1547510
H	5.1181580	-2.0391340	-1.0372070
C	6.9780350	0.0401860	1.6397870
C	7.6956910	-0.8197210	0.8080200
H	7.5646240	-2.2412230	-0.8097750
H	7.4960130	0.6244580	2.3948660
H	8.7737260	-0.9064210	0.9087380

5e

Symbol	X	Y	Z
C	-1.1820020	1.1304680	-0.2505780
C	1.2315500	1.0489140	-0.2333470
C	0.0477990	1.7911720	-0.1192660
C	-2.5163350	1.6024450	-0.2461160
C	-2.5614810	-0.5877150	-0.5912810
C	2.5902520	1.4486660	-0.2960930
C	3.4069500	0.3501810	-0.5504110
C	-3.4038090	0.5560900	-0.4825430
C	0.1165210	3.2586170	0.1463940
C	0.5267540	3.6775980	1.4192810
C	-0.1909930	4.2142660	-0.8386860
C	0.6233350	5.0255780	1.7639050
H	0.7699510	2.9240030	2.1636960
C	-0.0762210	5.5631890	-0.4859680
C	0.3139740	5.9921460	0.7850370
H	-0.2988850	6.3130410	-1.2417040
C	4.8760190	0.3504350	-0.6838320
C	5.6486430	1.2168170	0.1110960
C	5.5330800	-0.4839420	-1.6053330
C	7.0363390	1.2412570	-0.0048720
C	6.9231930	-0.4582020	-1.7117410
H	4.9510990	-1.1384770	-2.2442950
C	7.6805970	0.4008840	-0.9145080
H	7.6151260	1.9158350	0.6194680
H	7.4142300	-1.1075020	-2.4308540
H	8.7628140	0.4191580	-1.0038520
C	-4.8738420	0.6372180	-0.5665240
C	-5.6025160	-0.1585000	-1.4684240
C	-5.5745390	1.5399600	0.2540650
C	-6.9919300	-0.0600940	-1.5312730
H	-5.0762010	-0.8396940	-2.1276900
C	-6.9621440	1.6369560	0.1814900
C	-7.6778790	0.8346130	-0.7089900
H	-7.5384060	-0.6803340	-2.2358910
H	-7.4850240	2.3384260	0.8251880
H	-8.7599120	0.9097070	-0.7639630

N	1.2664390	0.1612120	0.1015780
N	-1.2448370	0.0816680	-0.0875420
B	-0.0167430	1.0414640	-0.0051770
F	-0.0978790	1.8473770	1.1354800
F	0.0158640	1.8231220	-1.1646680
C	-2.5480930	0.4205550	-0.2501320
H	-5.2467970	0.8116540	0.8396150
H	5.0408350	0.8301310	2.1597750
F	-2.7918660	-3.0881660	-0.3550090
F	3.0217950	-2.9022810	0.1570850
C	1.0768930	-3.5238250	-2.4605750
H	2.0952550	-3.1422460	-2.3284360
H	1.0962150	-4.2296410	-3.2948760
H	0.4461650	-2.6778560	-2.7500720
C	0.1590720	-7.7936910	0.0437030
H	0.5315240	-8.1993540	-0.9001260
H	0.8000900	-8.1740010	0.8480840
H	-0.8409340	-8.2090590	0.2162860
C	-0.8022880	-6.2406050	2.3838470
H	-1.6576390	-6.8960150	2.1803490
H	-0.0199990	-6.8701030	2.8245020
H	-1.1117560	-5.5155770	3.1406600
C	2.9025100	2.0275280	0.5483100
C	-3.0472340	1.8293090	-0.4688170
O	-3.8105730	2.0384120	-1.4056210
O	3.5571490	2.3499250	1.5372450
C	2.7223810	4.3275100	-0.3122190
H	3.3853130	4.4827120	0.5415380
H	1.7670130	4.8192030	-0.0811520
C	-2.9995230	4.1712480	0.2876560
C	-3.5992590	4.7591770	1.5687690
H	-2.1022490	4.7356260	-0.0020640
H	-3.7087520	4.2346860	-0.5404380
H	-4.5033490	4.1940000	1.8290750
N	-2.6548690	2.7614290	0.4368100
H	-1.8896390	2.5243250	1.0575570
C	-3.9357390	6.2488890	1.4310130
H	-3.0260650	6.8035820	1.1638050
H	-4.6322630	6.3862830	0.5931900
C	-4.5423400	6.8459110	2.7041270
H	-4.7715500	7.9085670	2.5763190
H	-5.4726780	6.3351050	2.9762460
H	-3.8545430	6.7533830	3.5521650
N	2.5102010	2.8884350	-0.4226610
H	1.8203670	2.5656110	-1.0905950
H	-2.8958100	4.6190060	2.4016160
C	4.1265930	7.0416510	-2.7675690
H	5.1066200	6.6040950	-2.9878280
H	3.4819210	6.8615570	-3.6351790
H	4.2594460	8.1238470	-2.6709190

N	-1.2637130	-0.2488560	-0.4621270
N	1.2276580	-0.3333250	-0.4371840
B	-0.0505100	-1.2258970	-0.2737960
F	-0.0819310	-1.7348660	1.0309690
F	-0.0750100	-2.2309110	-1.2079460
C	2.4979980	-0.7452760	-0.6164920
H	5.1567260	1.8711870	0.8227720
H	-5.0272620	2.1646120	0.9519570
F	3.0123720	2.7069180	-0.1676330
F	-2.8609660	2.8835350	-0.0879320
C	-0.6247220	3.8340650	-2.2349170
H	-1.6927430	3.5924540	-2.2629190
H	-0.4568990	4.6614580	-2.9292360
H	-0.0812790	2.9631200	-2.6121200
C	0.4090420	7.4663620	1.0931180
H	0.1403330	8.0703350	0.2230010
H	-0.2576580	7.7499460	1.9163800
H	1.4232830	7.7504540	1.3981830
C	1.0597580	5.4280370	3.1517560
H	1.9765170	6.0294730	3.1290590
H	0.2979570	6.0342520	3.6564340
H	1.2530340	4.5508900	3.7745050
C	-2.9867520	-1.9976440	-0.9286520
C	2.8276320	-2.1779760	-0.9634050
O	3.0327660	-2.4589070	-2.1420370
O	-3.2113800	-2.2718680	-2.1053550
C	-3.5166100	-4.2592790	-0.1812000
C	-2.9587730	-2.5703860	1.5202930
C	-4.7335860	-4.6318360	0.6651860
H	-2.6746650	-4.9176710	0.0690100
H	-3.7254910	-4.3416820	-1.2484650
C	-4.2070960	-3.0173190	2.2847860
H	-2.0736810	-3.0954740	1.8952420
H	-2.7909350	-1.5006610	1.6521330
H	-4.9539240	-5.6986510	0.5694990
H	-5.6110770	-4.0587090	0.3246340
H	-4.0497910	-2.9122460	3.3620700
H	-5.0637480	-2.3895310	1.9894770
C	2.7546470	-2.7685030	1.4810180
C	3.1792540	-4.4802560	-0.2360460
C	3.9553790	-3.3281420	2.2477540
H	1.8253790	-3.2190000	1.8459830
H	2.6795580	-1.6896190	1.6237870
C	4.3552490	-4.9627910	0.6121750
H	2.2831120	-5.0679320	0.0020500
H	3.3869620	-4.5688680	-1.3030420
H	3.7991530	-3.2223960	3.3251430
H	4.8642580	-2.7714480	1.9661260
H	4.4864470	-6.0431170	0.5053510
H	5.2794500	-4.4610820	0.2828610

C	3.5249790	6.4374640	-1.4954580
H	4.1744700	6.6634490	-0.6392570
H	2.5611850	6.9190070	-1.2812400
C	3.3242500	4.9201220	-1.5898930
H	4.2832040	4.4269000	-1.7925980
H	2.6708540	4.6911560	-2.4436620

5k

Symbol	X	Y	Z
C	-1.2019440	0.7204590	-0.3111910
C	1.2148390	0.6745780	-0.2800520
C	0.0192290	1.4095070	-0.2437750
C	-2.5386170	1.1448830	-0.1120680
C	-2.5371500	-1.0578140	-0.3359860
C	2.5753220	1.0566180	-0.1552480
C	3.3969610	-0.0679630	-0.1793640
C	-3.4002160	0.0502050	-0.1121990
C	0.0463620	2.8896080	-0.0637990
C	0.4746760	3.4051990	1.1671550
C	-0.3460340	3.7673600	-1.0908770
C	0.5150640	4.7746690	1.4261620
H	0.7762690	2.7125990	1.9481860
C	-0.2827500	5.1394480	-0.8261160
C	0.1284180	5.6635120	0.4020590
H	-0.5679790	5.8299540	-1.6166490
C	4.8599920	-0.1222550	-0.0328510
C	5.4822810	-1.2079720	0.6063940
C	5.6652020	0.9216440	-0.5210510
C	6.8686440	-1.2555530	0.7372460
C	7.0502210	0.8727310	-0.3821030
H	5.2018240	1.7677220	-1.0161400
C	7.6588120	-0.2167260	0.2435340
H	7.3312220	-2.1033000	1.2341780
H	7.6562080	1.6863200	-0.7699750
H	8.7390500	-0.2542620	0.3476100
C	-4.8573340	0.0466780	0.0940100
C	-5.6410810	1.1296160	-0.3418070
C	-5.4955020	-1.0272020	0.7377860
C	-7.0204060	1.1302360	-0.1488280
H	-5.1656900	1.9683970	-0.8378870
C	-6.8766130	-1.0251070	0.9225620
C	-7.6454540	0.0519520	0.4799160
H	-7.6092790	1.9740150	-0.4964770
H	-7.3514350	-1.8644380	1.4222780
H	-8.7214110	0.0529170	0.6263960
N	-1.2574020	-0.6675120	-0.4511590
N	1.2175380	-0.7177070	-0.4076750
B	-0.0302980	-1.5140000	-0.9476340
F	-0.0684480	-2.7864770	-0.4106230
F	-0.0016140	-1.5219670	-2.3240720
C	2.4889680	-1.1474120	-0.3517780

N	2.9109560	-3.0711070	0.0575970
N	-3.1329200	-2.8743250	0.0992440
O	-4.4953040	-4.3886070	2.0473850
O	4.1294510	-4.7158700	1.9960130

5l

Symbol	X	Y	Z
C	1.2232520	-0.2590420	0.0821340
C	-1.1883740	-0.2915000	0.0662750
C	0.0075370	0.4365880	0.0953900
C	2.5653650	0.1876860	0.0449130
C	2.5776270	-2.0390270	-0.0158550
C	-2.5447970	0.1182220	0.0889910
C	-3.3817860	-0.9911830	0.0388660
C	3.4330550	-0.8969570	-0.0259580
C	-0.0243040	1.9302330	0.0961240
C	-0.4195520	2.5840190	-1.0782660
C	0.3195450	2.6853490	1.2313550
C	-0.4714540	3.9744830	-1.1757550
H	-0.6885720	1.9842690	-1.9435980
C	0.2490980	4.0786650	1.1281350
C	-0.1293250	4.7402650	-0.0427010
H	0.5005080	4.6737540	2.0033580
C	-4.8547160	-0.9865210	0.0265490
C	-5.5837140	-1.8725940	-0.7856630
C	-5.5683480	-0.0687740	0.8182580
C	-6.9778500	-1.8553750	-0.7899110
C	-6.9610660	-0.0489300	0.8067200
H	-5.0233880	0.6277740	1.4464010
C	-7.6728800	-0.9452660	0.0071450
H	-7.5206300	-2.5485060	-1.4261410
H	-7.4920450	0.6660240	1.4286050
H	-8.7586970	-0.9309550	0.0018880
C	4.9037290	-0.8502450	-0.0929230
C	5.6192630	0.0982860	0.6599230
C	5.6280990	-1.7257830	-0.9206520
C	7.0093270	0.1586350	0.5959450
H	5.0781730	0.7877420	1.2991450
C	7.0199670	-1.6679640	-0.9774920
C	7.7170620	-0.7273290	-0.2186230
H	7.5416580	0.8973280	1.1882200
H	7.5589140	-2.3534060	-1.6251920
H	8.8010080	-0.6813160	-0.2651360
N	1.2861020	-1.6550020	0.0437210
N	-1.2134900	-1.6891100	0.0245090
B	0.0494940	-2.6133240	0.0317460
F	0.0693300	-3.3992690	-1.1116080
F	0.0517850	-3.4064050	1.1780870
C	-2.4949640	-2.1087810	0.0118590

H	4.8759720	-2.0061650	1.0223450
H	-4.9045390	-1.8552630	1.1149010
F	3.0013180	2.3106590	0.0015800
F	-2.9046600	2.4145340	0.0791410
C	-0.8169770	3.2804940	-2.4415880
H	-1.8688280	2.9751120	-2.4067710
H	-0.7313550	4.0743560	-3.1881490
H	-0.2414670	2.4203880	-2.7953090
C	0.1614750	7.1566280	0.6178310
H	-0.1679080	7.6928270	-0.2755580
H	-0.4883490	7.4581020	1.4481450
H	1.1707400	7.5066420	0.8659360
C	0.9709320	5.2818950	2.7727290
H	1.8505970	5.9305700	2.6837170
H	0.1914060	5.8736940	3.2674050
H	1.2335370	4.4552420	3.4374750
C	-2.9169540	-2.4970770	-0.5493000
C	2.8184190	-2.5977110	-0.5702320
O	3.2052450	-3.0362900	-1.6276870
O	2.6327510	-3.3130790	0.5489910
C	2.4905400	-5.3710090	1.7566770
H	1.4864030	-5.1129820	2.1040250
H	2.5643680	-6.4605890	1.6865990
H	3.2132420	-5.0248730	2.5011780
C	2.7637890	-4.7518550	0.4010440
H	3.7685960	-4.9789780	0.0331030
H	2.0443810	-5.0773690	-0.3555090
O	-3.2924960	-2.9310670	-1.6125890
O	-2.7872000	-3.2092400	0.5799720
C	-2.9783880	-4.6422580	0.4409640
H	-2.2703890	-5.0037230	-0.3099040
H	-3.9903830	-4.8285370	0.0695340
C	-2.7388720	-5.2631270	1.8022460
H	-2.8598640	-6.3489030	1.7391490
H	-1.7261020	-5.0468500	2.1529670
H	-3.4492200	-4.8807070	2.5408610
H	-5.0555150	-2.5630290	-1.4352720
H	5.0971010	-2.4398350	-1.5416800
F	-2.9537830	1.3879340	0.1421240
F	2.9352580	1.4719970	0.0767060
C	-2.8369430	-3.5629610	0.0122350
H	-2.6978730	-3.9964250	-0.9848100
H	-2.1694920	-4.0985930	0.6905150
H	-3.8721120	-3.7158570	0.3194350
C	2.9588590	-3.4834450	-0.0253050
H	2.3188260	-4.0391050	0.6632530
H	2.8145930	-3.9186890	-1.0207470
H	4.0031650	-3.6082580	0.2635140
C	0.7532280	2.0456110	2.5291400
H	1.8043820	1.7399660	2.4849070
H	0.6497900	2.7483750	3.3601780
H	0.1649800	1.1540060	2.7650600
C	-0.1745860	6.2482420	-0.0829190
H	0.1145800	6.6785560	0.8791740
H	0.5012990	6.6516180	-0.8466380
H	-1.1784960	6.6165140	-0.3264860
C	-0.8930610	4.6326500	-2.4670570
H	-1.7839540	5.2575540	-2.3308330
H	-0.1070660	5.2864820	-2.8639870
H	-1.1239520	3.8875500	-3.2325080

Electrochemical measurements

5a

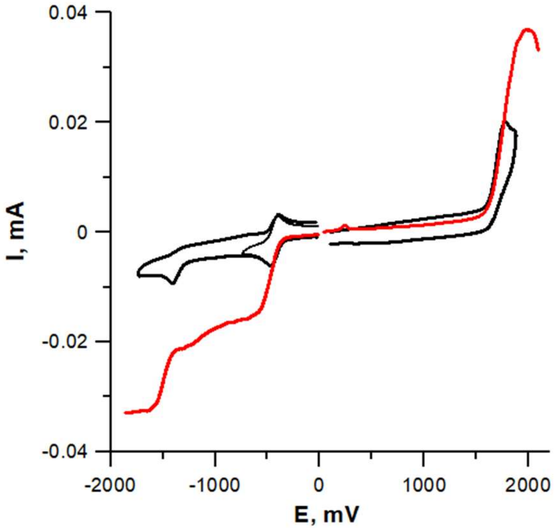

5e

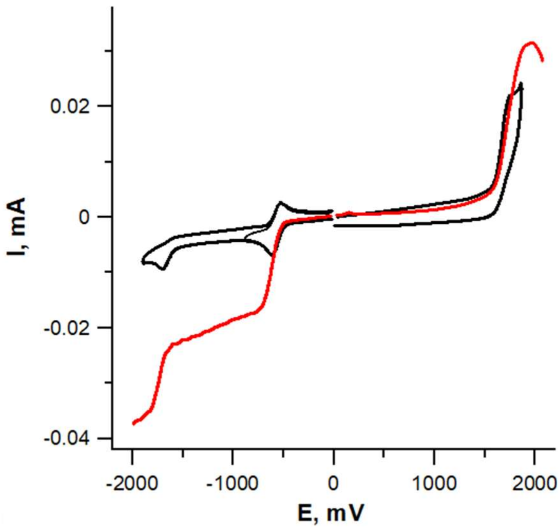

5k

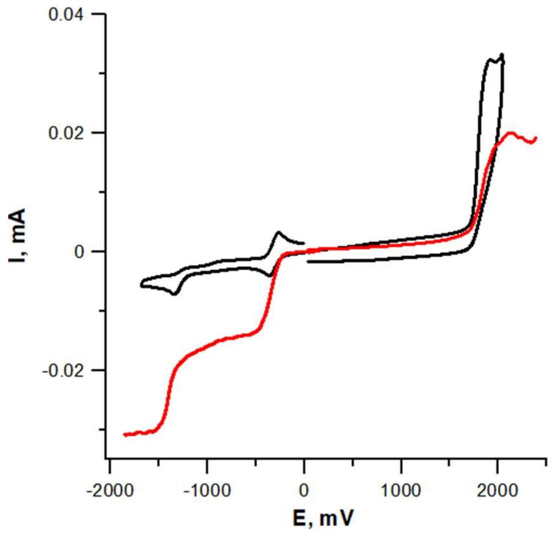

5l

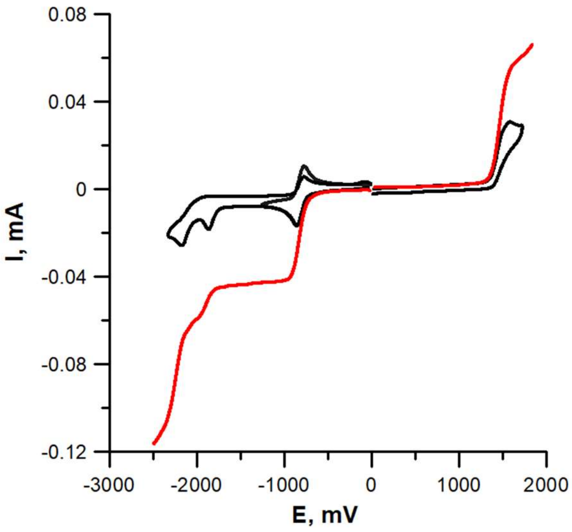

Fig.S13. Current-voltage curves of compounds **5a**, **5e**, **5k** and **5l** (cyclic voltammograms - **black**, obtained on a rotating disk electrode - **red**)

NMR spectra of obtained compounds

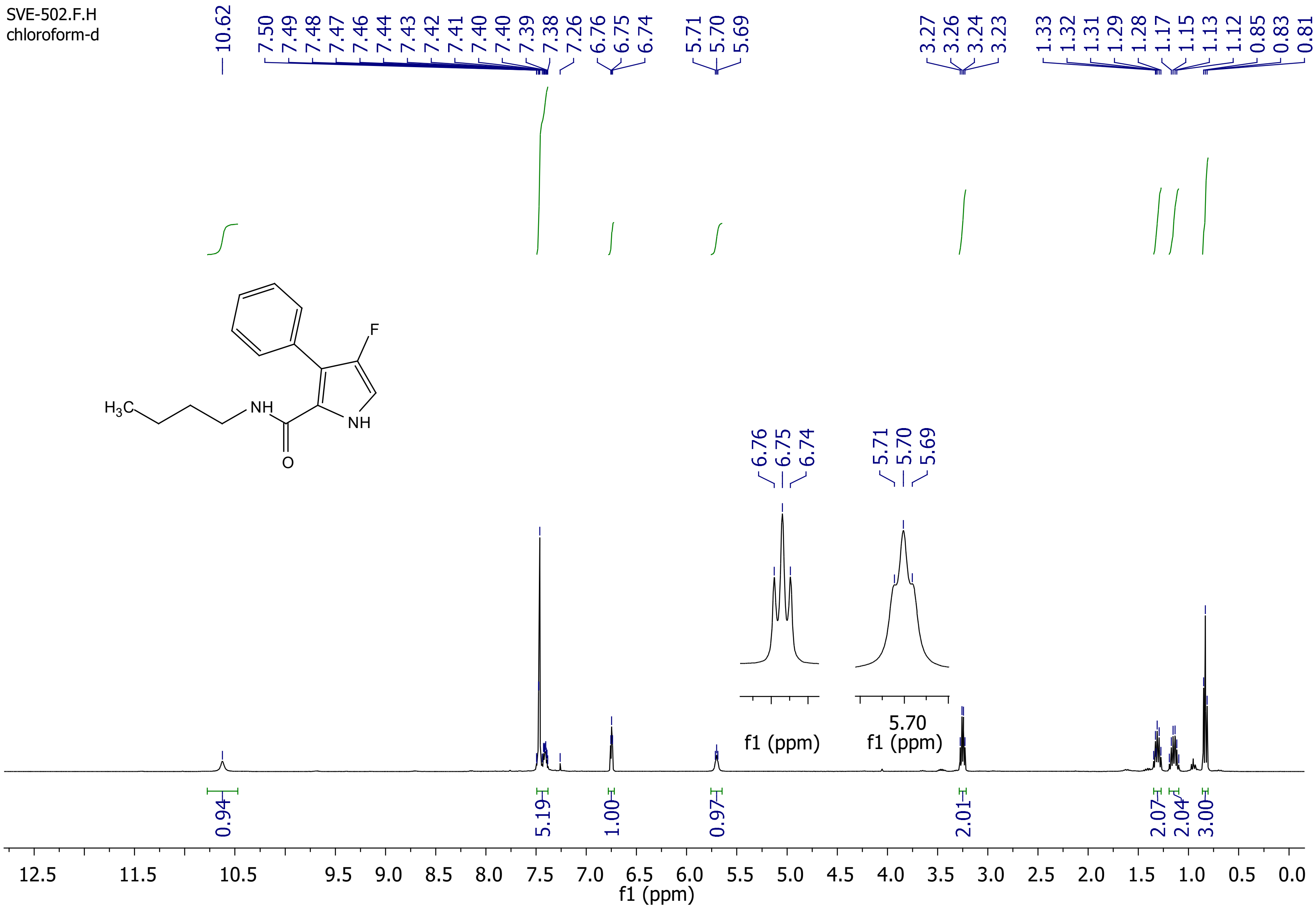

^{1}H NMR spectrum of *N*-butyl-4-fluoro-3-phenyl-1*H*-pyrrole-2-carboxamide (**2a**) in $CDCl_3$ at 400 MHz

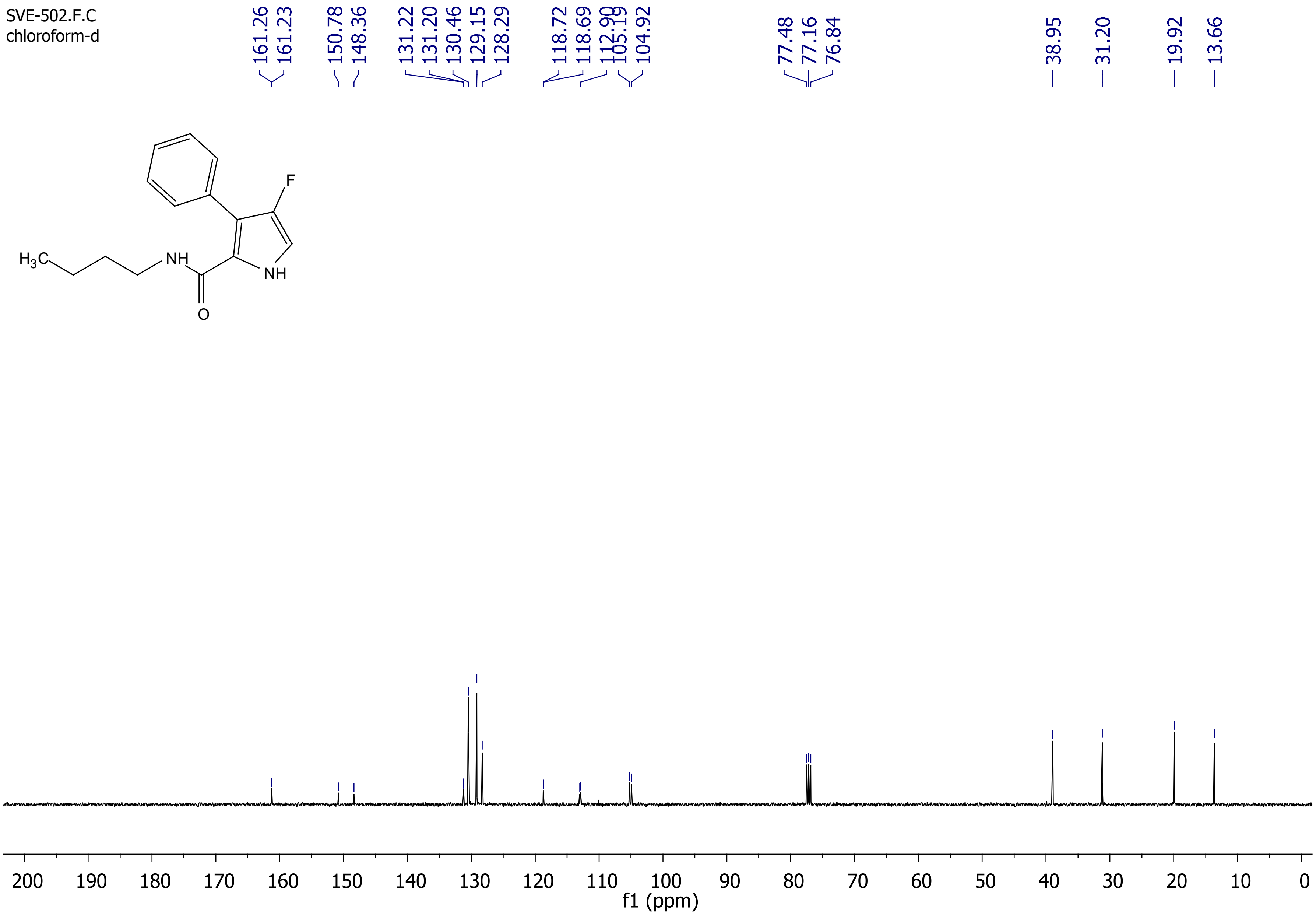

$^{13}C\{^{1}H\}$ NMR spectrum of *N*-butyl-4-fluoro-3-phenyl-1*H*-pyrrole-2-carboxamide (**2a**) in $CDCl_3$ at 100 MHz

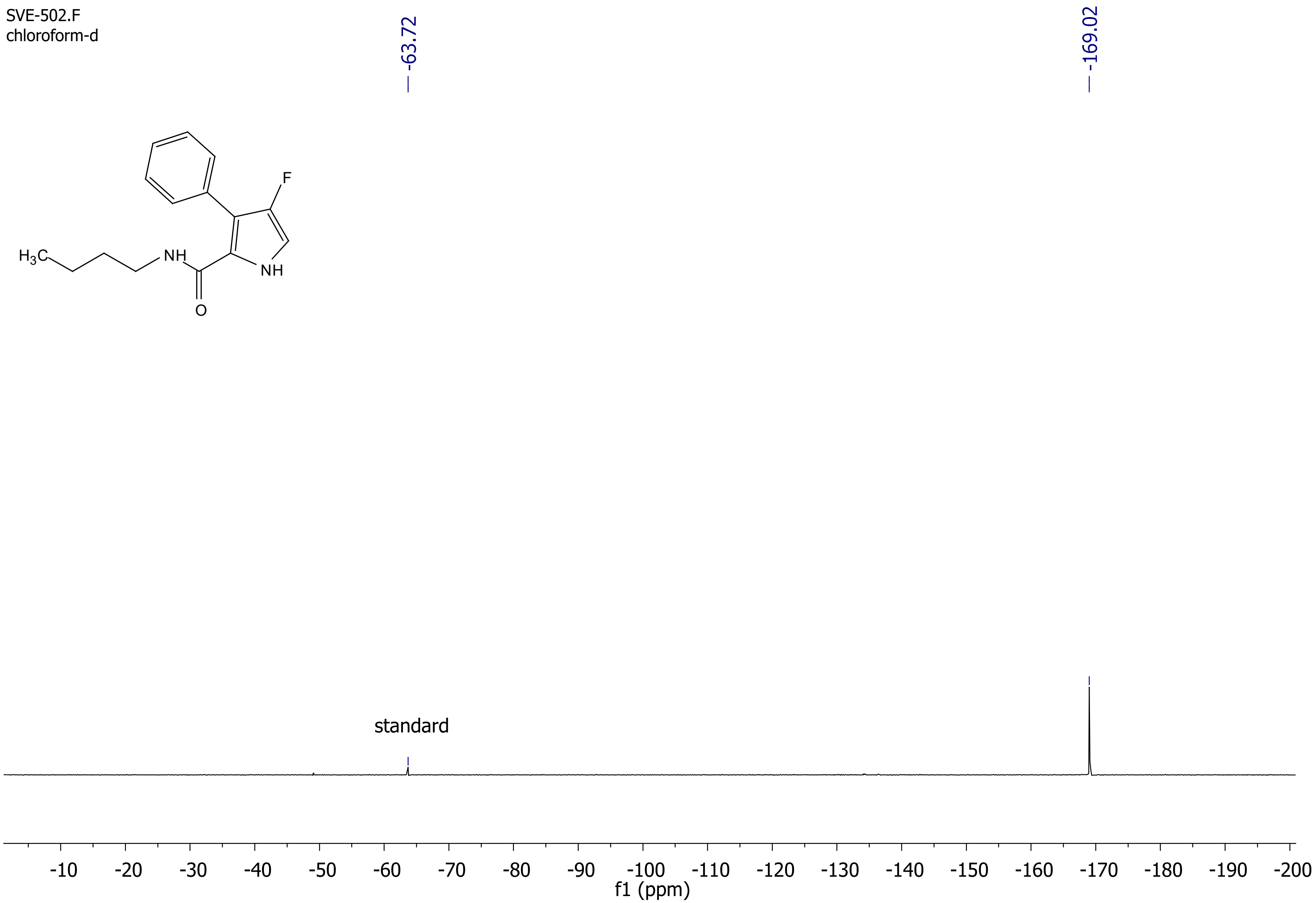

^{19}F NMR spectrum of *N*-butyl-4-fluoro-3-phenyl-1*H*-pyrrole-2-carboxamide (**2a**) in $CDCl_3$ at 376 MHz

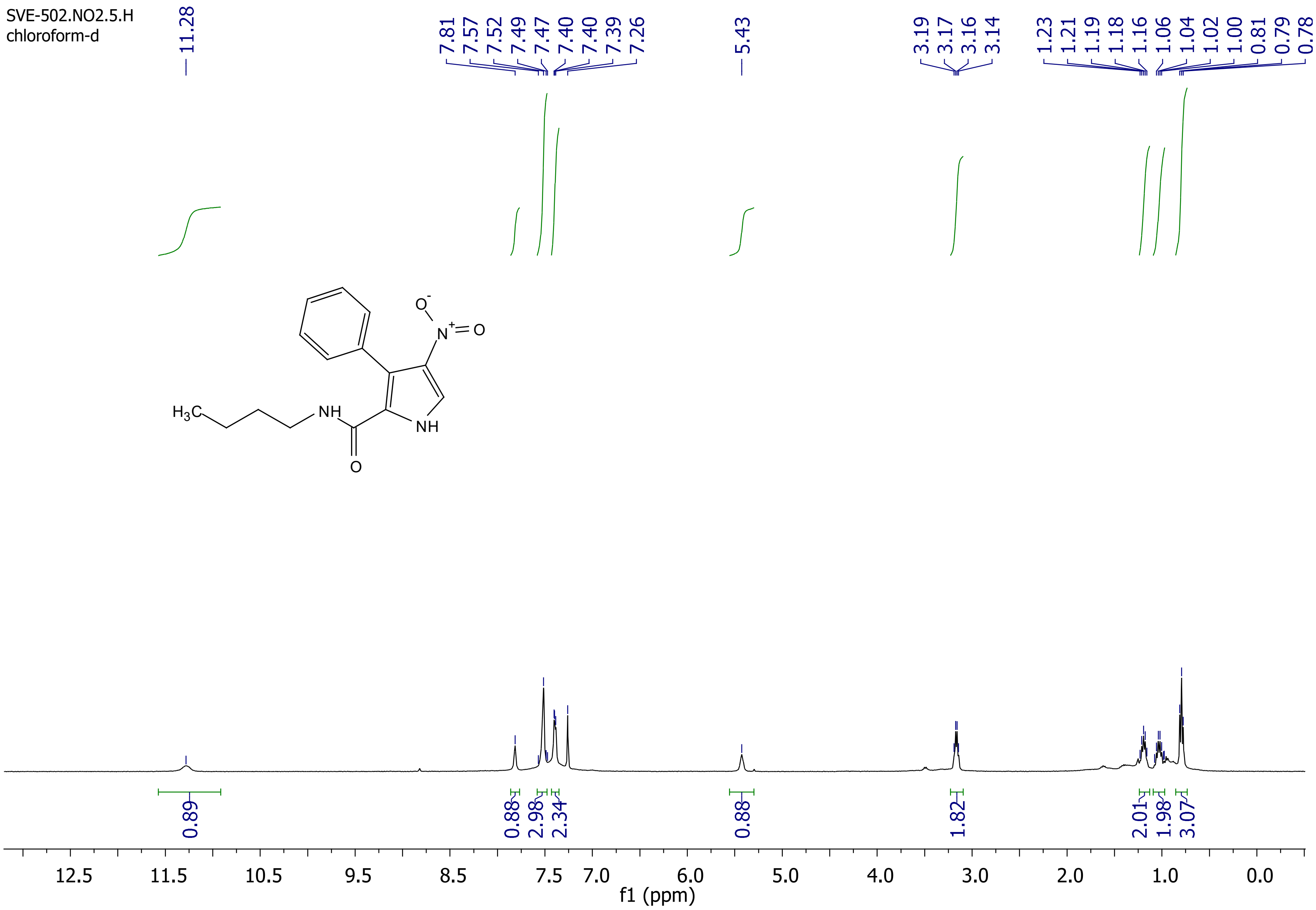

^{1}H NMR spectrum of *N*-butyl-4-nitro-3-phenyl-1*H*-pyrrole-2-carboxamide (**3a**) in $CDCl_3$ at 400 MHz

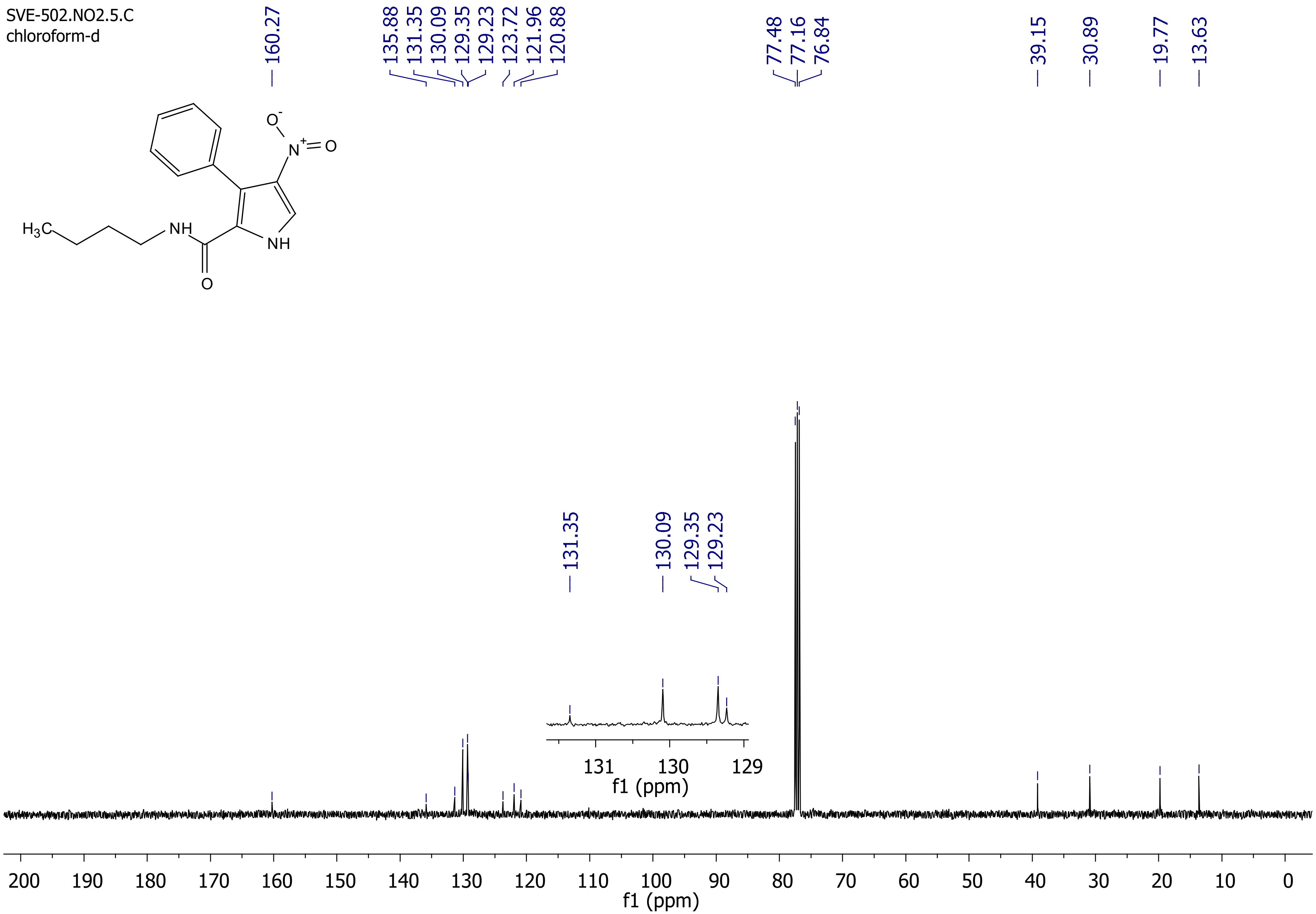

$^{13}C\{^1H\}$ NMR spectrum of *N*-butyl-4-nitro-3-phenyl-1*H*-pyrrole-2-carboxamide (**3a**) in $CDCl_3$ at 100 MHz

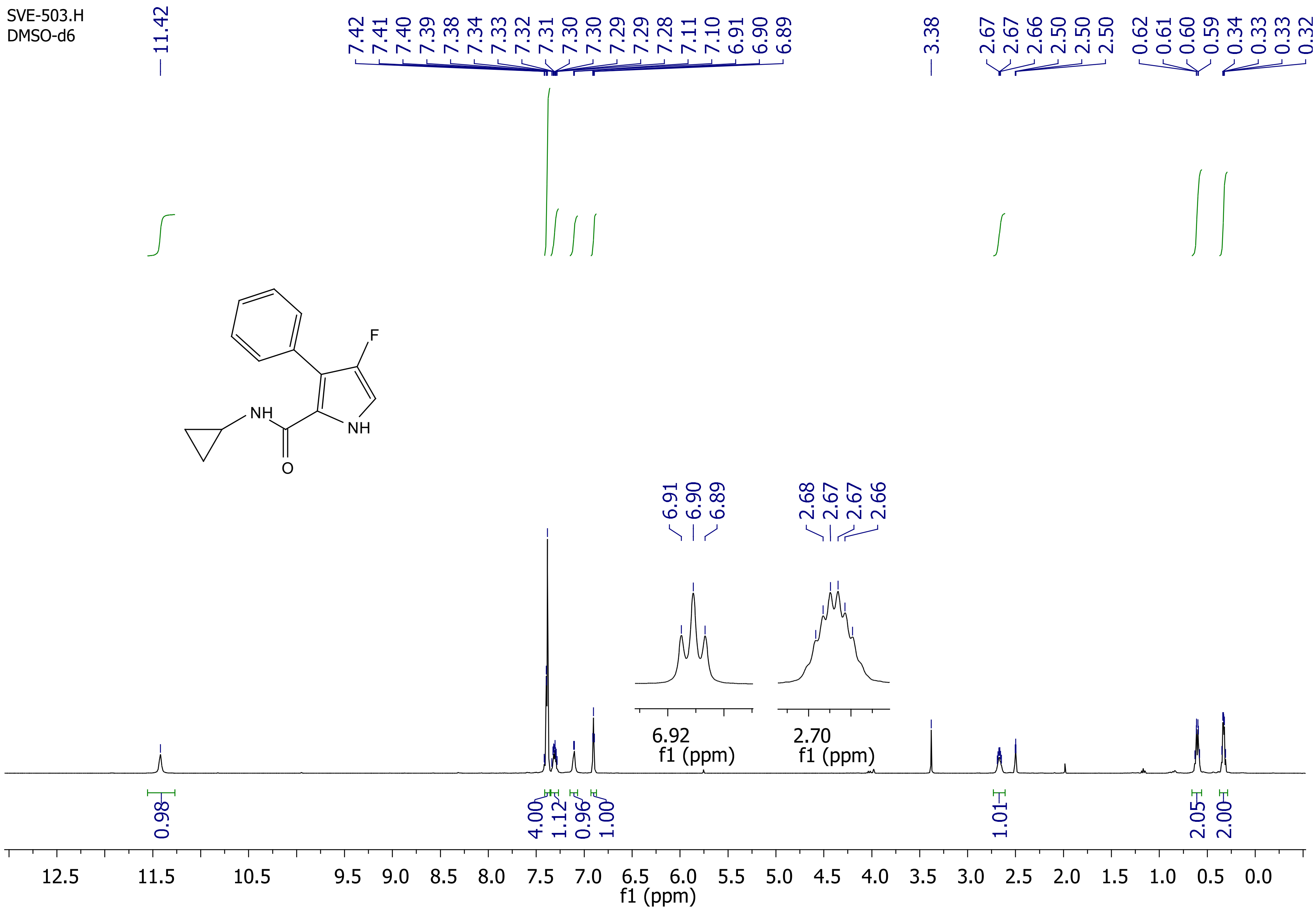

^{1}H NMR spectrum of *N*-cyclopropyl-4-fluoro-3-phenyl-1*H*-pyrrole-2-carboxamide (**2b**) in DMSO-d_6 at 400 MHz

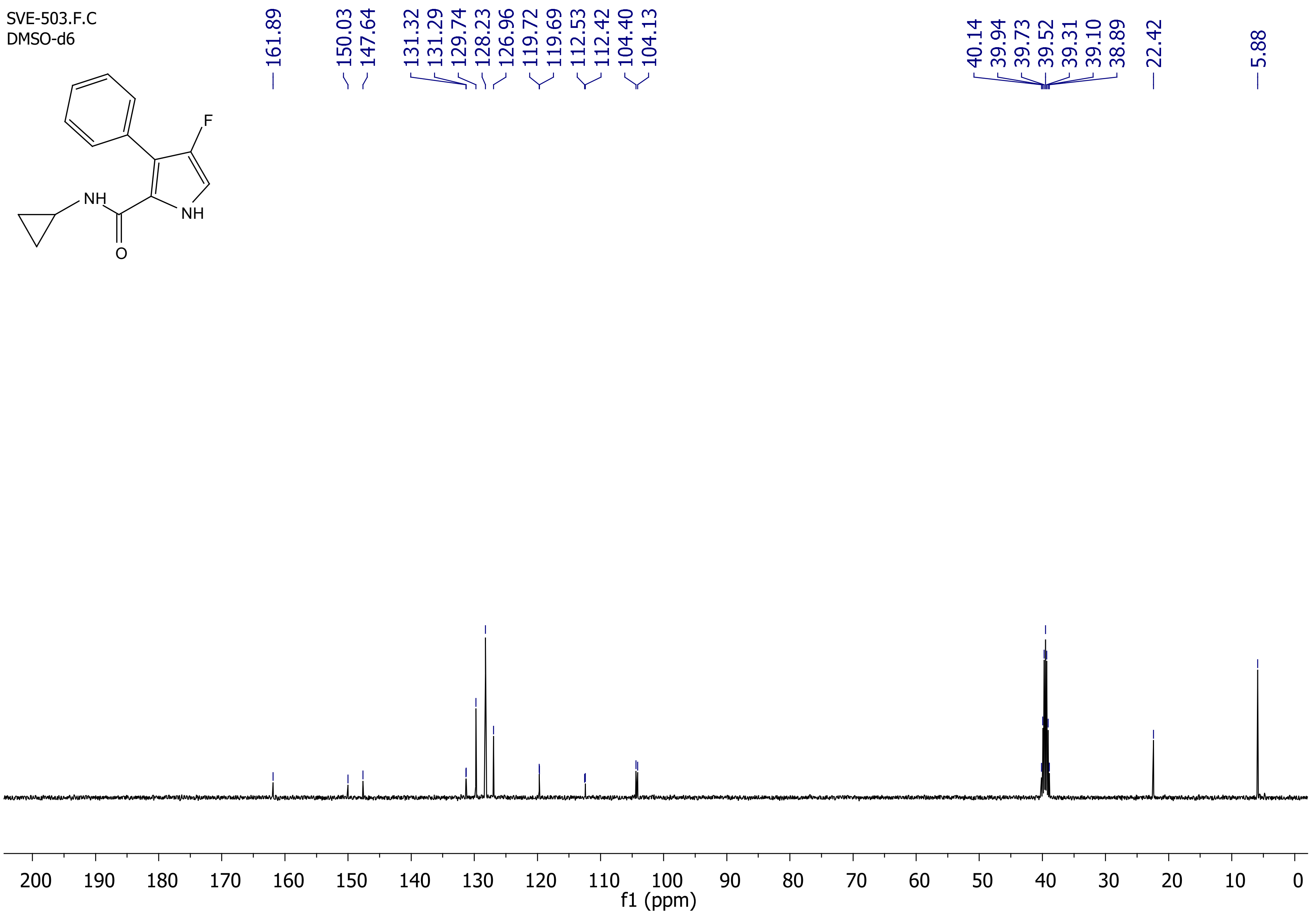

$^{13}C\{^{1}H\}$ NMR spectrum of *N*-cyclopropyl-4-fluoro-3-phenyl-1*H*-pyrrole-2-carboxamide (**2b**) in DMSO-d_6 at 100 MHz

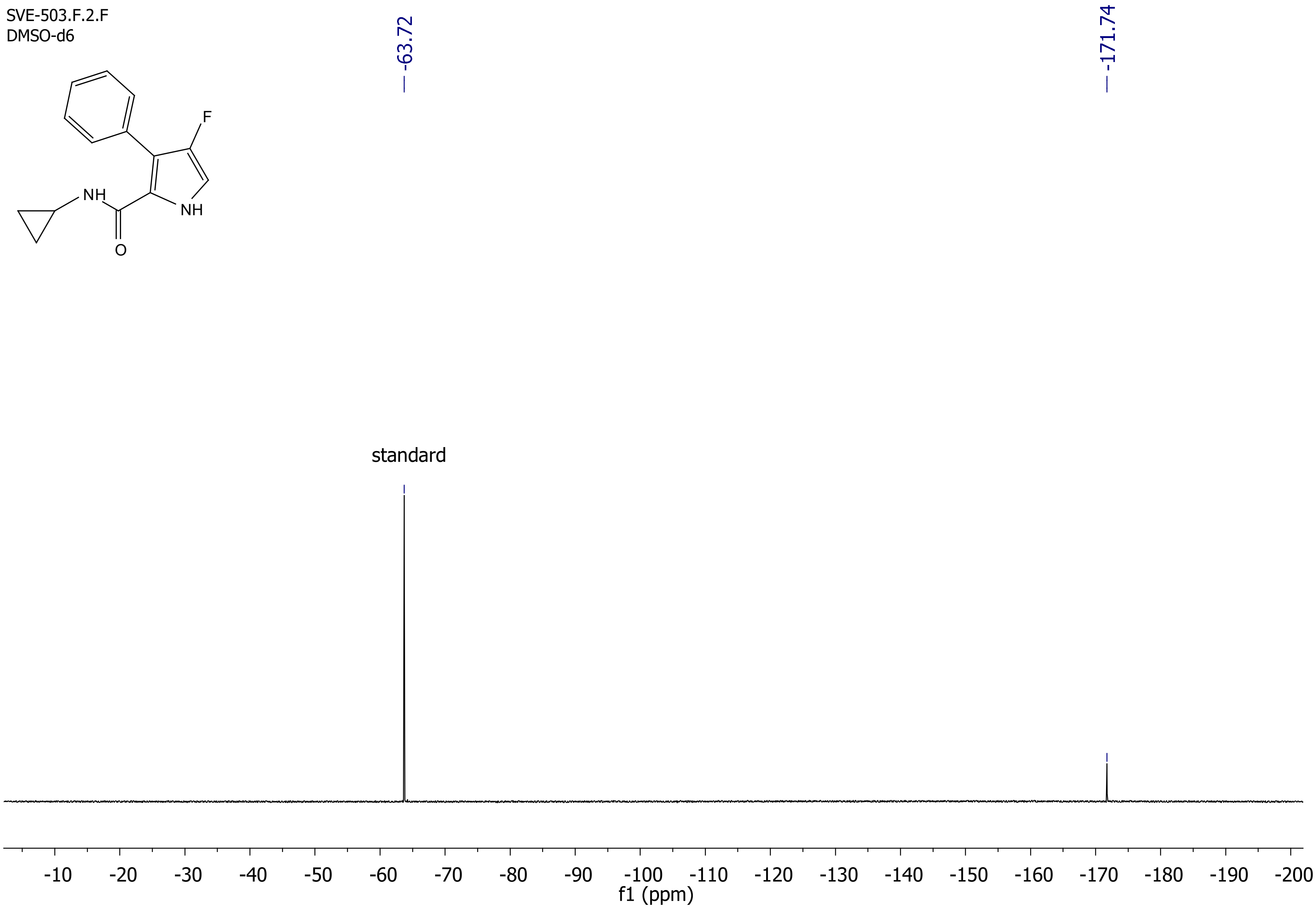

^{19}F NMR spectrum of *N*-cyclopropyl-4-fluoro-3-phenyl-1*H*-pyrrole-2-carboxamide (**2b**) in DMSO-d_6 at 376 MHz

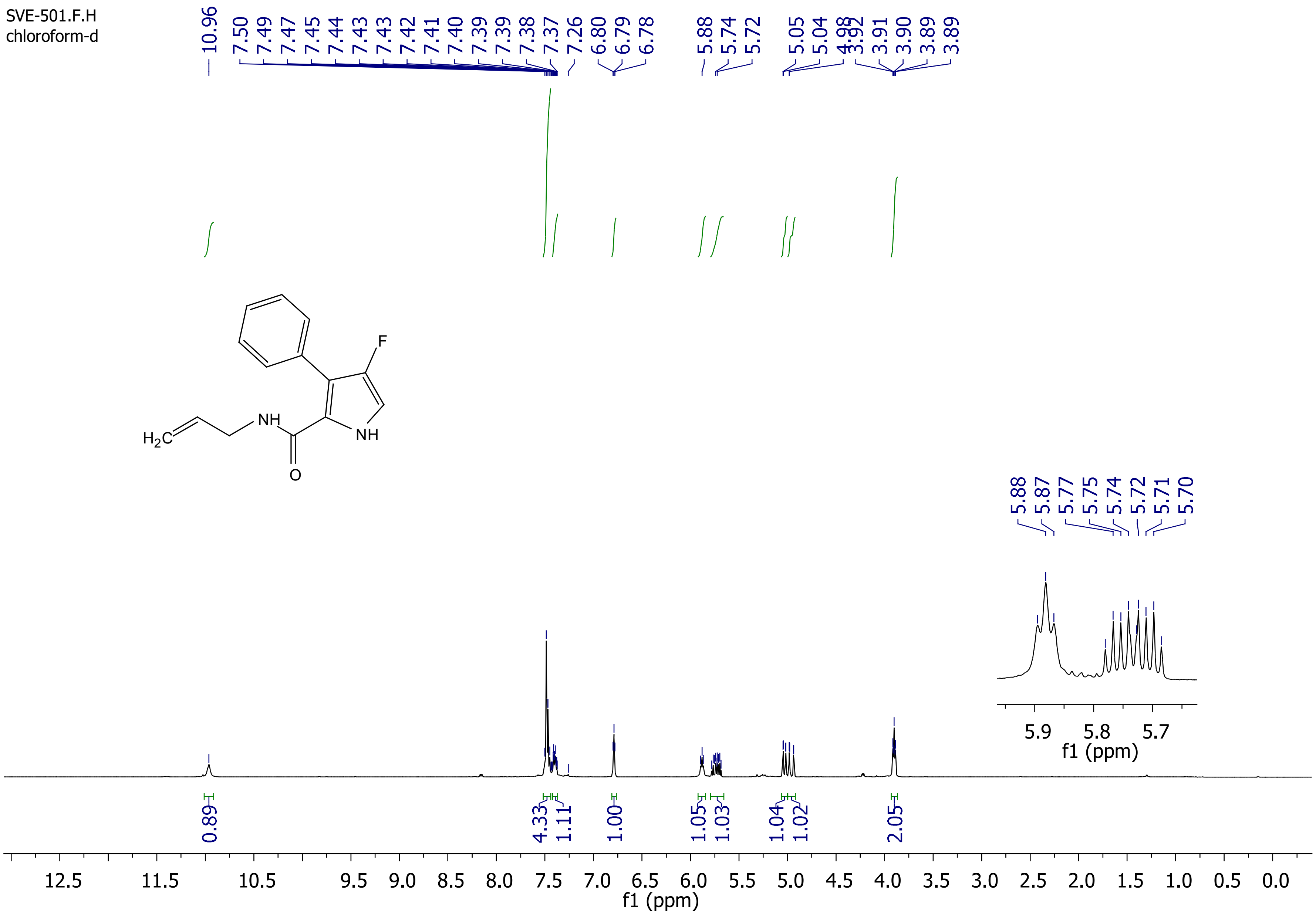

^{1}H NMR spectrum of *N*-allyl-4-fluoro-3-phenyl-1*H*-pyrrole-2-carboxamide (**2c**) in $CDCl_3$ at 400 MHz

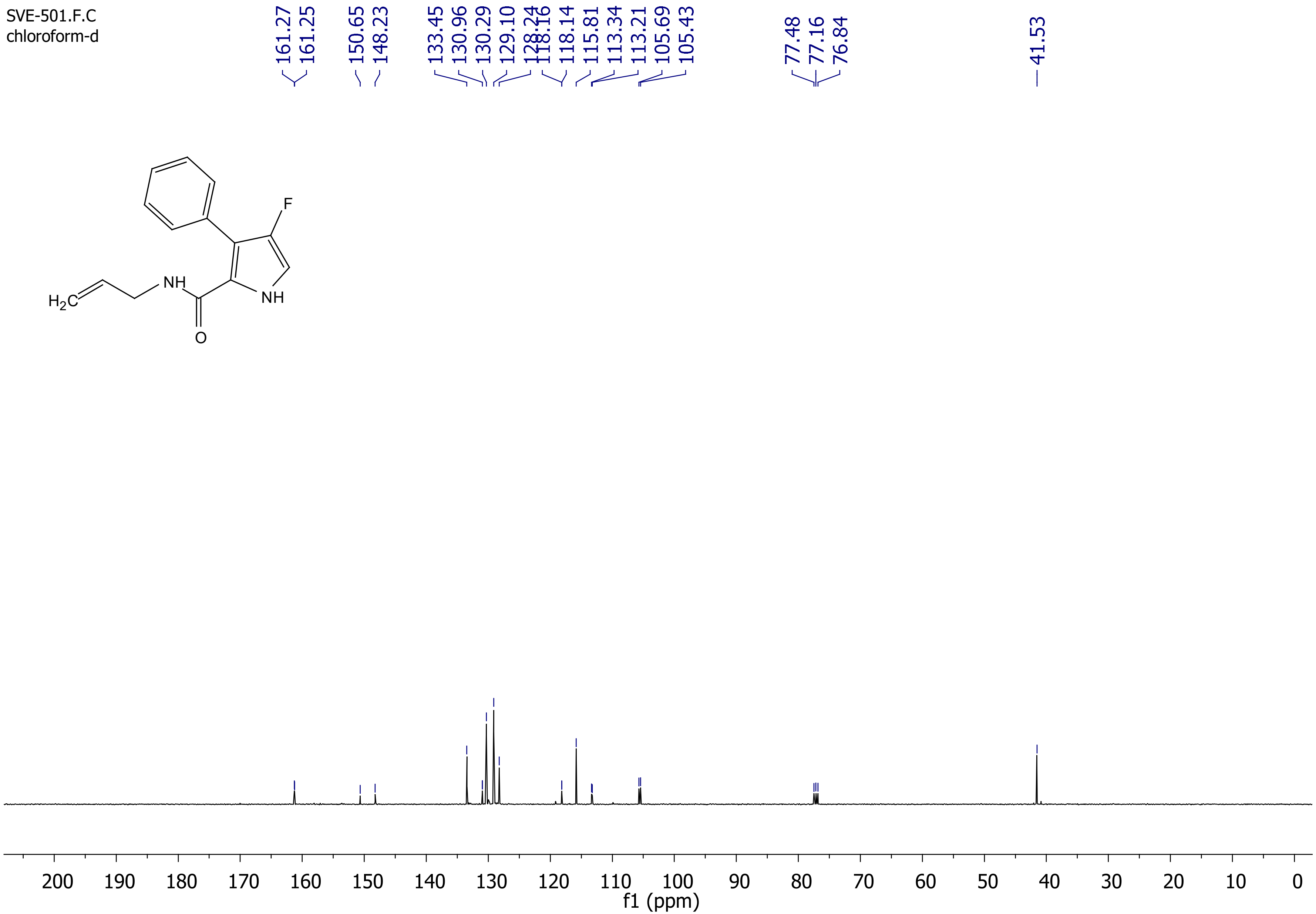

$^{13}C\{^{1}H\}$ NMR spectrum of *N*-allyl-4-fluoro-3-phenyl-1*H*-pyrrole-2-carboxamide (**2c**) in $CDCl_3$ at 100 MHz

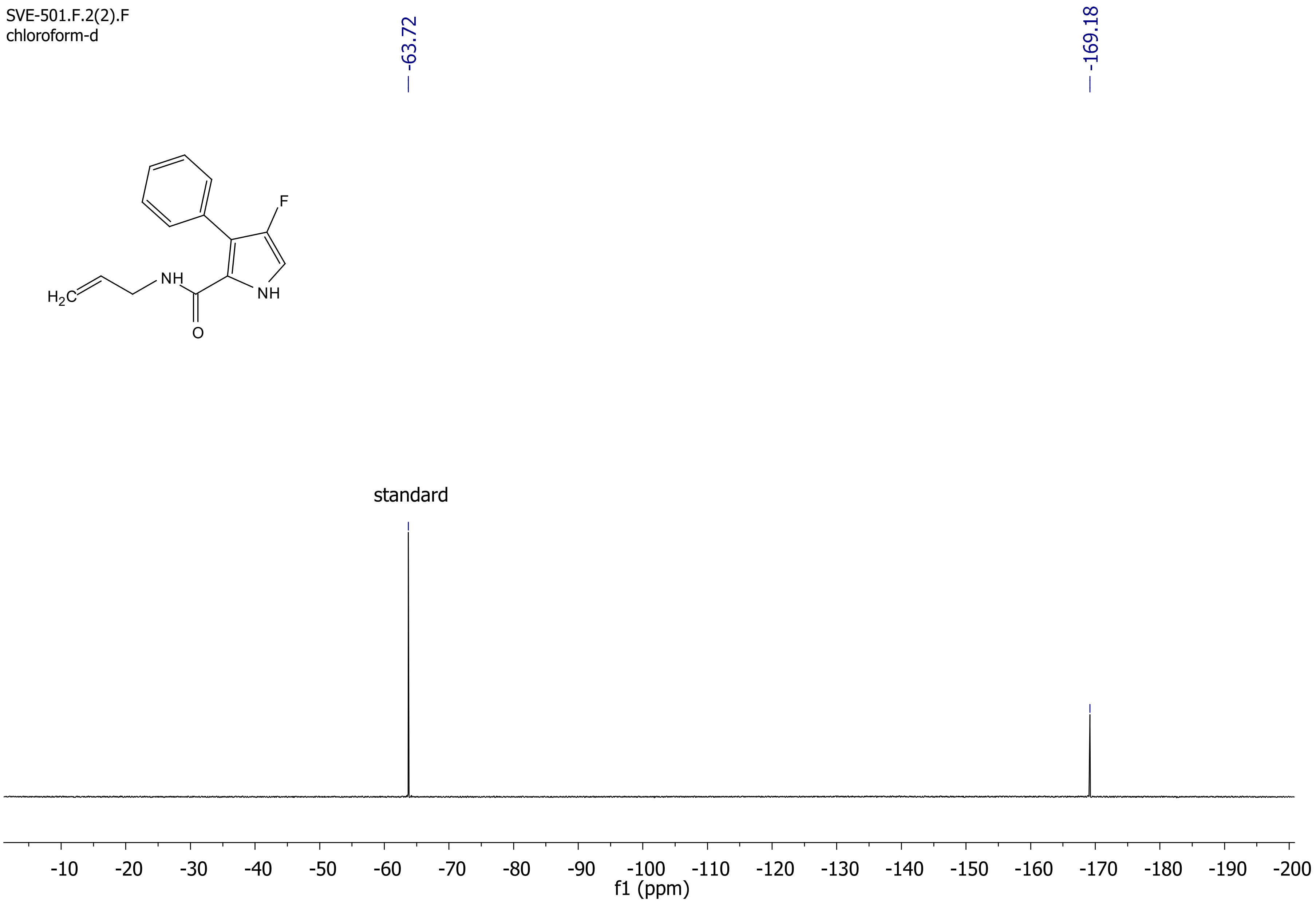

^{19}F NMR spectrum of *N*-allyl-4-fluoro-3-phenyl-1*H*-pyrrole-2-carboxamide (**2c**) in $CDCl_3$ at 376 MHz

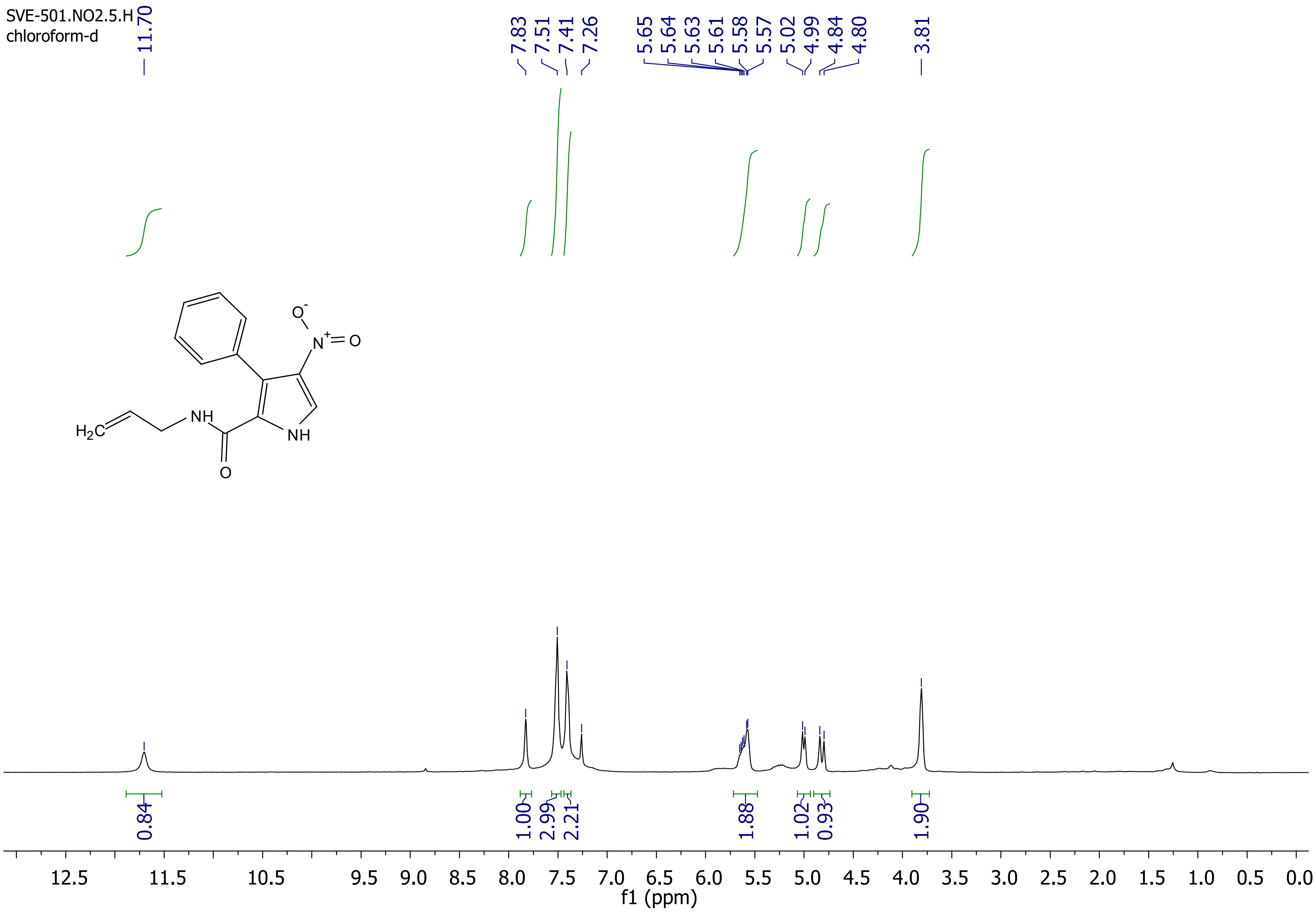

^{1}H NMR spectrum of *N*-allyl-4-nitro-3-phenyl-1*H*-pyrrole-2-carboxamide (**3c**) in $CDCl_3$ at 400 MHz

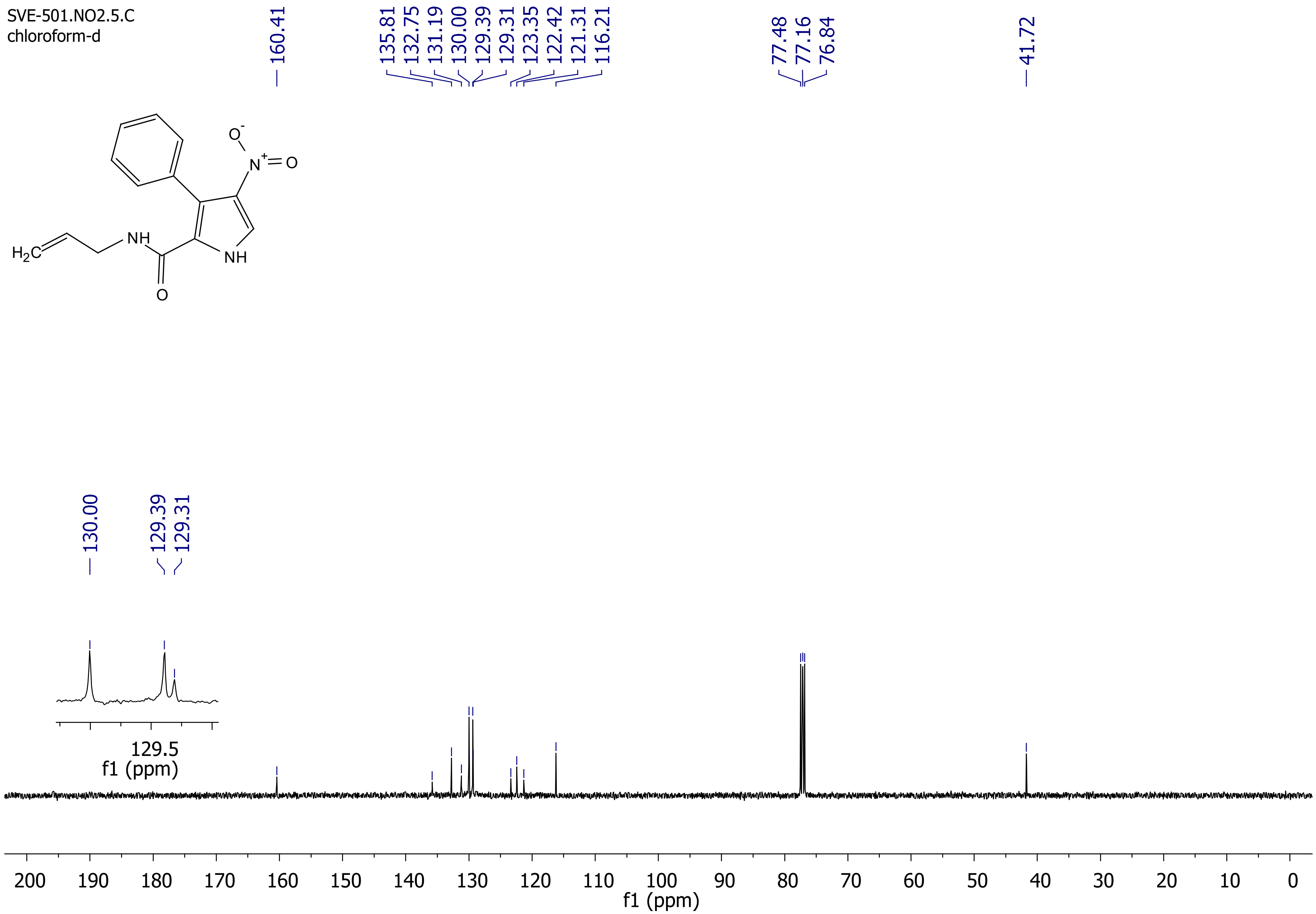

$^{13}C\{^{1}H\}$ NMR spectrum of *N*-allyl-4-nitro-3-phenyl-1*H*-pyrrole-2-carboxamide (**3c**) in $CDCl_3$ at 100 MHz

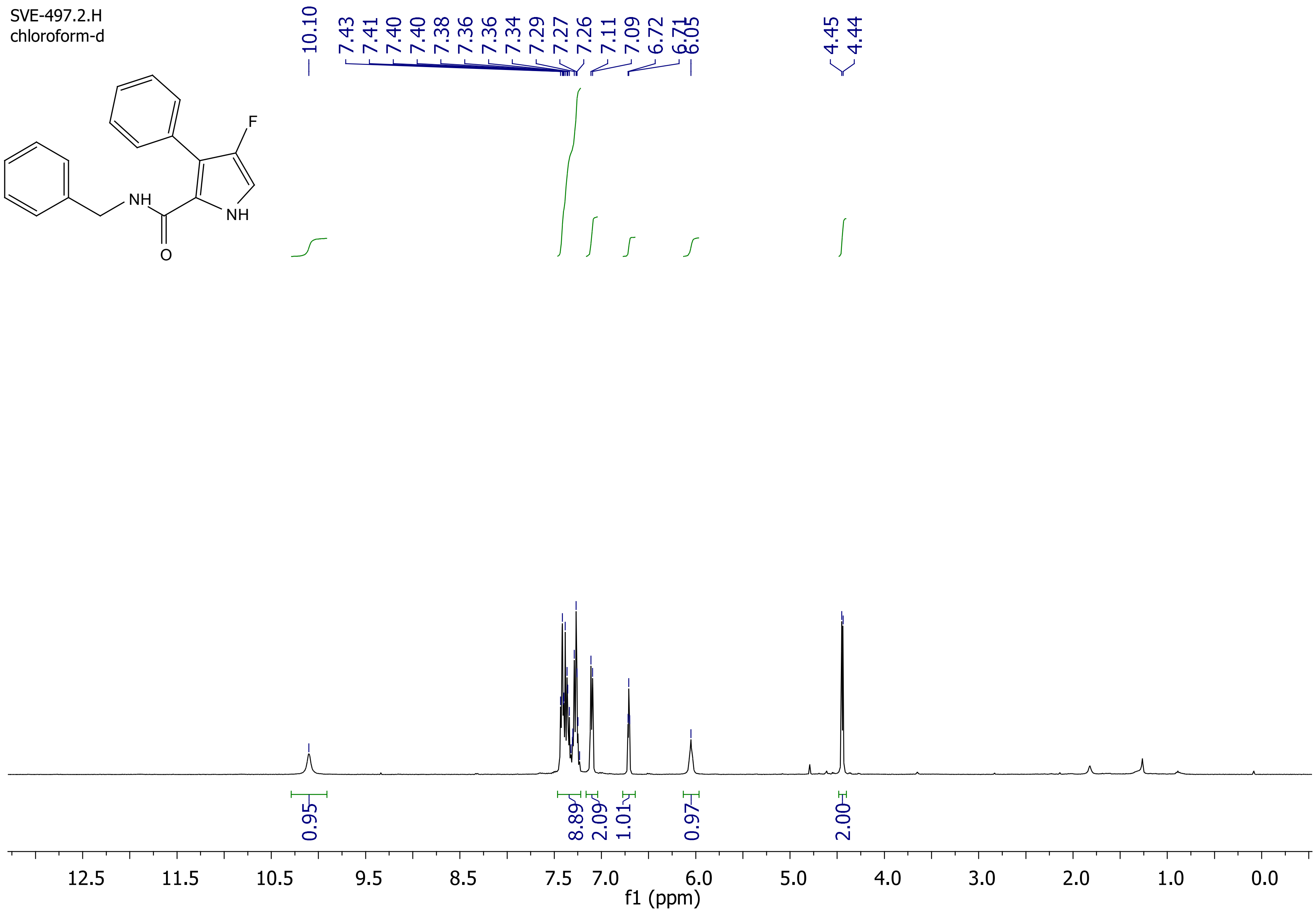

^{1}H NMR spectrum of *N*-benzyl-4-fluoro-3-phenyl-1*H*-pyrrole-2-carboxamide (**2d**) in $CDCl_3$ at 400 MHz

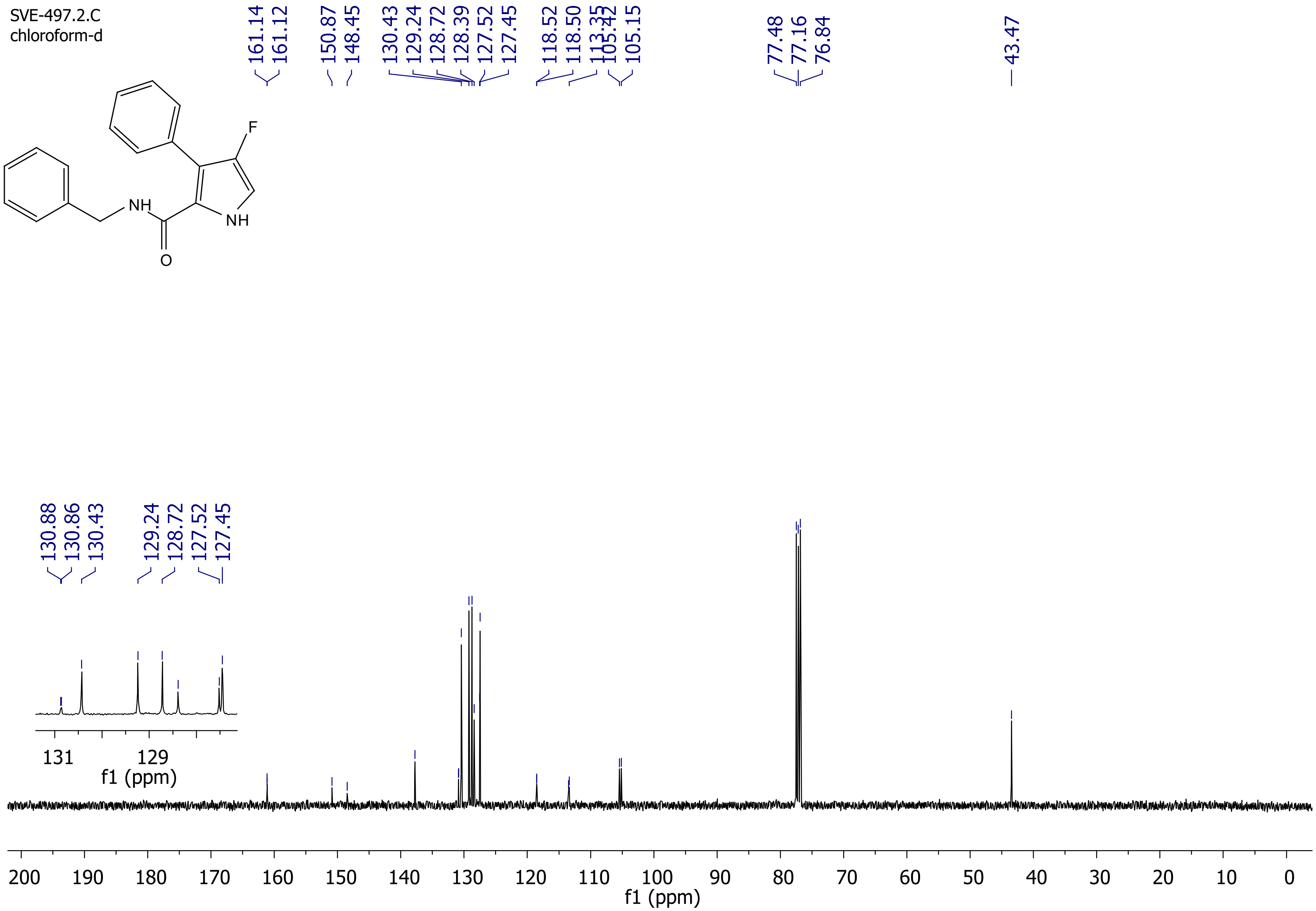

$^{13}C\{^{1}H\}$ NMR spectrum of *N*-benzyl-4-fluoro-3-phenyl-1*H*-pyrrole-2-carboxamide (**2d**) in $CDCl_3$ at 100 MHz

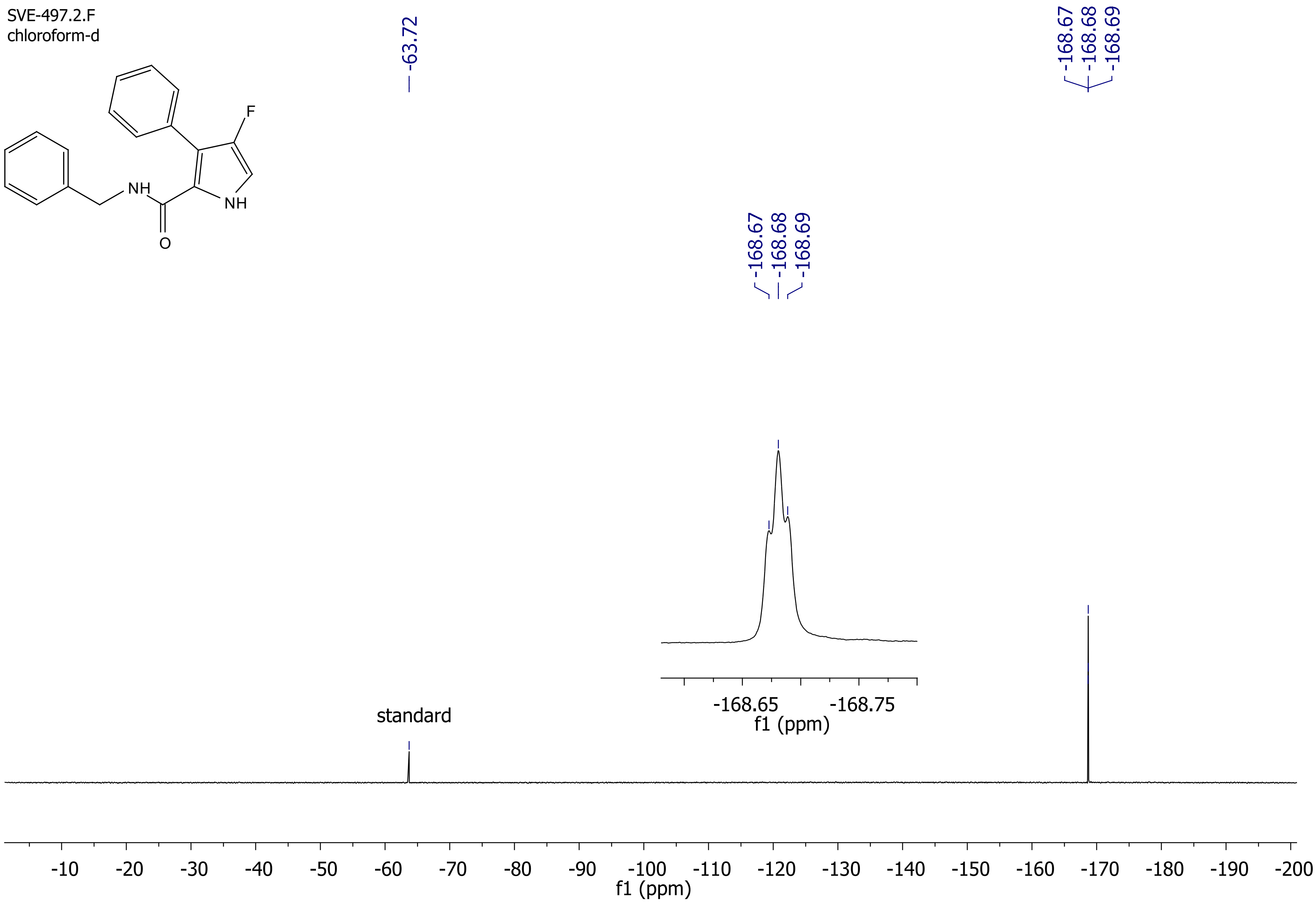

^{19}F NMR spectrum of *N*-benzyl-4-fluoro-3-phenyl-1*H*-pyrrole-2-carboxamide (**2d**) in $CDCl_3$ at 376 MHz

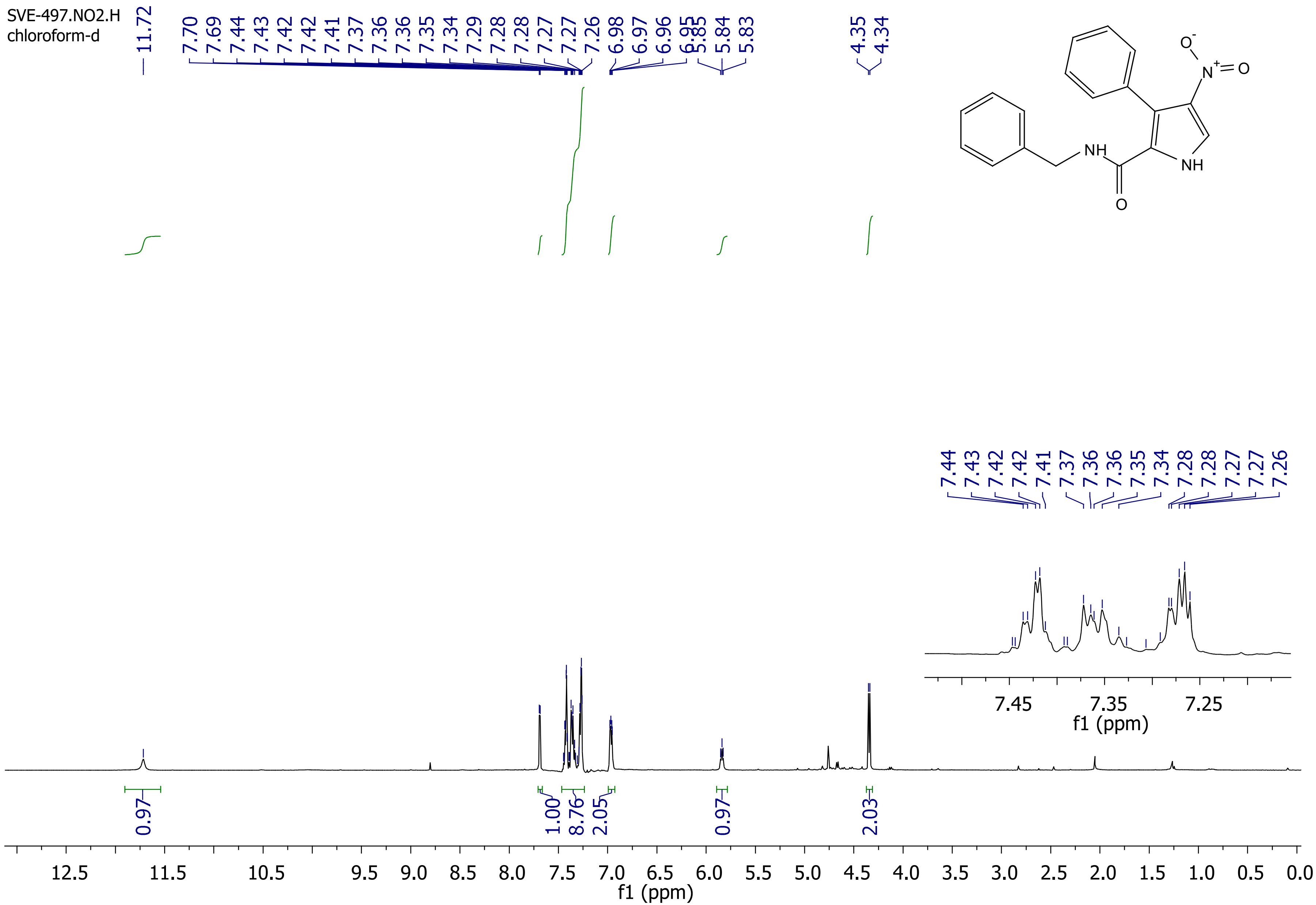

^{1}H NMR spectrum of *N*-benzyl-4-nitro-3-phenyl-1*H*-pyrrole-2-carboxamide (**3d**) in $CDCl_3$ at 400 MHz

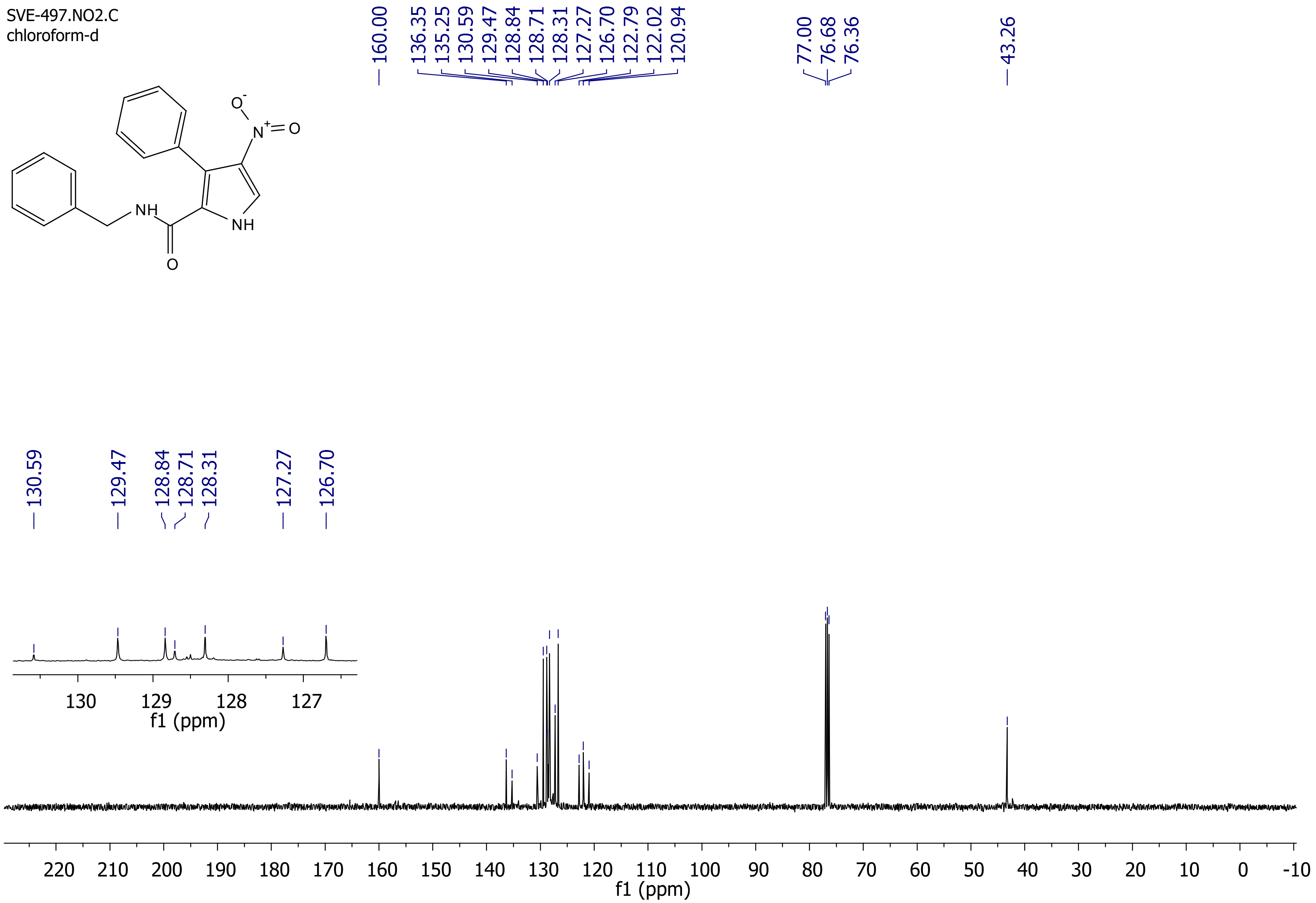

$^{13}C\{^1H\}$ NMR spectrum of *N*-benzyl-4-nitro-3-phenyl-1*H*-pyrrole-2-carboxamide (**3d**) in $CDCl_3$ at 100 MHz

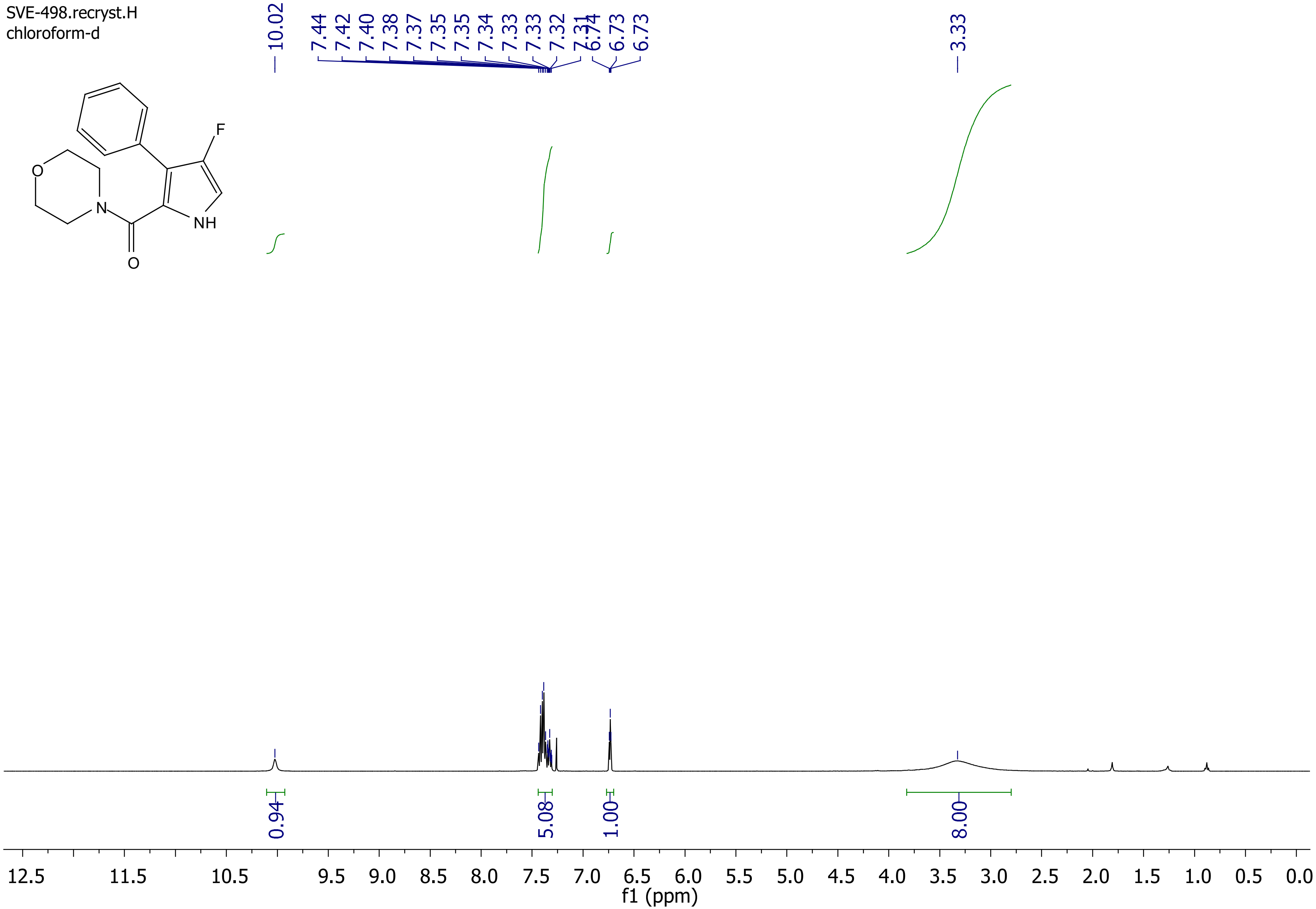

^{1}H NMR spectrum of (4-fluoro-3-phenyl-1*H*-pyrrol-2-yl)(morpholino)methanone (**2e**) in $CDCl_3$ at 400 MHz

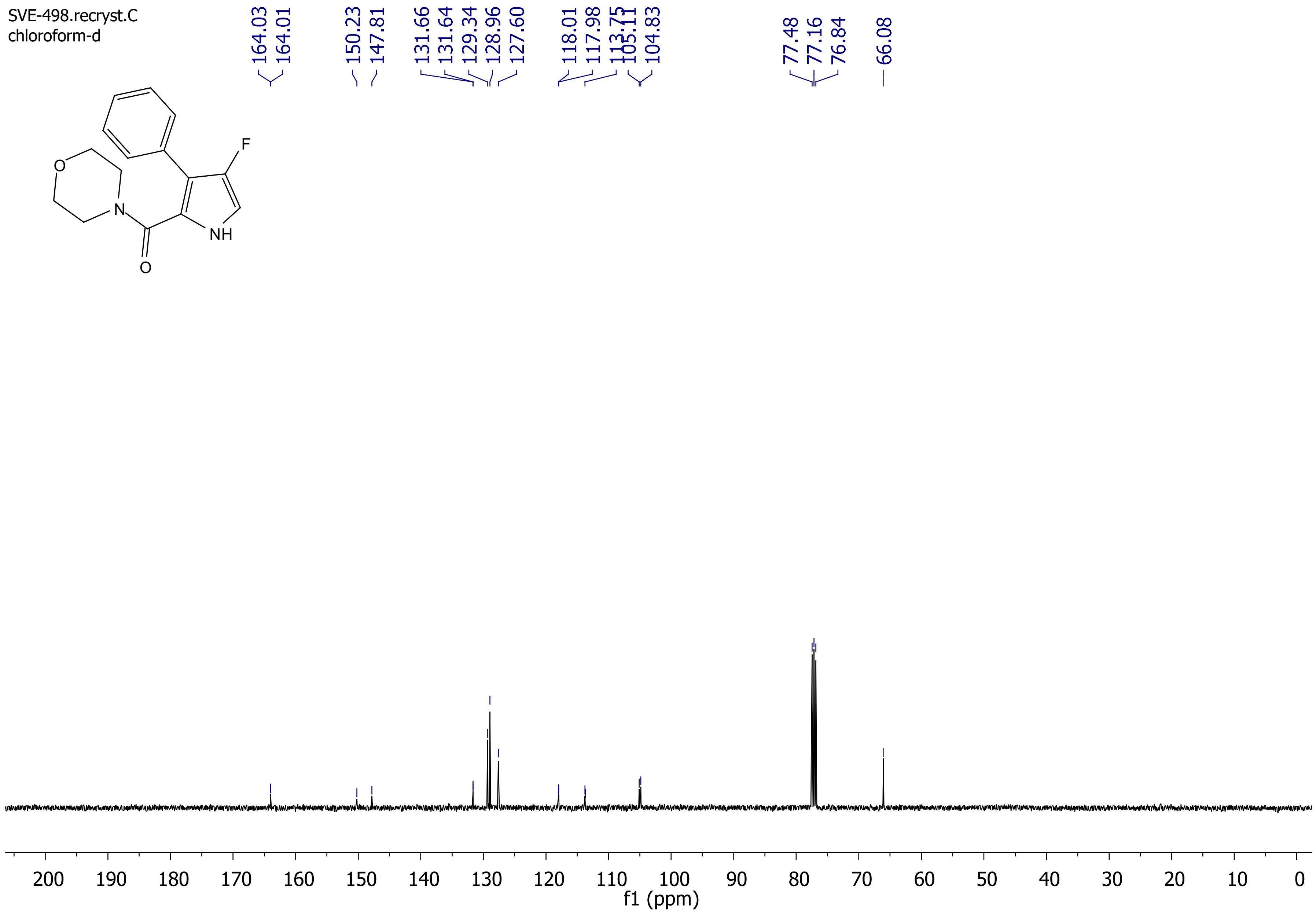

$^{13}C\{^1H\}$ NMR spectrum of (4-fluoro-3-phenyl-1*H*-pyrrol-2-yl)(morpholino)methanone (**2e**) in $CDCl_3$ at 100 MHz

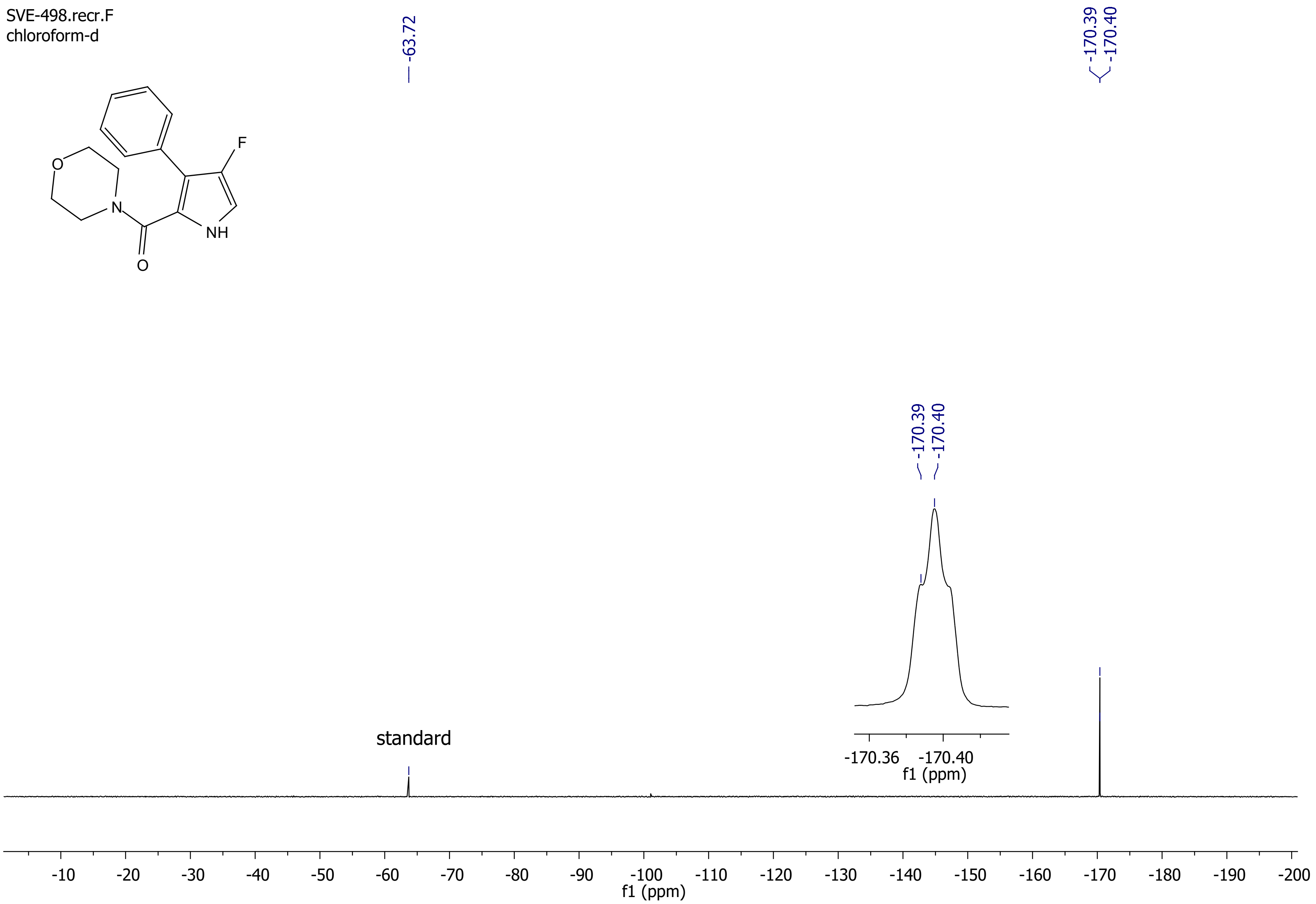

^{19}F NMR spectrum of (4-fluoro-3-phenyl-1*H*-pyrrol-2-yl)(morpholino)methanone (**2e**) in $CDCl_3$ at 376 MHz

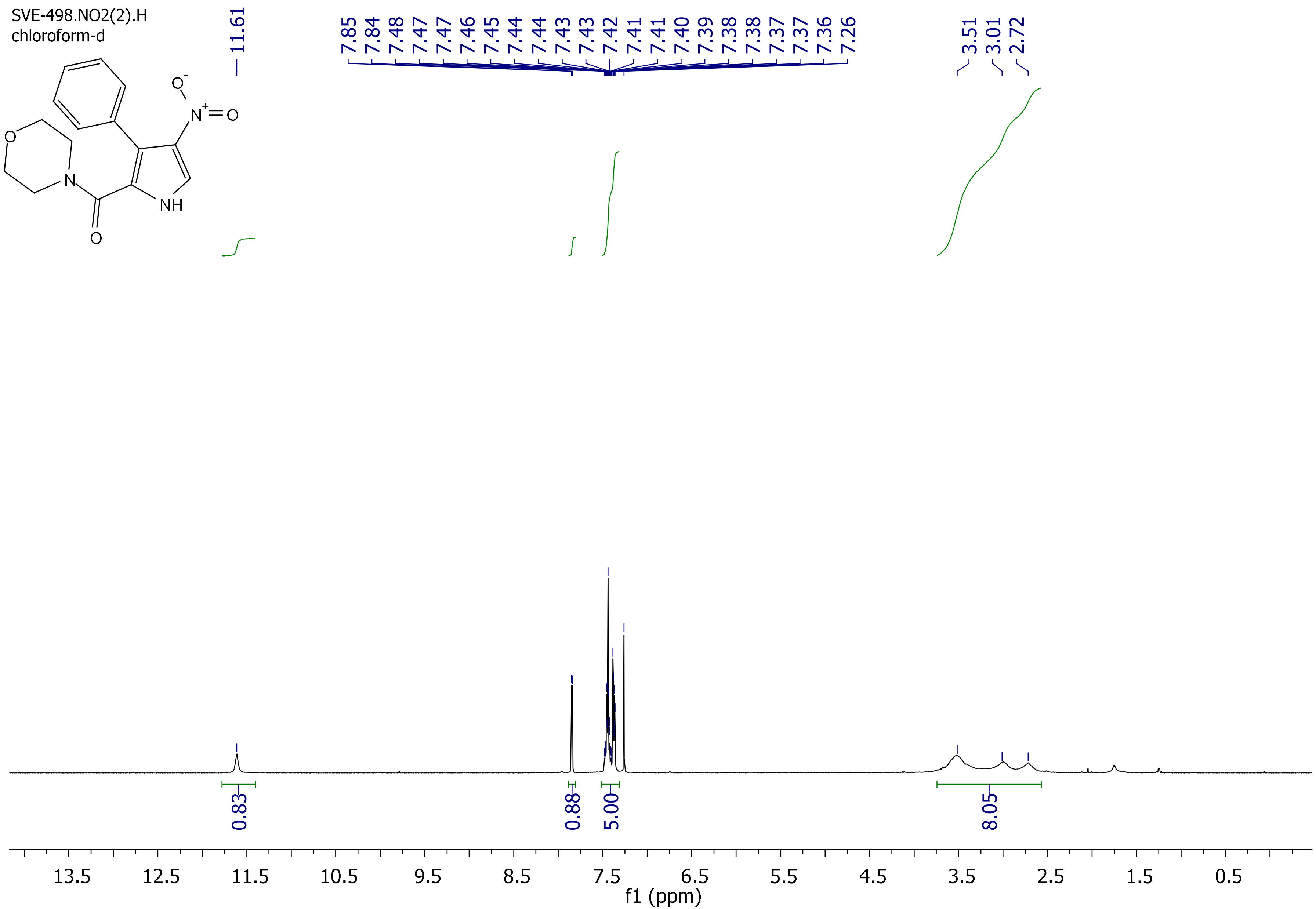

^{1}H NMR spectrum of morpholino(4-nitro-3-phenyl-1*H*-pyrrol-2-yl)methanone (**3e**) in $CDCl_3$ at 400 MHz

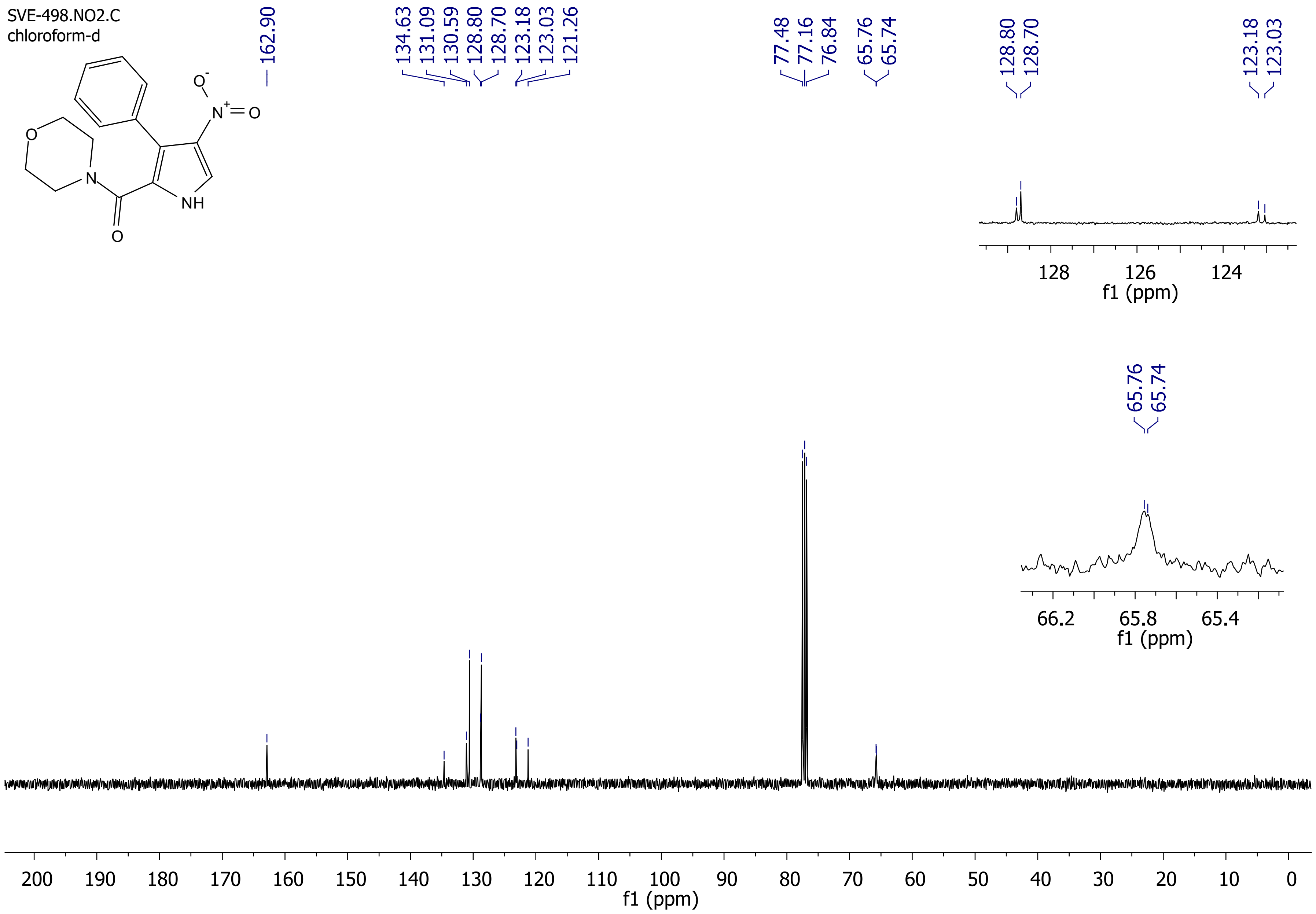

$^{13}C\{^1H\}$ NMR spectrum of morpholino(4-nitro-3-phenyl-1*H*-pyrrol-2-yl)methanone (**3e**) in $CDCl_3$ at 100 MHz

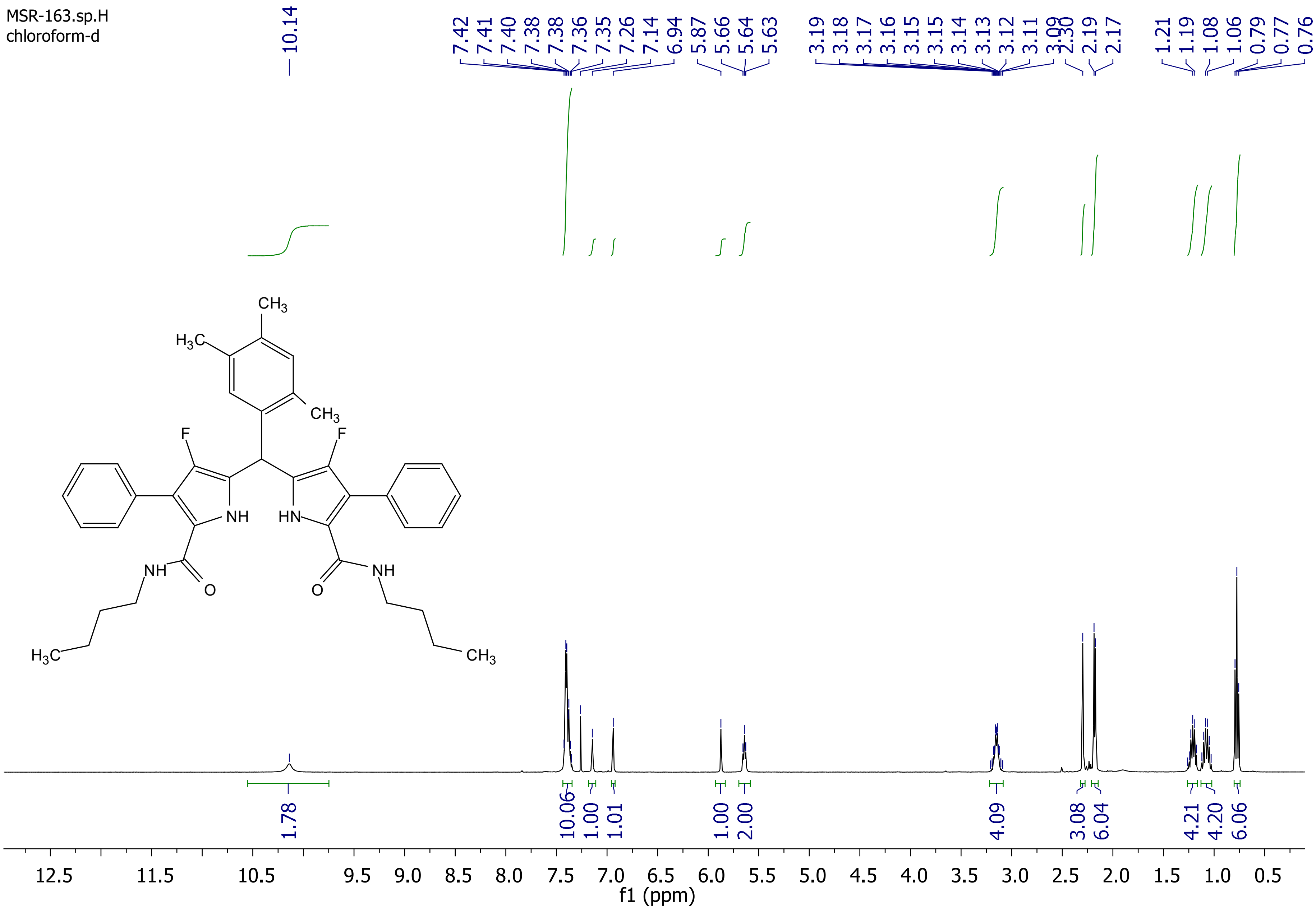

^{1}H NMR spectrum of 5,5'-((2,4,5-trimethylphenyl)methylene)bis(*N*-butyl-4-fluoro-3-phenyl-1*H*-pyrrole-2-carboxamide) (**4a**) in $CDCl_3$ at 400 MHz

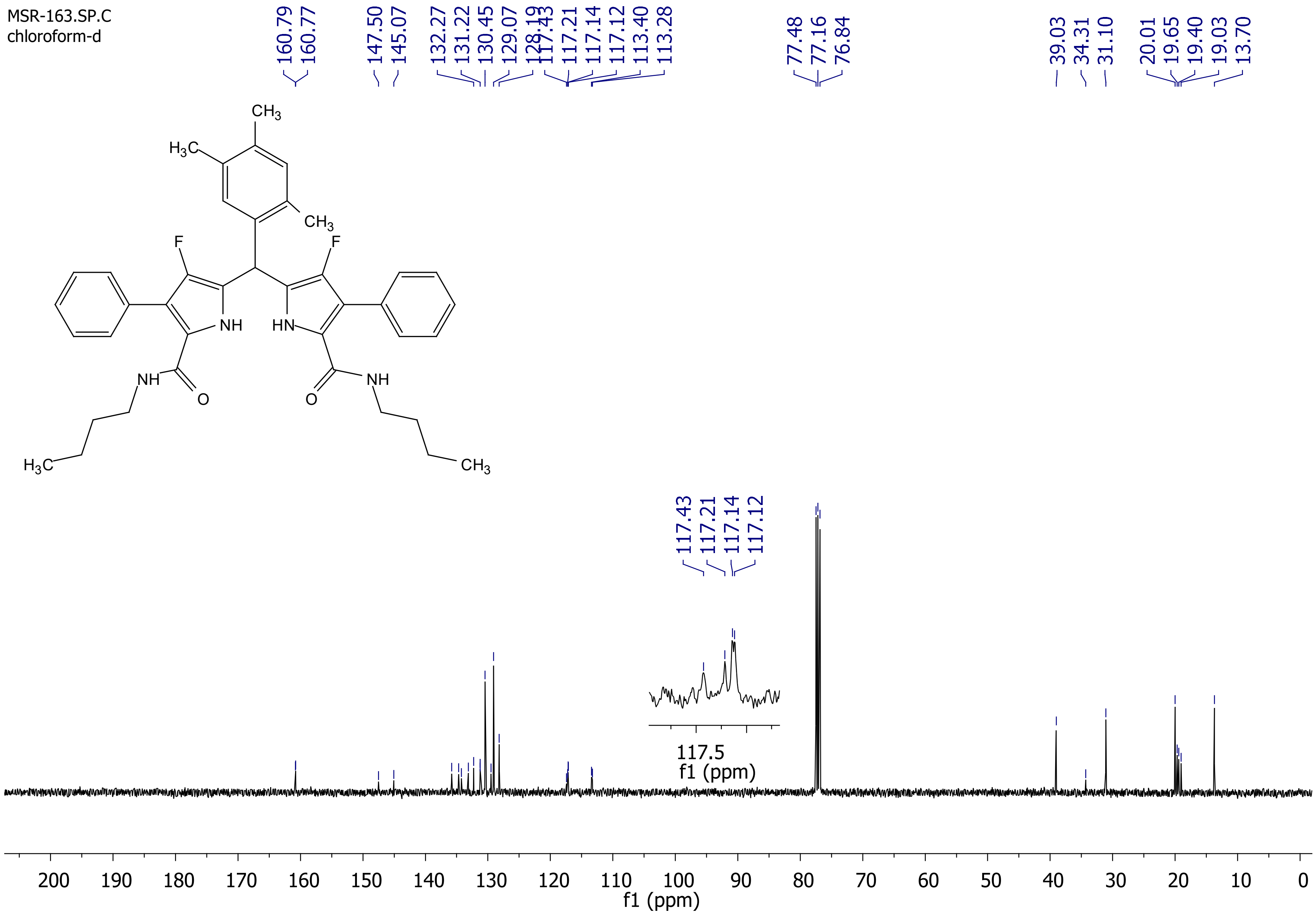

$^{13}C\{^{1}H\}$ NMR spectrum of 5,5'-((2,4,5-trimethylphenyl)methylene)bis(*N*-butyl-4-fluoro-3-phenyl-1*H*-pyrrole-2-carboxamide) (**4a**) in $CDCl_3$ at 100 MHz

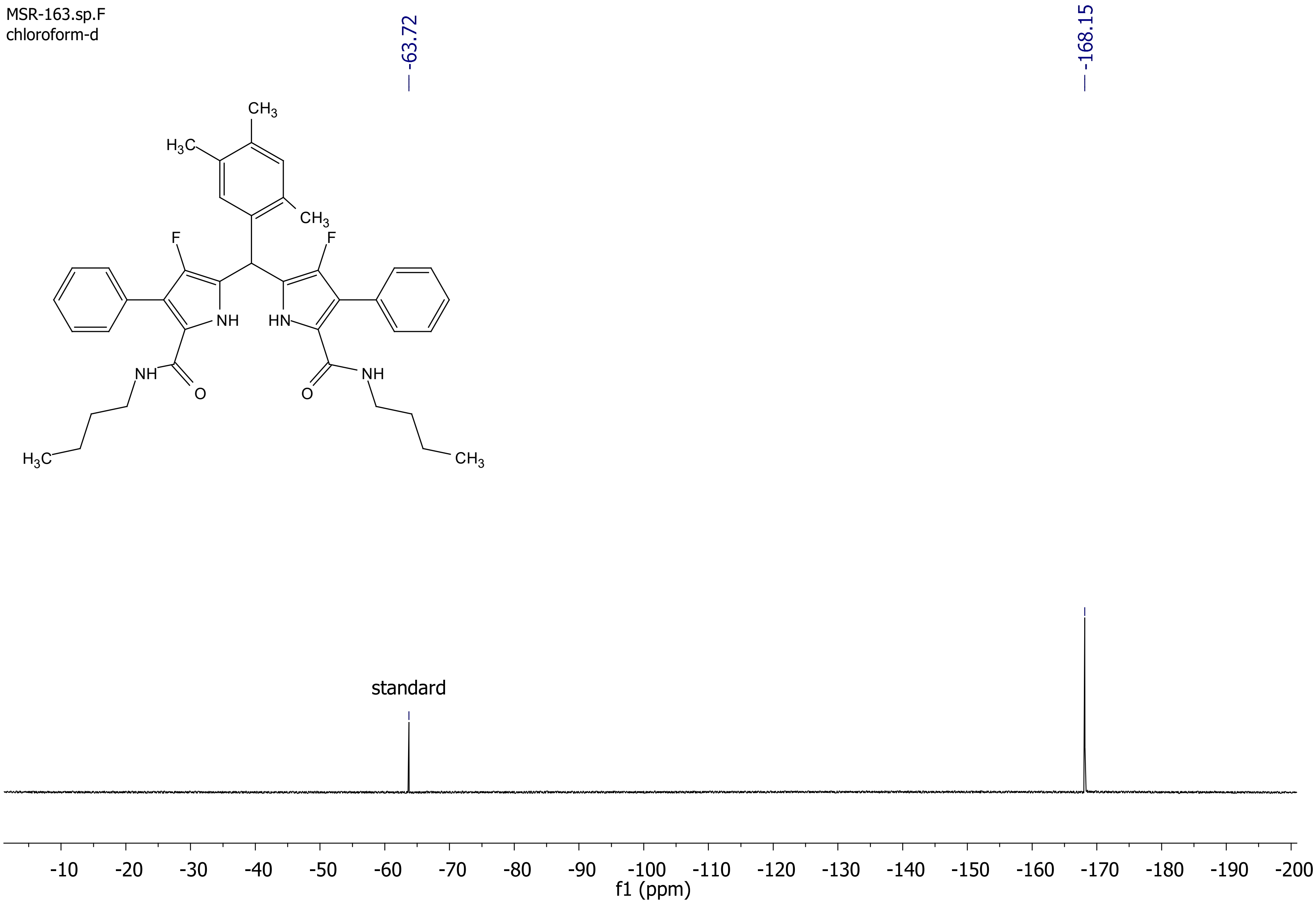

^{19}F NMR spectrum of 5,5'-((2,4,5-trimethylphenyl)methylene)bis(*N*-butyl-4-fluoro-3-phenyl-1*H*-pyrrole-2-carboxamide) (**4a**) in $CDCl_3$ at 376 MHz

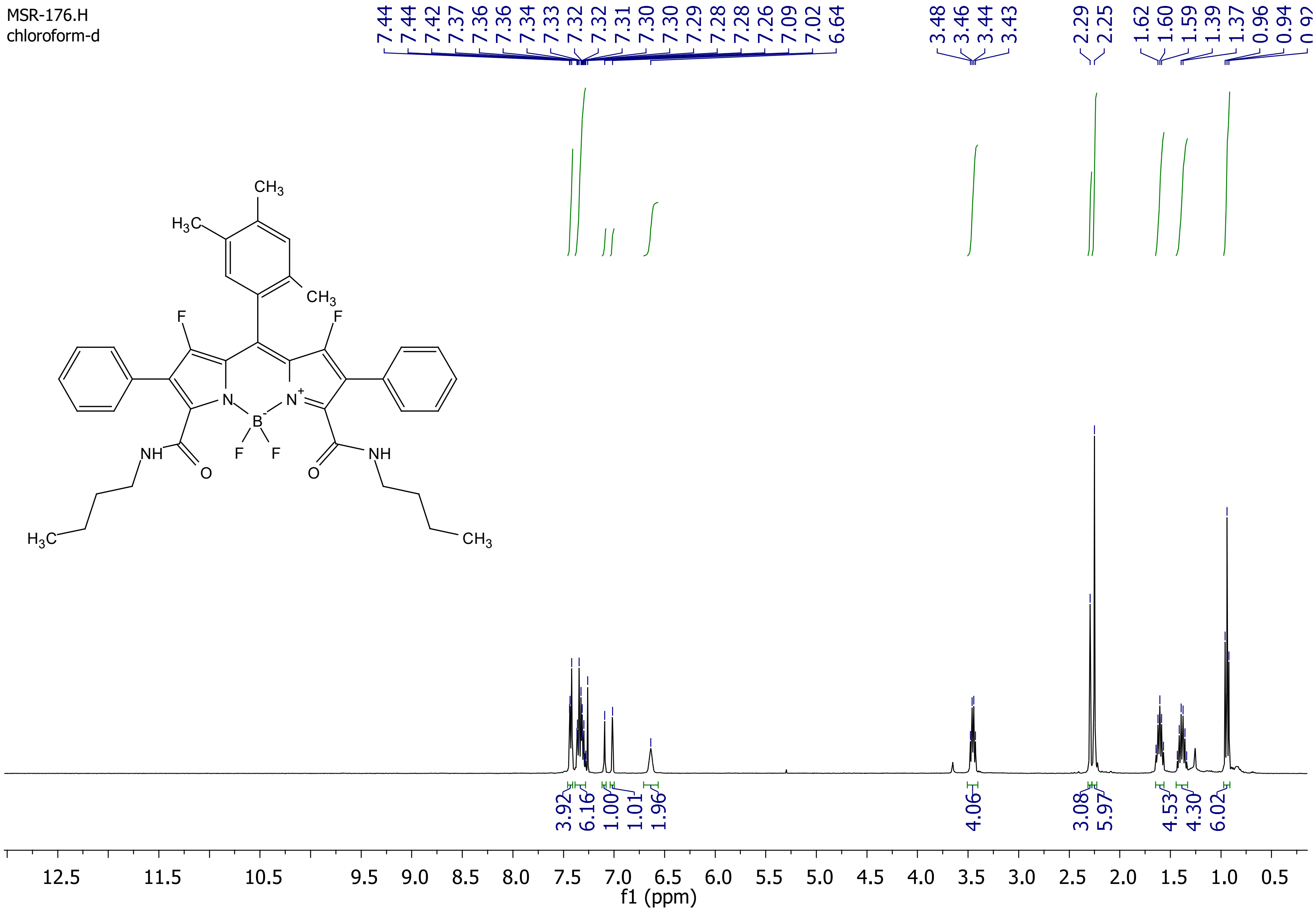

^{1}H NMR spectrum of 3,7-bis(butylcarbamoyl)-1,5,5,9-tetrafluoro-2,8-diphenyl-10-(2,4,5-trimethylphenyl)-5*H*-dipyrrolo[1,2-c:2',1'-f][1,3,2]diazaborinin-4-ium-5-uide (**5a**) in $CDCl_3$ at 400 MHz

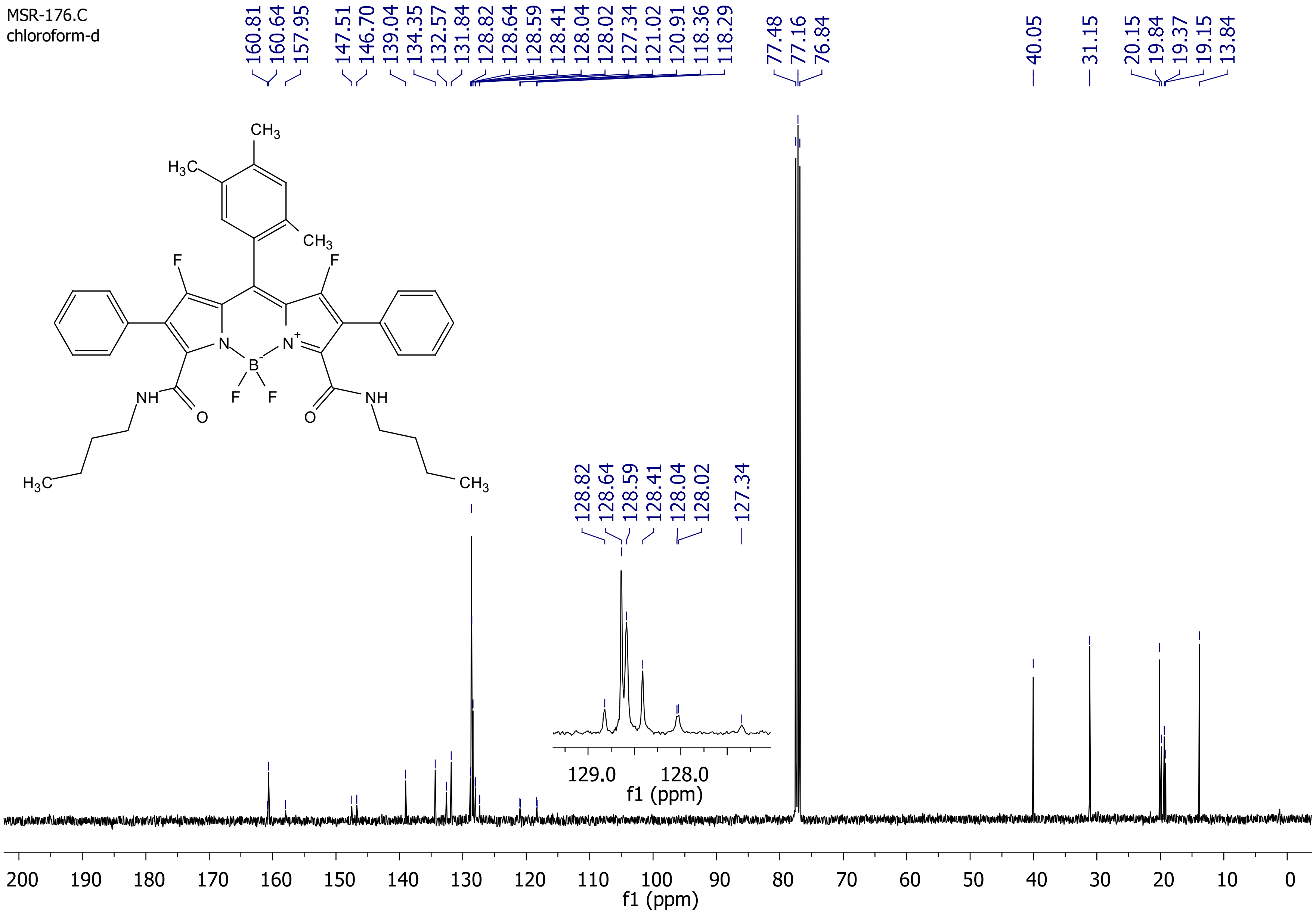

$^{13}C\{^{1}H\}$ NMR spectrum of 3,7-bis(butylcarbamoyl)-1,5,5,9-tetrafluoro-2,8-diphenyl-10-(2,4,5-trimethylphenyl)-5*H*-dipyrrolo[1,2-c:2',1'-f][1,3,2]diazaborinin-4-ium-5-uide (**5a**) in $CDCl_3$ at 100 MHz

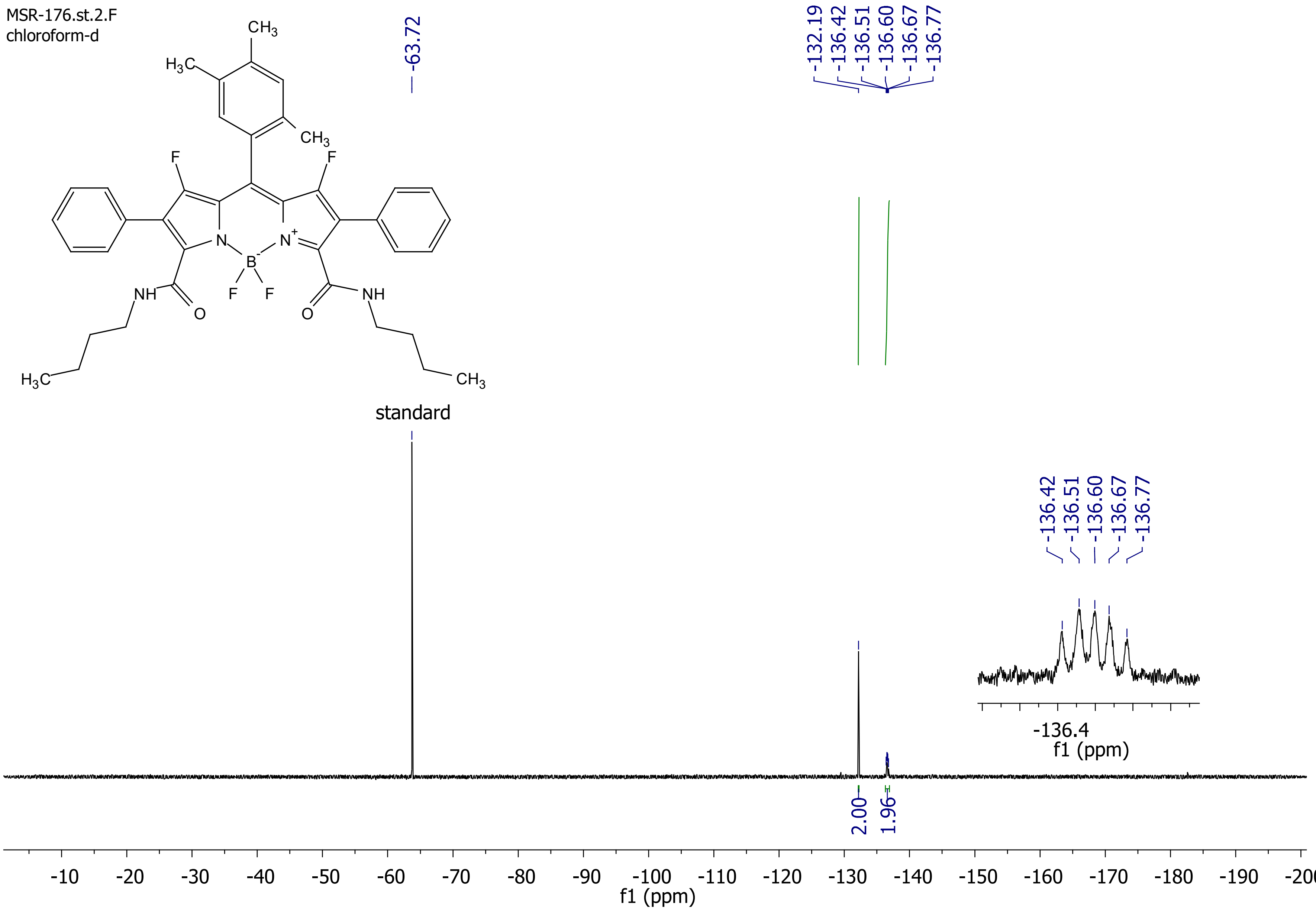

^{19}F NMR spectrum of 3,7-bis(butylcarbamoyl)-1,5,5,9-tetrafluoro-2,8-diphenyl-10-(2,4,5-trimethylphenyl)-5*H*-dipyrrolo[1,2-c:2',1'-f][1,3,2]diazaborinin-4-ium-5-uide (**5a**) in $CDCl_3$ at 376 MHz

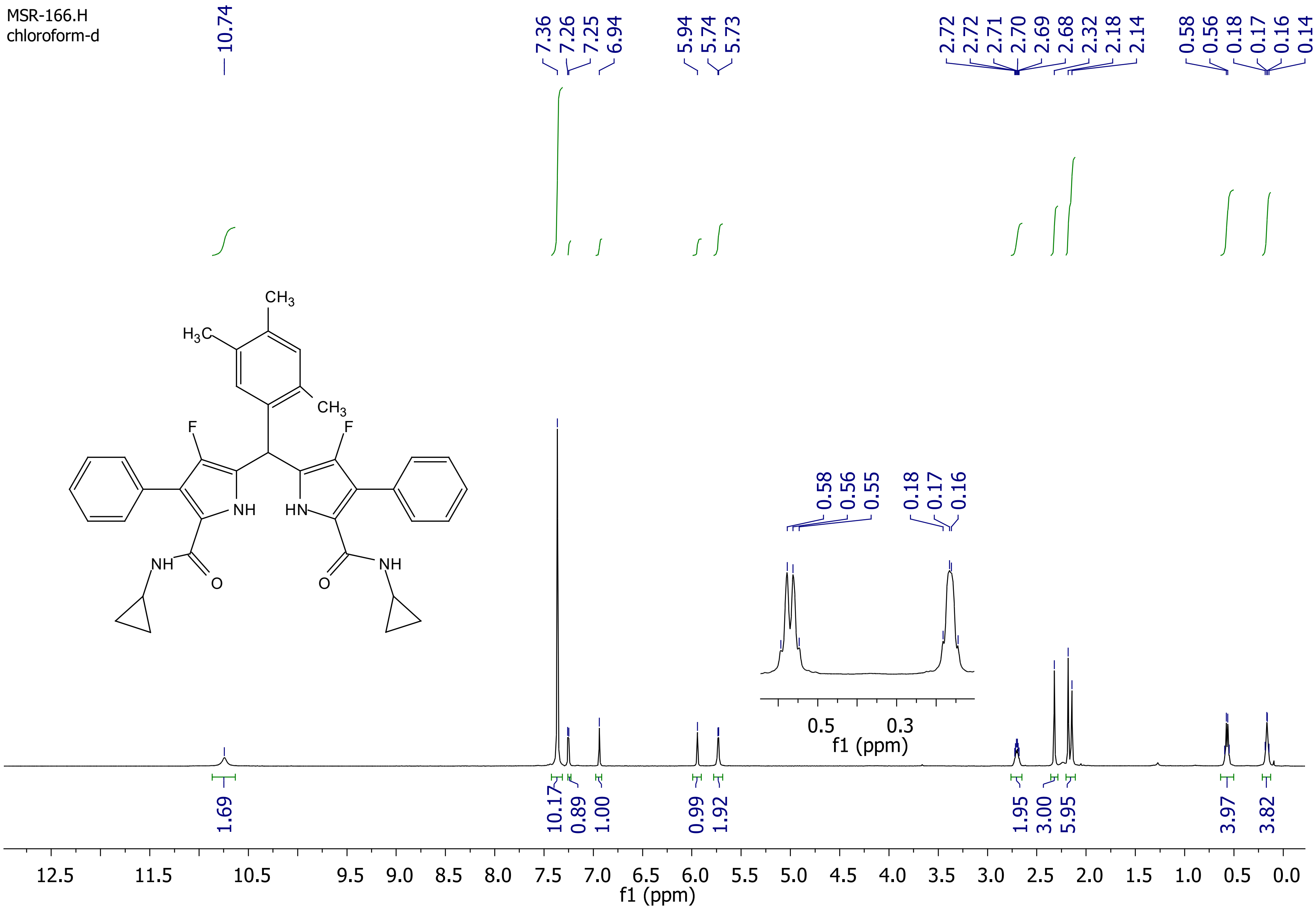

^{1}H NMR spectrum of 5,5'-((2,4,5-trimethylphenyl)methylene)bis(*N*-cyclopropyl-4-fluoro-3-phenyl-1*H*-pyrrole-2-carboxamide) (**4b**) in $CDCl_3$ at 400 MHz

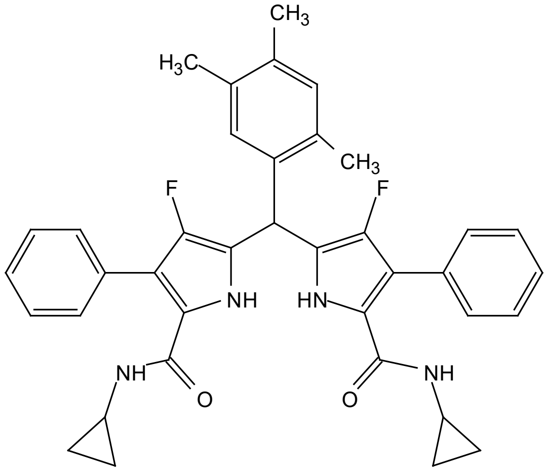

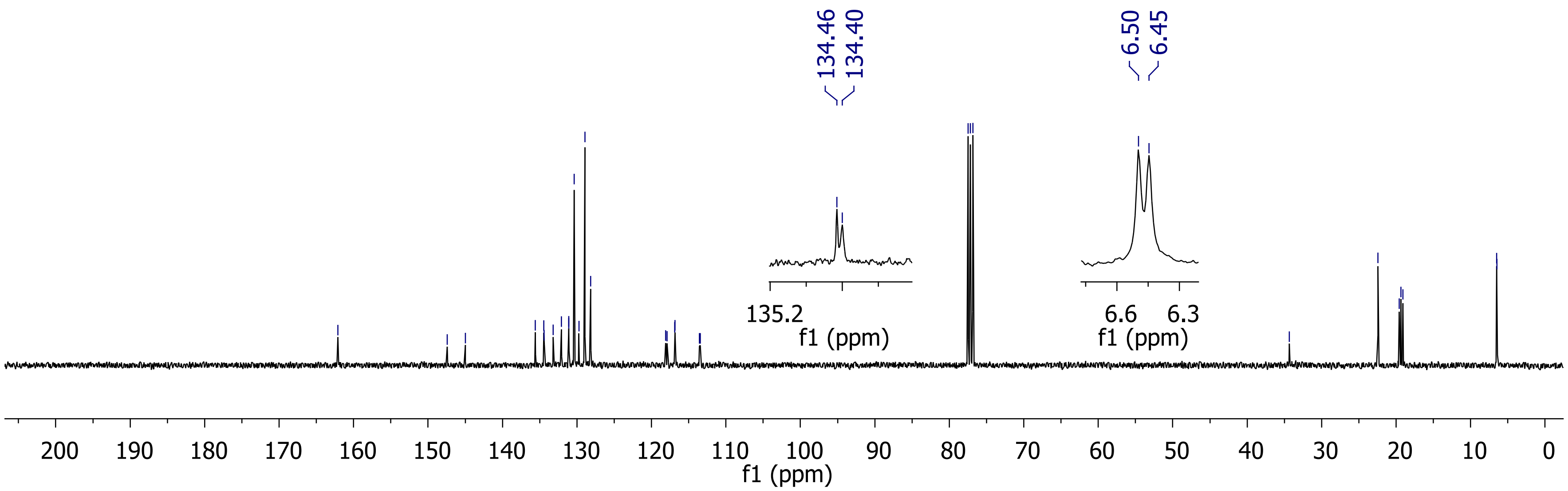

$^{13}C\{^1H\}$ NMR spectrum of 5,5'-((2,4,5-trimethylphenyl)methylene)bis(*N*-cyclopropyl-4-fluoro-3-phenyl-1*H*-pyrrole-2-carboxamide) (**4b**) in $CDCl_3$ at 100 MHz

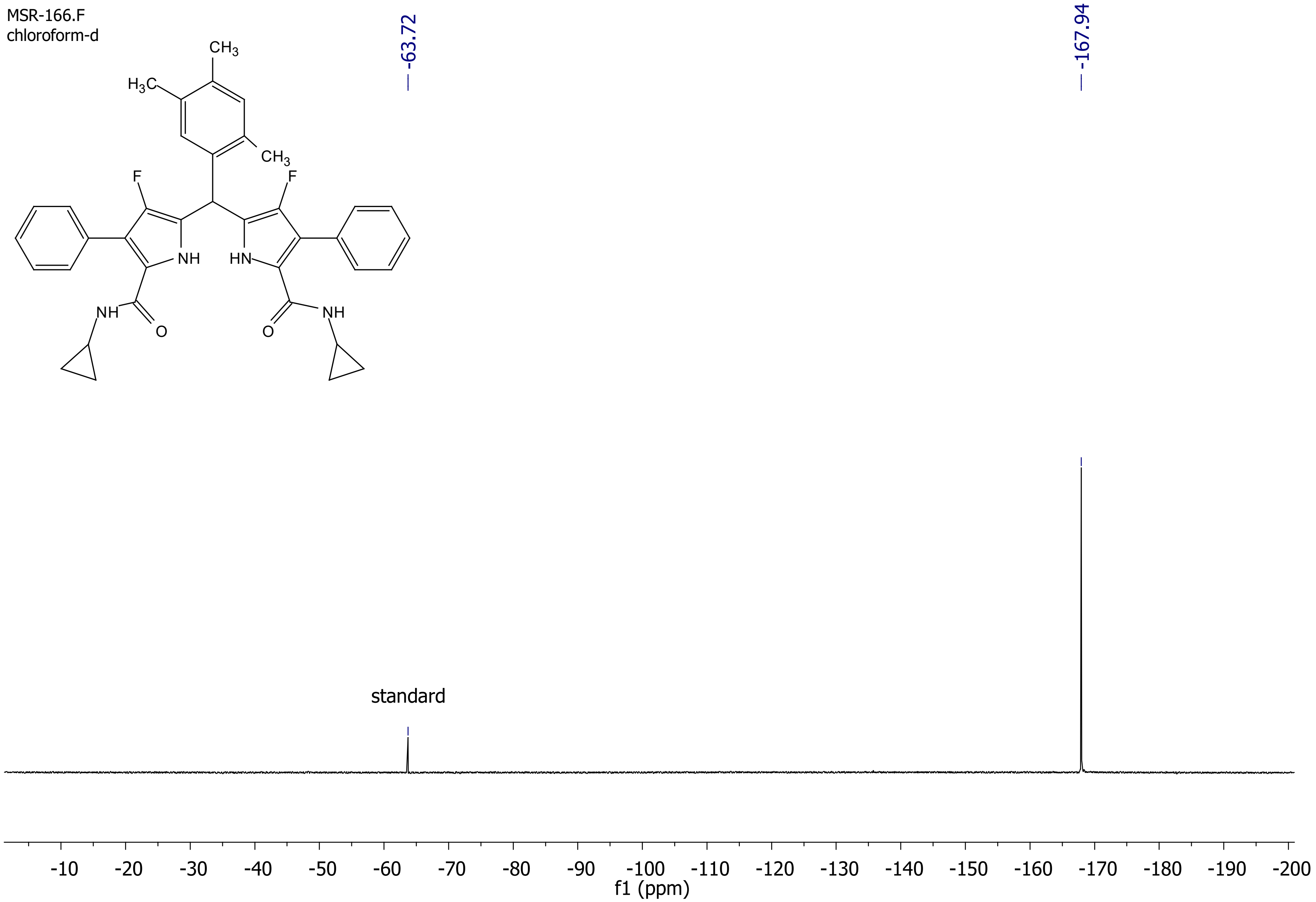

^{19}F NMR spectrum of 5,5'-((2,4,5-trimethylphenyl)methylene)bis(*N*-cyclopropyl-4-fluoro-3-phenyl-1*H*-pyrrole-2-carboxamide) (**4b**) in $CDCl_3$ at 376 MHz

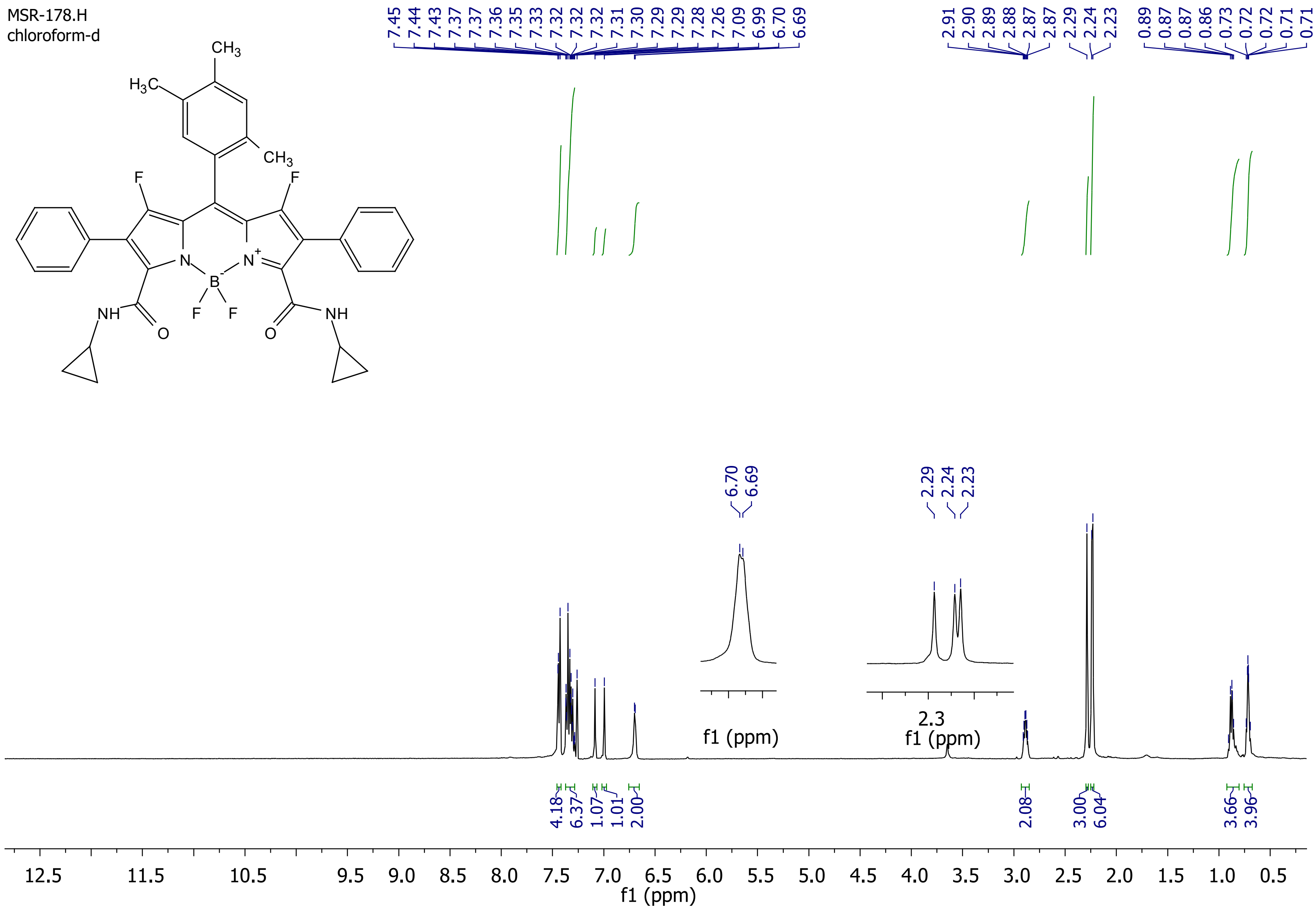

^{1}H NMR spectrum of 3,7-bis(cyclopropylcarbamoyl)-1,5,5,9-tetrafluoro-2,8-diphenyl-10-(2,4,5-trimethylphenyl)-5*H*-dipyrrolo[1,2-c:2',1'-f][1,3,2]diazaborinin-4-ium-5-uide (**5b**) in $CDCl_3$ at 400 MHz

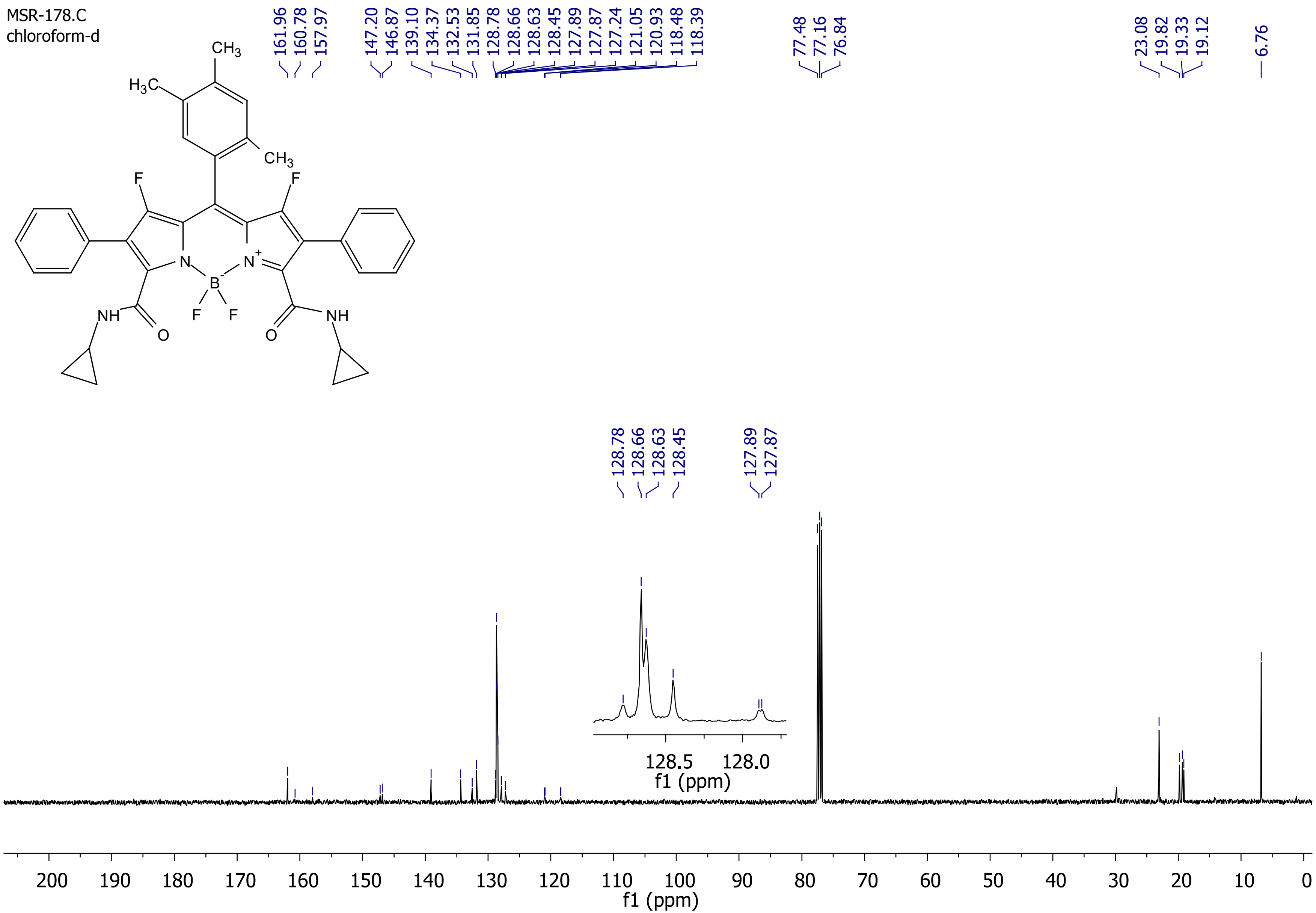

$^{13}C\{^{1}H\}$ NMR spectrum of 3,7-bis(cyclopropylcarbamoyl)-1,5,5,9-tetrafluoro-2,8-diphenyl-10-(2,4,5-trimethylphenyl)-5*H*-dipyrrolo[1,2-c:2',1'-f][1,3,2]diazaborinin-4-ium-5-uide (**5b**) in $CDCl_3$ at 100 MHz

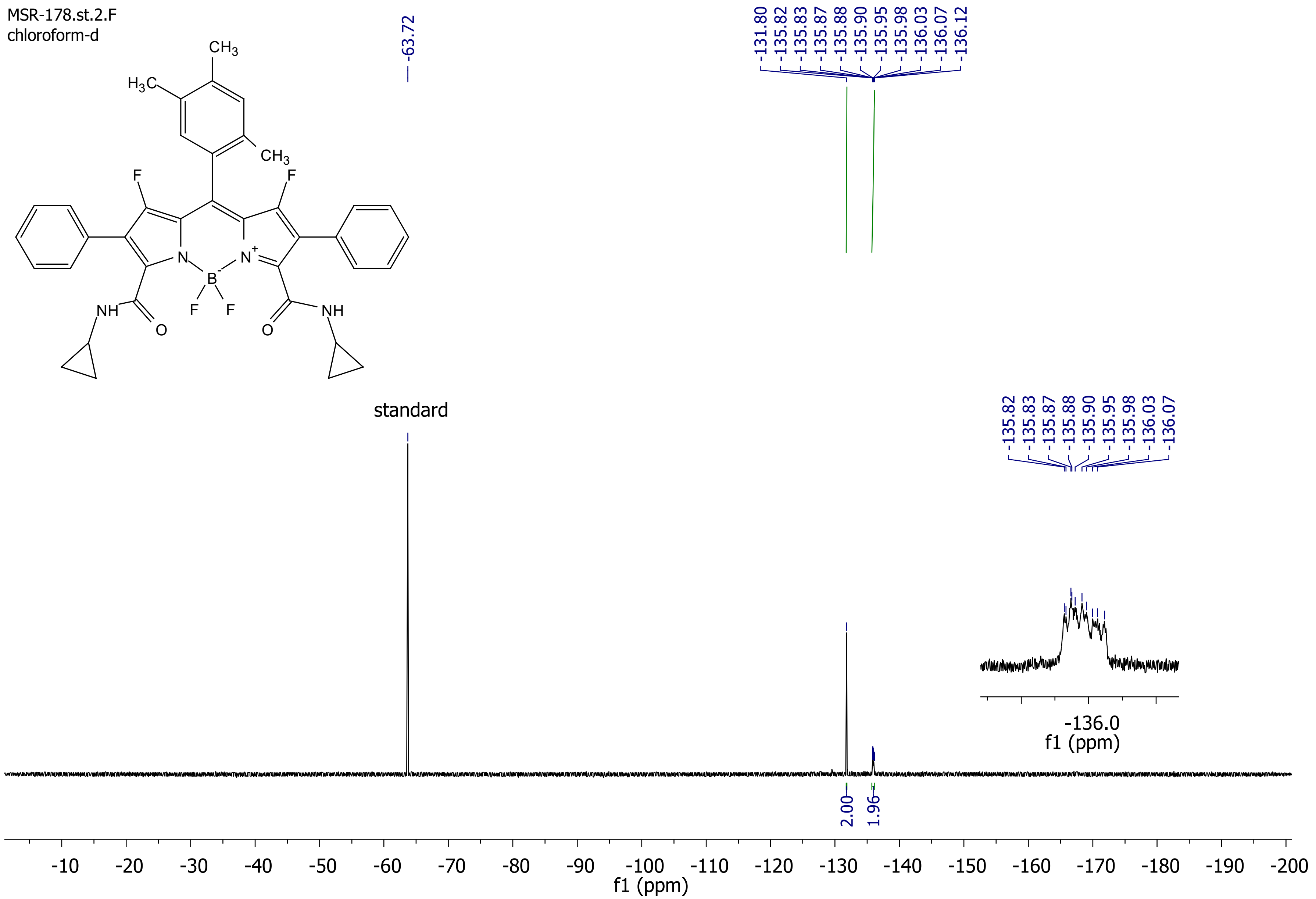

^{19}F NMR spectrum of 3,7-bis(cyclopropylcarbamoyl)-1,5,5,9-tetrafluoro-2,8-diphenyl-10-(2,4,5-trimethylphenyl)-5*H*-dipyrrolo[1,2-c:2',1'-f][1,3,2]diazaborinin-4-ium-5-uide (**5b**) in $CDCl_3$ at 376 MHz

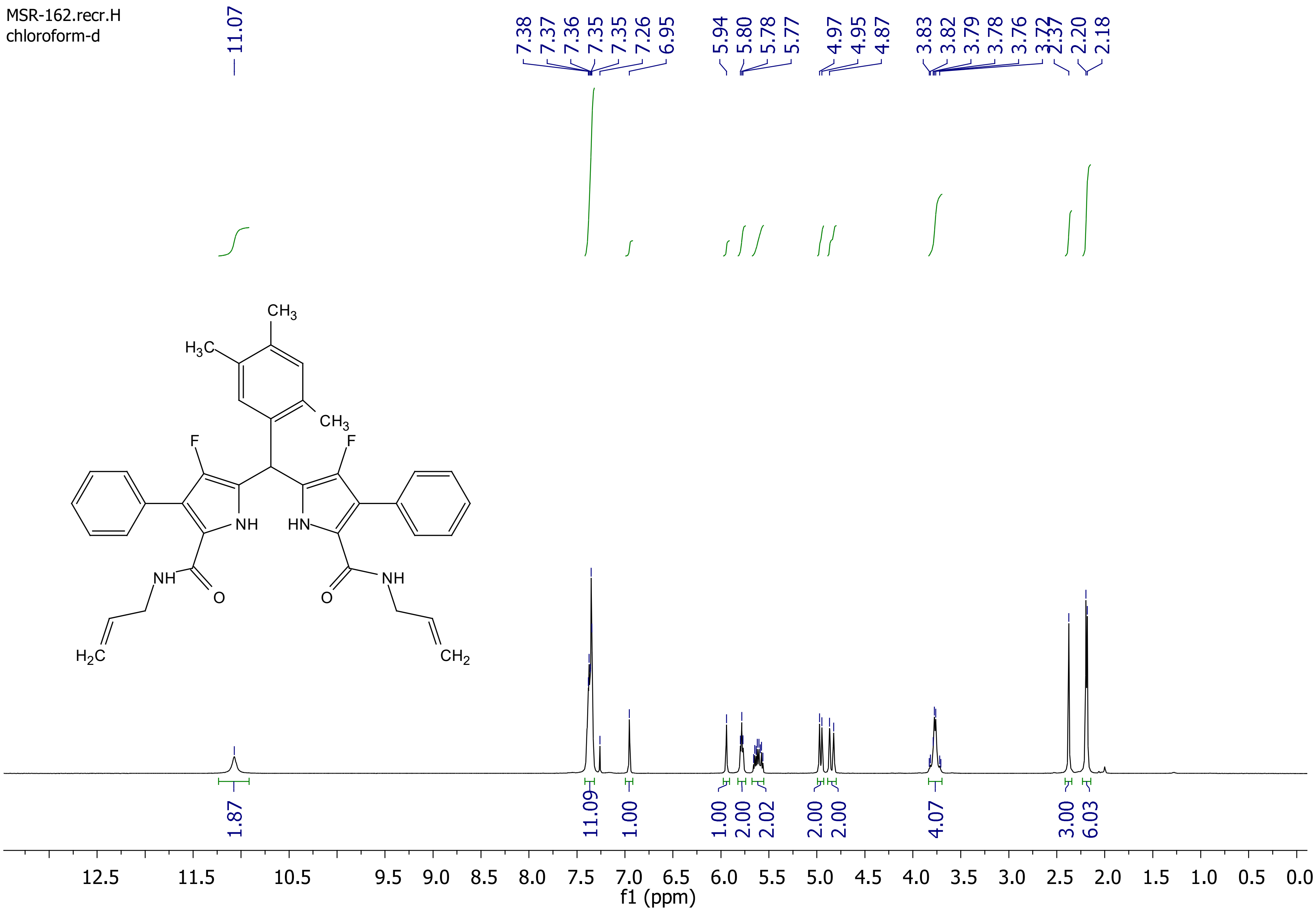

^{1}H NMR spectrum of 5,5'-((2,4,5-trimethylphenyl)methylene)bis(*N*-allyl-4-fluoro-3-phenyl-1*H*-pyrrole-2-carboxamide) (**4c**) in $CDCl_3$ at 400 MHz

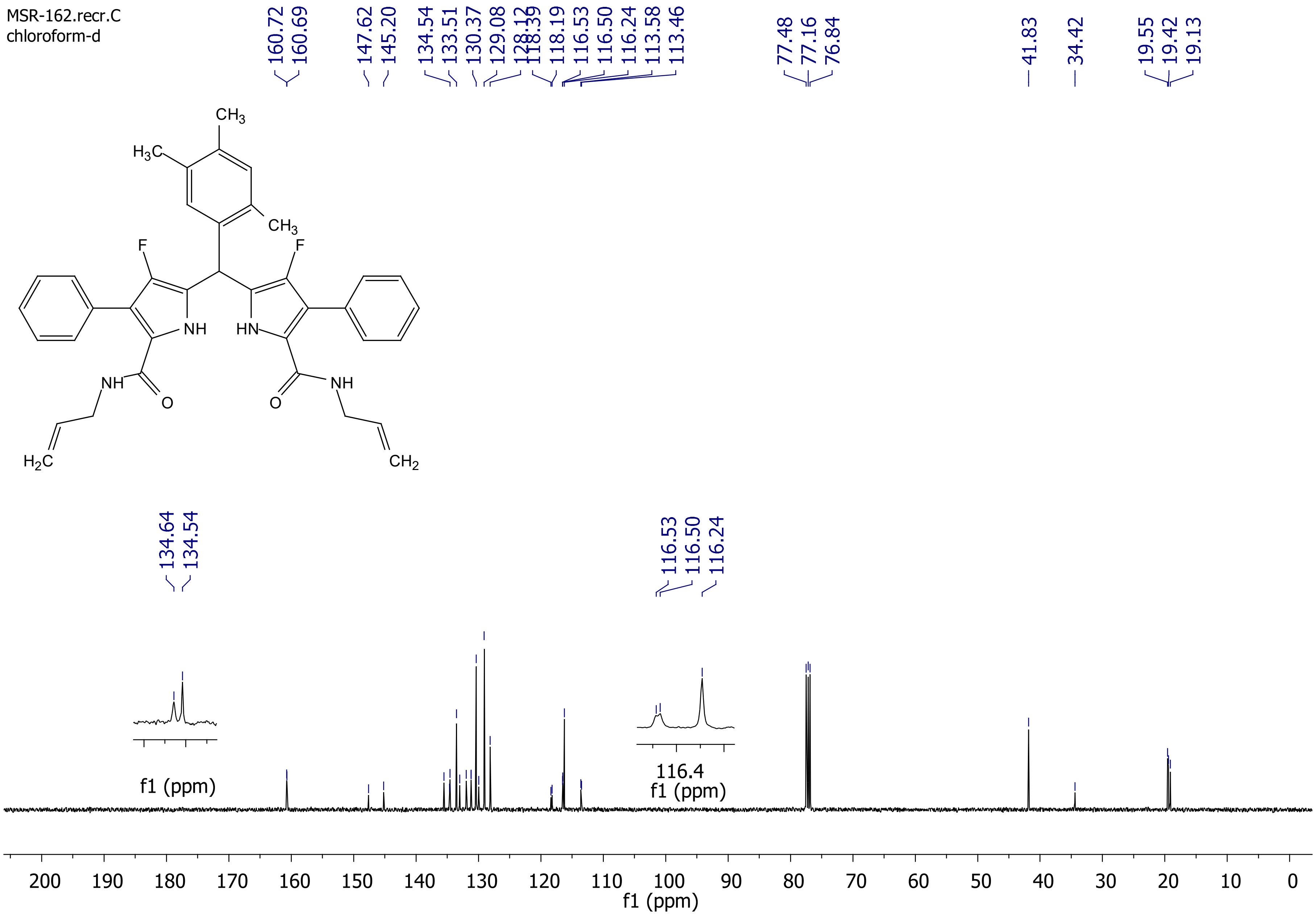

$^{13}C\{^{1}H\}$ NMR spectrum of 5,5'-((2,4,5-trimethylphenyl)methylene)bis(*N*-allyl-4-fluoro-3-phenyl-1*H*-pyrrole-2-carboxamide) (**4c**) in $CDCl_3$ at 100 MHz

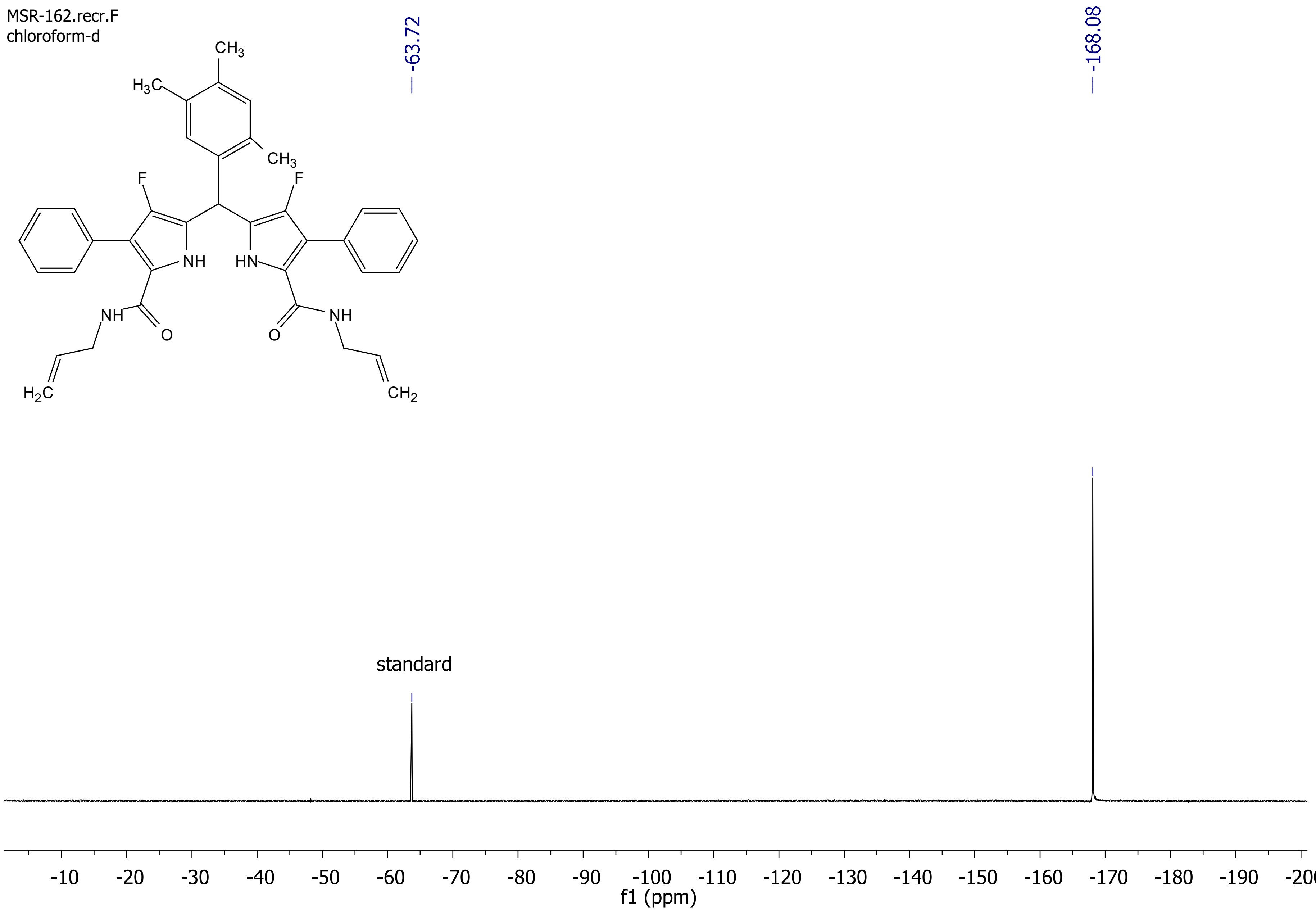

^{19}F NMR spectrum of 5,5'-((2,4,5-trimethylphenyl)methylene)bis(*N*-allyl-4-fluoro-3-phenyl-1*H*-pyrrole-2-carboxamide) (**4c**) in $CDCl_3$ at 376 MHz

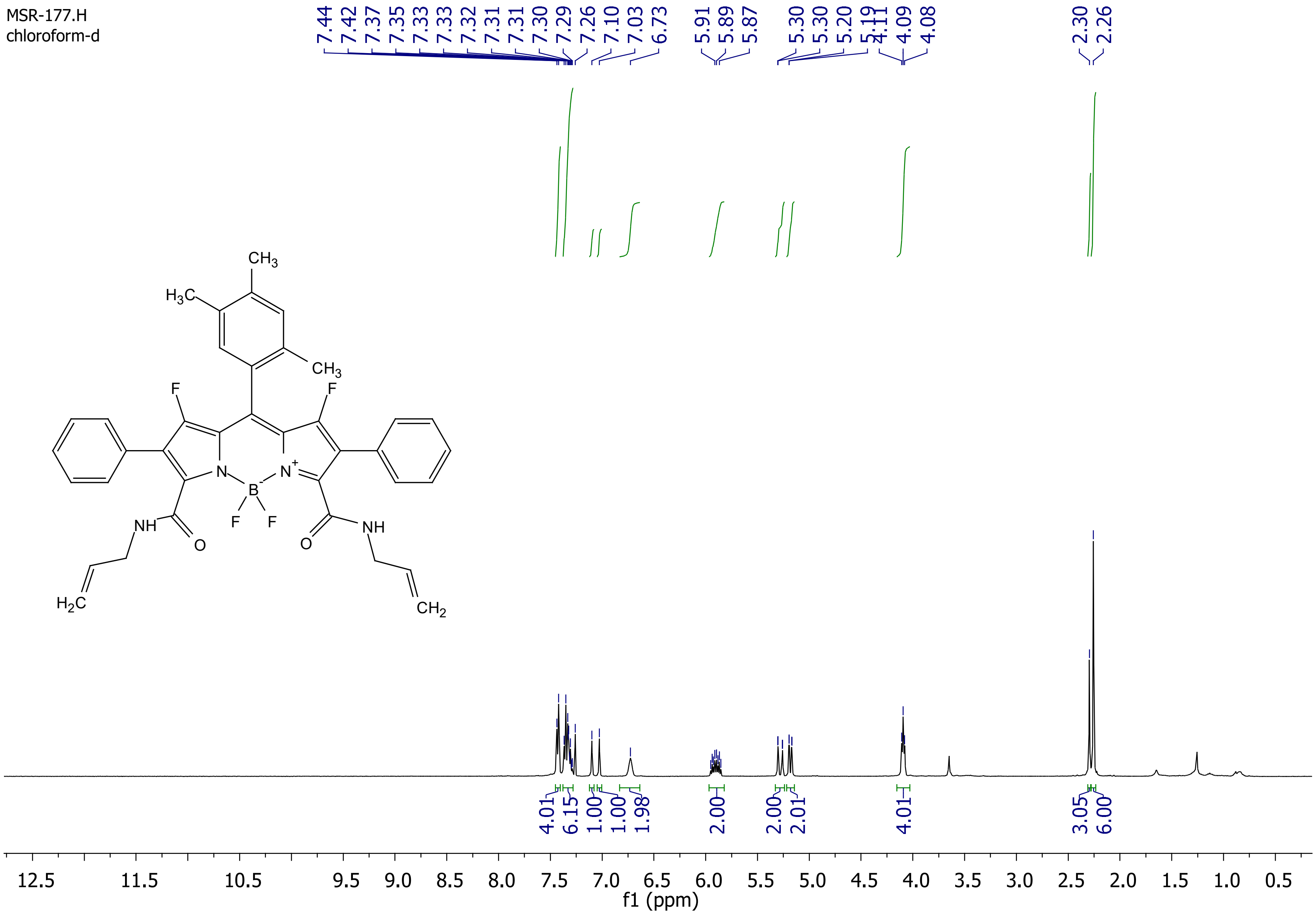

^{1}H NMR spectrum of 3,7-bis(allylcarbamoyl)-1,5,5,9-tetrafluoro-2,8-diphenyl-10-(2,4,5-trimethylphenyl)-5*H*-dipyrrolo[1,2-c:2',1'-f][1,3,2]diazaborinin-4-ium-5-uide (**5c**) in $CDCl_3$ at 400 MHz

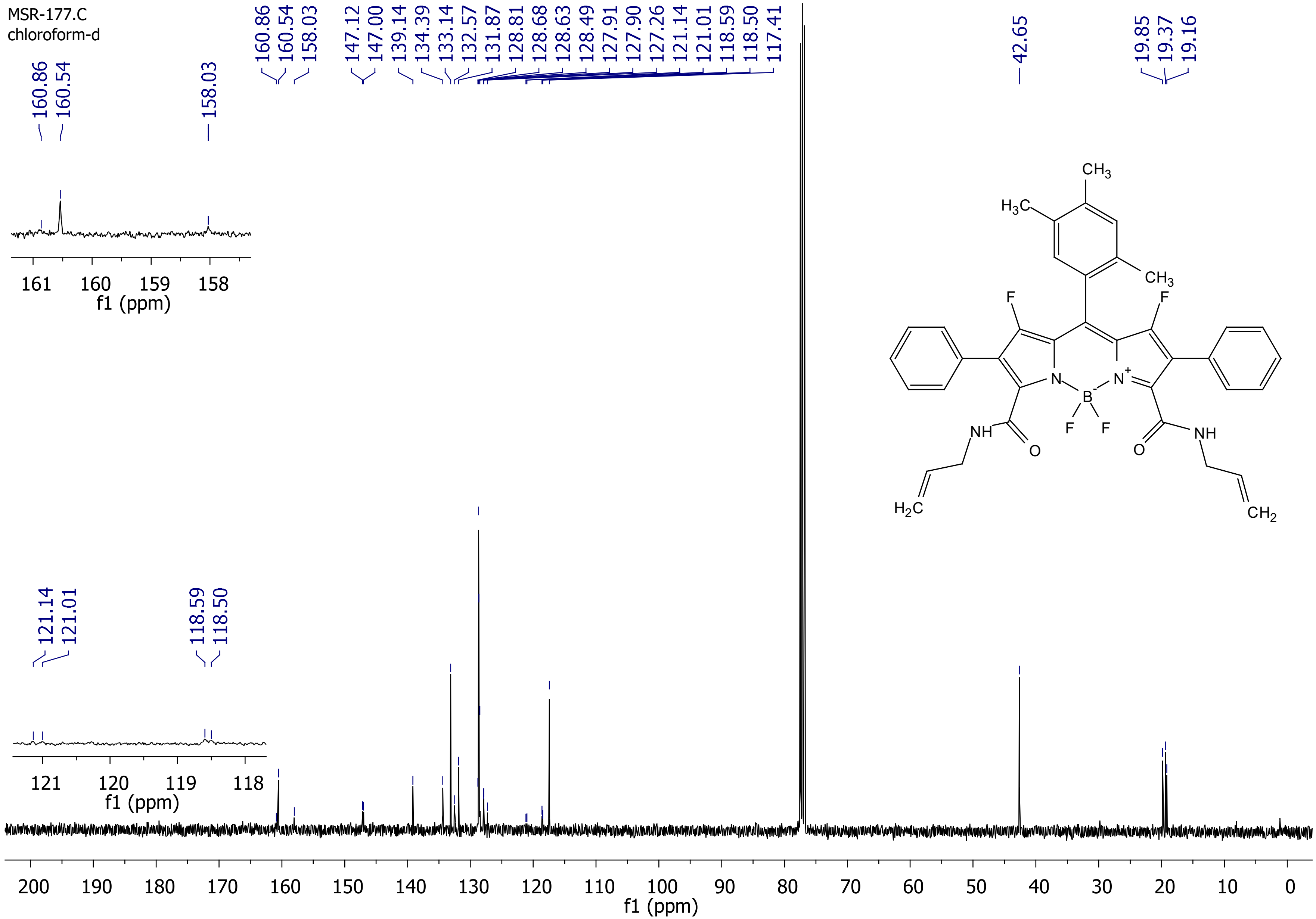

$^{13}C\{^1H\}$ NMR spectrum of 3,7-bis(allylcarbamoyl)-1,5,5,9-tetrafluoro-2,8-diphenyl-10-(2,4,5-trimethylphenyl)-5*H*-dipyrrolo[1,2-c:2',1'-f][1,3,2]diazaborinin-4-ium-5-uide (**5c**) in $CDCl_3$ at 100 MHz

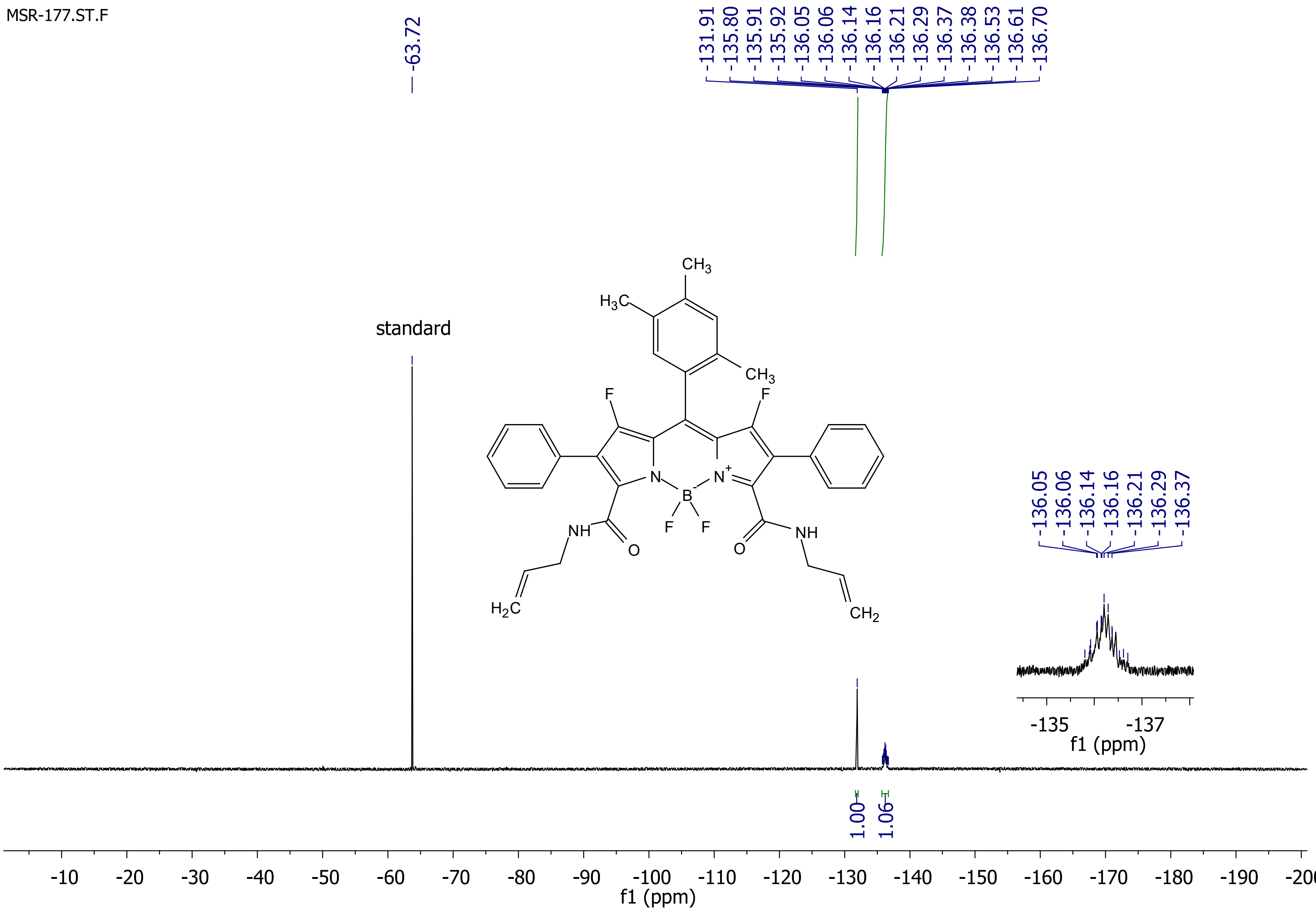

^{19}F NMR spectrum of 3,7-bis(allylcarbamoyl)-1,5,5,9-tetrafluoro-2,8-diphenyl-10-(2,4,5-trimethylphenyl)-5*H*-dipyrrolo[1,2-c:2',1'-f][1,3,2]diazaborinin-4-ium-5-uide (**5c**) in $CDCl_3$ at 376 MHz

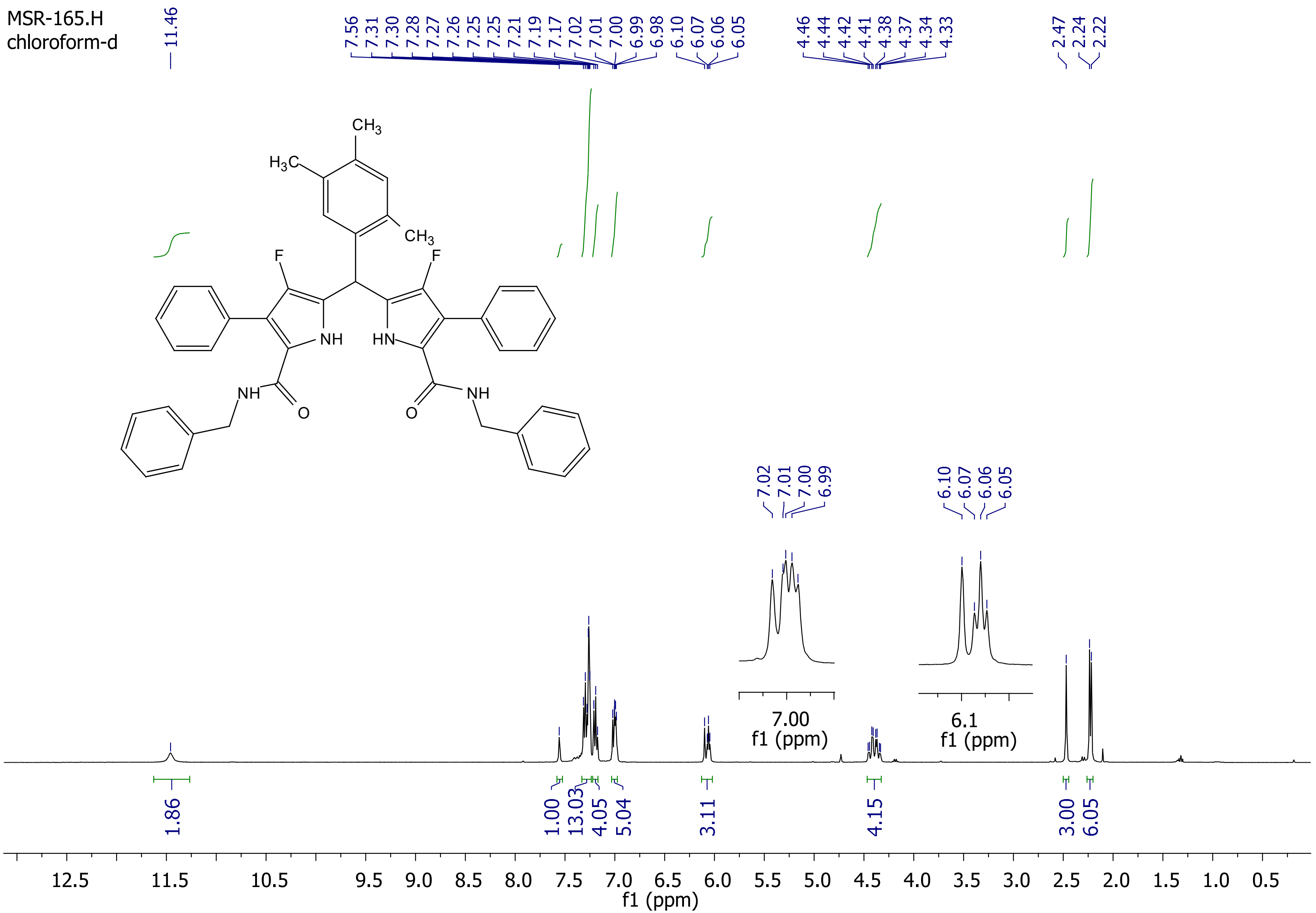

^{1}H NMR spectrum of 5,5'-((2,4,5-trimethylphenyl)methylene)bis(*N*-benzyl-4-fluoro-3-phenyl-1*H*-pyrrole-2-carboxamide) (**4d**) in $CDCl_3$ at 400 MHz

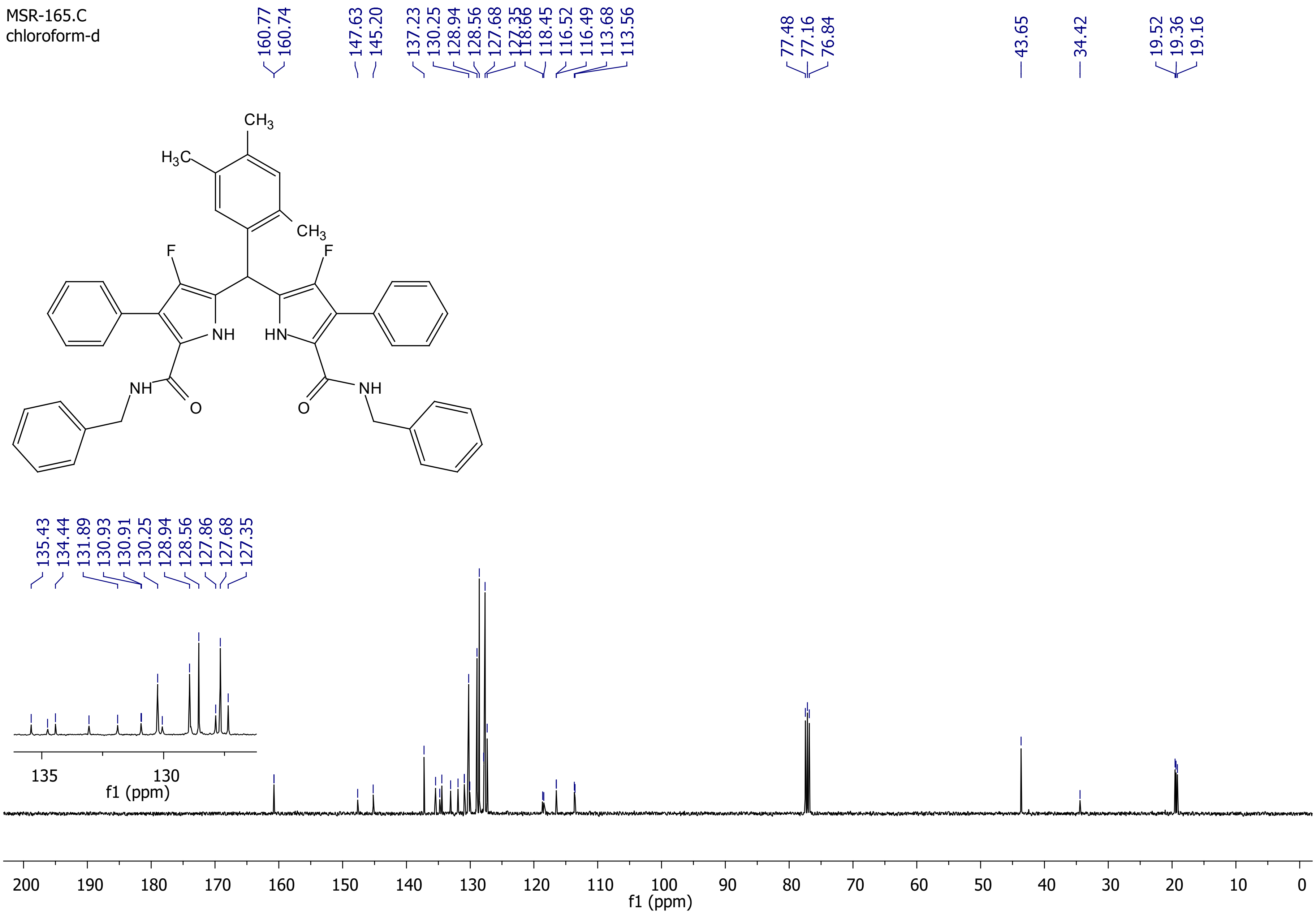

$^{13}C\{^{1}H\}$ NMR spectrum of 5,5'-((2,4,5-trimethylphenyl)methylene)bis(*N*-benzyl-4-fluoro-3-phenyl-1*H*-pyrrole-2-carboxamide) (**4d**) in $CDCl_3$ at 100 MHz

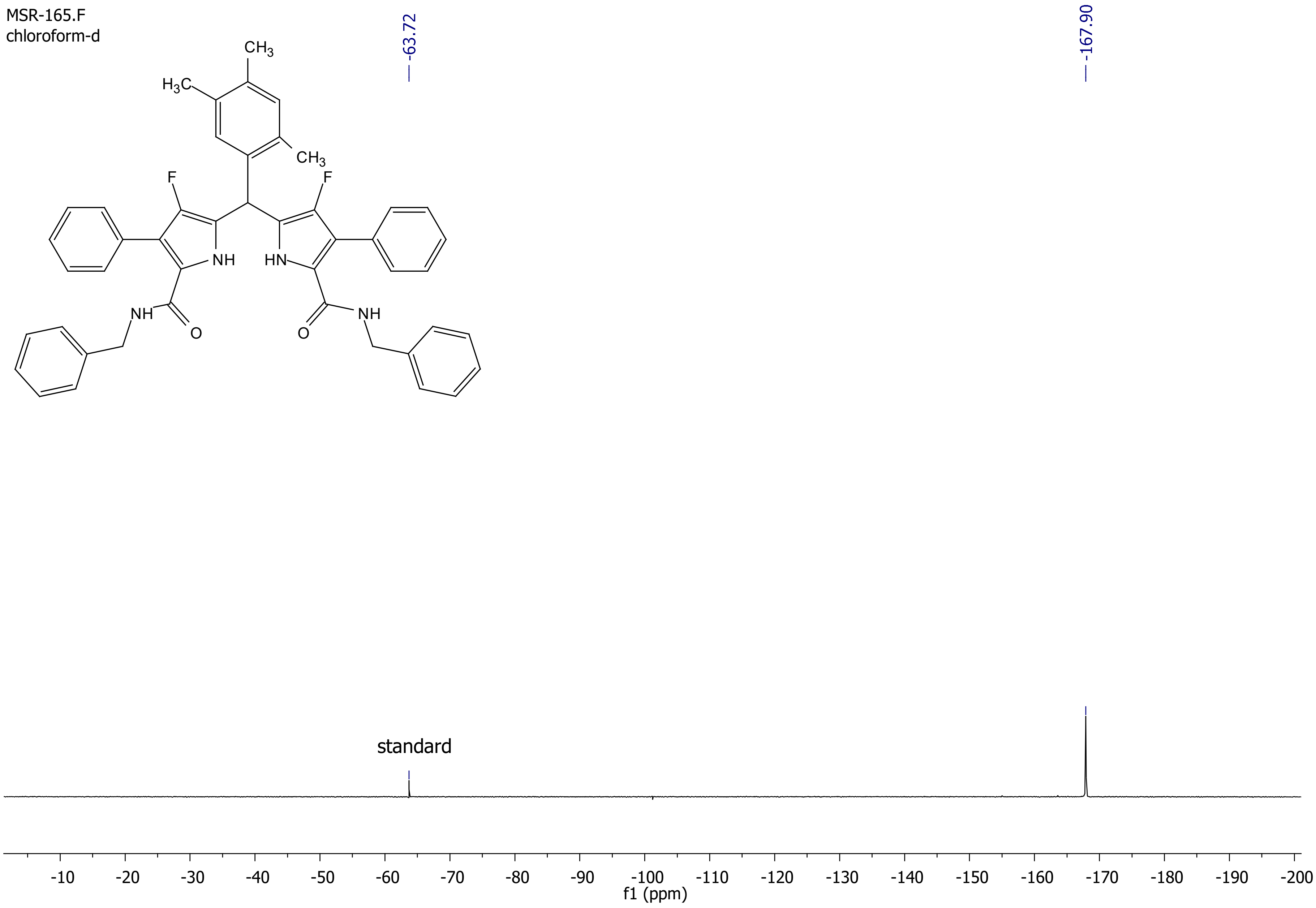

^{19}F NMR spectrum of 5,5'-((2,4,5-trimethylphenyl)methylene)bis(*N*-benzyl-4-fluoro-3-phenyl-1*H*-pyrrole-2-carboxamide) (**4d**) in $CDCl_3$ at 376 MHz

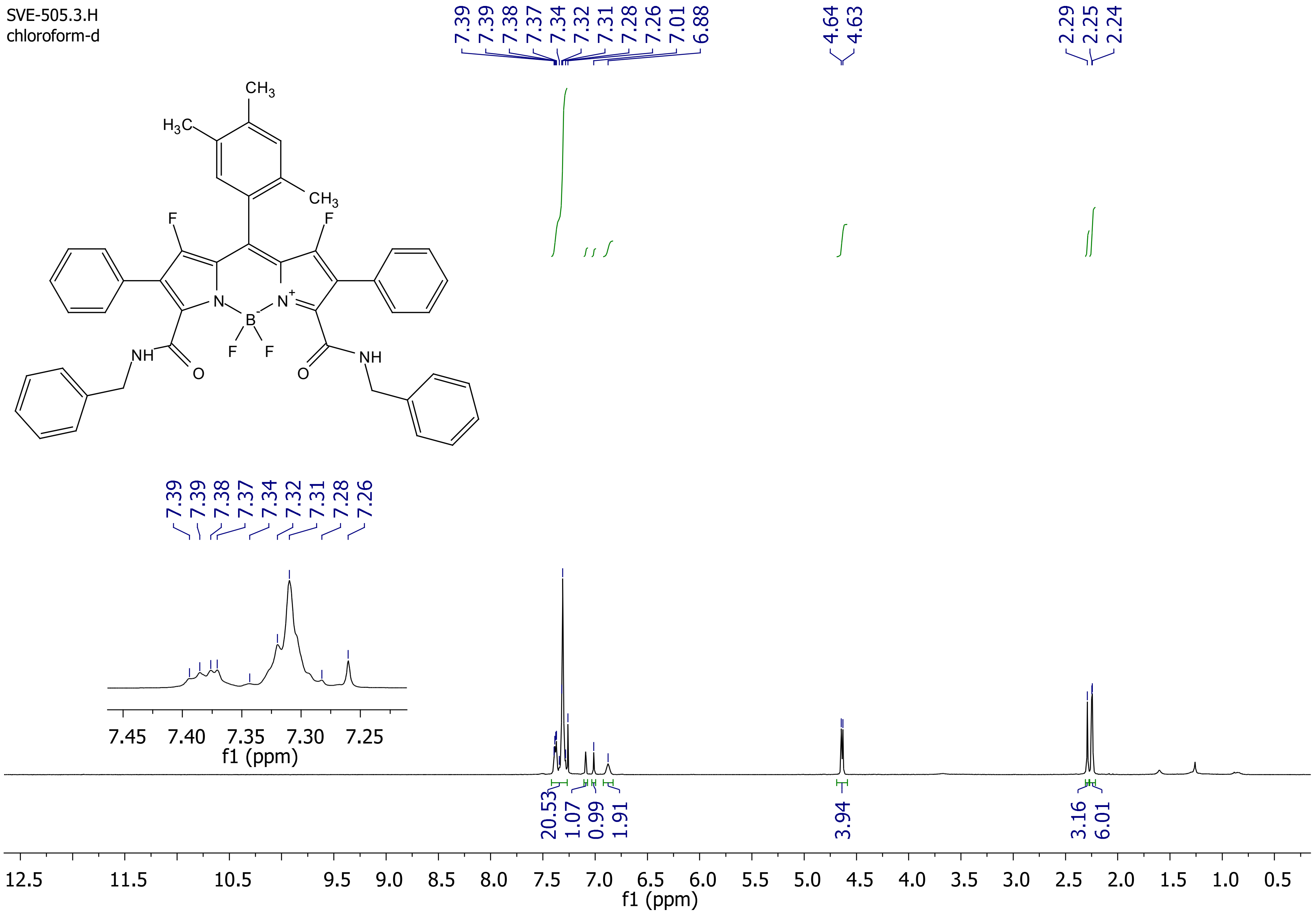

^{1}H NMR spectrum of 3,7-bis(benzylcarbamoyl)-1,5,5,9-tetrafluoro-2,8-diphenyl-10-(2,4,5-trimethylphenyl)-5*H*-dipyrrolo[1,2-c:2',1'-f][1,3,2]diazaborinin-4-ium-5-uide (**5d**) in $CDCl_3$ at 400 MHz

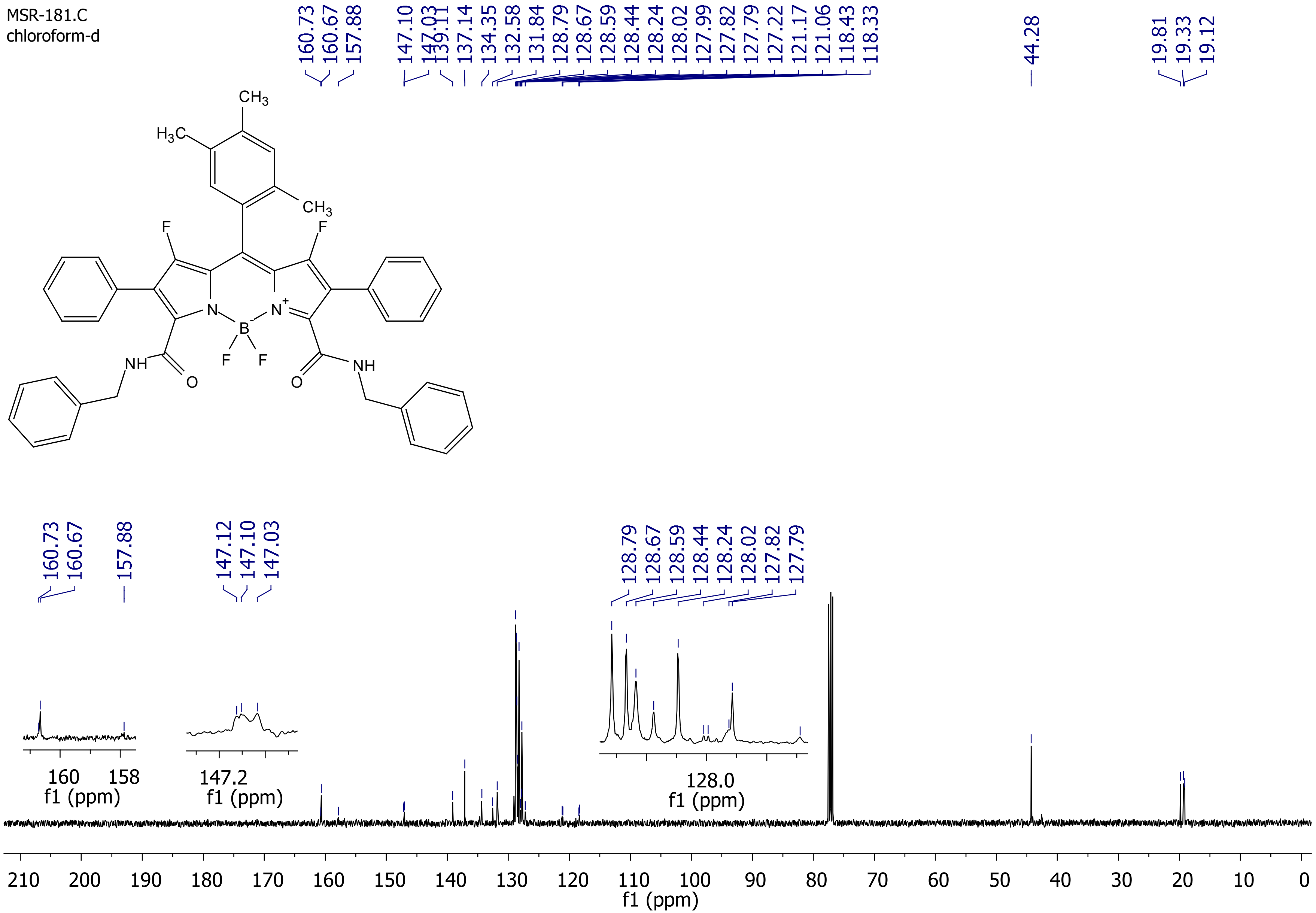

$^{13}C\{^1H\}$ NMR spectrum of 3,7-bis(benzylcarbamoyl)-1,5,5,9-tetrafluoro-2,8-diphenyl-10-(2,4,5-trimethylphenyl)-5*H*-dipyrrolo[1,2-c:2',1'-f][1,3,2]diazaborinin-4-ium-5-uide (**5d**) in $CDCl_3$ at 100 MHz

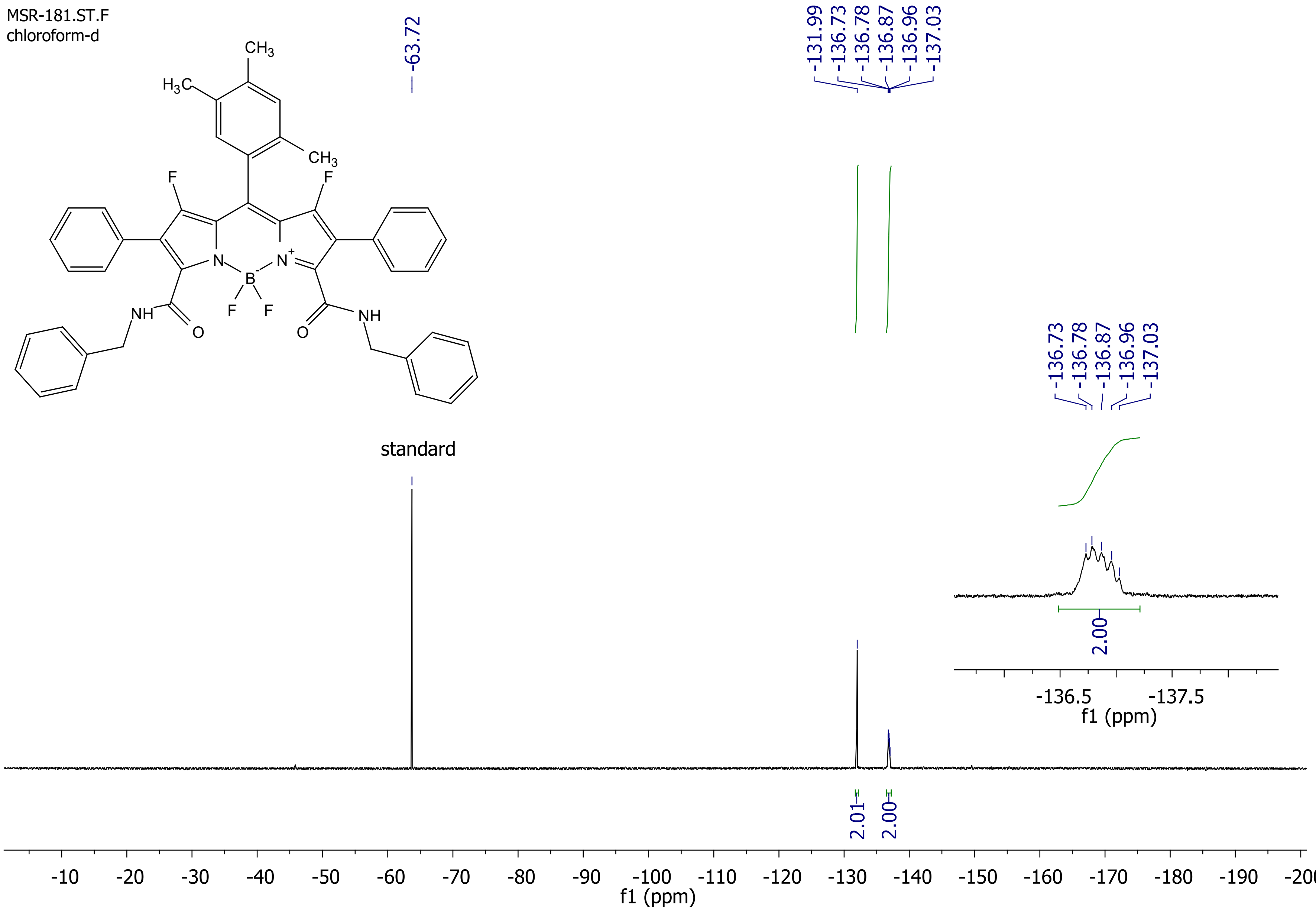

^{19}F NMR spectrum of 3,7-bis(benzylcarbamoyl)-1,5,5,9-tetrafluoro-2,8-diphenyl-10-(2,4,5-trimethylphenyl)-5*H*-dipyrrolo[1,2-c:2',1'-f][1,3,2]diazaborinin-4-ium-5-uide (**5d**) in $CDCl_3$ at 376 MHz

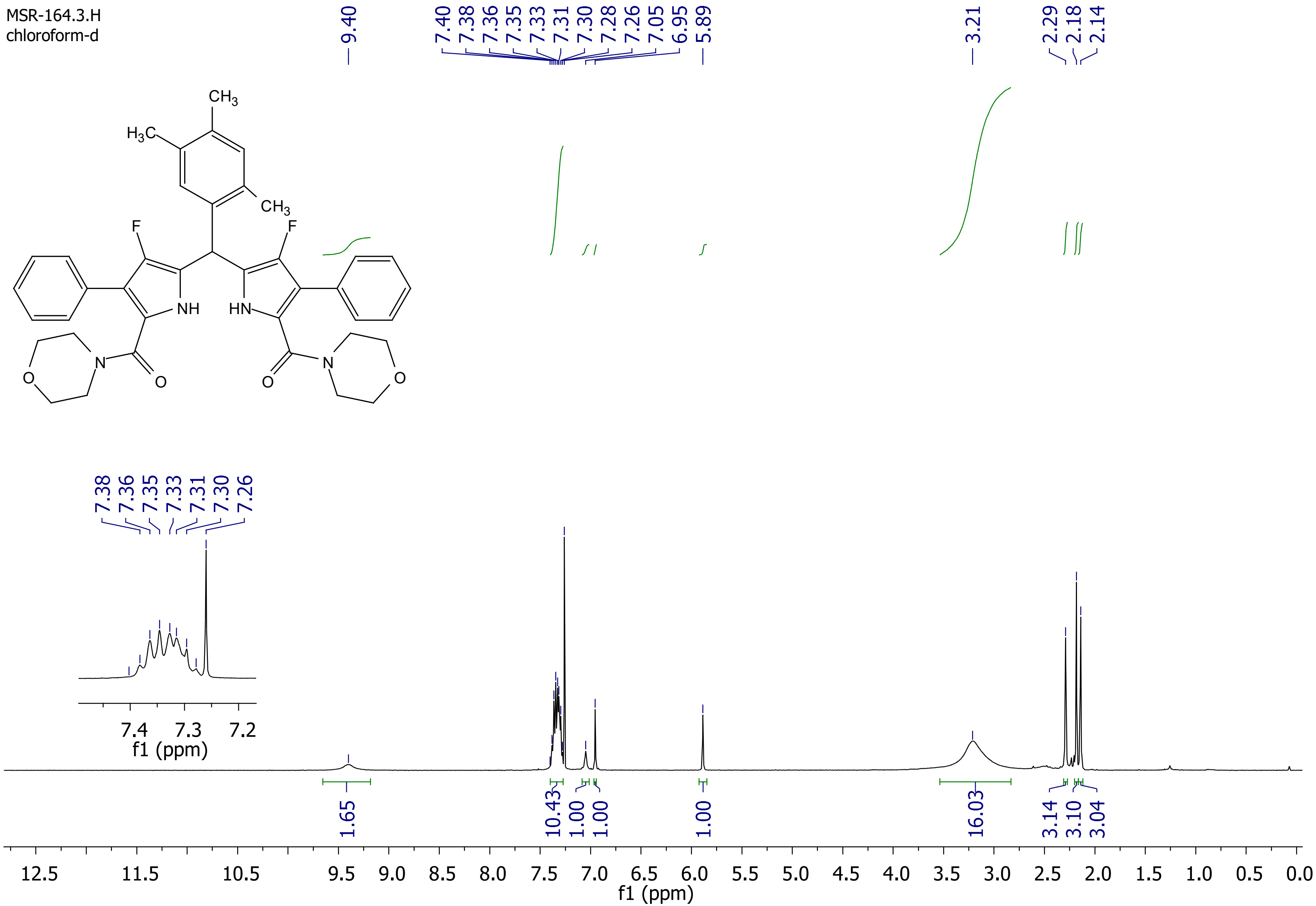

^{1}H NMR spectrum of (5,5'-((2,4,5-trimethylphenyl)methylene)bis(4-fluoro-3-phenyl-1*H*-pyrrole-5,2-diyl))bis(morpholinomethanone) (**4e**) in $CDCl_3$ at 400 MHz

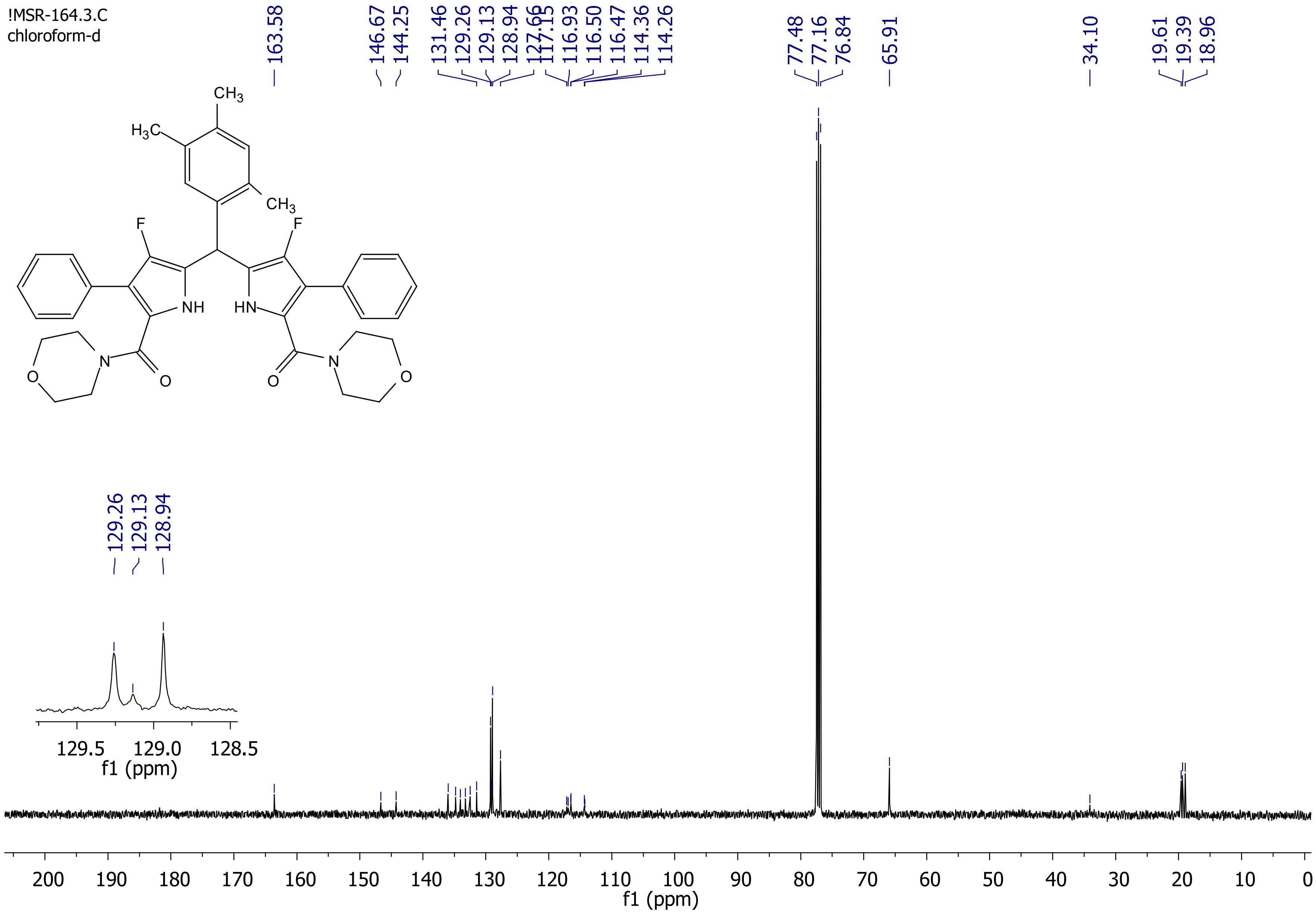

$^{13}C\{^{1}H\}$ NMR spectrum of (5,5'-((2,4,5-trimethylphenyl)methylene)bis(4-fluoro-3-phenyl-1*H*-pyrrole-5,2-diyl))bis(morpholinomethanone) (**4e**) in $CDCl_3$ at 100 MHz

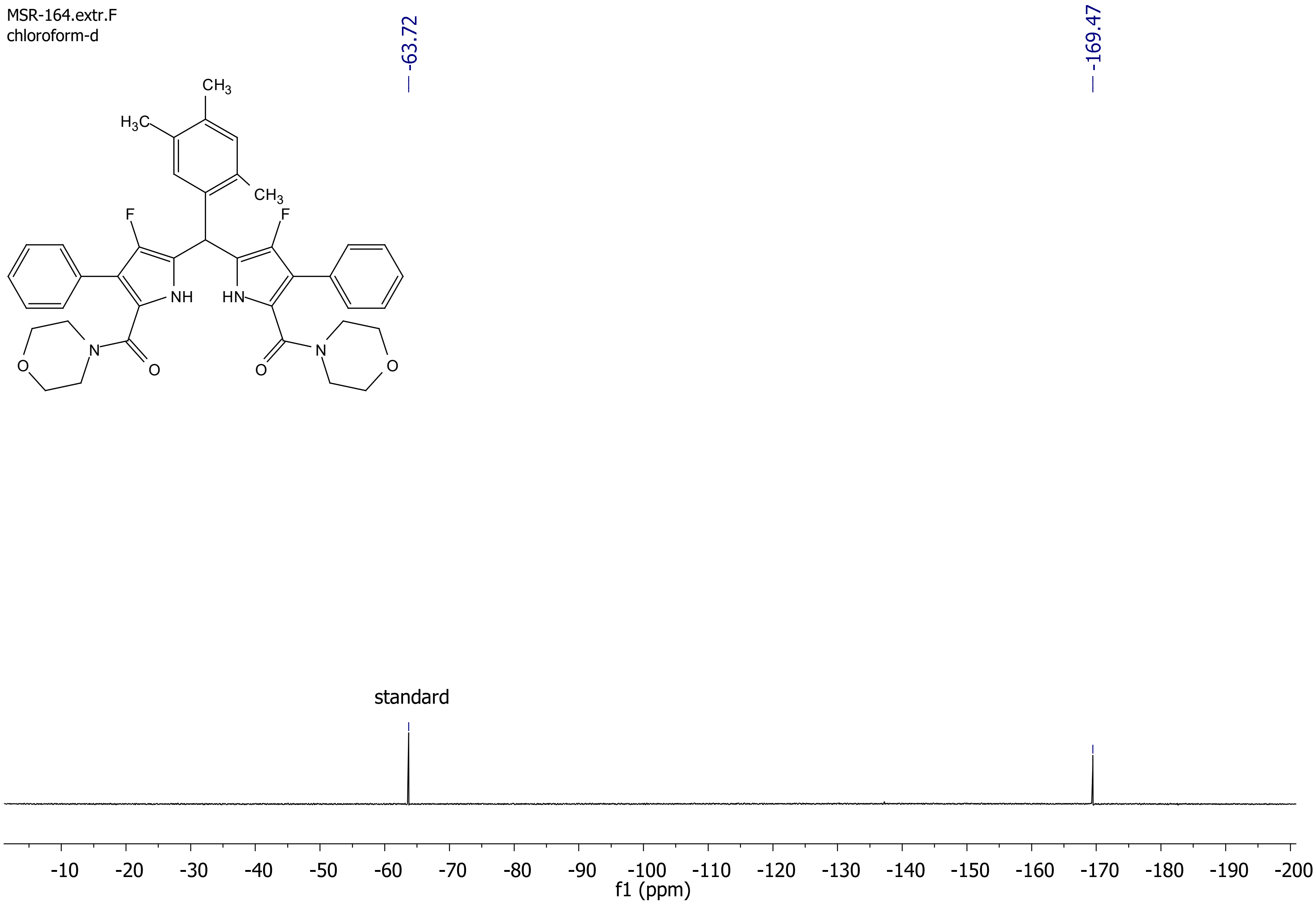

^{19}F NMR spectrum of (5,5'-((2,4,5-trimethylphenyl)methylene)bis(4-fluoro-3-phenyl-1*H*-pyrrole-5,2-diyl))bis(morpholinomethanone) (**4e**) in $CDCl_3$ at 376 MHz

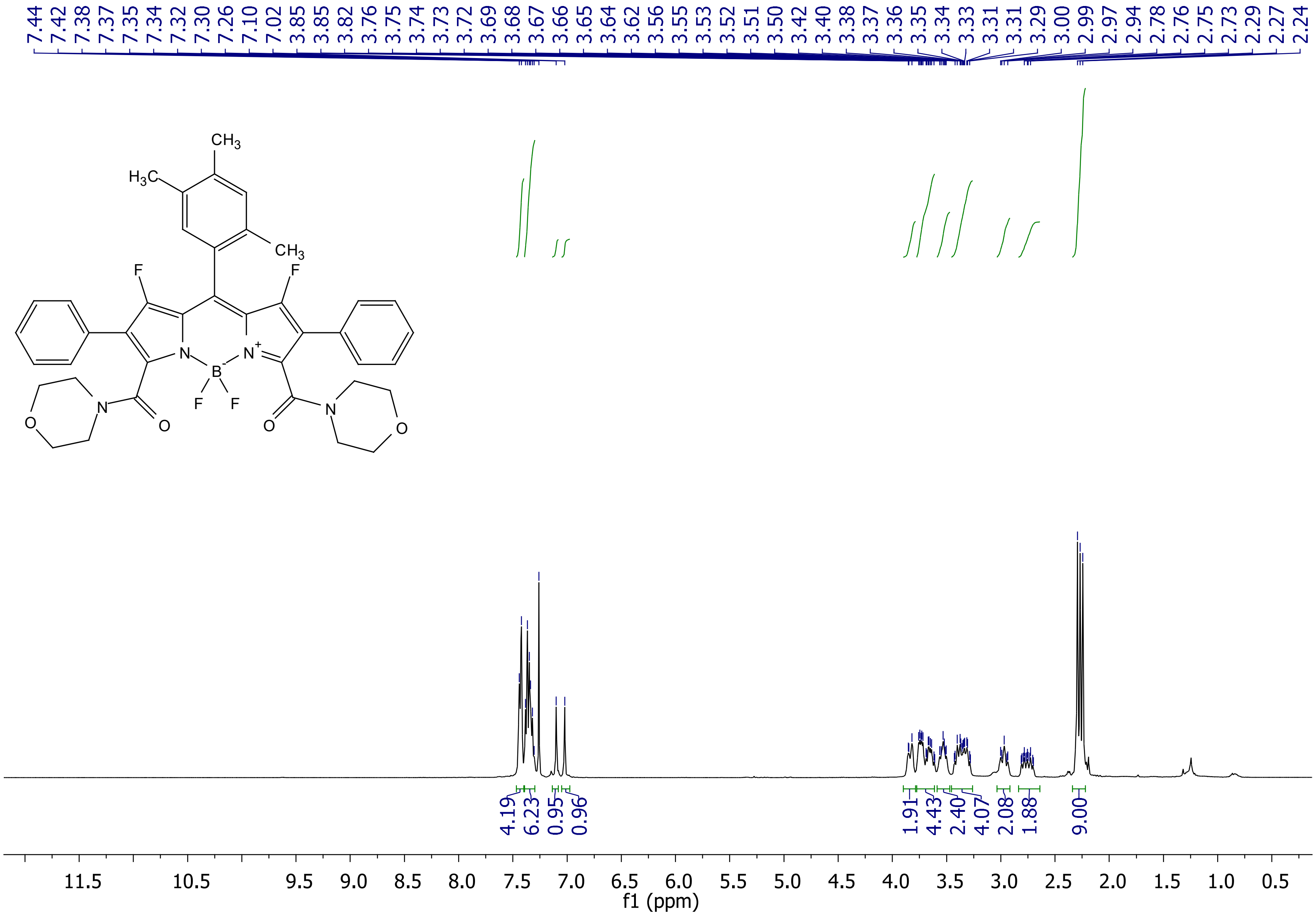

^{1}H NMR spectrum of 1,5,5,9-tetrafluoro-3,7-di(morpholine-4-carbonyl)-2,8-diphenyl-10-(2,4,5-trimethylphenyl)-5*H*-dipyrrolo[1,2-c:2',1'-f][1,3,2]diazaborinin-4-ium-5-uide (**5e**) in $CDCl_3$ at 400 MHz

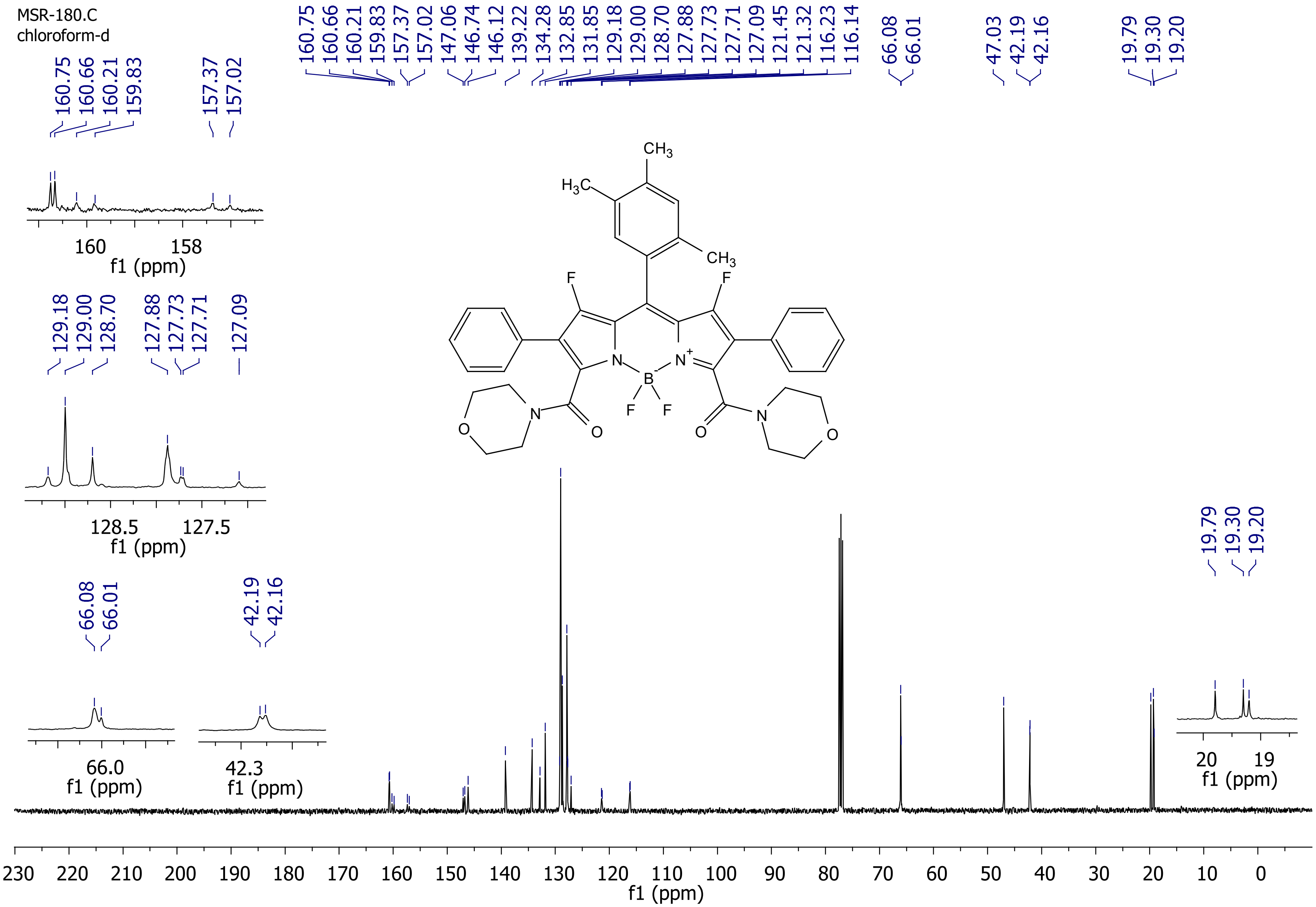

$^{13}C\{^{1}H\}$ NMR spectrum of 1,5,5,9-tetrafluoro-3,7-di(morpholine-4-carbonyl)-2,8-diphenyl-10-(2,4,5-trimethylphenyl)-5*H*-dipyrrolo[1,2-c:2',1'-f][1,3,2]diazaborinin-4-ium-5-uide (**5e**) in $CDCl_3$ at 100 MHz

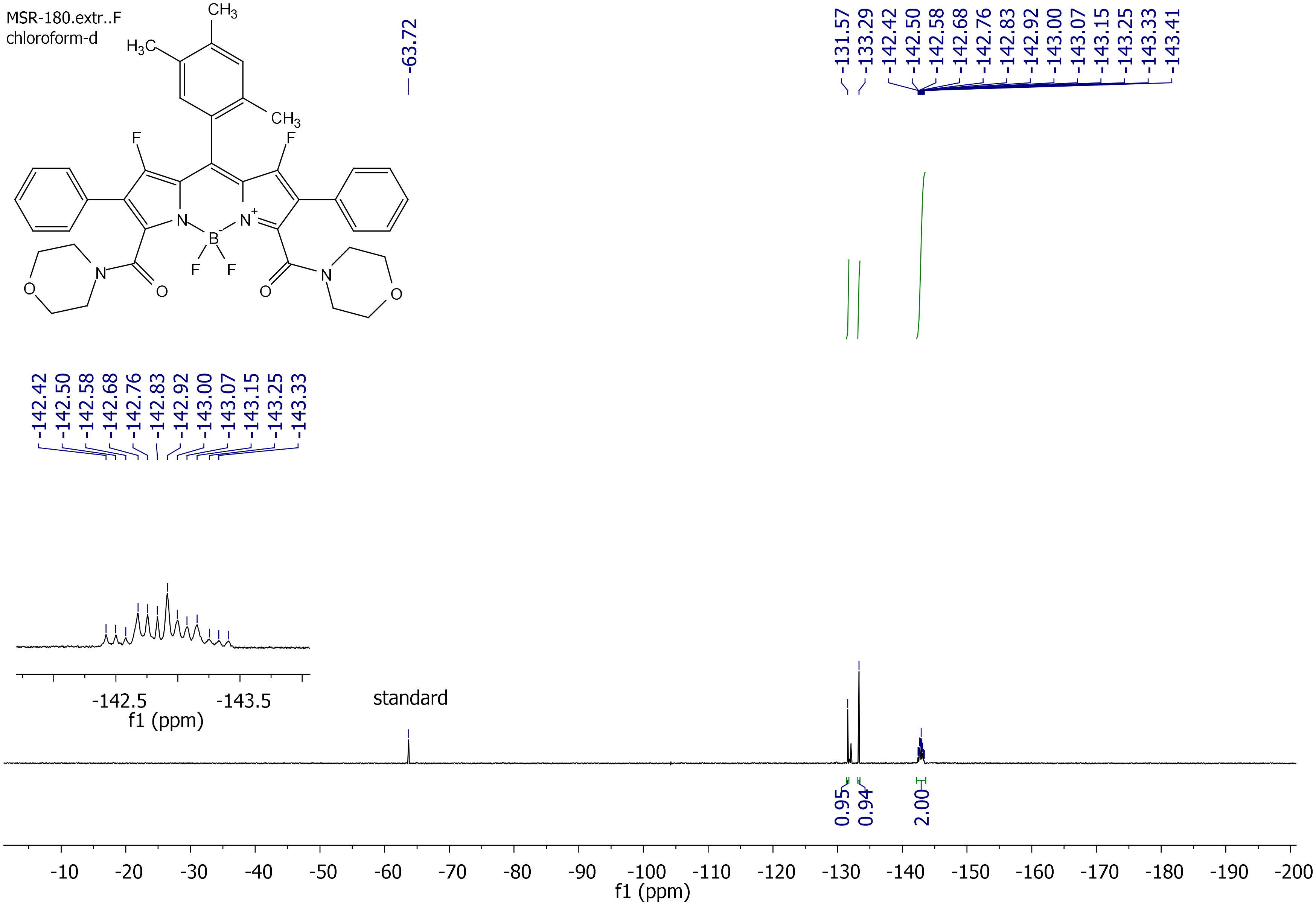

^{19}F NMR spectrum of 1,5,5,9-tetrafluoro-3,7-di(morpholine-4-carbonyl)-2,8-diphenyl-10-(2,4,5-trimethylphenyl)-5*H*-dipyrrolo[1,2-c:2',1'-f][1,3,2]diazaborinin-4-ium-5-uide (**5e**) in $CDCl_3$ at 376 MHz

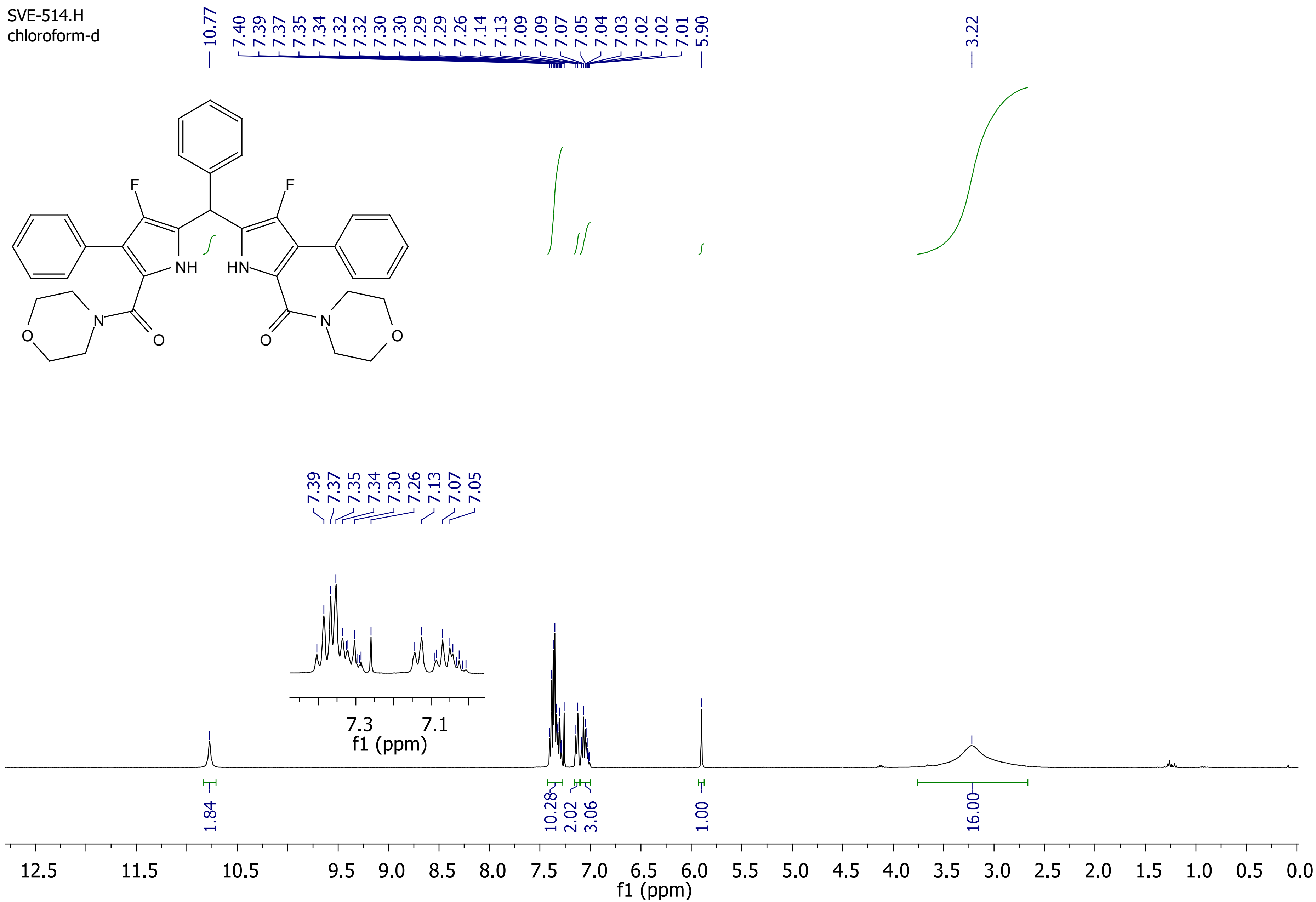

^{1}H NMR spectrum of (5,5'-(phenylmethylene)bis(4-fluoro-3-phenyl-1*H*-pyrrole-5,2-diyl))bis(morpholinomethanone) (**4f**) in $CDCl_3$ at 400 MHz

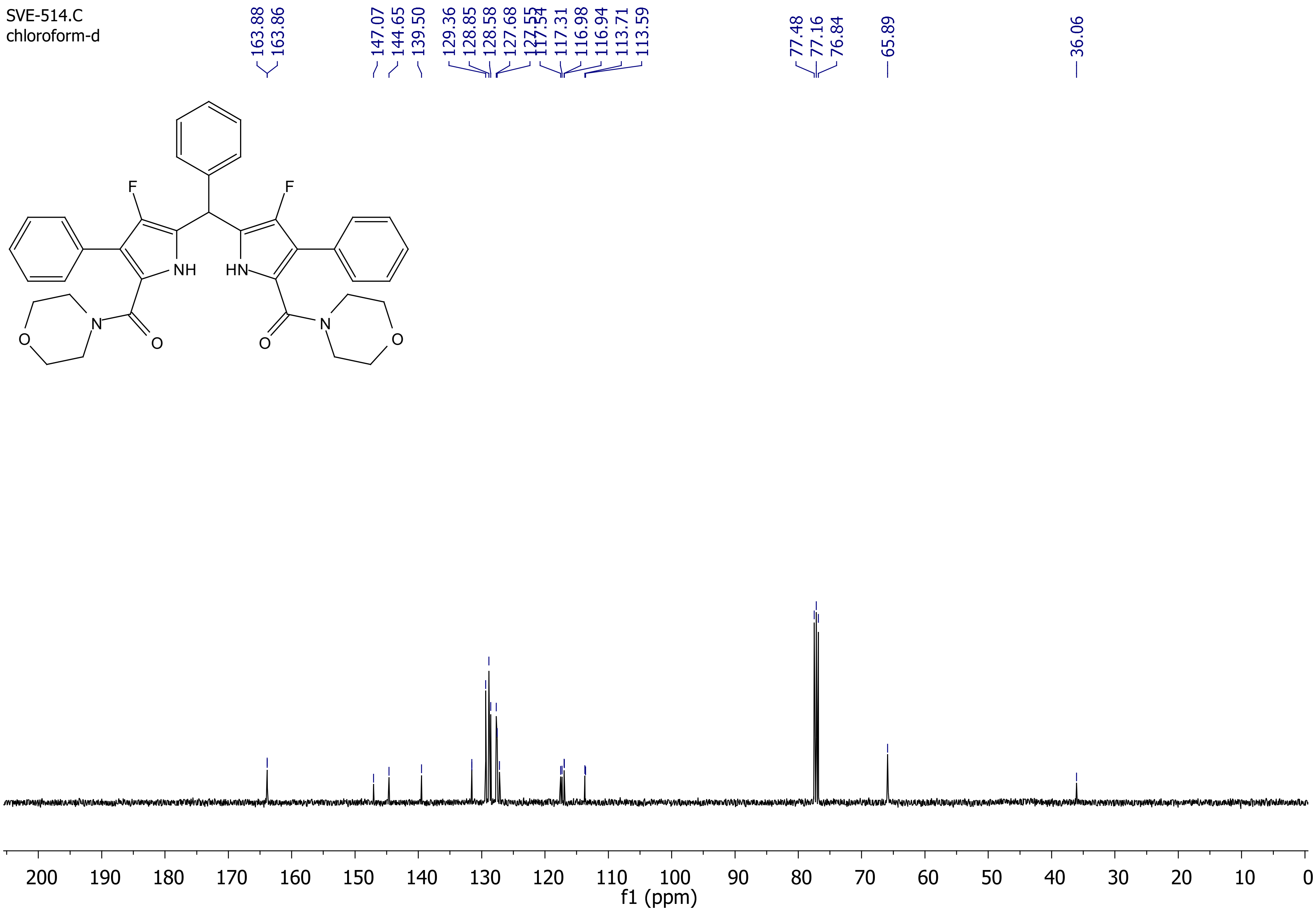

$^{13}C\{^{1}H\}$ NMR spectrum of (5,5'-(phenylmethylene)bis(4-fluoro-3-phenyl-1*H*-pyrrole-5,2-diyl))bis(morpholinomethanone) (**4f**) in $CDCl_3$ at 100 MHz

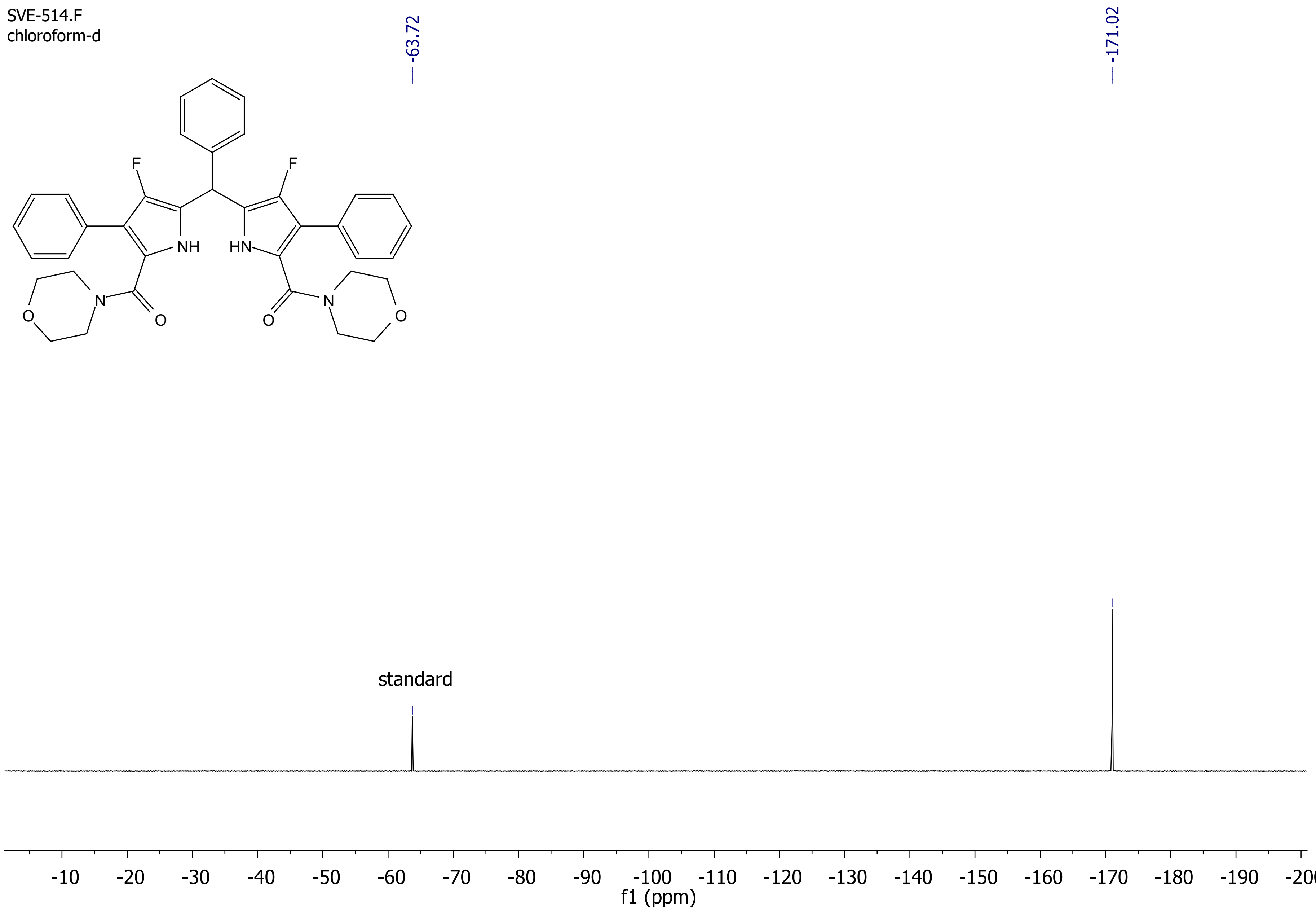

^{19}F NMR spectrum of (5,5'-(phenylmethylene)bis(4-fluoro-3-phenyl-1*H*-pyrrole-5,2-diyl))bis(morpholinomethanone) (**4f**) in $CDCl_3$ at 376 MHz

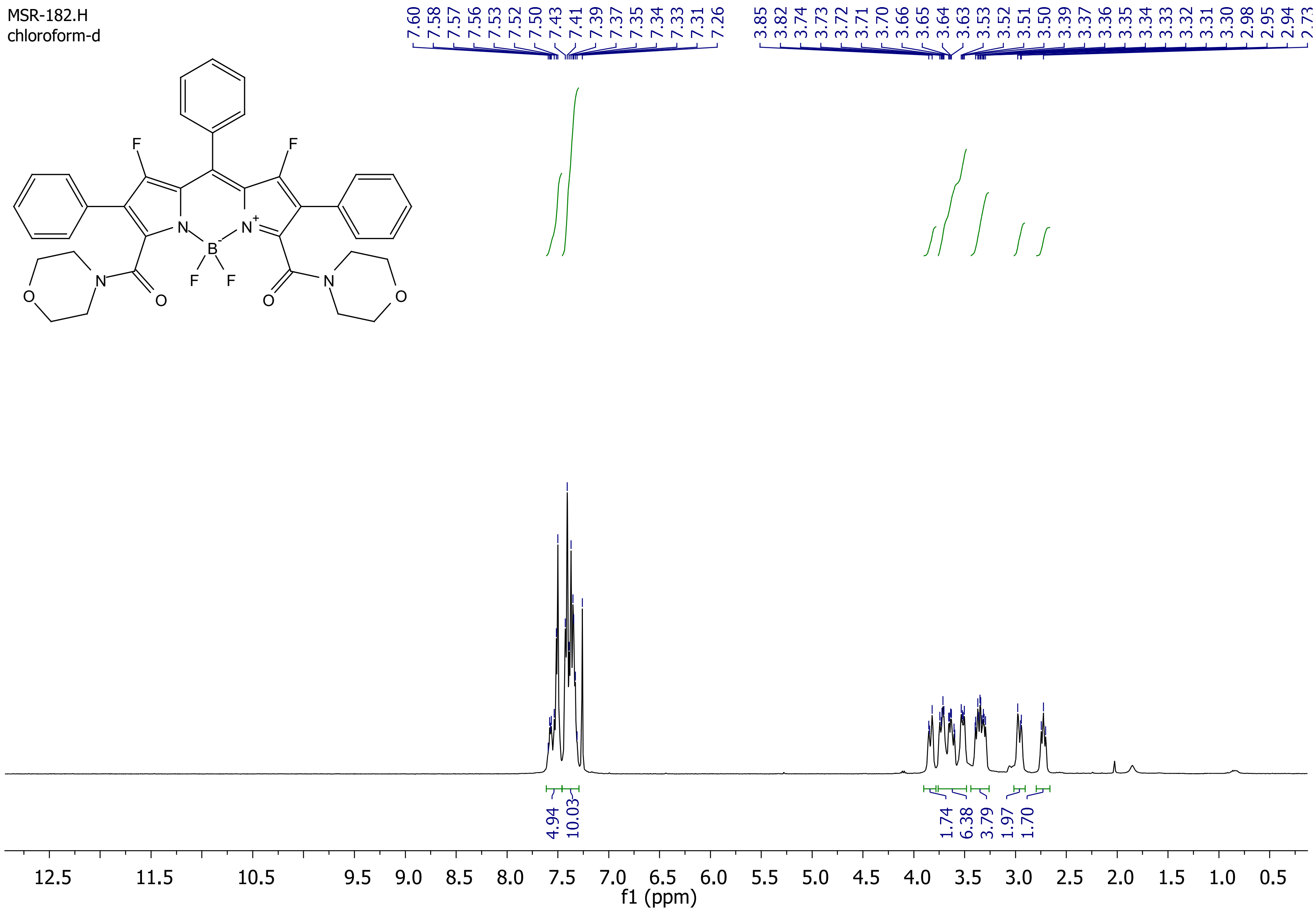

^{1}H NMR spectrum of 1,5,5,9-tetrafluoro-3,7-di(morpholine-4-carbonyl)-2,8,10-triphenyl-5*H*-dipyrrolo[1,2-c:2',1'-f][1,3,2]diazaborinin-4-ium-5-uide (**5f**) in $CDCl_3$ at 400 MHz

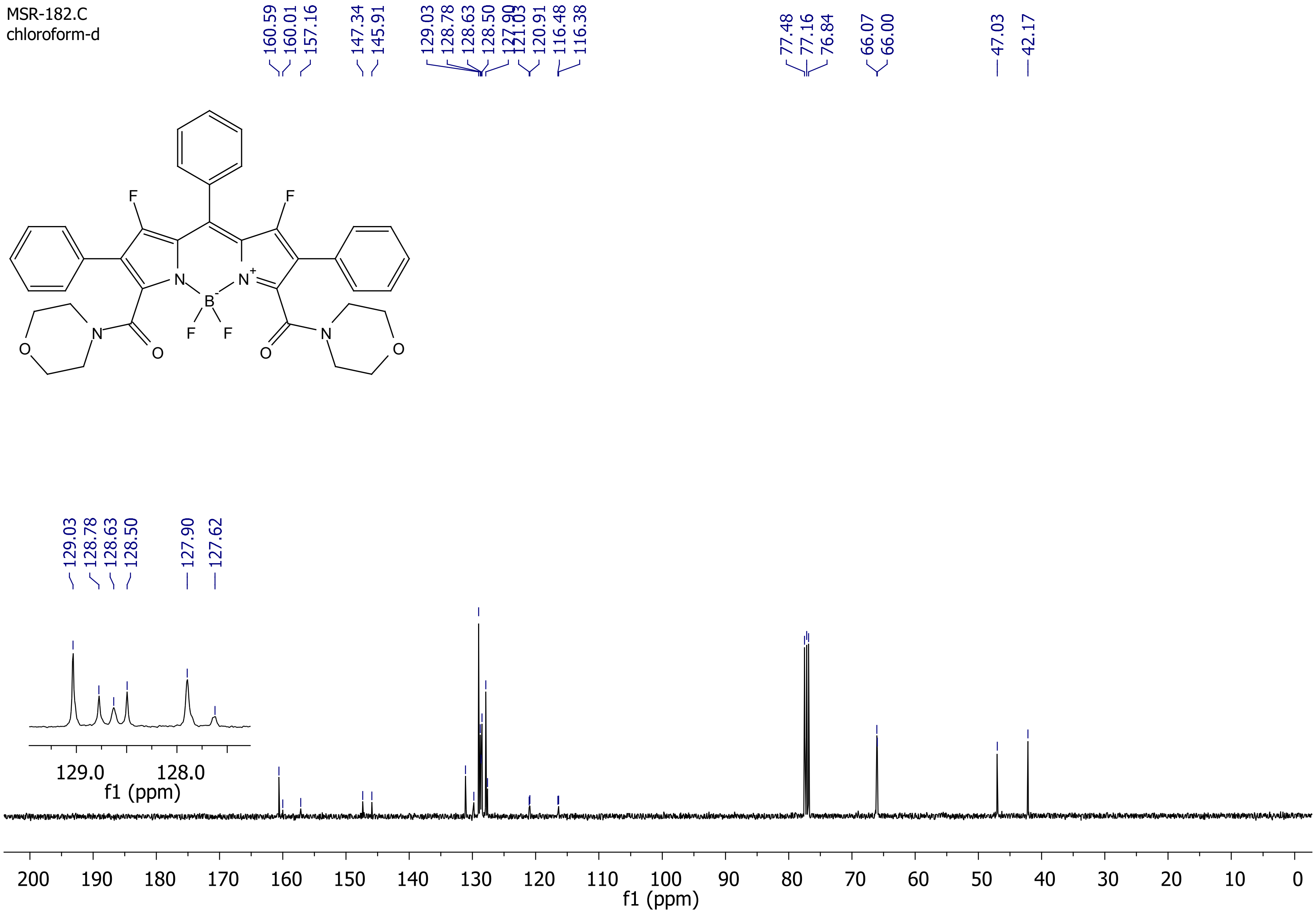

$^{13}C\{^{1}H\}$ NMR spectrum of 1,5,5,9-tetrafluoro-3,7-di(morpholine-4-carbonyl)-2,8,10-triphenyl-5*H*-dipyrrolo[1,2-c:2',1'-f][1,3,2]diazaborinin-4-ium-5-uide (**5f**) in $CDCl_3$ at 100 MHz

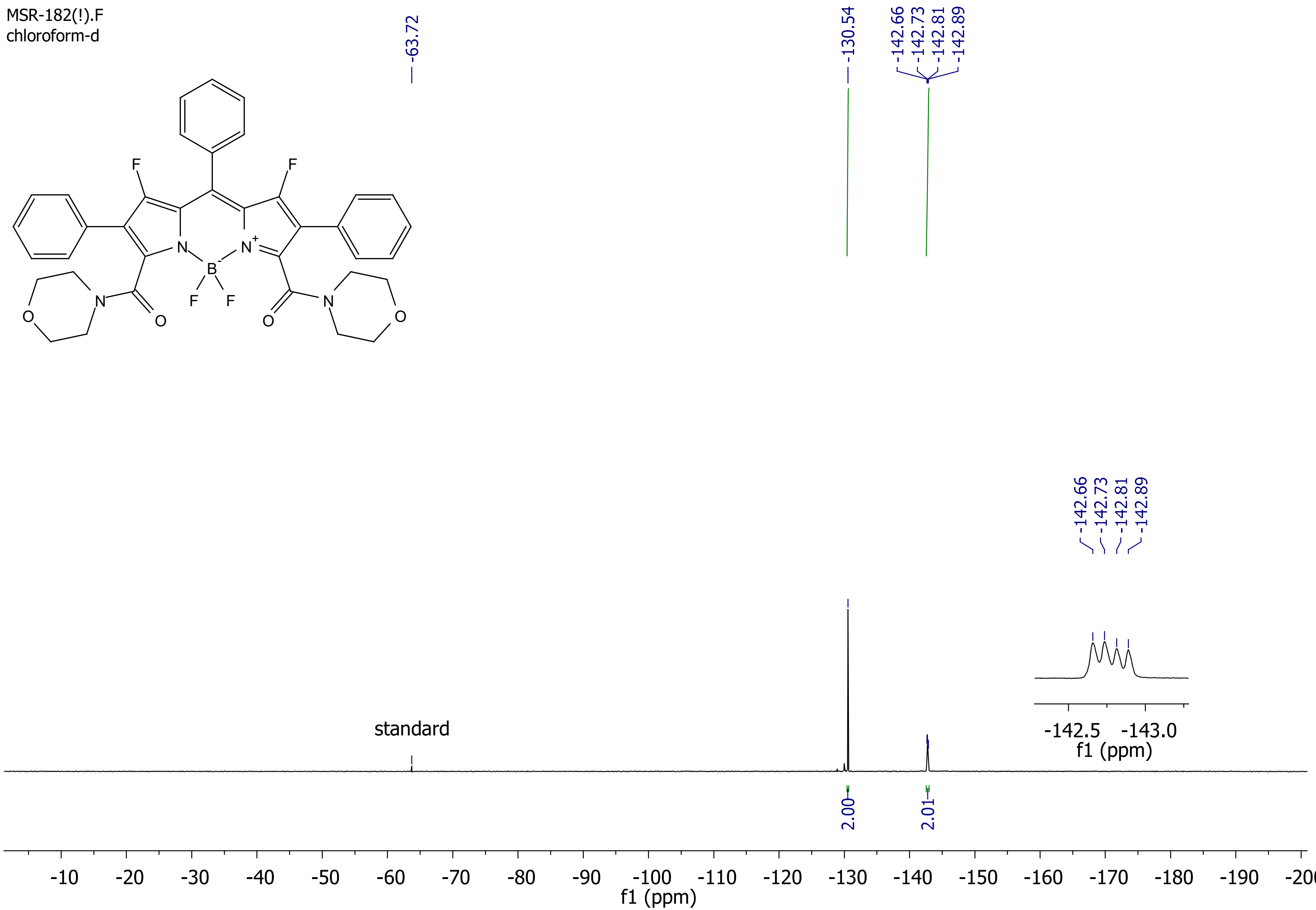

^{19}F NMR spectrum of 1,5,5,9-tetrafluoro-3,7-di(morpholine-4-carbonyl)-2,8,10-triphenyl-5*H*-dipyrrolo[1,2-c:2',1'-f][1,3,2]diazaborinin-4-ium-5-uide (**5f**) in $CDCl_3$ at 376 MHz

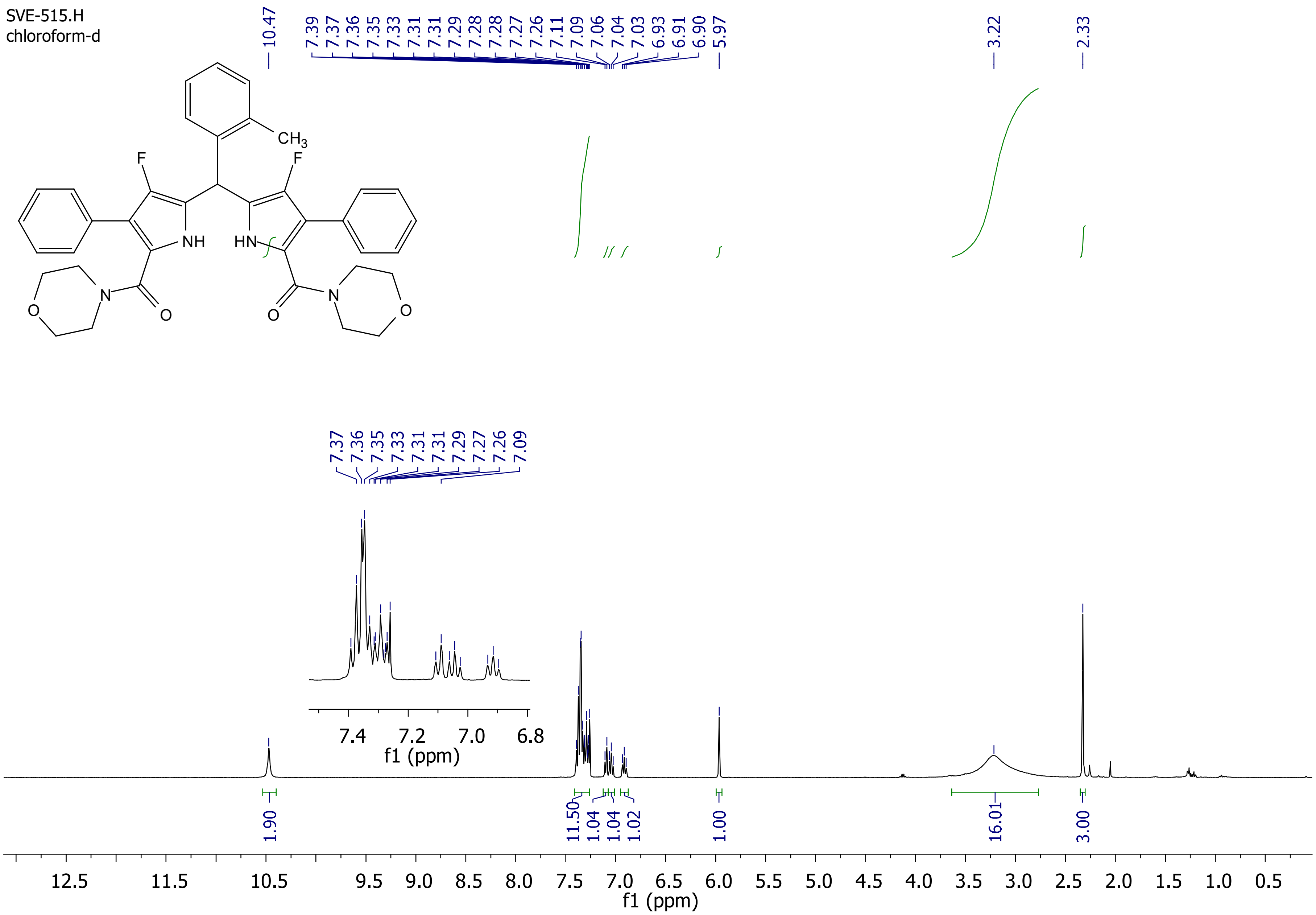

^{1}H NMR spectrum of (5,5'-(*o*-tolylmethylene)bis(4-fluoro-3-phenyl-1*H*-pyrrole-5,2-diyl))bis(morpholinomethanone) (**4g**) in $CDCl_3$ at 400 MHz

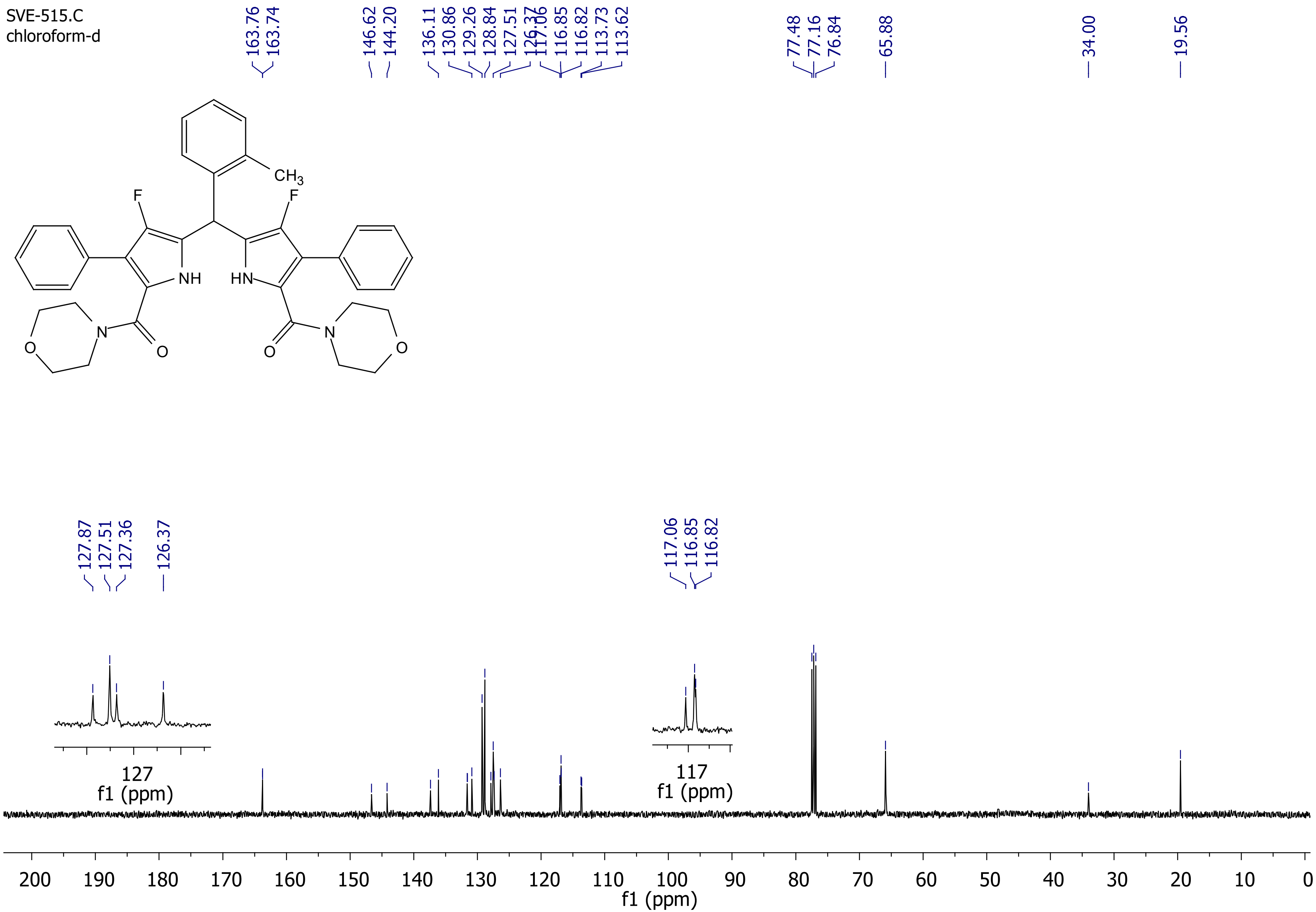

$^{13}C\{^1H\}$ NMR spectrum of (5,5'-(*o*-tolylmethylene)bis(4-fluoro-3-phenyl-1*H*-pyrrole-5,2-diyl))bis(morpholinomethanone) (**4g**) in $CDCl_3$ at 100 MHz

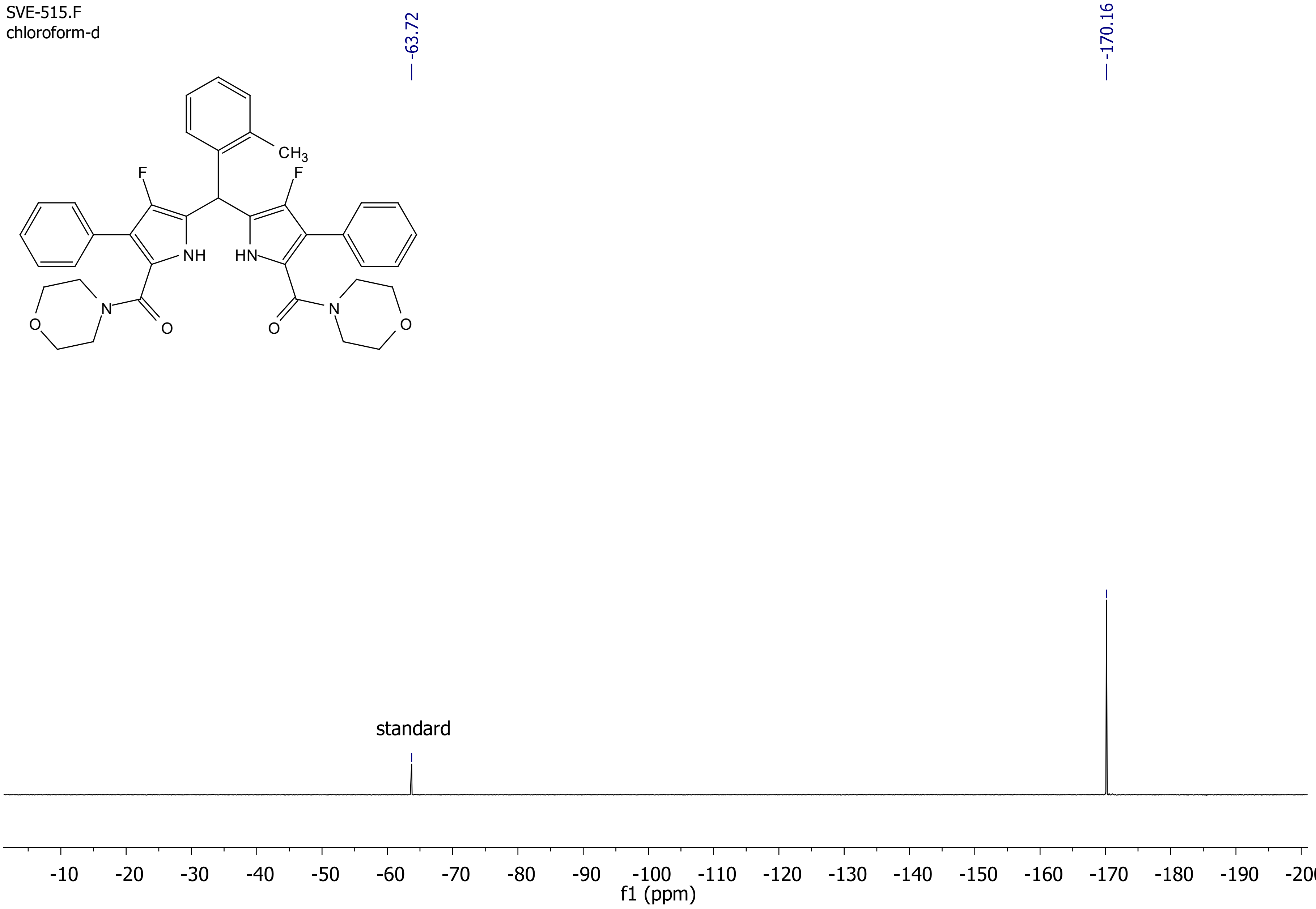

^{19}F NMR spectrum of (5,5'-(*o*-tolylmethylene)bis(4-fluoro-3-phenyl-1*H*-pyrrole-5,2-diyl))bis(morpholinomethanone) (**4g**) in $CDCl_3$ at 376 MHz

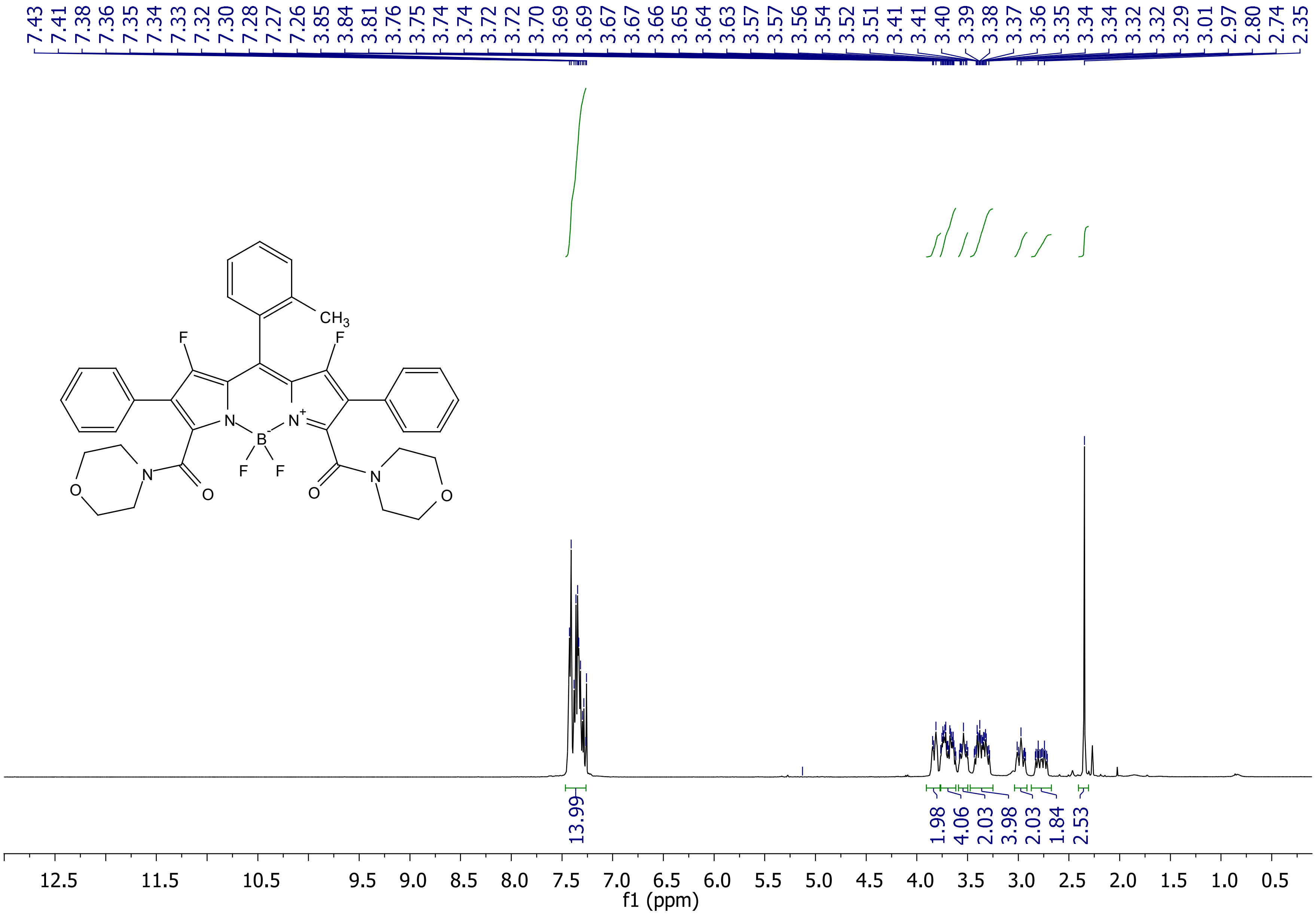

^{1}H NMR spectrum of 1,5,5,9-tetrafluoro-3,7-di(morpholine-4-carbonyl)-2,8-diphenyl-10-(*o*-tolyl)-5*H*-dipyrrolo[1,2-c:2',1'-f][1,3,2]diazaborinin-4-ium-5-uide (**5g**) in $CDCl_3$ at 400 MHz

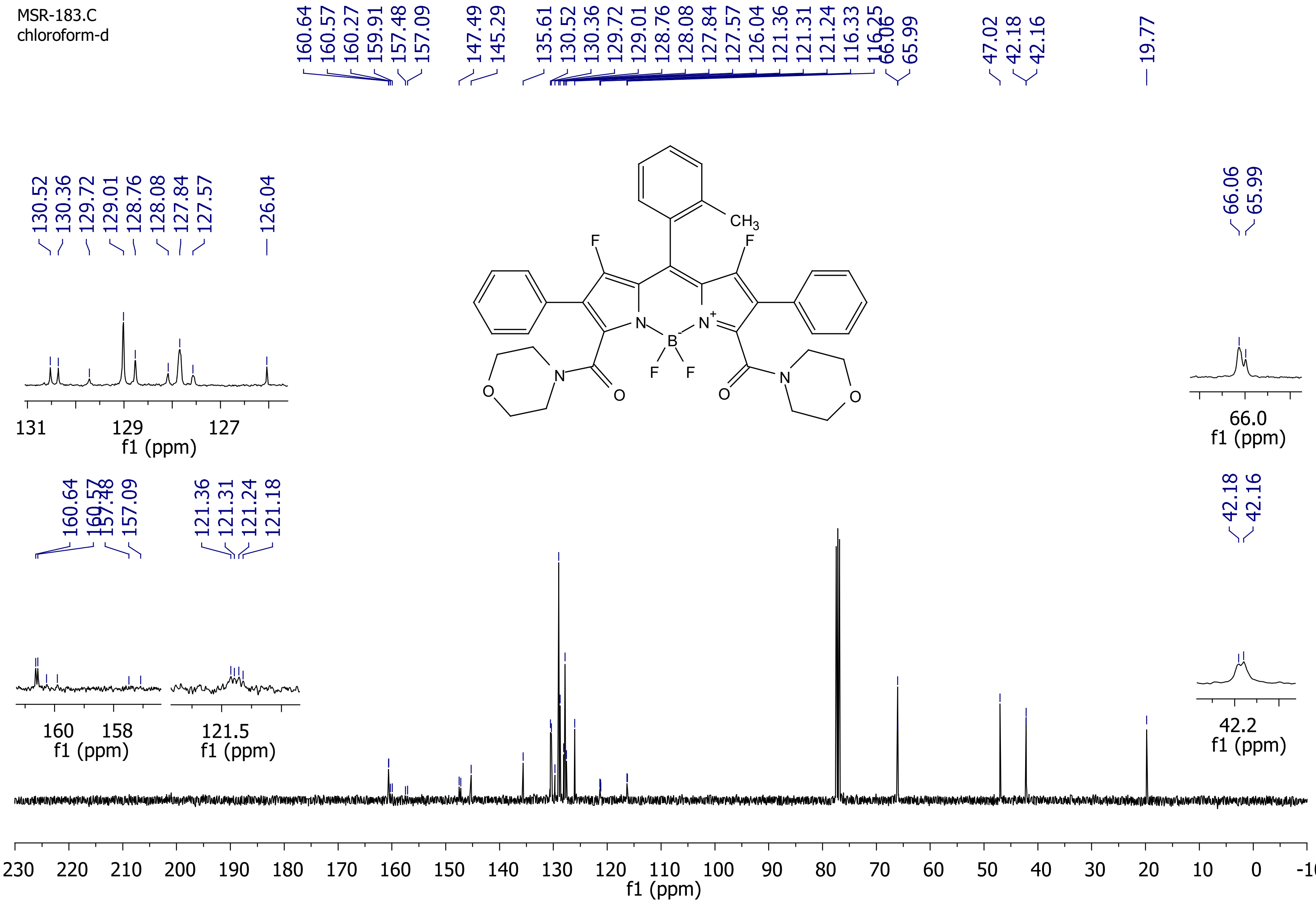

$^{13}C\{^{1}H\}$ NMR spectrum of 1,5,5,9-tetrafluoro-3,7-di(morpholine-4-carbonyl)-2,8-diphenyl-10-(*o*-tolyl)-5*H*-dipyrrolo[1,2-c:2',1'-f][1,3,2]diazaborinin-4-ium-5-uide (**5g**) in $CDCl_3$ at 100 MHz

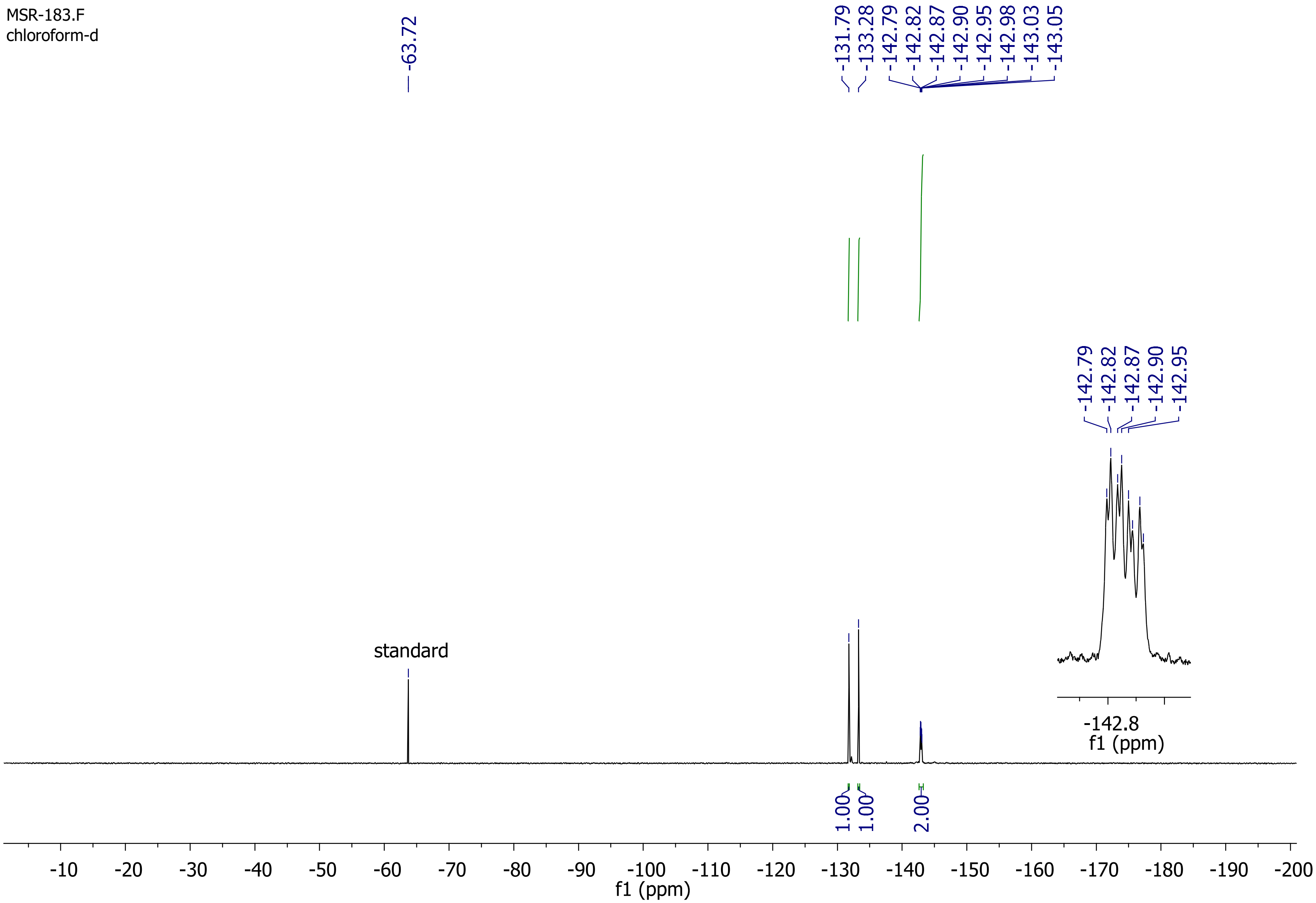

^{19}F NMR spectrum of 1,5,5,9-tetrafluoro-3,7-di(morpholine-4-carbonyl)-2,8-diphenyl-10-(*o*-tolyl)-5*H*-dipyrrolo[1,2-c:2',1'-f][1,3,2]diazaborinin-4-ium-5-uide (**5g**) in $CDCl_3$ at 376 MHz

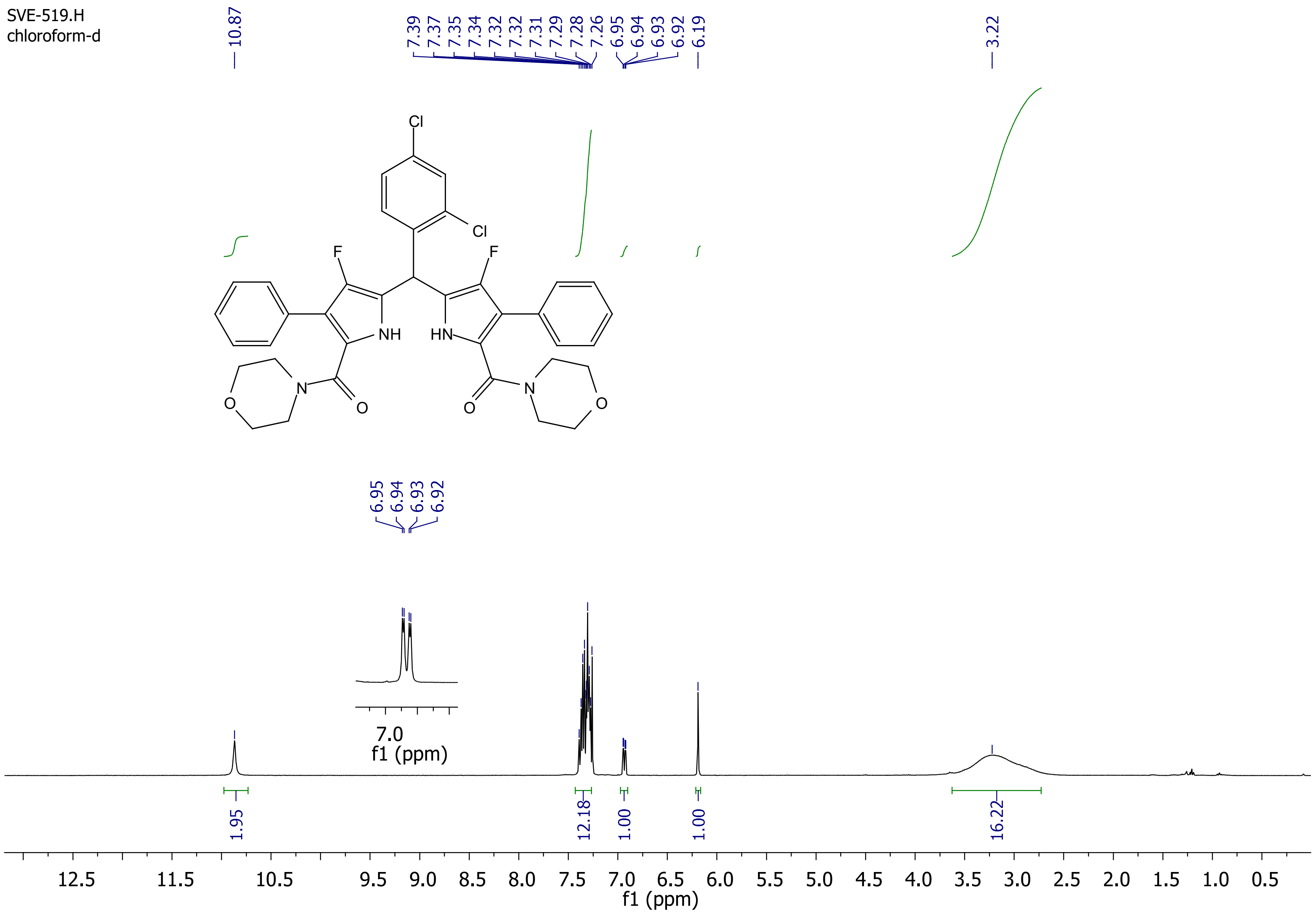

^{1}H NMR spectrum of (5,5'-((2,4-dichlorophenyl)methylene)bis(4-fluoro-3-phenyl-1*H*-pyrrole-5,2-diyl))bis(morpholinomethanone) (**4h**) in $CDCl_3$ at 400 MHz

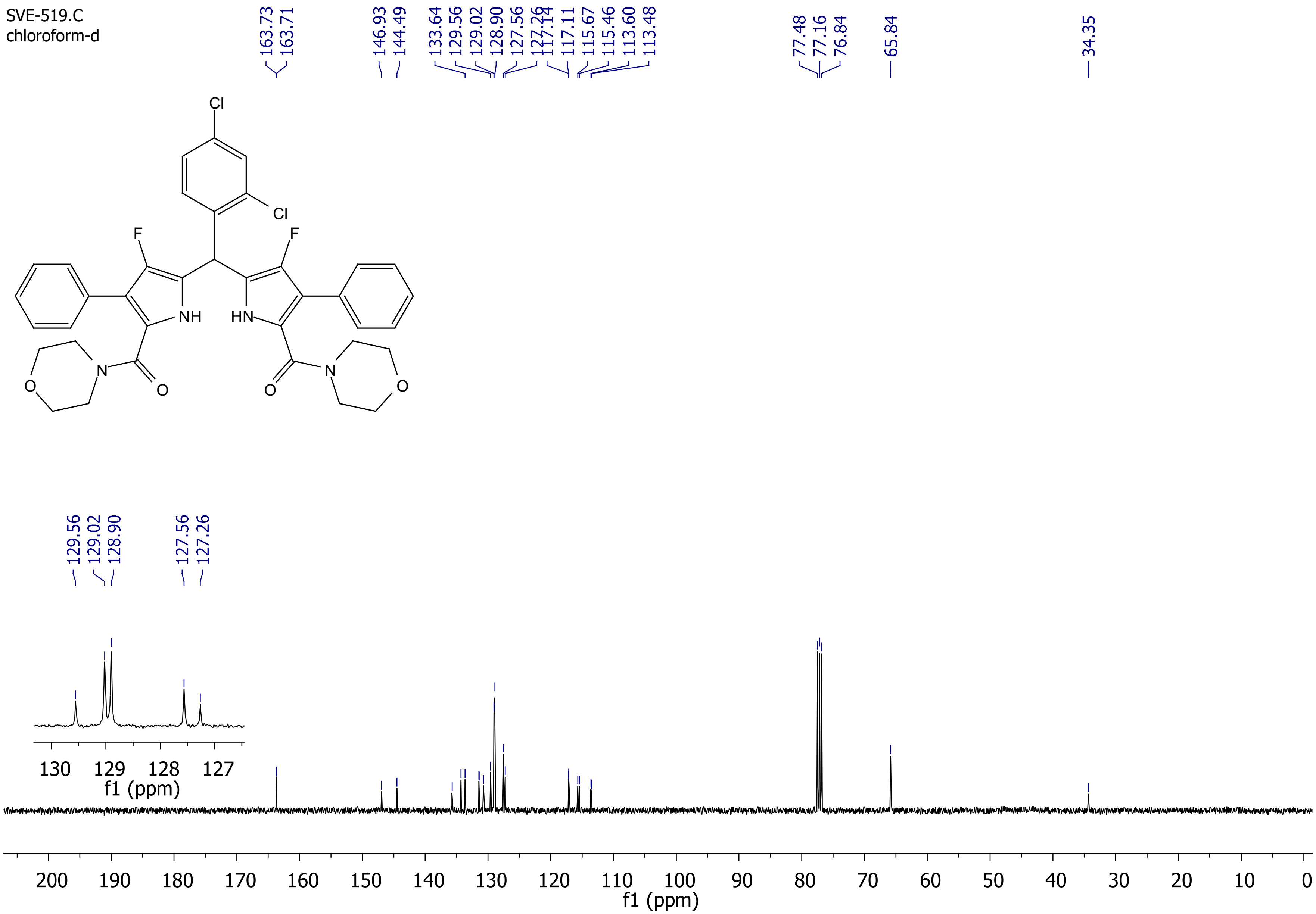

$^{13}C\{^1H\}$ NMR spectrum of (5,5'-((2,4-dichlorophenyl)methylene)bis(4-fluoro-3-phenyl-1*H*-pyrrole-5,2-diyl))bis(morpholinomethanone) (**4h**) in $CDCl_3$ at 100 MHz

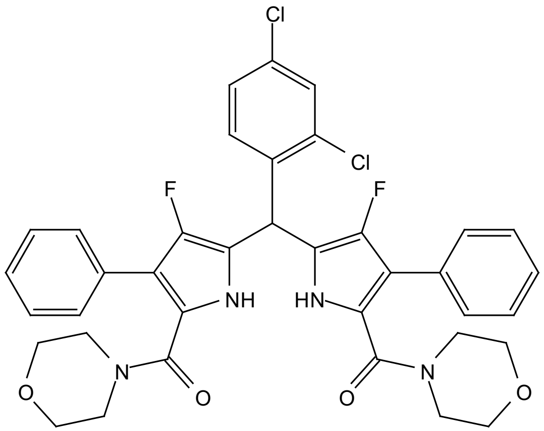

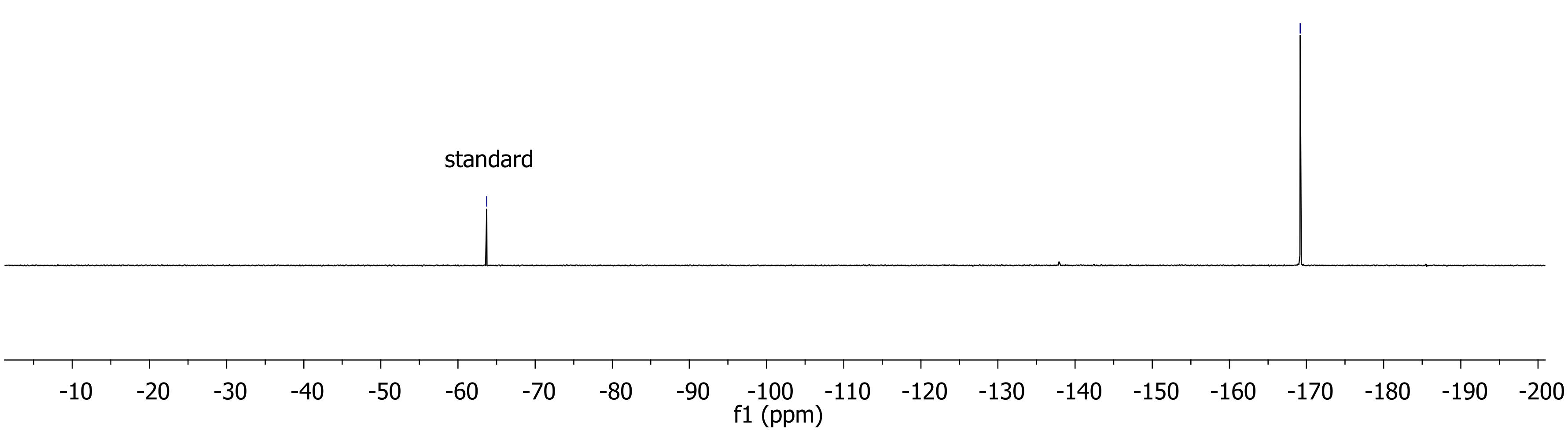

^{19}F NMR spectrum of (5,5'-((2,4-dichlorophenyl)methylene)bis(4-fluoro-3-phenyl-1*H*-pyrrole-5,2-diyl))bis(morpholinomethanone) (**4h**) in $CDCl_3$ at 376 MHz

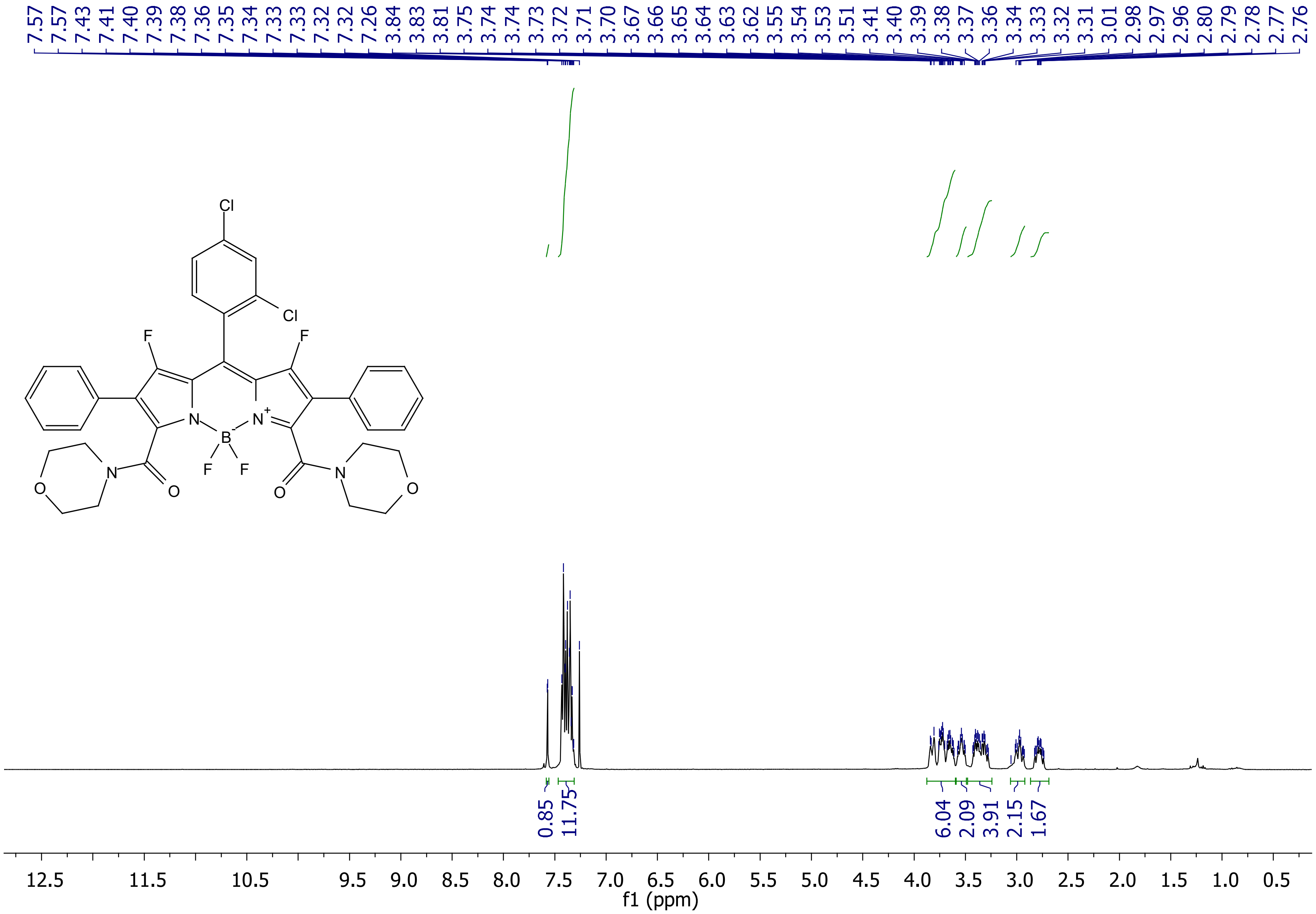

^{1}H NMR spectrum of 10-(2,4-dichlorophenyl)-1,5,5,9-tetrafluoro-3,7-di(morpholine-4-carbonyl)-2,8-diphenyl-5*H*-dipyrrolo[1,2-c:2',1'-f][1,3,2]diazaborinin-4-ium-5-uide (**5h**) in $CDCl_3$ at 400 MHz

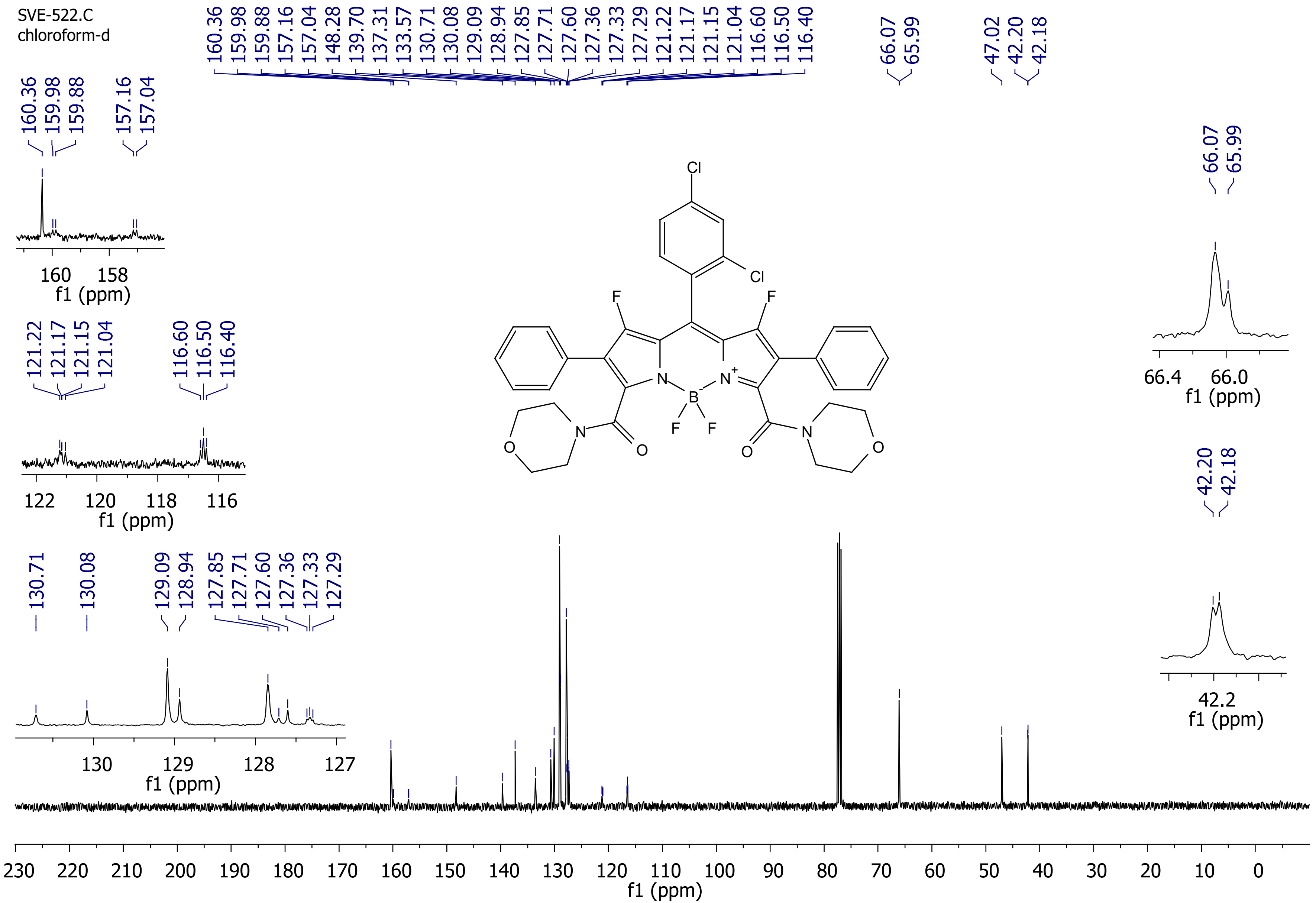

$^{13}C\{^1H\}$ NMR spectrum of 10-(2,4-dichlorophenyl)-1,5,5,9-tetrafluoro-3,7-di(morpholine-4-carbonyl)-2,8-diphenyl-5*H*-dipyrrolo[1,2-c:2',1'-f][1,3,2]diazaborinin-4-ium-5-uide (**5h**) in $CDCl_3$ at 100 MHz

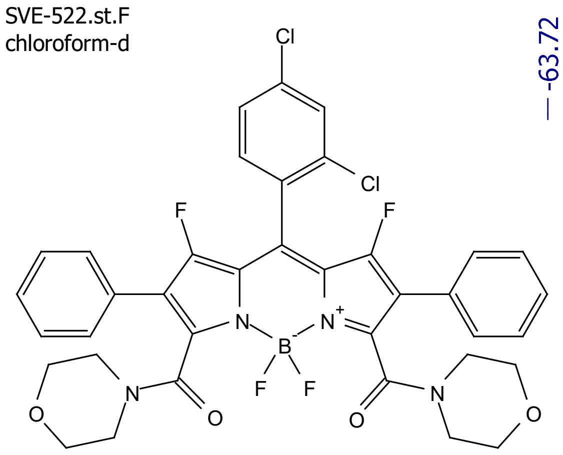

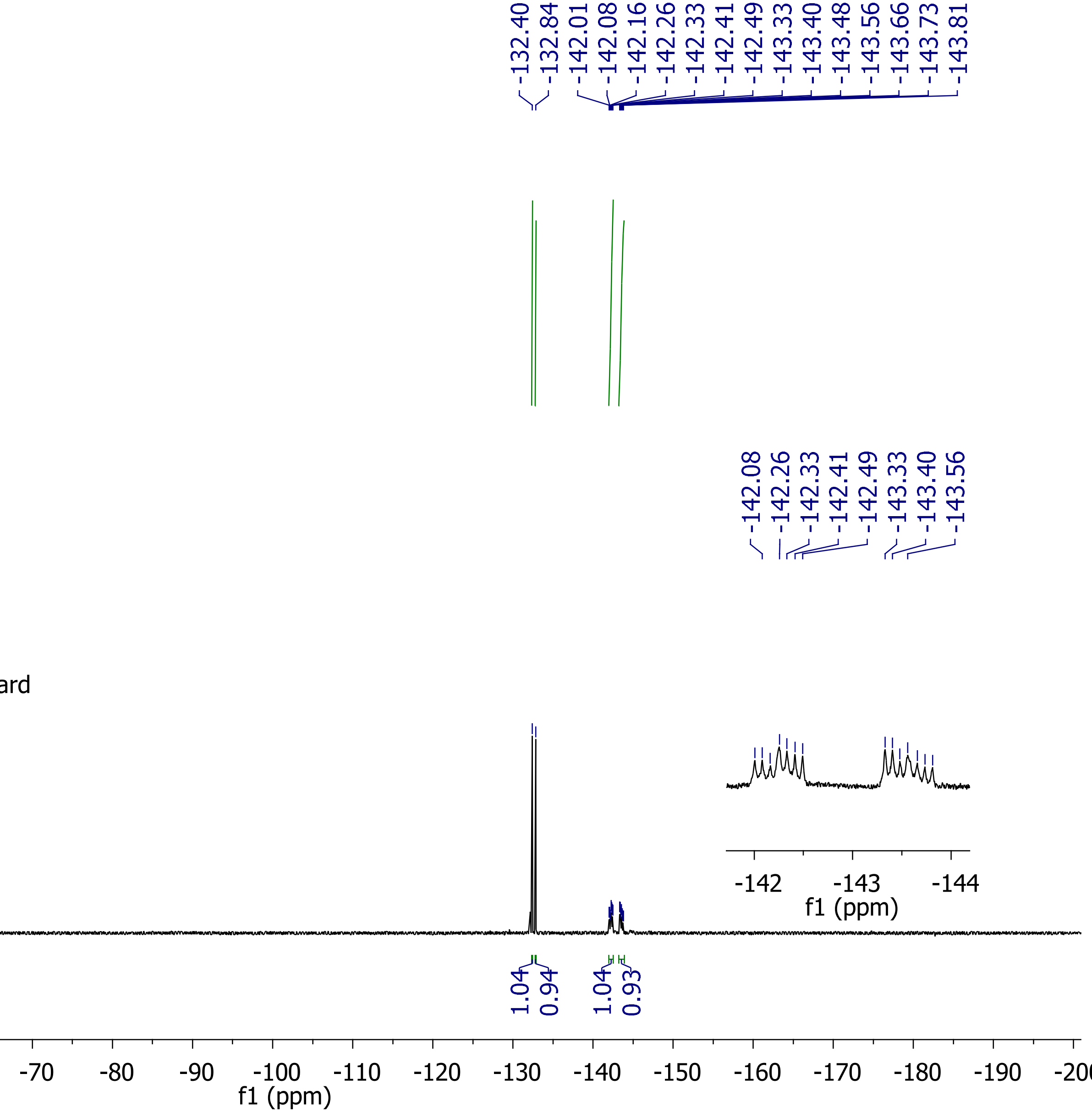

^{19}F NMR spectrum of 10-(2,4-dichlorophenyl)-1,5,5,9-tetrafluoro-3,7-di(morpholine-4-carbonyl)-2,8-diphenyl-5*H*-dipyrrolo[1,2-c:2',1'-f][1,3,2]diazaborinin-4-ium-5-uide (**5h**) in $CDCl_3$ at 376 MHz

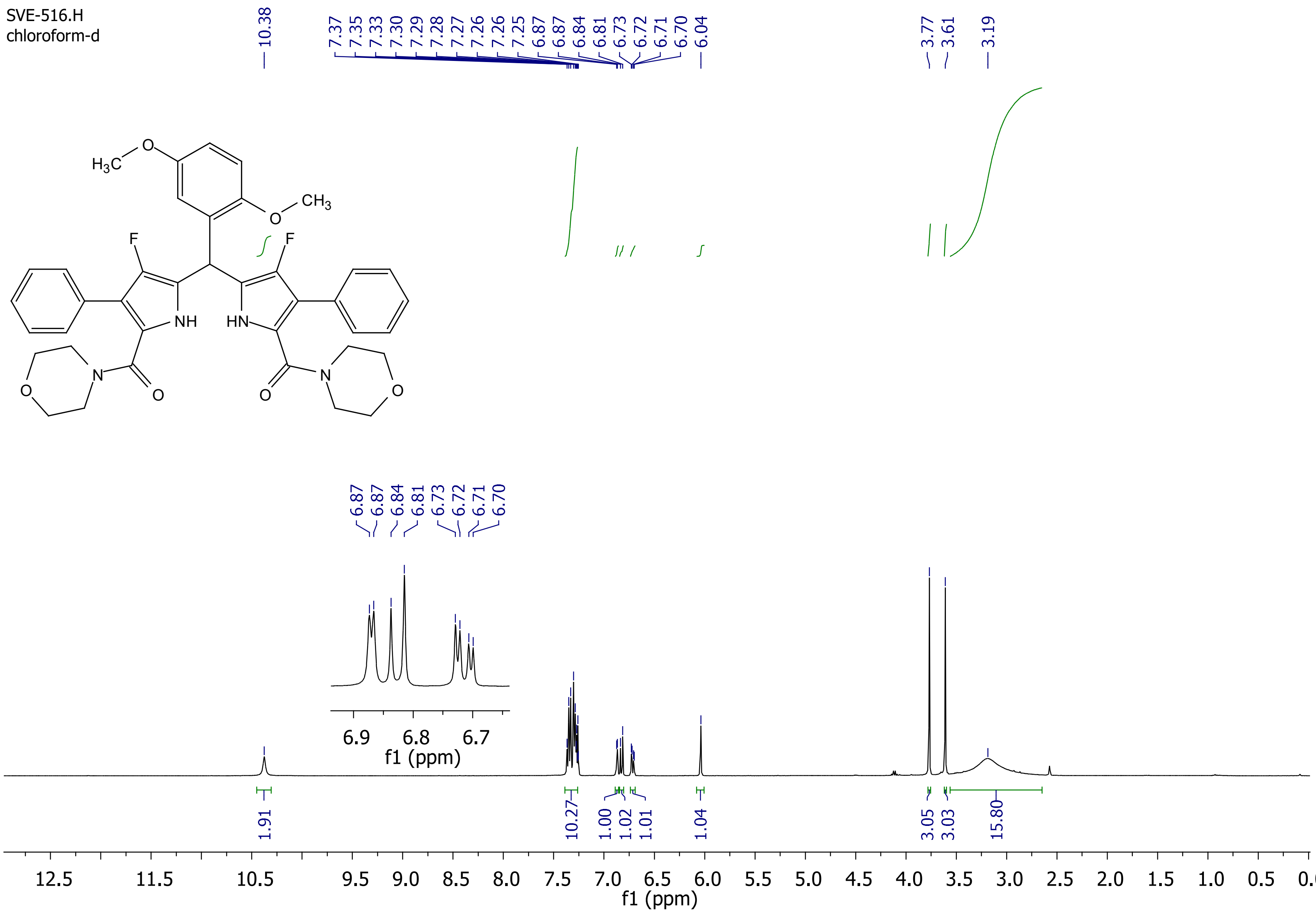

^{1}H NMR spectrum of (5,5'-((2,5-dimethoxyphenyl)methylene)bis(4-fluoro-3-phenyl-1*H*-pyrrole-5,2-diyl))bis(morpholinomethanone) (**4i**) in $CDCl_3$ at 400 MHz

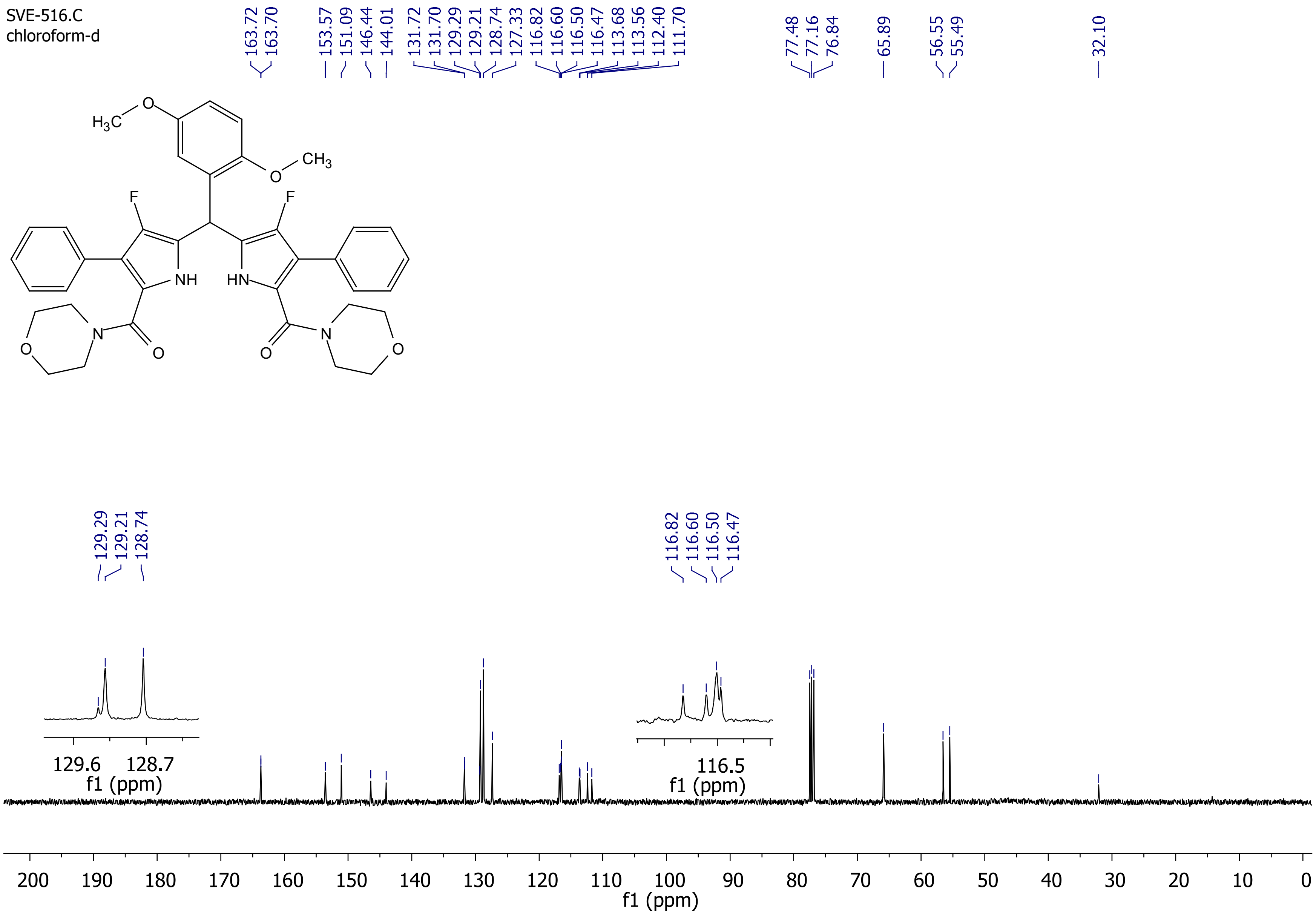

$^{13}C\{^1H\}$ NMR spectrum of (5,5'-((2,5-dimethoxyphenyl)methylene)bis(4-fluoro-3-phenyl-1*H*-pyrrole-5,2-diyl))bis(morpholinomethanone) (**4i**) in $CDCl_3$ at 100 MHz

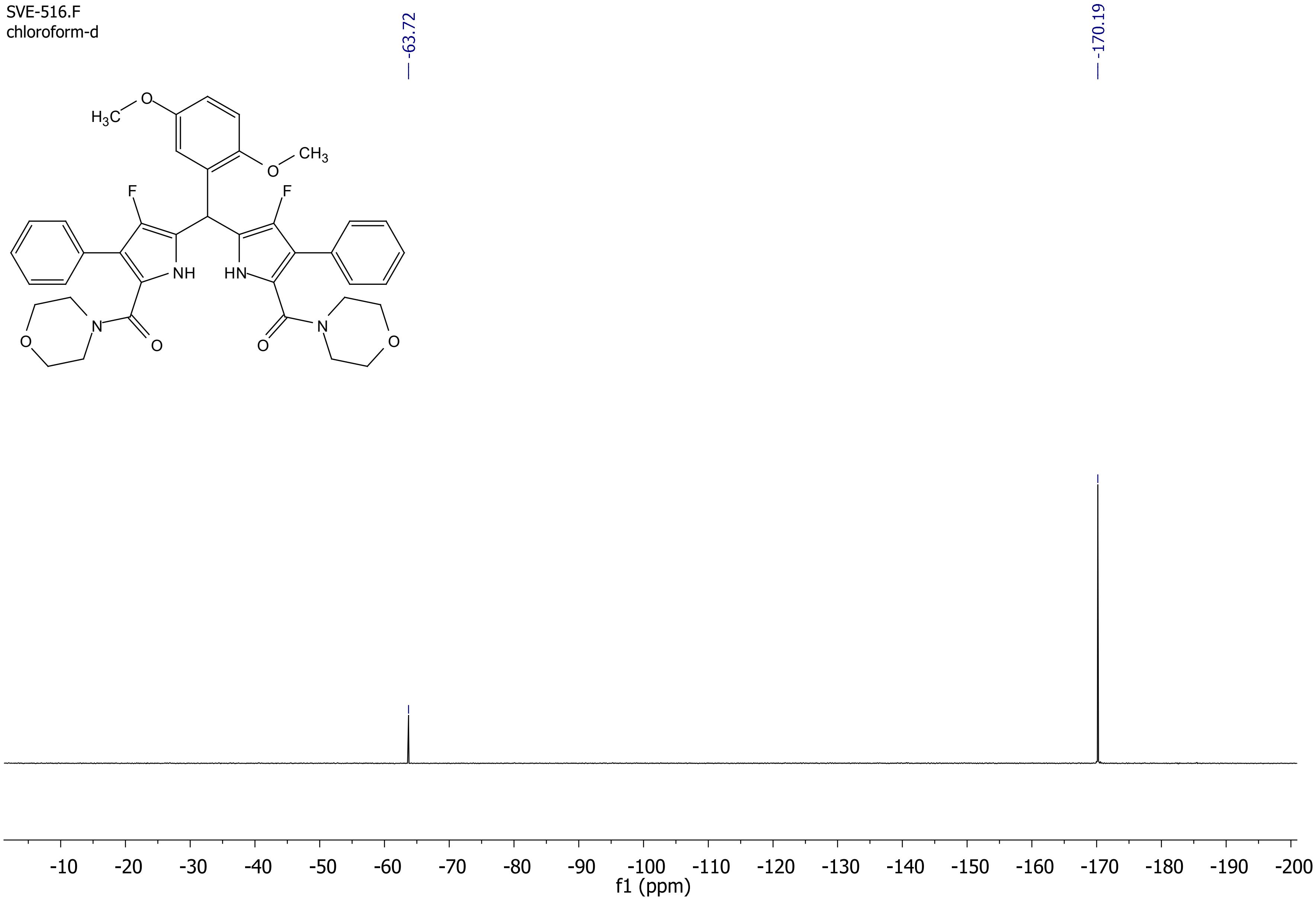

^{19}F NMR spectrum of (5,5'-((2,5-dimethoxyphenyl)methylene)bis(4-fluoro-3-phenyl-1*H*-pyrrole-5,2-diyl))bis(morpholinomethanone) (**4i**) in $CDCl_3$ at 376 MHz

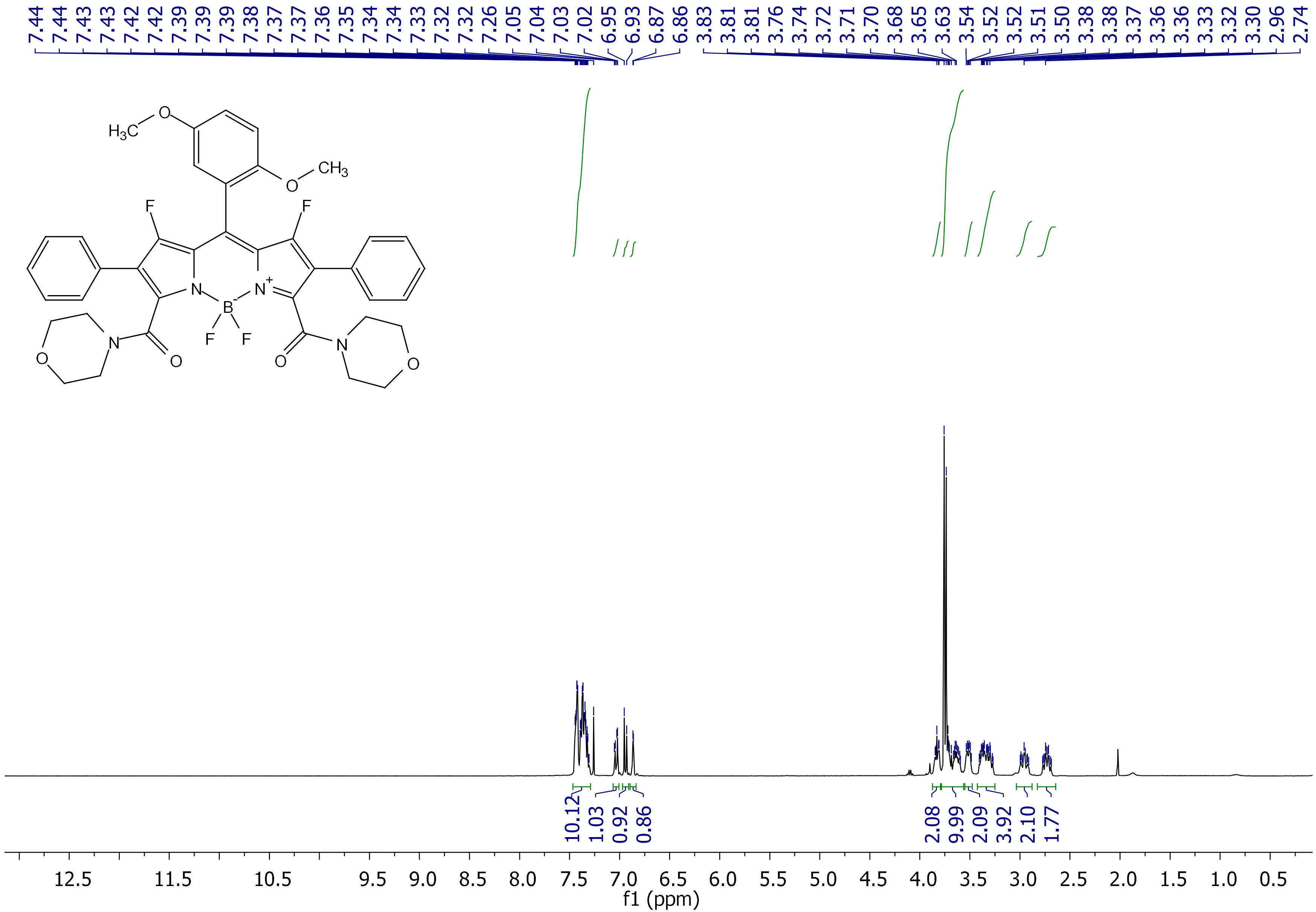

^{1}H NMR spectrum of 10-(2,5-dimethoxyphenyl)-1,5,5,9-tetrafluoro-3,7-di(morpholine-4-carbonyl)-2,8-diphenyl-5*H*-dipyrrolo[1,2-c:2',1'-f][1,3,2]diazaborinin-4-ium-5-uide (**5i**) in $CDCl_3$ at 400 MHz

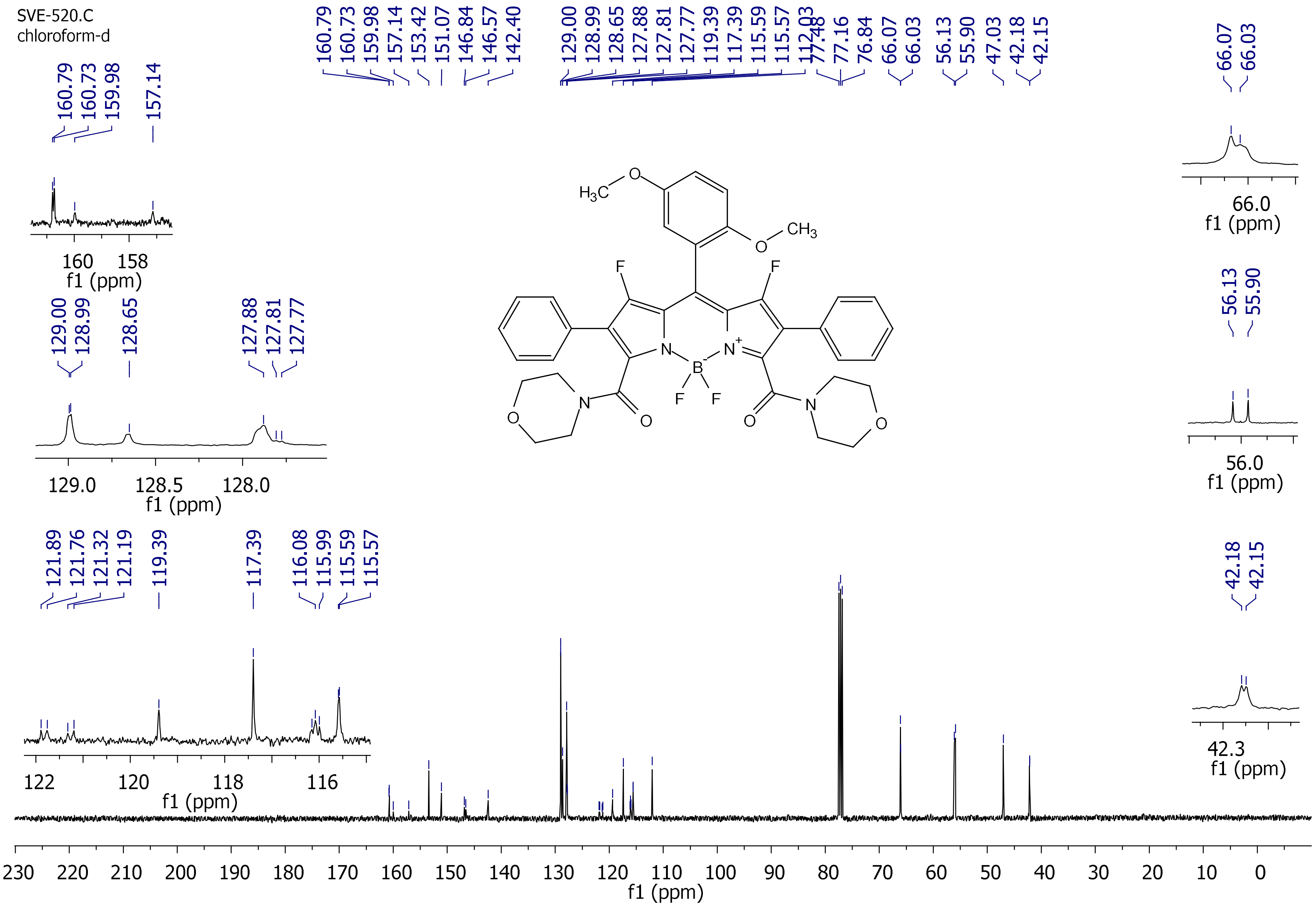

$^{13}C\{^{1}H\}$ NMR spectrum of 10-(2,5-dimethoxyphenyl)-1,5,5,9-tetrafluoro-3,7-di(morpholine-4-carbonyl)-2,8-diphenyl-5*H*-dipyrrolo[1,2-c:2',1'-f][1,3,2]diazaborinin-4-ium-5-uide (**5i**) in $CDCl_3$ at 100 MHz

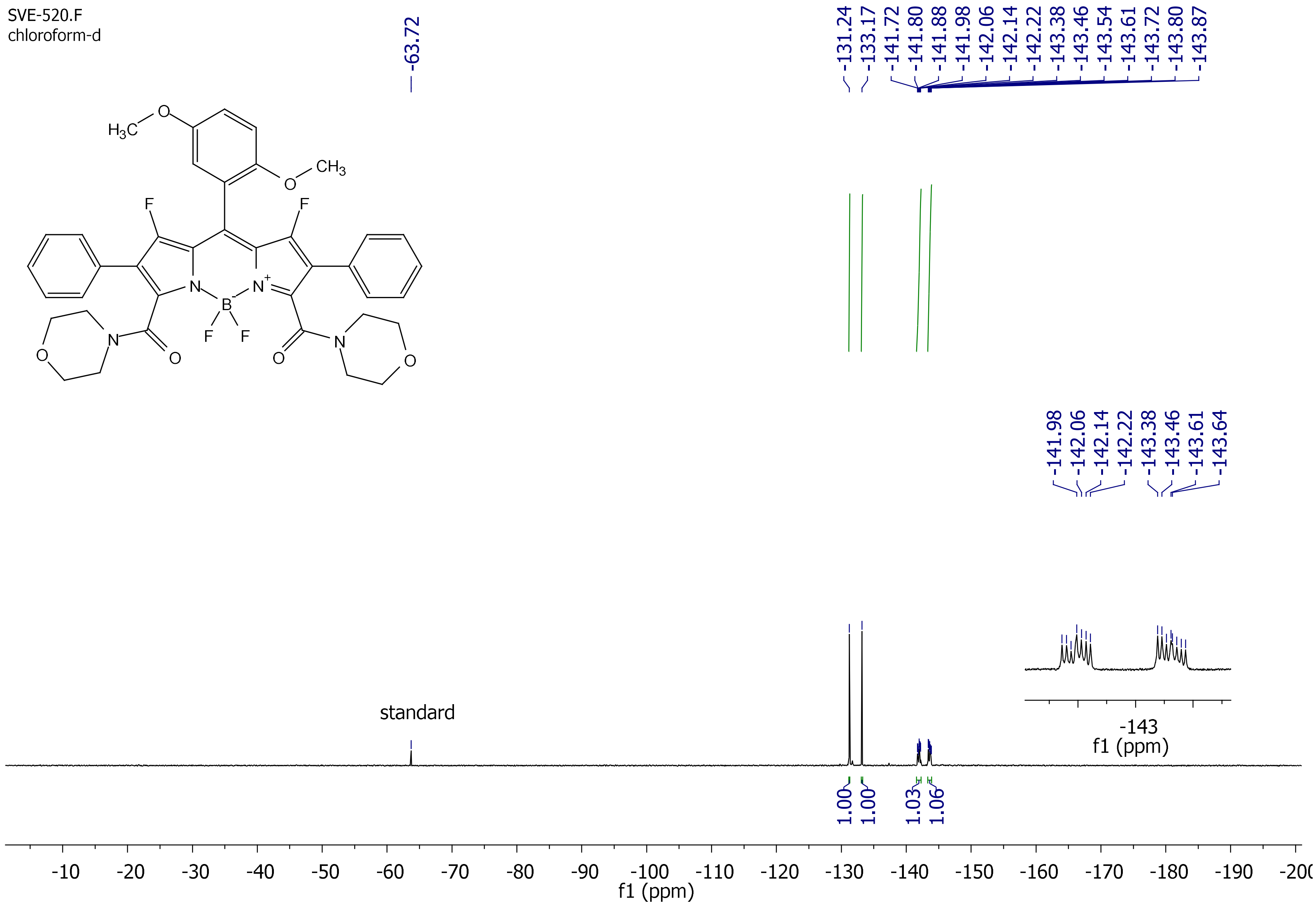

^{19}F NMR spectrum of 10-(2,5-dimethoxyphenyl)-1,5,5,9-tetrafluoro-3,7-di(morpholine-4-carbonyl)-2,8-diphenyl-5*H*-dipyrrolo[1,2-c:2',1'-f][1,3,2]diazaborinin-4-ium-5-uide (**5i**) in $CDCl_3$ at 376 MHz

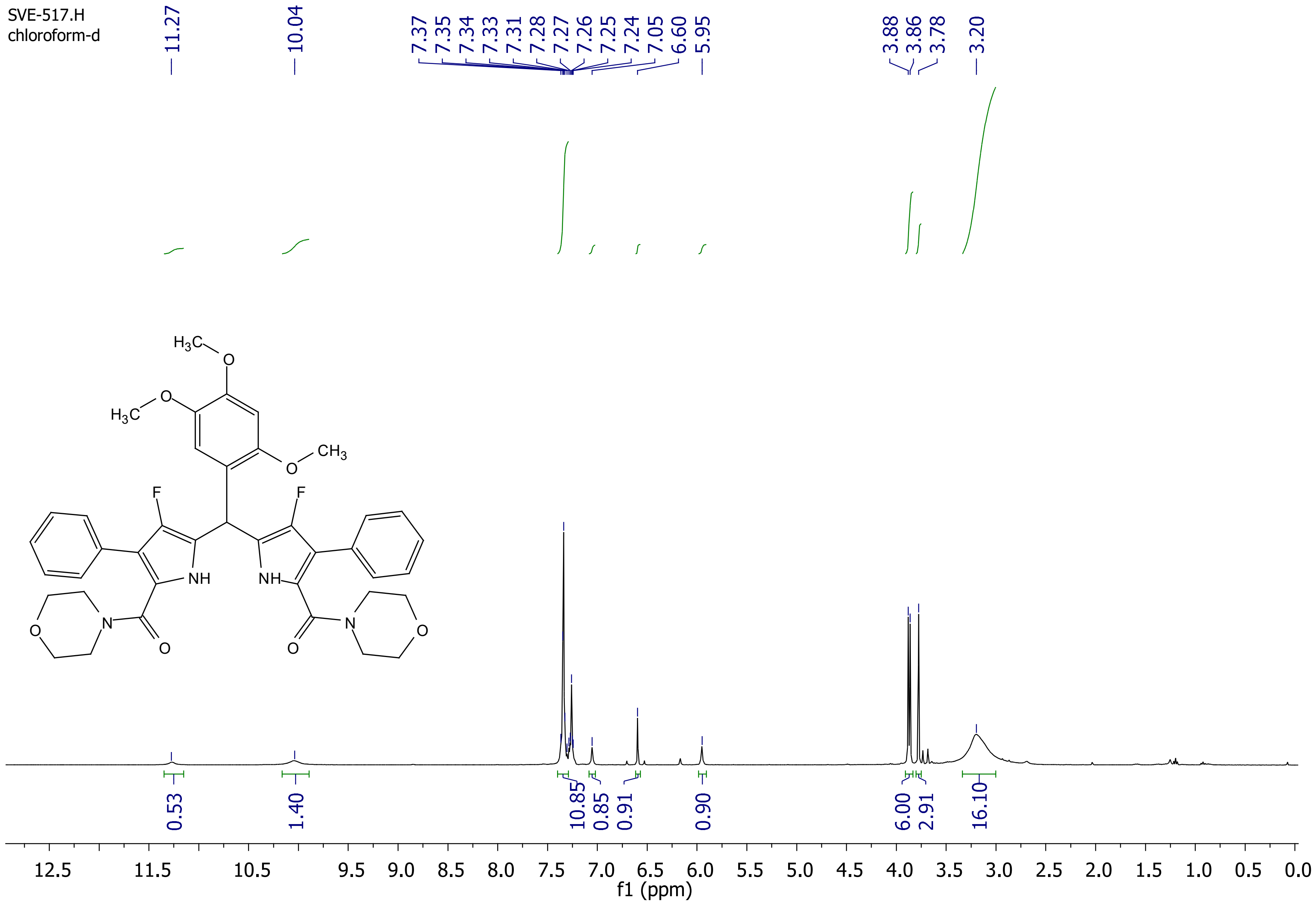

^{1}H NMR spectrum of (5,5'-((2,4,5-trimethoxyphenyl)methylene)bis(4-fluoro-3-phenyl-1*H*-pyrrole-5,2-diyl))bis(morpholinomethanone) (**4j**) in $CDCl_3$ at 400 MHz

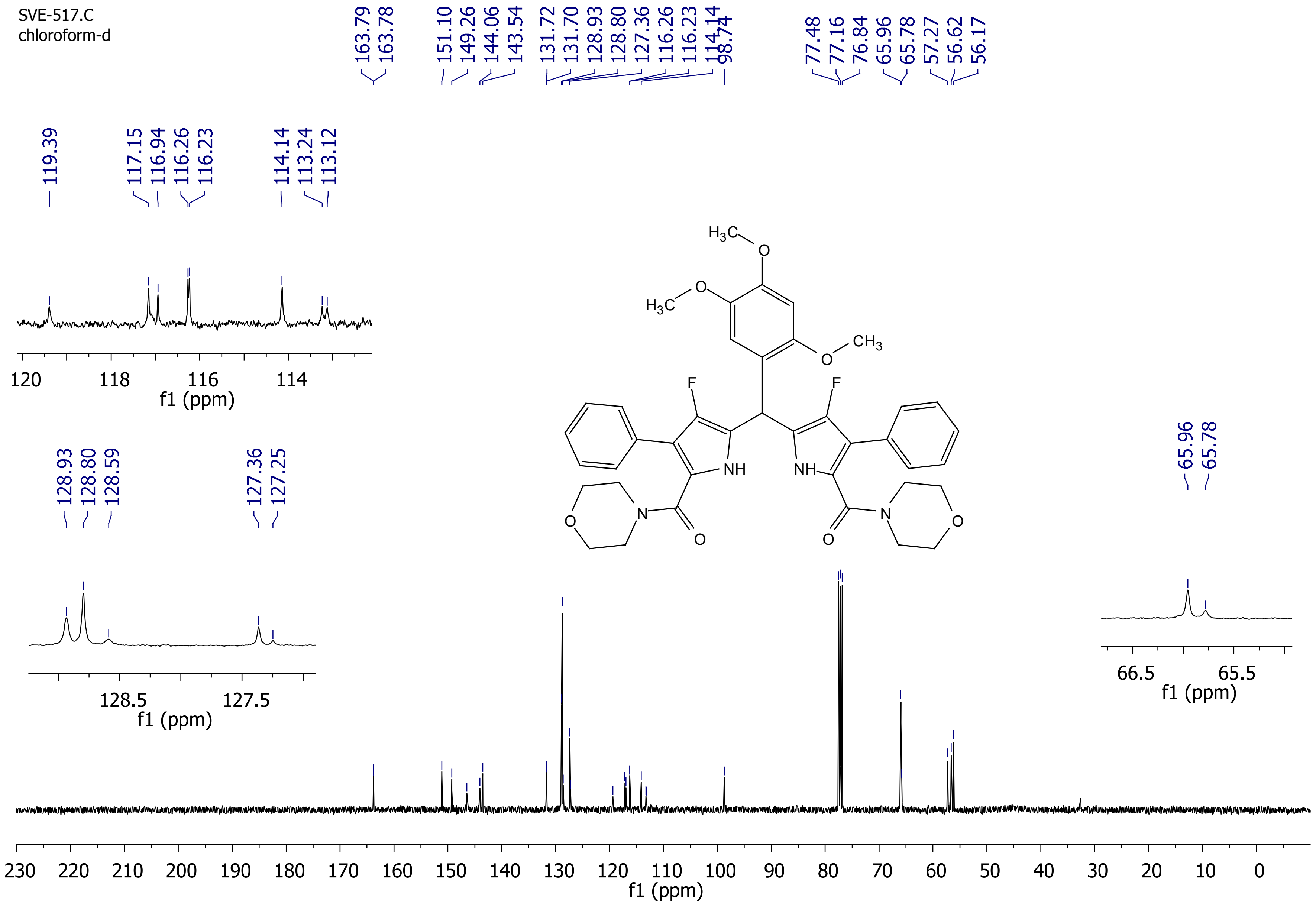

$^{13}C\{^1H\}$ NMR spectrum of (5,5'-((2,4,5-trimethoxyphenyl)methylene)bis(4-fluoro-3-phenyl-1*H*-pyrrole-5,2-diyl))bis(morpholinomethanone) (**4j**) in $CDCl_3$ at 100 MHz

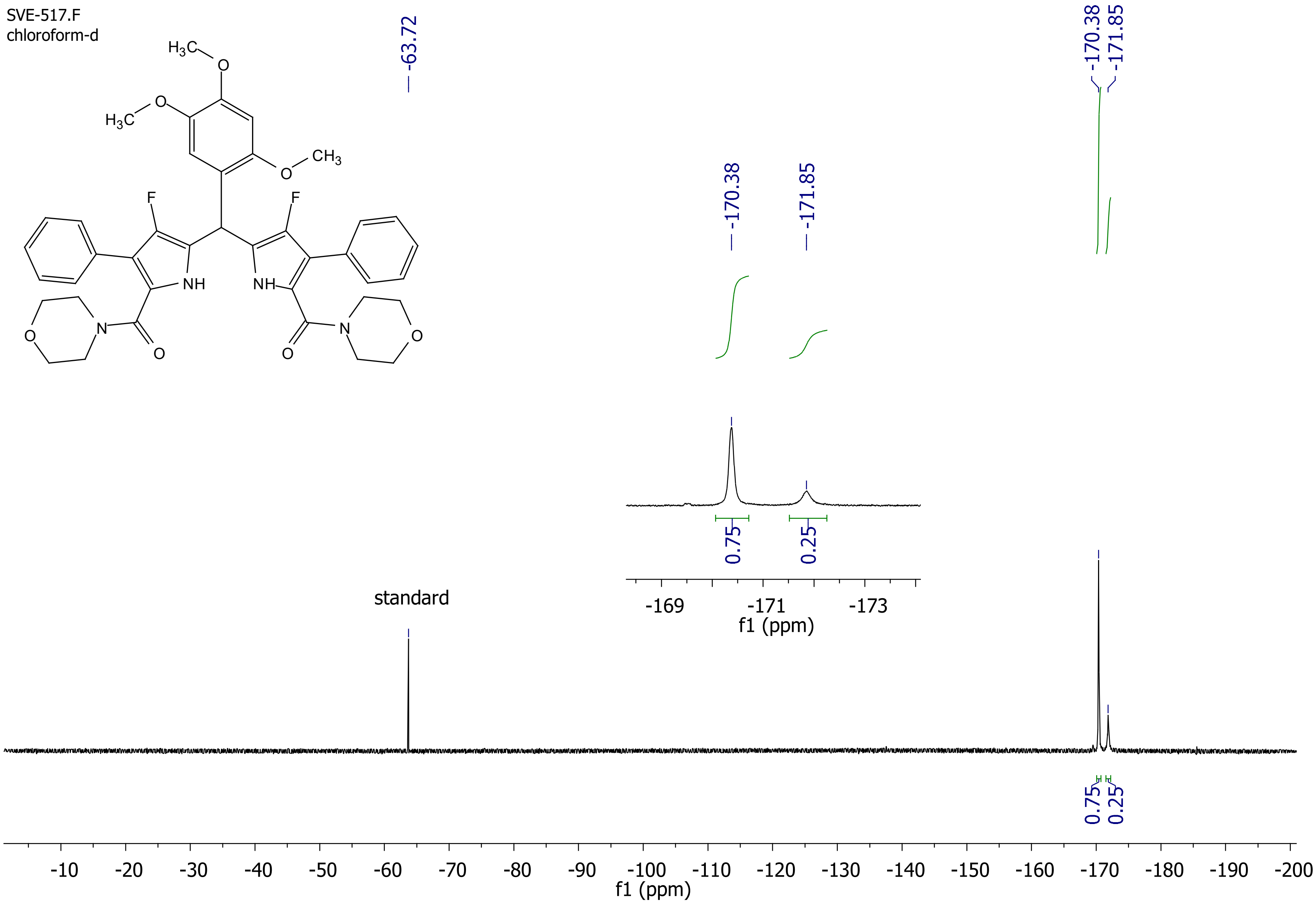

^{19}F NMR spectrum of (5,5'-((2,4,5-trimethoxyphenyl)methylene)bis(4-fluoro-3-phenyl-1*H*-pyrrole-5,2-diyl))bis(morpholinomethanone) (**4j**) in $CDCl_3$ at 376 MHz

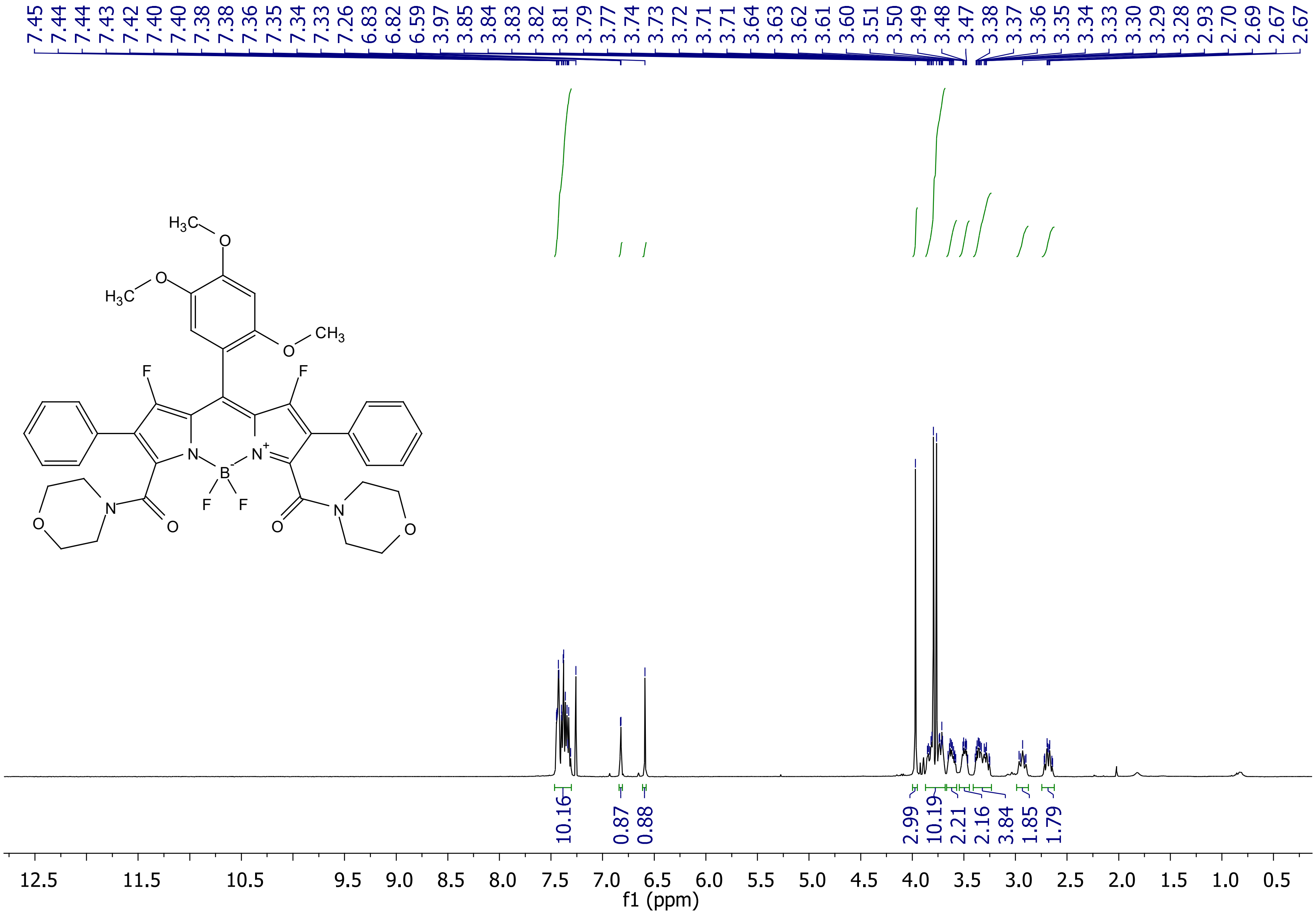

^{1}H NMR spectrum of 1,5,5,9-tetrafluoro-3,7-di(morpholine-4-carbonyl)-2,8-diphenyl-10-(2,4,5-trimethoxyphenyl)-5*H*-dipyrrolo[1,2-c:2',1'-f][1,3,2]diazaborinin-4-ium-5-uide (**5j**) in $CDCl_3$ at 400 MHz

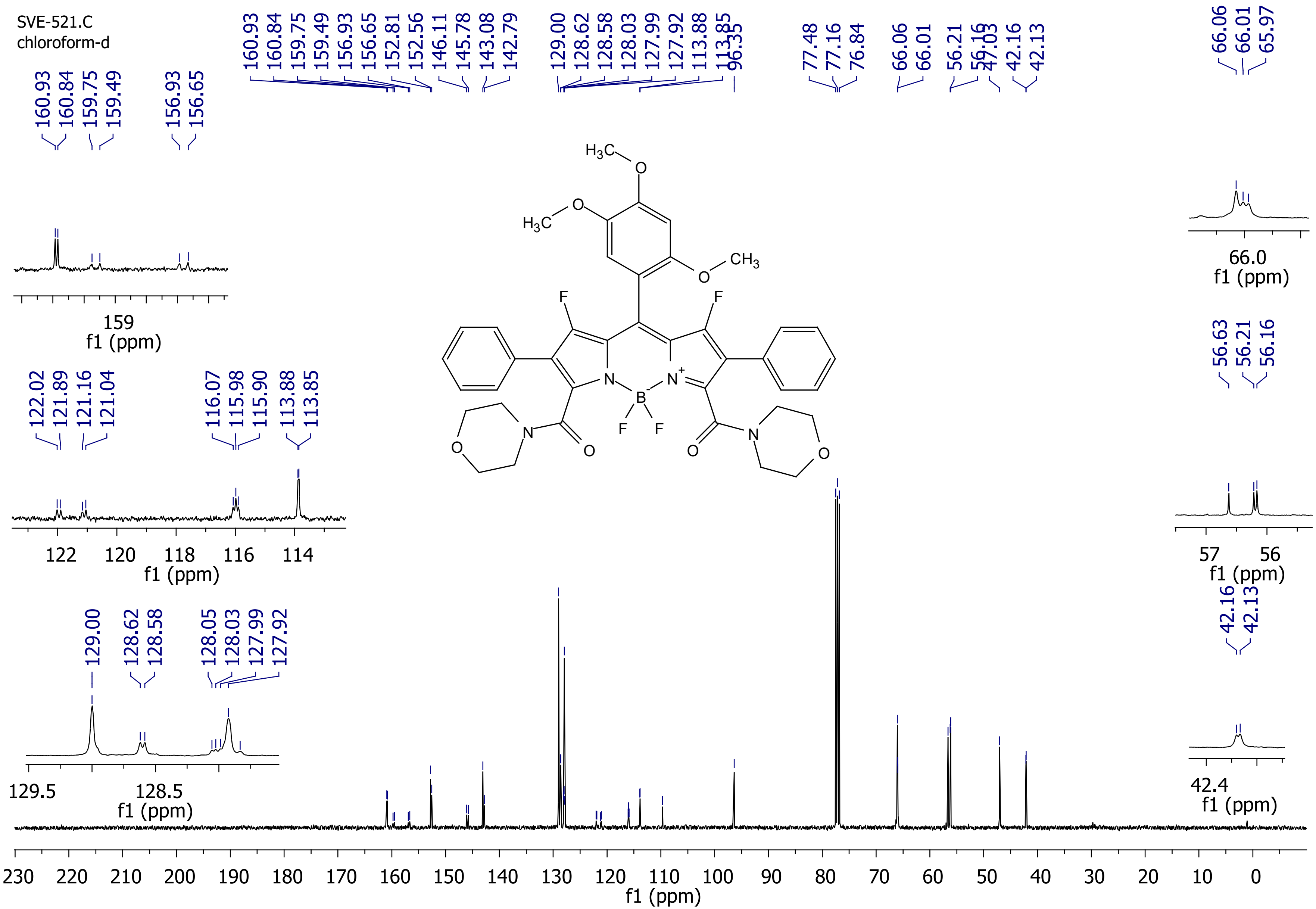

$^{13}C\{^1H\}$ NMR spectrum of 1,5,5,9-tetrafluoro-3,7-di(morpholine-4-carbonyl)-2,8-diphenyl-10-(2,4,5-trimethoxyphenyl)-5*H*-dipyrrolo[1,2-c:2',1'-f][1,3,2]diazaborinin-4-ium-5-uide (**5j**) in $CDCl_3$ at 100 MHz

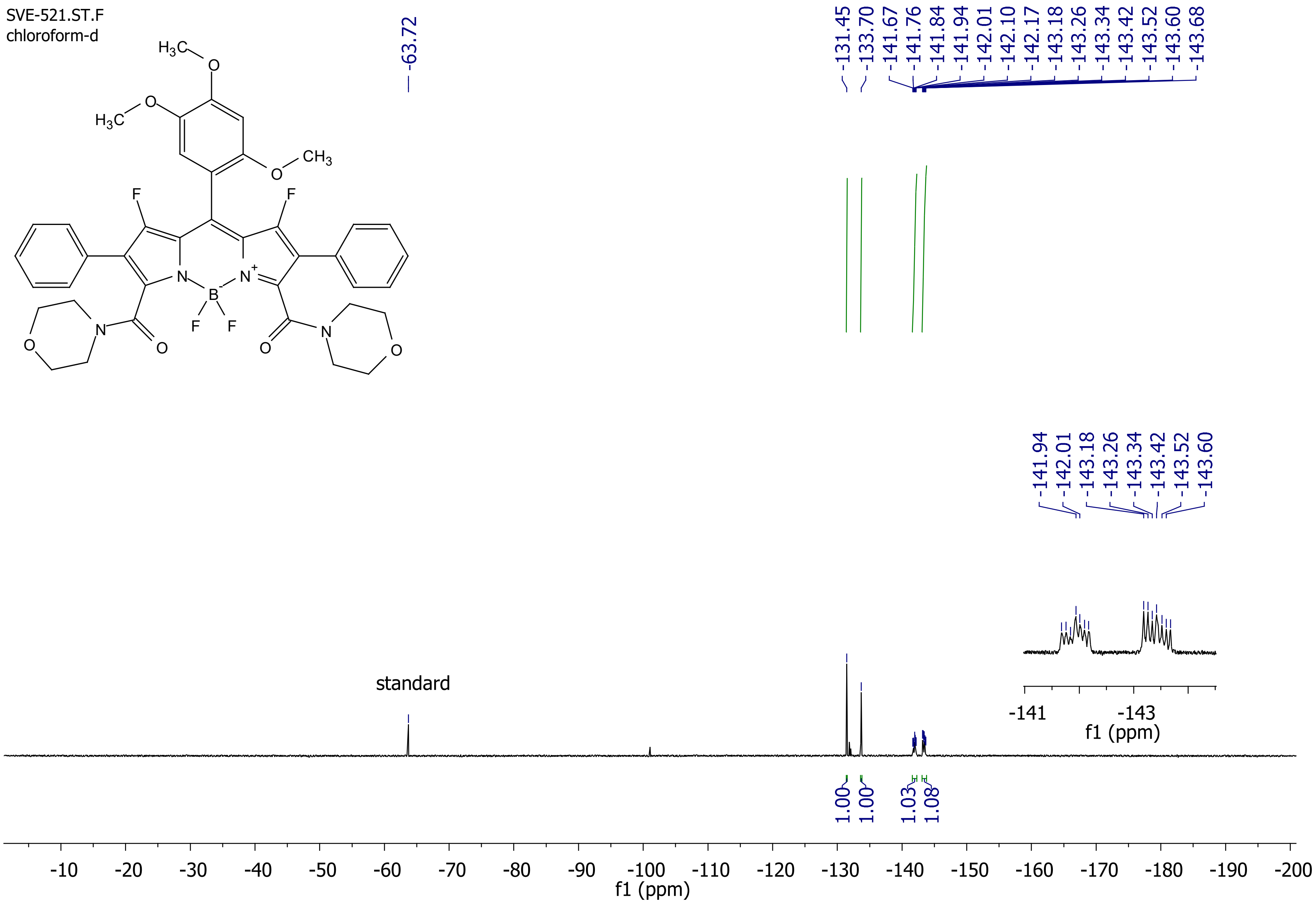

^{19}F NMR spectrum of 1,5,5,9-tetrafluoro-3,7-di(morpholine-4-carbonyl)-2,8-diphenyl-10-(2,4,5-trimethoxyphenyl)-5*H*-dipyrrolo[1,2-c:2',1'-f][1,3,2]diazaborinin-4-ium-5-uide (**5j**) in $CDCl_3$ at 376 MHz

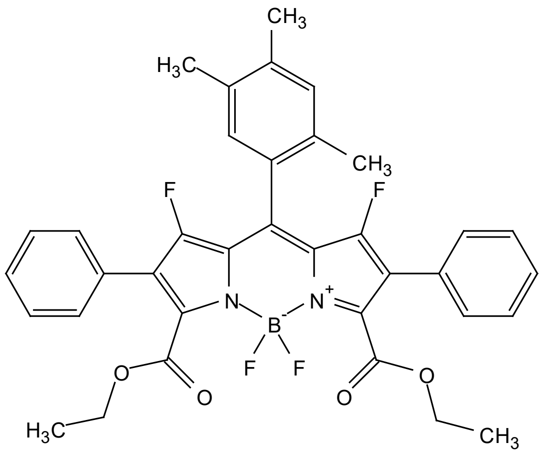

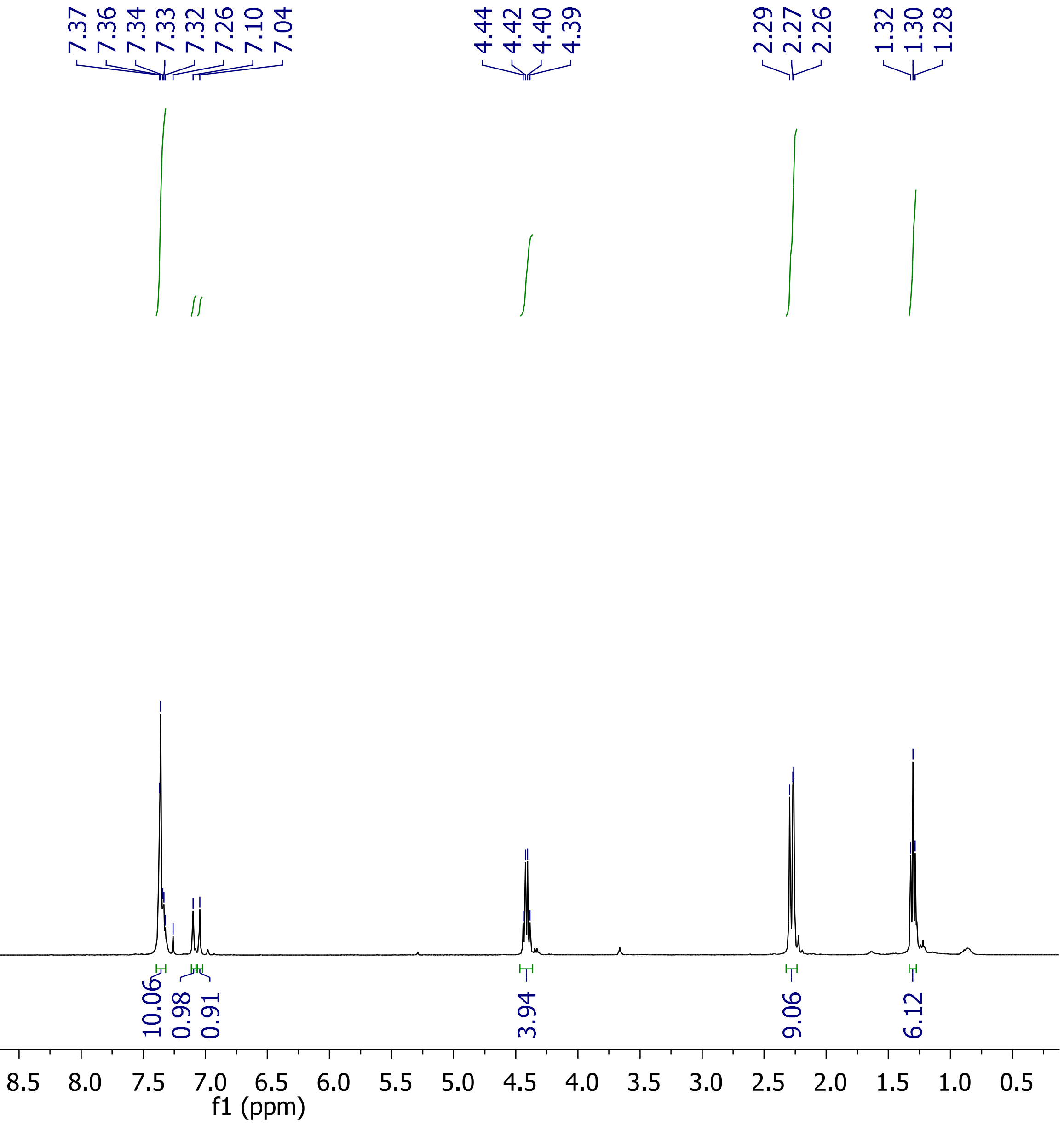

^{1}H NMR spectrum of 3,7-bis(ethoxycarbonyl)-1,5,5,9-tetrafluoro-2,8-diphenyl-10-(2,4,5-trimethylphenyl)-5*H*-dipyrrolo[1,2-c:2',1'-f][1,3,2]diazaborinin-4-ium-5-uide (**5k**) in $CDCl_3$ at 400 MHz

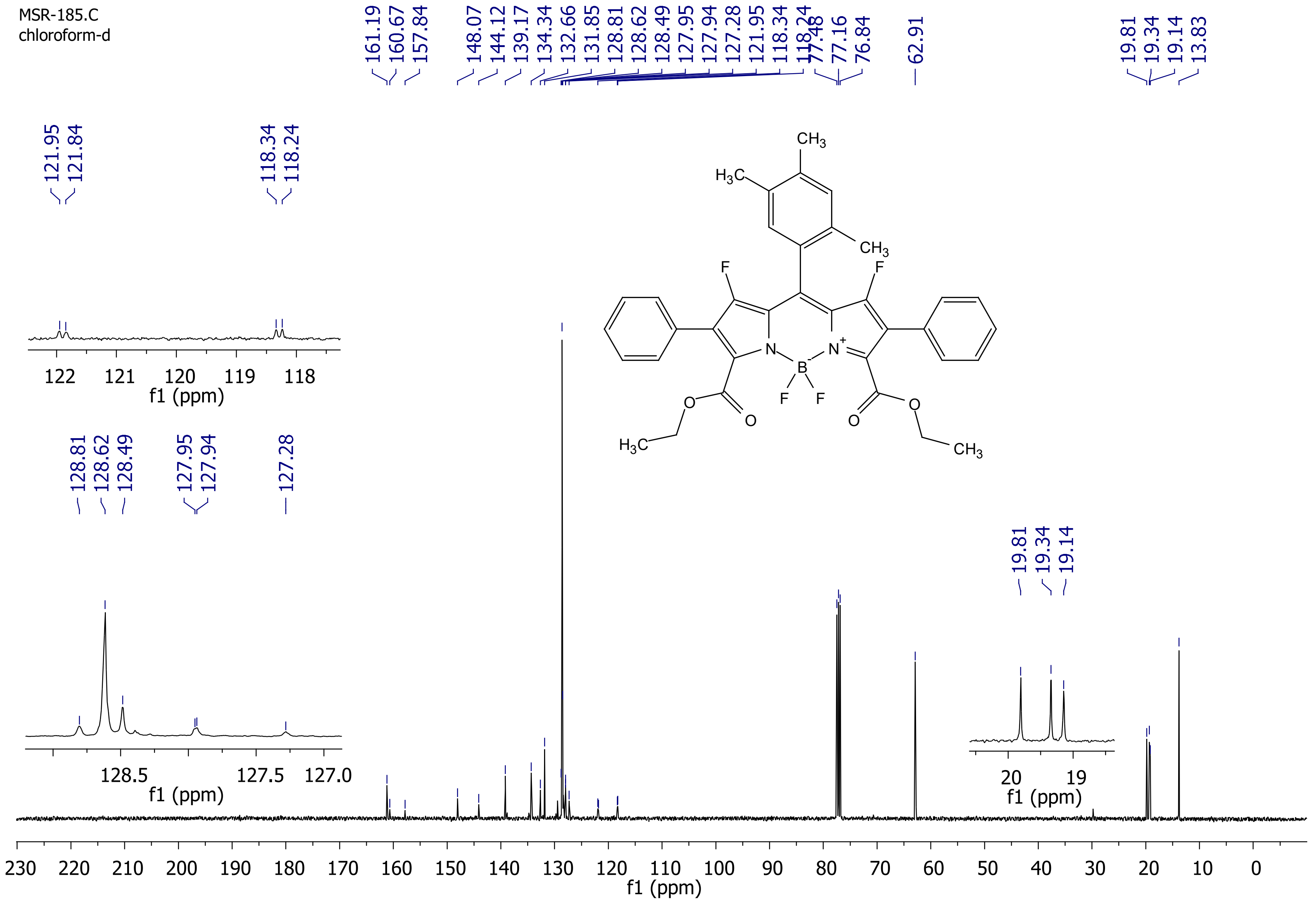

$^{13}C\{^{1}H\}$ NMR spectrum of 3,7-bis(ethoxycarbonyl)-1,5,5,9-tetrafluoro-2,8-diphenyl-10-(2,4,5-trimethylphenyl)-5*H*-dipyrrolo[1,2-c:2',1'-f][1,3,2]diazaborinin-4-ium-5-uide (**5k**) in $CDCl_3$ at 100 MHz

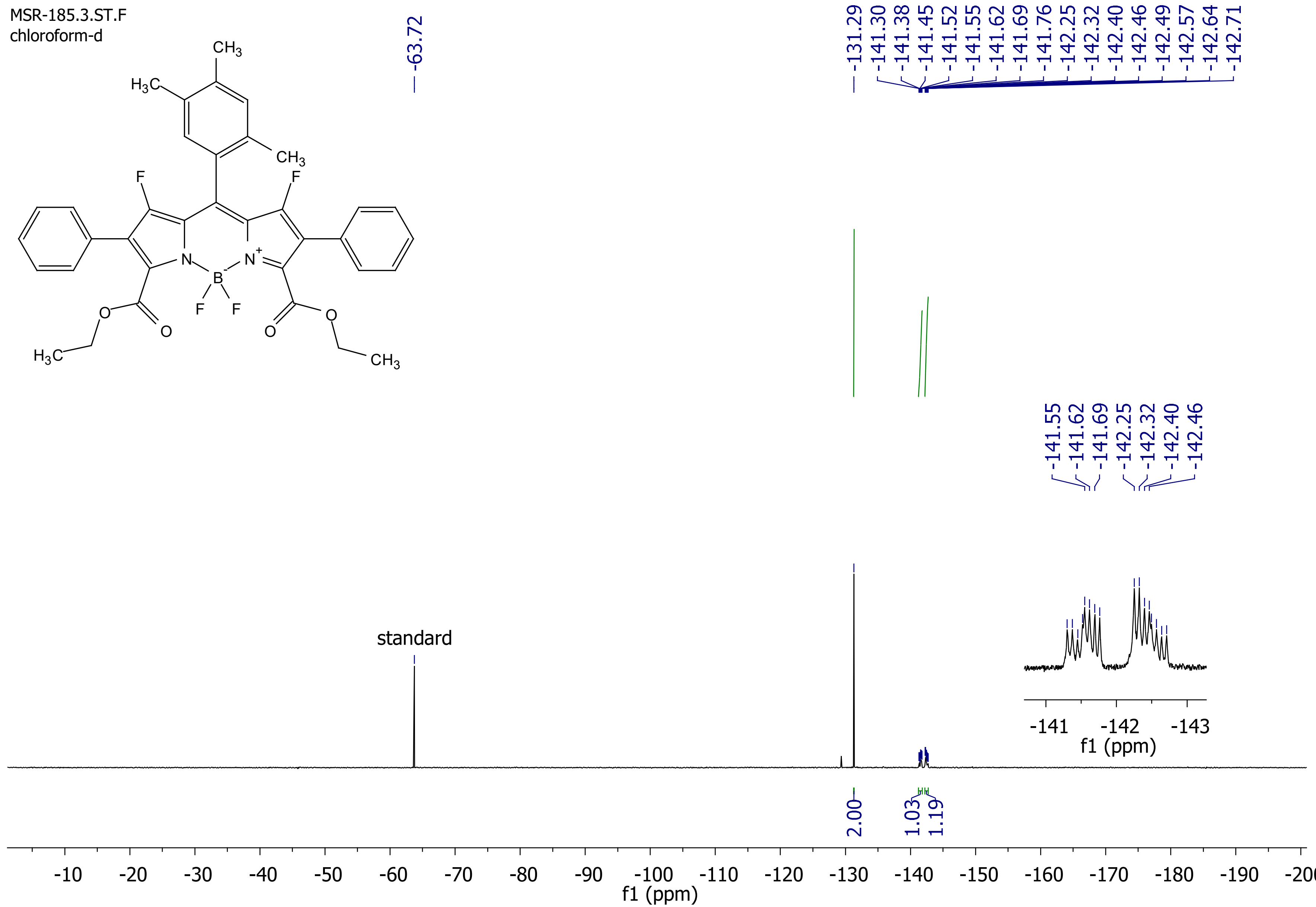

^{19}F NMR spectrum of 3,7-bis(ethoxycarbonyl)-1,5,5,9-tetrafluoro-2,8-diphenyl-10-(2,4,5-trimethylphenyl)-5*H*-dipyrrolo[1,2-c:2',1'-f][1,3,2]diazaborinin-4-ium-5-uide (**5k**) in $CDCl_3$ at 376 MHz
